# X-ray liquidography decodes complex motions in azobenzene isomerization

**DOI:** 10.1038/s41586-026-11068-4

**Published:** 2026-09-30

**Authors:** Jungmin Kim, Hosung Ki, Seonggon Lee, Alekos Segalina, Yunbeom Lee, Hyotcherl Ihee

**Affiliations:** 1 Department of Chemistry, Korea Advanced Institute of Science and Technology (KAIST), Daejeon, Republic of Korea; 2 Center for Advanced Reaction Dynamics (CARD), Institute for Basic Science (IBS), Daejeon, Republic of Korea

**Keywords:** Chemical physics, Physical chemistry, Chemistry, Reaction kinetics and dynamics

## Abstract

Capturing ultrafast structural rearrangements of organic molecules in solution remains a central challenge, with isomerization being a key example. Yet, despite decades of studies, a detailed atomic-level structural understanding of how isomerization occurs remains elusive for most molecules. Even for azobenzene, a textbook case of *trans*–*cis* isomerization with its deceptively simple structure of two phenyl rings linked by an azo bridge, the mechanism remains contentious. Extensive experimental and theoretical studies^1–33^ have proposed disparate pathways, including rotation^1–15^, inversion^16–19^, hula twist^20,21^ and inversion-assisted rotation^22–30^, without reaching consensus owing to the lack of direct structural evidence. Here we apply femtosecond X-ray liquidography to *trans*-azobenzene in solution and resolve, with atomic-level precision, the molecular structures of two transient intermediates bridging the *trans* and *cis* forms. The unveiled structures reveal that the *trans*-to-*cis* conversion proceeds through a sequence of distinct motions, initiated by C–N torsion, a motion that has received little attention in previous studies, and subsequently dominated by N–N rotation. This work provides structural insights into the volume-conserving nature of azobenzene isomerization and establishes X-ray liquidography as a versatile tool for molecular filming of structural dynamics in solutes lacking heavy atoms, overcoming the limitations imposed by dominant solvent scattering.

## Abstract (web summary)

Time-resolved X-ray liquidography films the structural changes of azobenzene during *trans*-to-*cis* isomerization in solution with atomic-level precision.

## Main

**Fig. 1:**
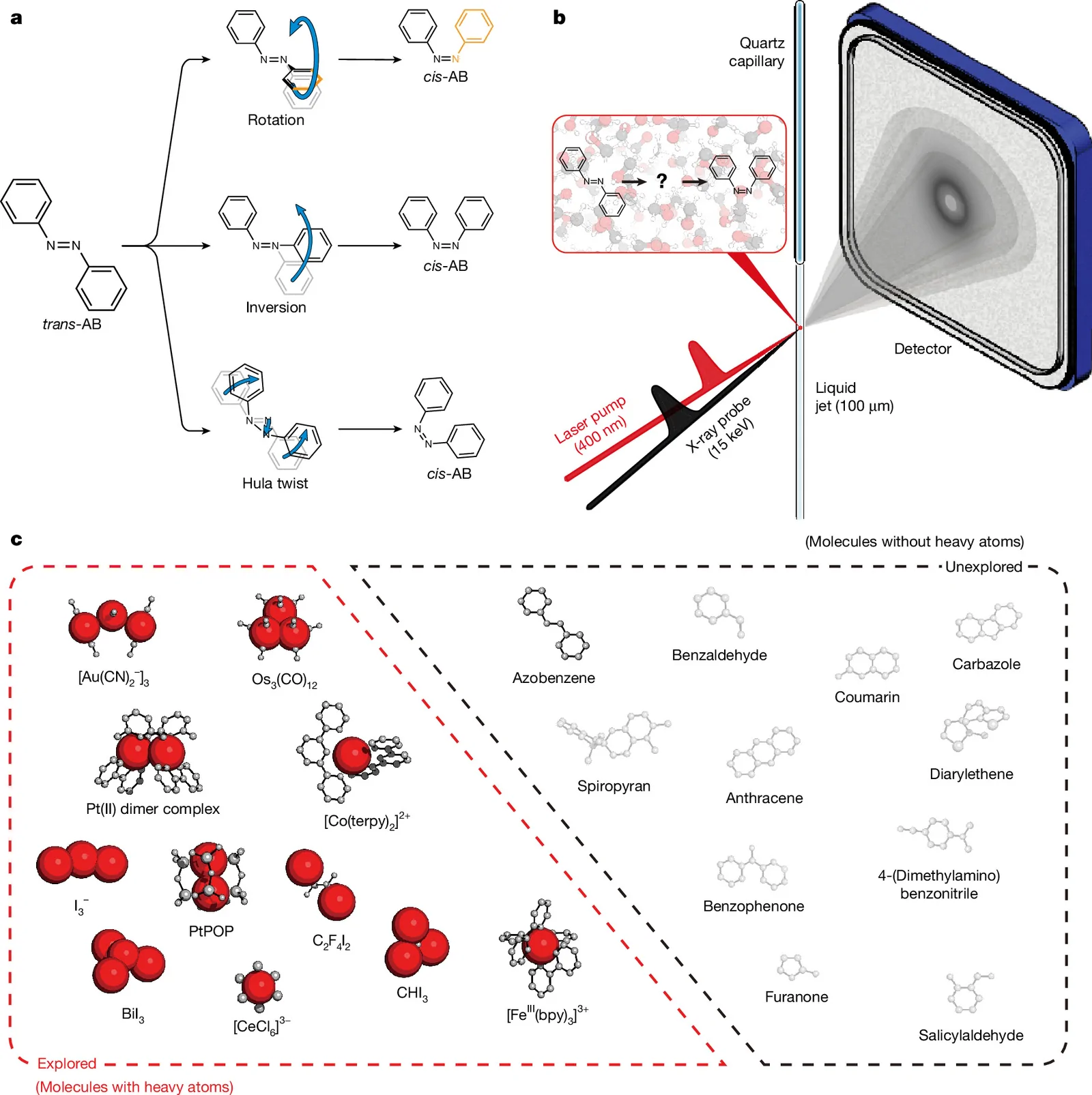
Schematic illustration of the isomerization mechanisms, the fs-TRXL experiment, and the comparison of molecular scopes previously accessible and newly enabled in this work. **a**, Three representative mechanisms proposed for the *trans*-to-*cis* isomerization of *trans*-AB from previous studies. Each mechanism is described in Supplementary Note 1. **b**, Schematic of the fs-TRXL experiment performed in this study (see Methods for details). **c**, Small molecules classified into two groups: one containing heavy atoms and the other having no heavy atoms. Heavy atoms are shown in red. Owing to the strong scattering from heavy atoms, molecules in the first group produce pronounced solute scattering signals and have been routinely investigated by TRXL to uncover their structural dynamics, whereas no successful TRXL study has yet been reported for those in the second group. In this work, azobenzene, a representative of the second group, has been successfully studied by fs-TRXL.

Three representative mechanisms for the nπ* photoisomerization (S_0_ → S_1_) of *trans*-azobenzene (*trans*-AB), proposed in extensive experimental (Extended Data Table 1) and theoretical (Extended Data Table 2) studies, are illustrated in Fig. 1a. Notably, even studies using identical experimental techniques have yielded conflicting interpretations, and theoretical predictions vary widely (Extended Data Table 2). This ongoing ambiguity arises primarily from the lack of direct structural evidence for transient intermediates, which spectroscopy alone cannot resolve. To overcome this limitation and directly determine both the transient structures and reaction kinetics, we used femtosecond time-resolved X-ray liquidography (fs-TRXL), a structure-sensitive technique that retrieves molecular structural changes from time-resolved X-ray solution scattering patterns^34–40^. A 400-nm femtosecond laser pulse excited *trans*-AB in methanol to induce the nπ* transition. After a controlled time delay (*t*), a femtosecond X-ray pulse from an X-ray free-electron laser (XFEL) beamline probed the transient molecular structures (Fig. 1b). Thousands of laser-on and laser-off scattering images were collected at each delay, processed to obtain isotropic and anisotropic components^36^. The laser-off components were then subtracted from the laser-on components, and the resulting difference signals were averaged to yield the isotropic and anisotropic difference scattering curves, denoted as Δ*S*_iso_(*q*, *t*)^raw^ and Δ*S*_aniso_(*q*, *t*)^raw^, respectively. This procedure isolates the laser-induced difference scattering as a function of *q*, the magnitude of the momentum transfer vector. For visualization in the figures, difference curves are plotted as *q*Δ*S*(*q*, *t*) to enhance the high-*q* features associated with solute structural changes.

**Fig. 2:**
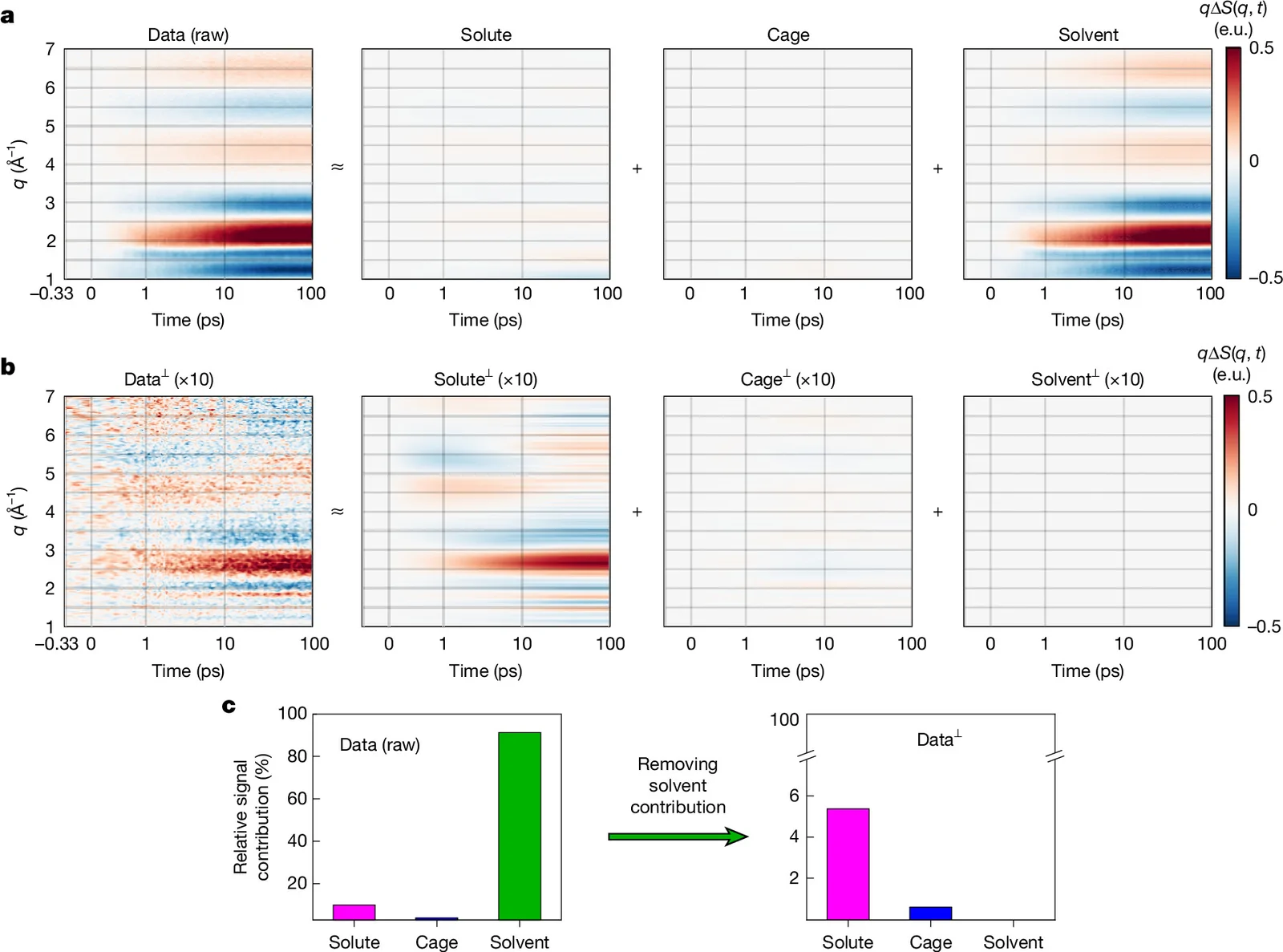
Contributions of solute, cage and solvent terms to the overall difference scattering curve for the *trans*-to-*cis* isomerization of azobenzene in methanol. **a**, The relative amplitudes and shapes of the solute, cage and solvent terms comprising the raw data (*q*Δ*S*_iso_(*q*, *t*)^raw^, given in electron units, e.u.). The raw data are dominated by the solvent term. **b**, The relative amplitudes and shapes of the solute, cage and solvent terms comprising the PEPC-treated data (*q*Δ*S*^⊥^_iso_(*q*, *t*)). All four panels are scaled by a factor of 10 to enhance visibility, as indicated in each panel heading. The solvent contribution is absent. **c**, Comparison of the relative contributions from solute, cage and solvent terms. The contributions of these terms, multiplied by *q*, are quantified by calculating the area under their respective curves. The dominance of the solvent contribution to the difference scattering signal (over 90% of the total signal from *trans*-AB solution) presents a considerable challenge in extracting the subtle structural changes of the solute molecules. This work successfully isolates and reconstructs the structural information of the solute, demonstrating the sensitivity and power of TRXL analysis even in the presence of overwhelming background signals. Source data

**Extended Data Fig. 1:**
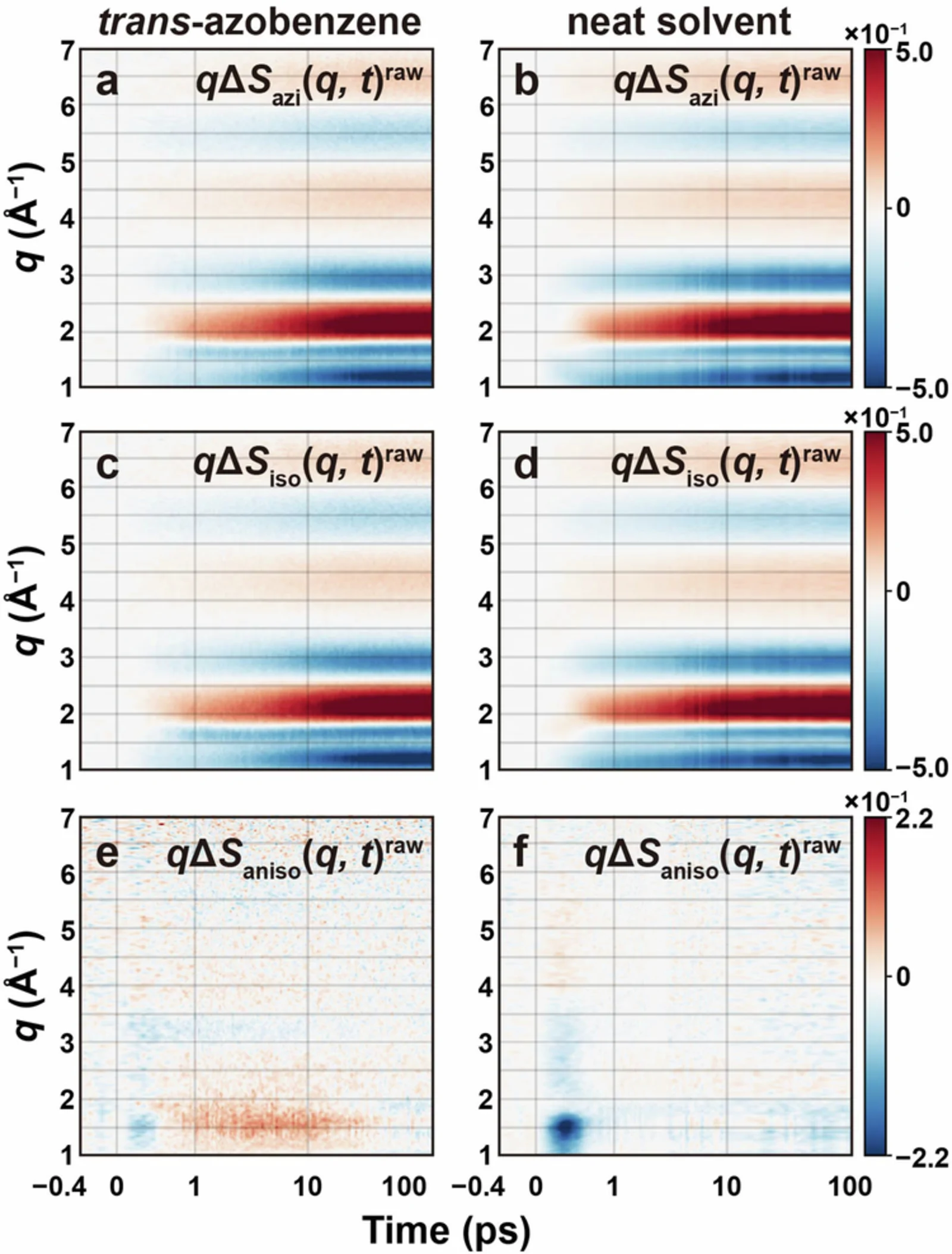
Comparison of femtosecond time-resolved X-ray liquidography (fs-TRXL) data for the *trans*-AB solution and neat solvent. **a,b**, Azimuthally averaged difference scattering signals, *q*Δ*S*_azi_(*q*, *t*)^raw^, for the *trans*-AB solution (**a**) and the neat solvent (**b**). The superscript “raw” indicates that the data have not been subjected to PEPC treatment, meaning that solvent contributions are still present. **c**,**d**, Isotropic difference scattering signals, *q*Δ*S*_iso_(*q*, *t*)^raw^, for the *trans*-AB solution (**c**) and the neat solvent (**d**). **e**,**f**, Anisotropic difference scattering signals, *q*Δ*S*_aniso_(*q*, *t*)^raw^, for the *trans*-AB solution (**e**) and neat solvent (**f**). The neat solvent data in panels **b** and **d** were scaled so that the signal at 100 ps matches that of the *trans*-AB solution data, and the same scaling factor was applied to panel **f**. Source data

Although TRXL effectively probes liquid-phase structural dynamics^34–40^, its small-molecule application has been largely limited to those containing heavy atoms, which enhance scattering contrast relative to the scattering signals from solvent molecules (Fig. 1c). Applying TRXL to small molecules without heavy atoms is challenging, as demonstrated by our experiment on azobenzene. Comparison of *q*Δ*S*_iso_(*q*, *t*)^raw^ and *q*Δ*S*_aniso_(*q*, *t*)^raw^ for azobenzene solution and neat solvent (Extended Data Fig. 1) shows nearly identical profiles, indicating that the signals of the azobenzene solution are dominated by the solvent heating terms. Figure 2a shows the solute, cage and solvent terms constituting *q*Δ*S*_iso_(*q*, *t*)^raw^. The *q*Δ*S*_iso_(*q*, *t*)^raw^ is nearly identical to the solvent term, whereas the solute and cage contributions are virtually negligible relative to the dominant solvent term. This overwhelming solvent background presents a major challenge in data analysis and explains why successful TRXL studies of small molecules without heavy atoms have been lacking so far.

## Overcoming the solvent background

**Extended Data Fig. 2:**
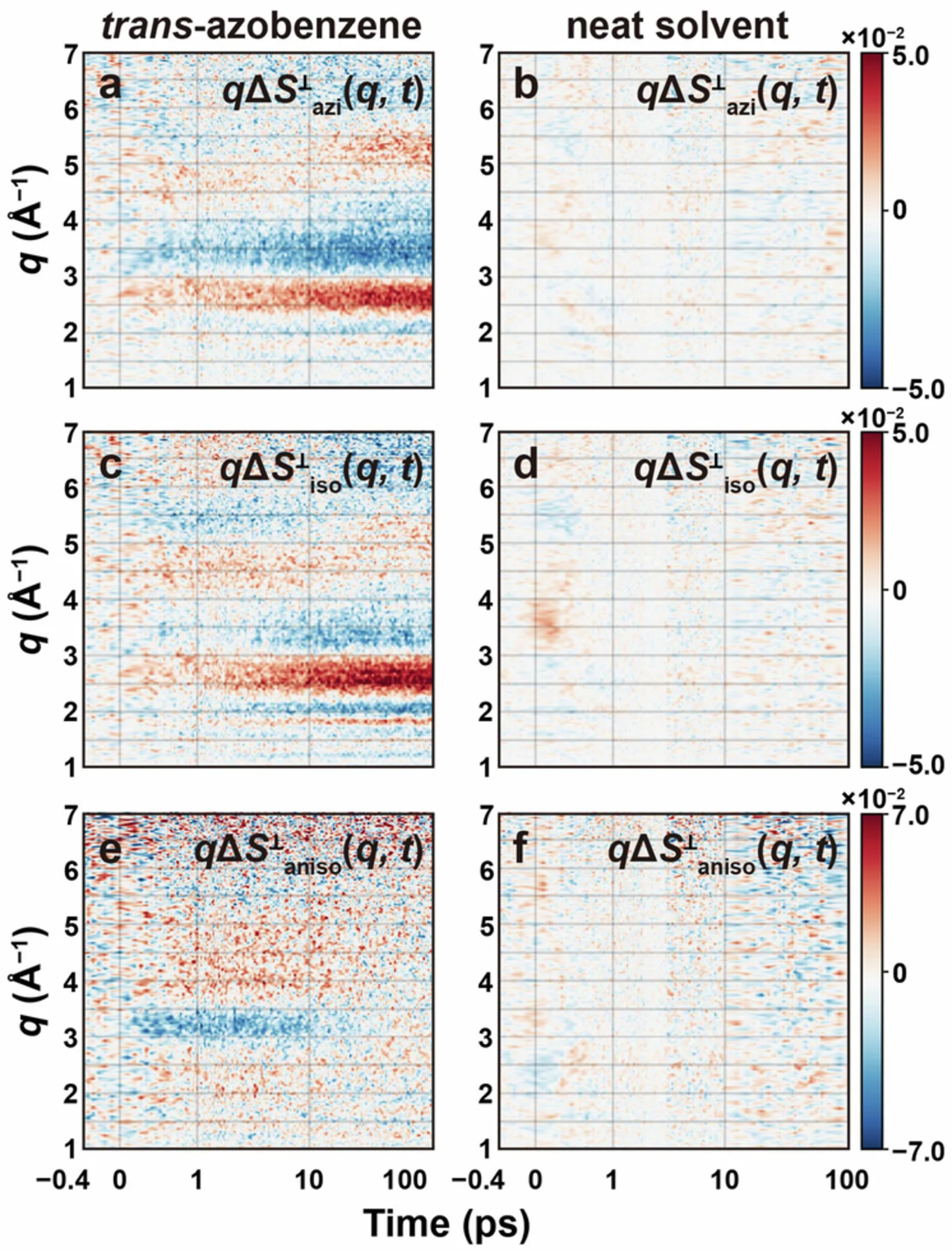
Solvent-independent fs-TRXL data for the *trans*-AB solution and neat solvent. **a,b**, *q*Δ*S*^⊥^_azi_(*q*, *t*) for the *trans*-AB solution (**a**) and neat solvent (**b**). **c**,**d**, *q*Δ*S*^⊥^_iso_(*q*, *t*) for the *trans*-AB solution (**c**) and neat solvent (**d**). **e**,**f**, *q*Δ*S*^⊥^_aniso_(*q*, *t*) for the *trans*-AB solution (**e**) and neat solvent (**f**). Source data

**Extended Data Fig. 3:**
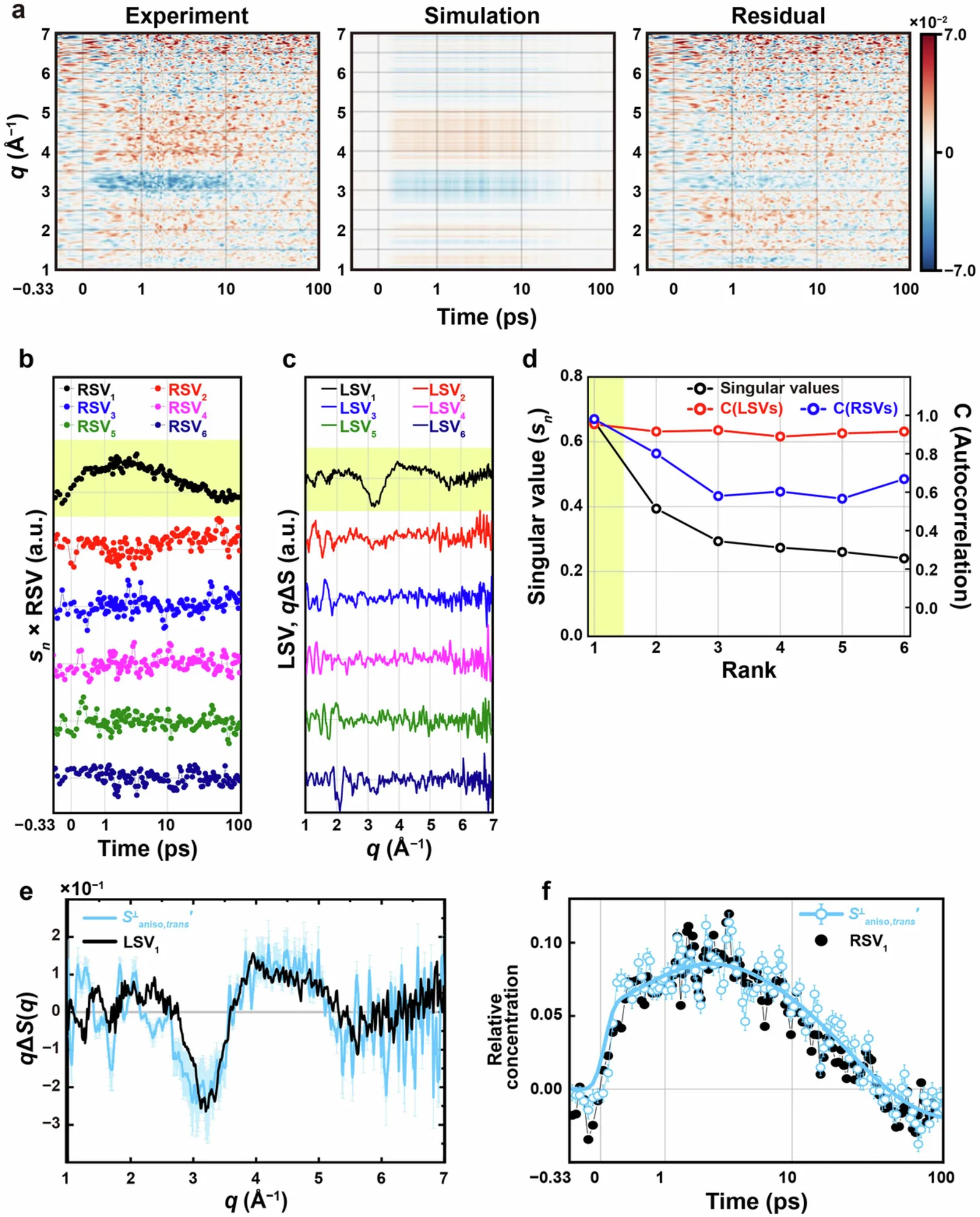
Spatiotemporal analysis of anisotropic fs-TRXL data for *trans*-AB. **a**, Experimental (left), simulated (middle), and residual (right) solvent-independent anisotropic fs-TRXL data. The simulated data are the LCF reconstruction, given by the product of *S*^⊥^_aniso__,__*trans*_′(*q*) (blue curve in **e**) and its time-dependent contribution obtained from the LCF (blue circles in **f**). **b–d**, SVD analysis of the solvent-independent anisotropic fs-TRXL data. **b**, The six leading RSVs, each scaled by its corresponding singular value *s*_*n*_ so that its amplitude reflects its contribution to the total signal. **c**, The six corresponding LSVs, each plotted as *q·*LSV(*q*) to emphasize the signal in the high-*q* region (*q* > 2 Å^−1^). **d**, Singular values (black dots and line, left axis) and the autocorrelation values of the LSVs (red dots and line, right axis) and RSVs (blue dots and line, right axis) as a function of component rank. A component is distinguishable from noise only if it has a large singular value together with high LSV and RSV autocorrelations; the first component (yellow shading) alone satisfies all of these criteria, identifying it as the sole substantial contributor to the anisotropic signal. **e**, Experimental *q·*LSV_1_(*q*) (black curve) and the simulated anisotropic scattering curve of *trans*-AB, *S*^⊥^_aniso__,__*trans*_′(*q*) (blue curve), which arises from the photoselected ground-state depletion (hole). *S*^⊥^_aniso__,__*trans*_′(*q*) was obtained by applying the standard expression for a photoselectively excited ensemble (equation (S18); Supplementary Method 7) to the *trans*-AB structure and then applying the PEPC procedure. In contrast to a conventional LCF, in which the basis curve is fixed and only its concentration is fitted, here the shape of *S*^⊥^_aniso__,__*trans*_′(*q*) is also optimized: the orientation of the transition dipole within the molecular frame is a global fitting parameter, common to all time delays, refined jointly with the time-dependent concentration *c*(*t*) by minimizing the chi-square between the measured *S*^⊥^_aniso_(*q*, *t*) and the reconstruction *S*^⊥^_aniso__,__*trans*_′(*q*)·*c*(*t*) (the Experiment and Simulation maps in **a**). The light-blue vertical bars represent the 1*σ* (standard deviation) uncertainty of *S*^⊥^_aniso__,__*trans*_′(*q*), obtained by a parametric bootstrap of the LCF analysis. **f**, Time-resolved relative contribution *c*(*t*) of *S*^⊥^_aniso__,__*trans*_′(*q*) (blue circles), superimposed with the 1st RSV (black circles) for comparison. Error bars represent 1*σ* uncertainties obtained from a parametric bootstrap of the LCF analysis. *c*(*t*) is fitted with a multiexponential function convolved with the Gaussian IRF (blue solid line), yielding time constants of 1.2 ± 0.2 and 24.6 ± 1.3 ps with an IRF-limited rise. Source data

To address this challenge, we used a data analysis methodology called projection to extract the perpendicular component (PEPC)^41^. The PEPC transforms the measured scattering data into another data space orthogonal to the known solvent components, producing solvent-independent signals whose features are determined solely by solute structural changes, regardless of variations in solvent response. This allows direct analysis of the solute-related term without interference from solvent contributions and enables extraction of solute-specific kinetics and structural information. Applying PEPC to Δ*S*_iso_(*q*, *t*)^raw^ and Δ*S*_aniso_(*q*, *t*)^raw^ effectively extracted the weak solute-related signal embedded in the dominant solvent background, a task that would have been difficult without this approach. The resulting solvent-independent curves multiplied by *q*, *q*Δ*S*^⊥^_iso_(*q*, *t*) and *q*Δ*S*^⊥^_aniso_(*q*, *t*), are shown in Extended Data Fig. 2, where the superscript ⊥ denotes the component of the difference scattering signal that is projected onto the subspace orthogonal to the known solvent contributions by the PEPC procedure. These curves retain clear time-dependent features that reflect the structural dynamics of *trans*-AB. Figure 2b shows the decomposition of *q*Δ*S*^⊥^_iso_(*q*, *t*) into the solute, cage and solvent terms. Unlike *q*Δ*S*_iso_(*q*, *t*)^raw^, which is dominated by the solvent term, *q*Δ*S*^⊥^_iso_(*q*, *t*) comprises only the solute and cage contributions. For validation, the same PEPC procedure was also applied to the neat solvent data to confirm that the transformation effectively removes solvent contributions. As expected, *q*Δ*S*^⊥^_iso_(*q*, *t*) from the neat solvent shows no distinct features, verifying that the PEPC treatment yields solvent-independent signals. The anisotropic data show similar trends: *q*Δ*S*^⊥^_aniso_(*q*, *t*) for the *trans*-AB solution shows a discernible pattern, whereas that of the neat solvent remains featureless beyond noise (see Supplementary Note 2 and Extended Data Fig. 3 for a detailed discussion). As Δ*S*^⊥^_aniso_(*q*, *t*) for the *trans*-AB solution primarily reflects the rotational dephasing of ground-state molecules, which is unrelated to the *trans*-to-*cis* photoisomerization process of interest, subsequent analyses focus on the isotropic component, Δ*S*^⊥^_iso_(*q*, *t*).

## Identification of the kinetic model

**Fig. 3:**
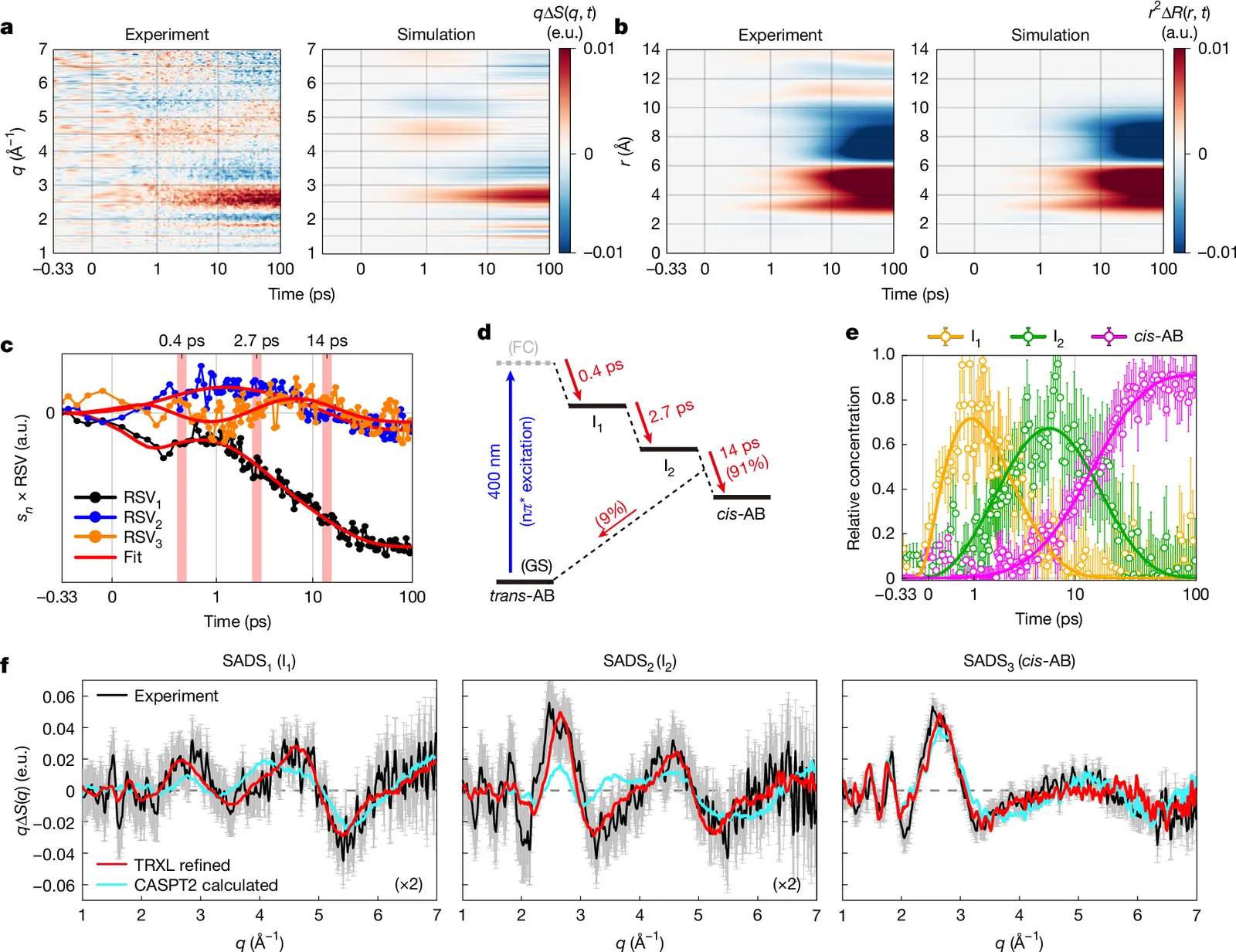
Spatiotemporal analysis of TRXL data for *trans*-AB using kinetic analysis and Fourier sine transformation. **a**,**b**, Solvent-independent experimental and simulated TRXL data, displayed as *q*Δ*S*^⊥^(*q*) (reciprocal space, in electron units, e.u.) and *r*^2^Δ*R*(*r*) (real space, in arbitrary units, a.u.), respectively. *r* and Δ*R*(*r*) denote the interatomic distance in real space and the difference radial distribution function, respectively. The simulated data are reconstructed as the product of the simulated SADSs (SADS_*n*_′^⟂^; TRXL-refined red curves in **f**) and the kinetic-model concentration profiles (*m*_*n*_(*t*); solid lines in **e**). **a**, Data displayed as *q*Δ*S*^⊥^(*q*) in reciprocal space, including both solute and cage contributions. **b**, Data displayed as *r*^2^Δ*R*(*r*) in real space, showing the solute-only component. **c**, Exponential fits (red curves) to the three substantial RSVs (black, blue and orange dots with lines) from the SVD analysis of Δ*S*^⊥^_iso_(*q*, *t*) from the azobenzene solution. *s*_n_ denotes the *n*th singular value for each RSV. **d**, Kinetic model constructed based on the number of reaction intermediates and time constants identified in the SVD analysis. FC and GS denote Franck–Condon and ground states, respectively. **e**, Time-resolved relative concentration profiles of intermediates I_1_ (orange) and I_2_ (green) and *cis*-AB (magenta), obtained from the kinetic model shown in **d**. Circles represent the best-fit relative populations determined by linear combination fitting (LCF) at each time delay (Supplementary Method 11). Error bars represent 1*σ* fitting uncertainties (standard errors) derived from the linear combination fitting analysis. **f**, Experimental (black) and simulated (red, TRXL-refined; cyan, CASPT2-calculated) SADSs corresponding to the kinetic model shown in **d**. SADS_1_ and SADS_2_ are scaled by a factor of 2 to enhance visibility. The grey vertical bars represent 1*σ* (standard deviation) uncertainties of the experimental SADSs. Source data

**Extended Data Fig. 4:**
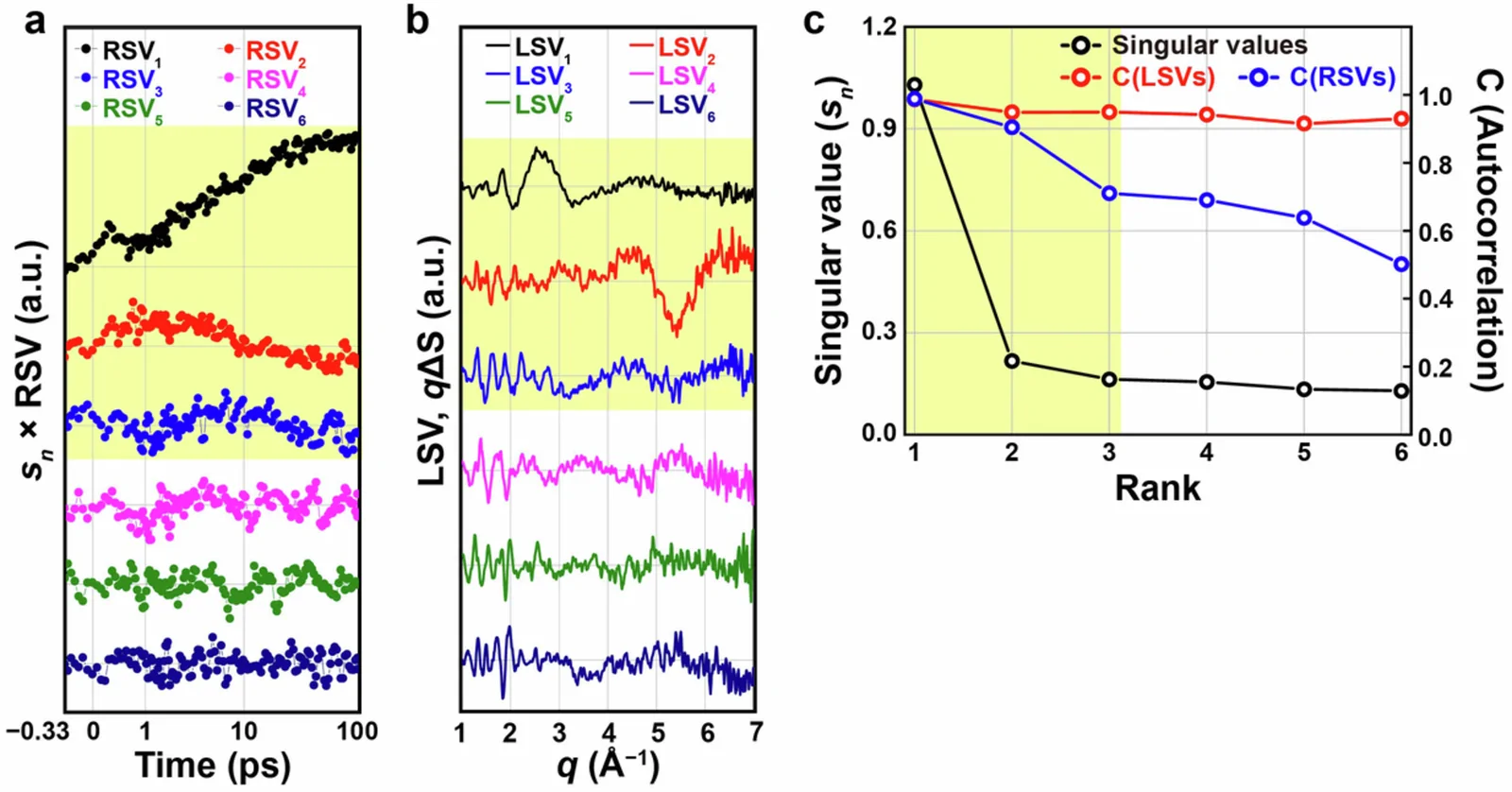
Singular value decomposition (SVD) analysis of solvent-independent isotropic fs-TRXL data for the *trans*-AB solution. **a**, The top six RSVs. Each RSV is scaled by its respective singular value to reflect its contribution to the signal. *s*_*n*_ denotes the *n*th singular value for each RSV. **b**, The top six LSVs. Each LSV was multiplied by *q* to plot *q*·LSV(*q*), emphasizing the region beyond *q* > 2 Å^−1^, where the solute signal becomes pronounced. **c**, Singular values (black dots and curve, left axis) and autocorrelation values for the LSVs (red dots and curve, right axis) and the RSVs (blue dots and curve, right axis). Three dominant components were identified as the main contributors to the signal and are highlighted in the yellow-shaded area. Source data

**Extended Data Fig. 5:**
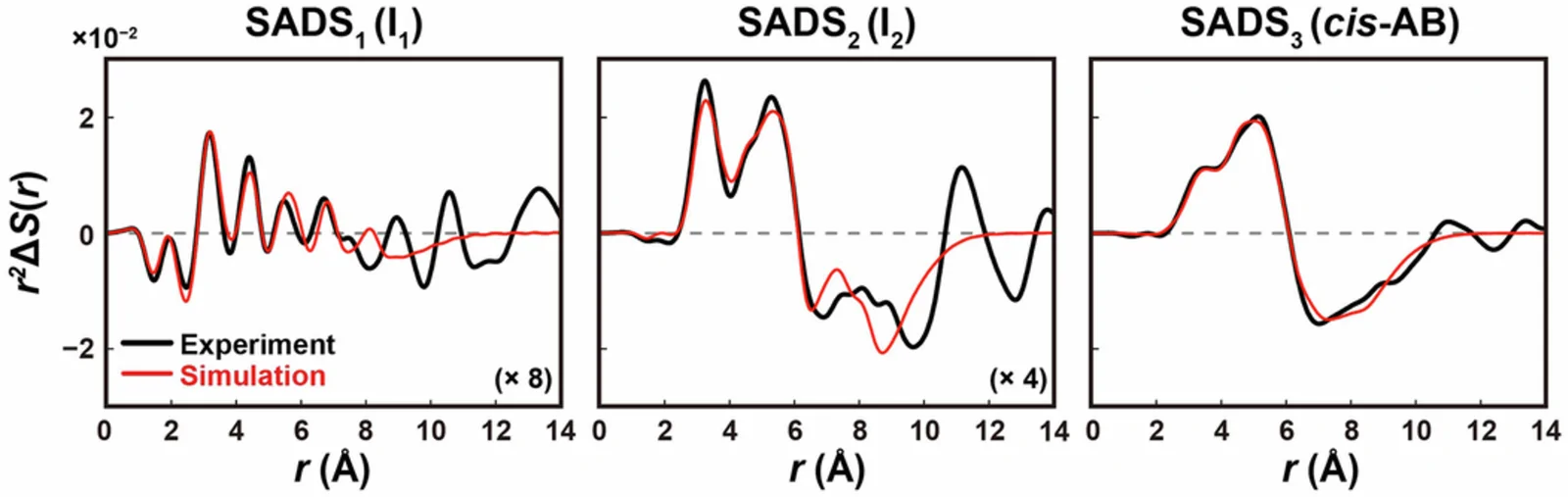
Solute-only SADSs in *r*-space. Experimental (black curves) and simulated (red curves) real-space SADSs are presented as *r*^2^SADS_solute_(*r*) in *r*-space. Negative amplitudes indicate interatomic distances depleted relative to *trans*-AB, while positive amplitudes indicate newly formed distances. For clarity, the SADS_1_ (I_1_) and SADS_2_ (I_2_) curves are vertically scaled by factors of 8 and 4, respectively, as indicated in each panel. A detailed discussion is provided in Supplementary Note 12. Source data

To extract detailed kinetic information, we conducted kinetic analysis on Δ*S*^⊥^_iso_(*q*, *t*) using singular value decomposition (SVD), which decomposes the data into time-invariant features in *q* space (left singular vectors (LSVs)), their relative contributions (singular values) and their time profiles (right singular vectors (RSVs))^42^, as detailed in Supplementary Method 1. The results of the SVD analysis of Δ*S*^⊥^_iso_(*q*, *t*) show that the experimental data consist of three substantial components (Extended Data Fig. 4). For quantitative analysis, the first three RSVs were globally fitted with a sum of exponential functions sharing common time constants across all three RSVs, yielding three distinct time constants: *τ*_1_ = 0.4 ± 0.2 ps, *τ*_2_ = 2.7 ± 1.0 ps and *τ*_3_ = 14 ± 4 ps (Fig. 3c). The observation of three distinct time constants is consistent with a three-step sequential model widely adopted in previous studies^4,21,32^, despite differences in the specific assignment of the intermediates (Supplementary Note 8). Guided by this framework (Fig. 3d), we assigned these time constants to the sequential transitions: the first intermediate (I_1_) forms from the Franck–Condon state with *τ*_1_, then converts to the second intermediate (I_2_) with *τ*_2_, and I_2_ relaxes to *cis*-azobenzene (*cis*-AB) with *τ*_3_. The branching yields at each step, determined from the scaling coefficients obtained in the subsequent structural refinement (Supplementary Method 12.3), indicate that the I_1_ → I_2_ conversion proceeds with unity yield within experimental uncertainty (100 ± 19%), whereas the I_2_ → *cis*-AB yield is 91 ± 7%, consistent with a predominantly sequential pathway. The concentrations of I_1_, I_2_ and *cis*-AB according to this kinetic model are shown in Fig. 3e. Consequently, Δ*S*^⊥^_iso_(*q*, *t*) can be represented by three species-associated difference scattering curves (SADSs; Fig. 3f), with SADS_1_ assigned to I_1_, SADS_2_ to I_2_, and SADS_3_ to *cis*-AB (Supplementary Method 2). The corresponding real-space SADSs, obtained by Fourier sine transform (Supplementary Method 10), are shown in Extended Data Fig. 5.

## Structural analysis of I_1_ and I_2_

With the kinetic framework established, structural analysis was carried out to elucidate the atomic motions underlying the observed intermediates. Previous quantum chemical studies have proposed various minima on the excited-state potential energy surface of azobenzene, but few have explicitly identified both I_1_ and I_2_, and the geometries proposed for these intermediates vary substantially among studies (Extended Data Table 2). Earlier calculations typically described the first intermediate as either purely rotational or concerted inversion geometry, without fully addressing potential coupling between multiple internal coordinates. To obtain a more reliable reference, we performed complete active space second-order perturbation theory (CASPT2) calculations with an extended active space and refined basis set (Methods). These revealed two distinct minima on the S_1_ potential energy surface, located between the Franck–Condon region of *trans*-AB and the conical intersection connecting to the S_0_ state. The identification of these two distinct minima provides independent theoretical support for the existence of two intermediates, consistent with our experimental assignment of I_1_ and I_2_.

As a first step towards structural validation, we compared the experimentally derived SADSs with their simulated counterparts from the CASPT2-calculated structures (Fig. 3f). To explore whether structures with better agreement with the experimental data exist, we then refined the structures of I_1_, I_2_ and *cis*-AB by minimizing the reduced chi-square (*χ*_*ν*_^2^), which quantifies the difference between the experimental and simulated SADSs, using the structural degrees of freedom associated with the candidate isomerization motions.

**Fig. 4:**
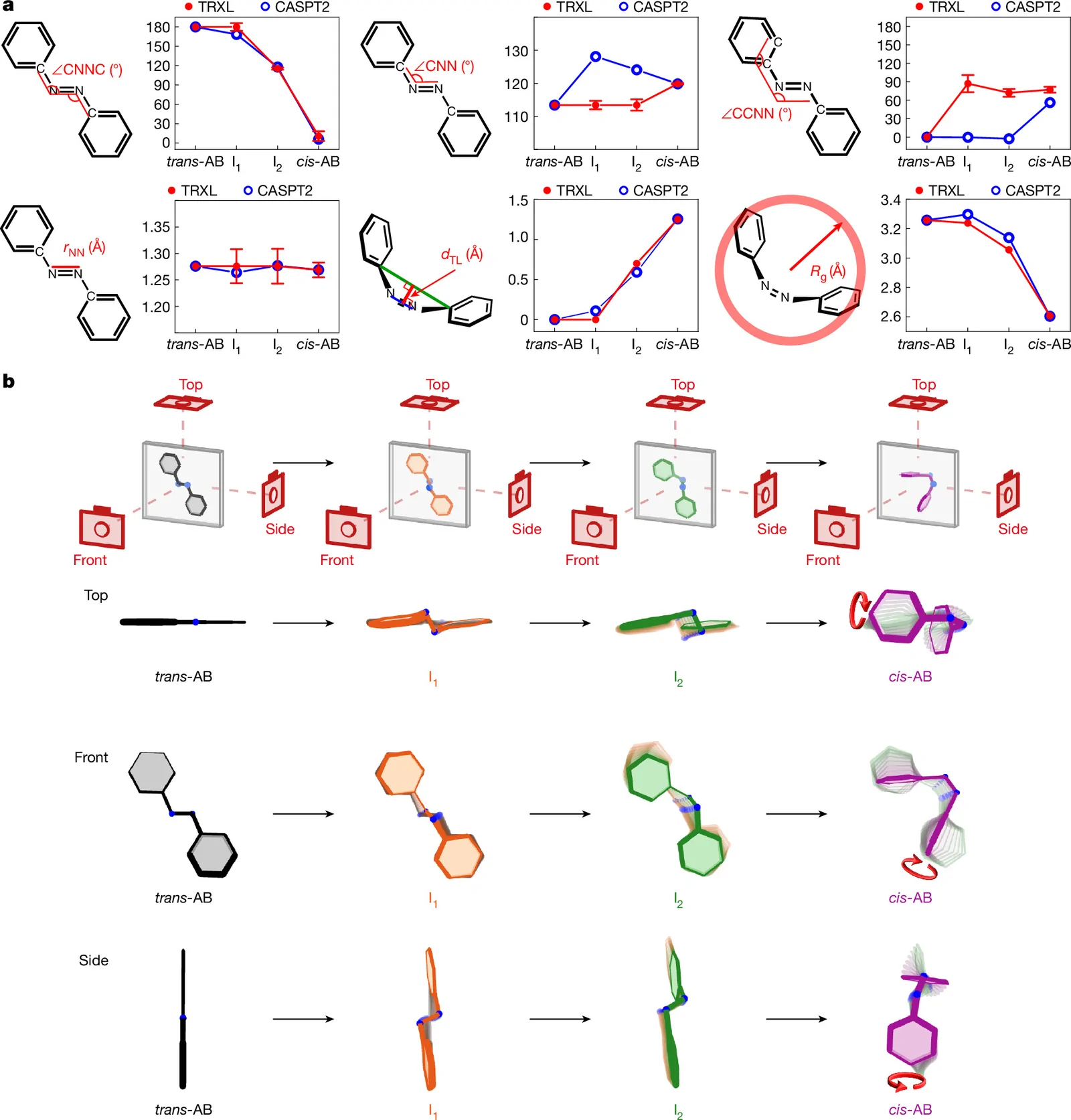
Structural dynamics of the *trans*-to-*cis* isomerization of *trans*-AB determined by TRXL. **a**, Changes in six key structural parameters (∠CNNC, ∠CNN, ∠CCNN, *r*_NN_, *d*_TL_ and *R*_g_) during the photoisomerization of *trans*-AB revealed by TRXL, compared with CASPT2 calculations. Structural parameters obtained from the TRXL-refined and CASPT2-calculated geometries are plotted in red and blue, respectively. Error bars on TRXL-refined parameters represent 1*σ* fitting uncertainties (standard errors) derived from the profile-likelihood analysis of the structural refinement. The definition of each structural parameter is shown in the molecular model on the left side of the corresponding panel. **b**, Three-dimensional molecular structures of *trans*-AB, I_1_, I_2_ and *cis*-AB obtained from TRXL, aligned along a volume-conserving pathway that minimizes the swept volume. A non-volume-conserving counterpart is shown in Supplementary Fig. 8. First panel: schematics illustrating the three viewing orientations (top, front and side) used in this figure: top (second panel), front (third panel) and side (bottom panel). To effectively convey the structural changes, the subpanels of I_1_, I_2_ and *cis*-AB include the preceding structure, with the transition depicted by a sequence of interpolated geometries. Red arrows serve to highlight the direction of motion where intricate. These views visualize the sequential structural transformations: bicycle-pedal motion of the azo group accompanying CNT from *trans*-AB to I_1_, coupled motions dominated by ROT from I_1_ to I_2_ and from I_2_ to *cis*-AB. Source data

The structural refinement was performed using as few degrees of freedom as possible. The three classical mechanisms illustrated in Fig. 1a describe direct *trans*-to-*cis* pathways without accounting for intermediates that might have greater motional flexibility. To allow such flexibility, we represented these mechanisms using combinations of four basic structural motions, each defined by a characteristic structural parameter shown in Fig. 4a: N–N rotation (ROT), inversion (INV), N–N bond-length change (NND) and C–N torsion (CNT). The most intuitive mechanism, N–N rotation, is represented simply as a change in the CNNC dihedral angle (∠CNNC), defined as ROT. For the inversion mechanism, we considered general inversion motions, termed INV, in which the ∠CNNs of both rings are allowed to vary independently in either direction. Finally, CNT describes independent torsion of each phenyl ring about its respective C–N bond axis (∠CCNN). Although CNT has received little attention in previous studies, it was added to the motional pool to account for the natural adjustment of the azo group or phenyl rings that may accompany the structural rearrangement. The hula-twist mechanism can be accounted for by a combination of all four motions.

For I_1_ and I_2_, we fitted SADS_1_ and SADS_2_ using a systematic scan for parsimonious motions (SSPM), starting with single-motion models and then expanding to test all possible combinations of two, three or four motions (Supplementary Method 12). Introducing more degrees of freedom naturally tends to lower the *χ*^2^ values. However, if the additional degrees of freedom are not truly relevant for describing the SADSs, the resulting reduction in *χ*^2^ would be expected to be relatively small. To objectively balance this trade-off between fit quality and model complexity, we used the Bayesian information criterion (BIC) to identify the motion(s) with the strongest statistical support while preventing over-parameterization (Supplementary Method 13). The SSPM procedure was performed iteratively. The best I_1_ structure from the first round of *trans*-AB → I_1_ SSPM was used to calculate the cage term for the subsequent round, and this cycle was repeated until the resulting best structure showed no substantial deviation from the input structure, indicating convergence. Once the final I_1_ structure was obtained, the I_1_ → I_2_ SSPM was similarly performed iteratively to obtain the I_2_ structure. For *cis*-AB, SSPM was not used. Instead, starting from the CASPT2-calculated *cis*-AB structure, we optimized its structural parameters to best fit SADS_3_. The refinement strategy for *cis*-AB differed from that for the intermediates: it focused on determining the optimal structure of the final product, rather than characterizing the structural motion from I_2_.

**Extended Data Fig. 6:**
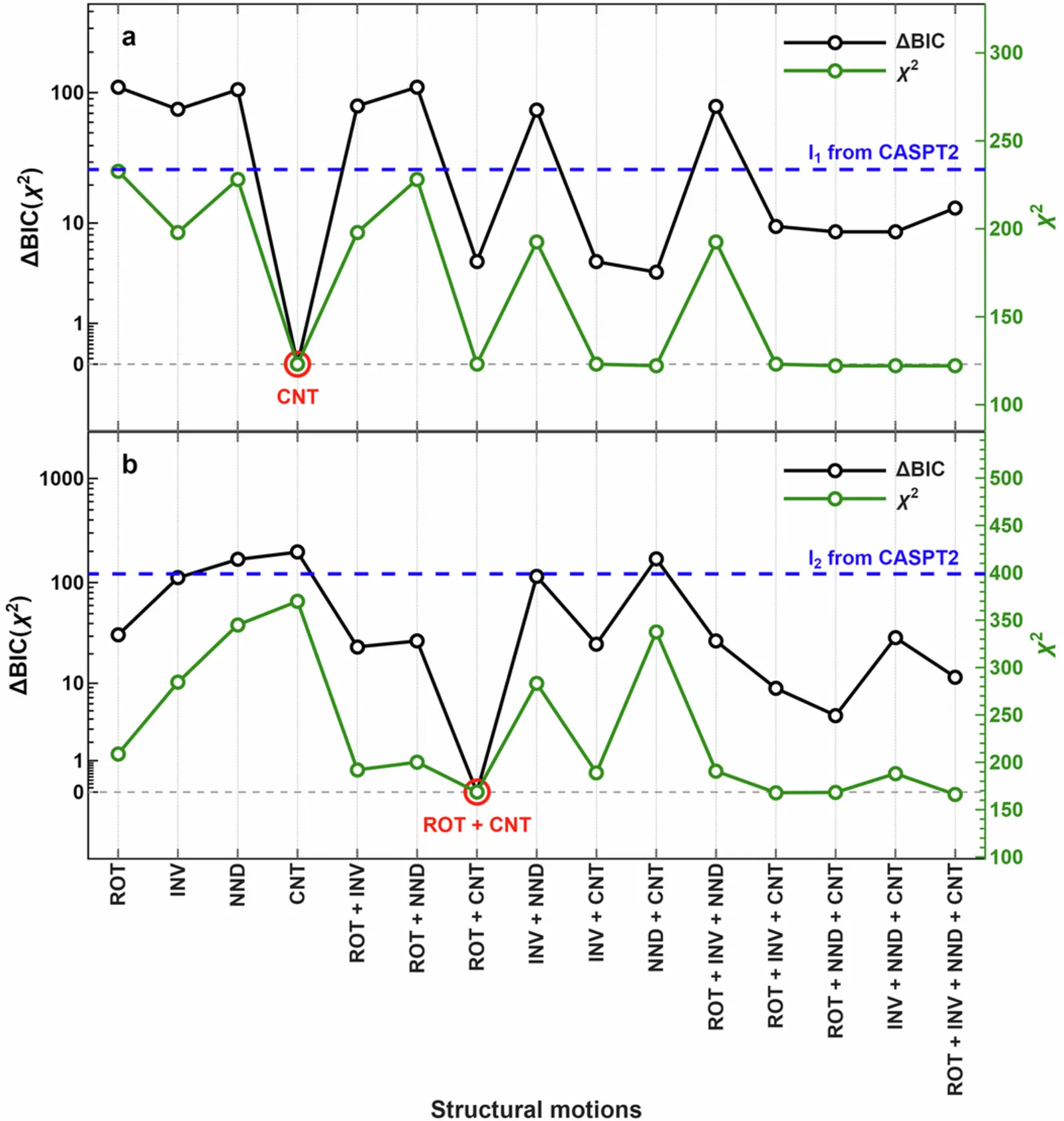
Selection of parsimonious structural motions using SSPM (Systematic Scan for Parsimonious Motions). SSPM identifies the simplest combination of structural motions consistent with the experimental data by evaluating all possible combinatorial subsets of the four basis motions and ranking them using the Bayesian Information Criterion (BIC). **a**, SSPM results for I_1_ starting from *trans*-AB. **b**, SSPM results for I_2_ starting from I_1_. In all panels, the right *y*-axis shows *χ*^2^ values (green) for visual reference, and the left *y*-axis shows ΔBIC(*χ*^2^) values (black), defined as the difference in BIC relative to the best (lowest-BIC) model. BIC serves as the sole criterion for model selection; ΔBIC = 0 corresponds to the optimal model, and larger values indicate progressively less favourable models. The *x*-axis denotes different combinations of structural motions. The four basis motions considered are N–N bond rotation (ROT), inversion (INV), N–N bond-length change (NND), and C–N bond torsion (CNT), evaluated individually and in all possible combinations (15 models in total). The blue dashed horizontal lines represent the *χ*^2^ values calculated from the CASPT2-calculated structures of I_1_ and I_2_. The BIC-optimal models are highlighted with red circles. I_1_ is most parsimoniously described by CNT alone, and I_2_ is most parsimoniously described by ROT + CNT.

To further validate and quantify the motions identified by SSPM, as well as to determine the governing motions for structures to which SSPM was not applied (CASPT2-calculated structures and the TRXL-refined *cis*-AB structure), we developed two complementary methods: basis-motion analysis (BMA), which evaluates how closely a target structure can be reproduced by applying specific single or combined motions to the starting structure (Supplementary Method 14), and motional contribution analysis (MoCA), which quantifies the individual contribution of each motion within a given assignment (Supplementary Method 15 and Supplementary Table 5). The analysis using SSPM, BIC, BMA and MoCA showed that *trans*-AB → I_1_ can be adequately described by a single motion of CNT (Extended Data Fig. 6a). The I_1_ → I_2_ conversion requires a combination of ROT (67%) and CNT (33%), indicating that ROT is the dominant motion (Extended Data Fig. 6b). The BMA and MoCA further reveal that the I_2_ → *cis*-AB conversion is characterized by ROT (56%) + INV (39%) + CNT (5%), indicating that the dominant motion is ROT, with a substantial contribution from INV. These reveal that the TRXL-refined isomerization pathway follows a CNT-first, INV-last sequence: CNT alone drives the *trans*-AB → I_1_ step, ROT becomes the dominant motion in I_1_ → I_2_, and INV becomes substantial only in I_2_ → *cis*-AB. Application of the same BMA, BIC and MoCA analyses to the CASPT2-calculated structures reveals the same set of elementary motions but in a fundamentally different temporal order, with INV contributing early and CNT late (Supplementary Tables 3–5 and Supplementary Notes 4 and 6), underscoring the importance of direct experimental structural determination.

Although the involvement of more complex structural coordinates, especially involving those not extensively explored here such as CCCN improper angles, CCN angles and C–N distances, cannot be ruled out, the determined motional assignment provides a sufficient and statistically robust description of the experimental observations, within the framework of the minimal set of structural motions necessary to describe the data. Furthermore, although the 400-nm excitation imparts excess vibrational energy, the small refined mean-square displacement (*σ*^2^) values for the two intermediates (*σ*^2^ = 0.017 ± 0.003 Å^2^ for I_1_ and * σ*^2^ = 0.005 ± 0.002 Å^2^ for I_2_; *σ*^2^ was fixed at 0 for *cis*-AB, the thermally relaxed product) provide strong evidence that their structural ensembles remain well defined. This suggests that the excess energy is effectively dissipated to the solvent rather than inducing large-amplitude randomized motions, thereby preserving the dominant reaction coordinate.

## Structures of I_1_ and I_2_

Refinement showed that the *cis*-AB structure required relatively minimal adjustment, remaining highly similar to the CASPT2-calculated structure (Extended Data Table 3), with a small *χ*_*ν*_^2^ reduction of 19%. In contrast, the refined I_1_ and I_2_ structures showed substantially improved agreement with the experimental data, yielding *χ*_*ν*_^2^ reductions of 47% and 58%, respectively, relative to the initial CASPT2-calculated structures (Fig. 3f). To compare structures, we examined six key parameters: (1) ∠CNNC, which measures the relative orientation of the two phenyl rings about the N–N bond and is primarily modified by ROT; (2) ∠CNN, which measures the bending of the C–N bond relative to the N–N bond at each nitrogen atom and is primarily modified by INV; (3) ∠CCNN, which measures the twist of each phenyl ring out of the CNN plane and is predominantly modified by CNT; (4) *r*_NN_, the N–N bond length, which is directly modified by NND; (5) *d*_TL_, defined as the shortest distance between the line connecting the two N atoms and the line connecting the two C atoms bonded to them, which quantifies the out-of-plane displacement of the azo group; and (6) *R*_g_, the radius of gyration. The changes in these six parameters along the *trans*-AB → I_1_ → I_2_ → *cis*-AB pathway are shown in Fig. 4a.

The TRXL-refined I_1_ structure deviates markedly from the planar configuration of *trans*-AB, exhibiting ∠CCNN angles (87.0°, −87.0°) whereas the ∠CNNC dihedral and ∠CNN angles remain unchanged from those of *trans*-AB. This nonplanarity can be understood to arise from the nπ* excitation, which weakens the *π*-bond character of the N=N bond, disrupts the delocalized *π*-conjugation across the molecule, and relaxes the planarity constraint of the ground-state structure. The TRXL-refined I_2_ structure retains similarly large ∠CCNN angles (−71.9°, −71.9°) and additionally shows a substantial ∠CNNC rotation to 115.5°, indicating that ROT becomes active at this stage while CNT persists. A detailed comparison of the TRXL-refined and CASPT2-calculated structures is provided in Supplementary Note 6.

Numerous time-resolved spectroscopic studies on the nπ* excitation of *trans*-AB have proposed inconsistent mechanisms^1,4,16,17,19,21,23,31–33^, including rotation^1,4^, inversion^16,17,19^, hula twist^21^ and inversion-assisted rotation^23^, while three studies left the mechanism unassigned^31–33^ (Extended Data Table 1 and Supplementary Note 8). Theoretical investigations show a similar lack of consensus (Extended Data Table 2 and Supplementary Note 7). Most support a rotation-driven mechanism^2,3,5–15,22,24–30^, either a rotation about the N–N bond (which some studies instead call torsion) or an inversion-assisted rotation, whereas a few proposed alternatives such as inversion^18^ or pedal-like motion^20^. The widely invoked inversion-assisted rotation mechanism, which couples ∠CNN and ∠CNNC changes, fails to reproduce the pronounced change in ∠CCNN revealed by the TRXL experiment. In this context, the experimentally identified pathway provides a crucial reference for future theoretical studies.

The persistent disagreement among previous experimental and theoretical studies probably stems from over-reliance on limited structural parameters. For example, mechanisms are often classified as rotation based solely on ∠CNNC changes^2,3,5–15^. Our structural analysis indicates that the initial motion does not involve ∠CNNC changes, and that even at later stages, these are accompanied by changes in other parameters, such as ∠CCNN, rather than representing pure ROT. Similarly, focusing only on the changes in ∠CNNC and ∠CNN shown in Fig. 4a might incorrectly support the inversion-assisted rotation mechanism, which has been proposed not only by computational studies but also by a recent transient-absorption study^23^. However, our findings demonstrate that the structural evolution up to I_2_ does not involve substantial changes in ∠CNN.

Experimental uncertainties arising from the finite signal-to-noise ratio may influence the extent of motions determining the numerical values of the refined structural parameters, such as bond angles and dihedral angles of I_1_ and I_2_. Although future higher-quality data might lead to quantitative adjustments, they are unlikely to alter the fundamental mechanistic assignment. Our primary conclusion regarding the dominant reaction coordinate, involving CNT and ROT, is robustly supported by our systematic evaluation of alternative structural models, although the involvement of more complex structural coordinates unexplored in this work cannot be ruled out.

## Overview of the isomerization

The structural trajectory based on the determined structures is visualized from three orthogonal views (front, top and side) in Fig. 4b and Supplementary Video 2, and from an oblique view in Supplementary Video 1. The unveiled pathway resolves a long-standing contradiction in azobenzene photoisomerization studies. The independence of the nπ*-excited *trans*-to-*cis* isomerization kinetics from solvent viscosity has been taken as evidence for a volume-conserving pathway that requires little rearrangement of the surrounding solvent^21^. However, numerous theoretical studies have proposed rotation about the N–N bond as the primary isomerization coordinate^2,3,5–15,22,24–30^, which would entail large phenyl-ring sweeps and correspondingly strong solvent friction, contradicting the observed viscosity independence.

The TRXL-refined intermediates shed light on this apparent contradiction. I_1_ and I_2_ are more compact than gas-phase CASPT2-calculated counterparts, as reflected in a smaller *R*_g_ (Fig. 4a, Extended Data Table 3 and Supplementary Note 9), consistent with isomerization within a solvent cage favouring geometries that displace less solvent. The compactness of individual intermediates alone, however, does not ensure a volume-conserving pathway: the solvent volume swept during transitions is critical. To quantify this, we compared two limiting scenarios for each transition: an N–N fixed frame where only the phenyl rings move, and a minimum-displacement frame where all atoms rearrange concertedly (Supplementary Note 11 and Supplementary Table 7). The minimum-displacement pathway sweeps only 20 Å^3^, 37 Å^3^ and 114 Å^3^ for *trans*-AB → I_1_, I_1_ → I_2_, and I_2_ → *cis*-AB transitions, respectively, compared with 104 Å^3^, 65 Å^3^ and 115 Å^3^ for the intuitive N–N fixed pathway, representing reductions of 81%, 44% and approximately 0.5%. The reduction is largest for *trans*-AB → I_1_, where the near-90° ∠CCNN change is achieved via bicycle-pedal-like concerted displacement of the azo bridge (Supplementary Videos 1 and 2) rather than phenyl-ring sweeps (Supplementary Videos 3 and 4). Even for I_1_ → I_2_, where ROT becomes active, the accompanying CNT and the optimally pre-configured ∠CCNN of I_1_ ensure that the phenyl rings do not sweep through large volumes of solvent. Although isotropic TRXL data provide interatomic distances but not absolute molecular orientations, the observed solvent-viscosity independence^21^ favours the volume-conserving scenario (Fig. 4b) over the ring-sweep alternative (Supplementary Fig. 8). The TRXL-derived pathway thus reconciles the observed solvent-viscosity independence with the substantial ∠CNNC changes required to reach the *cis*-AB product. The isomerization achieves a 170° cumulative ∠CNNC rotation from *trans*-AB to *cis*-AB not by large-amplitude motion of any single group, but by coordinated small displacements of all atoms, including active participation of the nitrogen atoms, minimizing solvent-cage disruption.

Using TRXL, we provide insight into the long-standing debate over the mechanism of the *trans*-to-*cis* isomerization of azobenzene. This study emphasizes the necessity of analysing global structural changes over isolated geometric parameters to fully grasp reaction pathways. The successful TRXL data collection and analysis of a molecule composed solely of carbon, nitrogen and hydrogen, without heavy atoms, establishes its applicability to light-atom organic systems, paving the way for revealing the structural dynamics and mechanisms of diverse organic reactions in solution.

## Methods

### Data collection

TRXL experiments were conducted at the XSS beamline of the Pohang Accelerator Laboratory X-ray free-electron laser (PAL-XFEL) facility. Key experimental parameters are summarized in Supplementary Table 1. A laser-pump and X-ray-probe scheme was used to initiate the photoisomerization reaction of azobenzene. Optical laser pulses at a wavelength of 400 nm, generated as the second harmonic of a femtosecond titanium:sapphire laser system, were used as the pump pulses. These pump pulses with an energy of 8.5 µJ were focused onto the sample with a spot size of 55 × 66 µm^2^ (full-width at half-maximum (FWHM), horizontal × vertical), corresponding to a laser fluence of 1.5 mJ mm^−2^. The repetition rate of the optical laser was 30 Hz. The X-ray beam, used as the probe, was focused onto the sample with a spot size of 20 µm × 20 µm (FWHM), delivering approximately 1 × 10^12^ photons per pulse at a repetition rate of 60 Hz, with a centre energy of 15.01 keV. By utilizing the X-ray with twice the repetition rate of the laser, we alternately measured laser-on and laser-off scattering images. The optical laser and X-ray beams were overlapped at a 10° crossing angle at the sample position. The temporal resolution of the experiment, defined by the instrument response function (IRF), was determined by the temporal widths of optical laser and X-ray pulses, the velocity mismatch due to the capillary jet width and timing jitter. On the basis of our data analysis, the IRF value was found to be 169 ± 5 fs (FWHM; Supplementary Method 1).

The sample solution, 50-mM *trans*-AB (Sigma-Aldrich, 98%) dissolved in methanol, was flowed through a 100-µm capillary using a gear pump to create an open jet for the pump–probe measurement. The jet speed was approximately 3 m s^−1^, sufficient to prevent multiple exposures of the sample to the pump laser pulses. Unlike typical TRXL experiments where the sample solution is recirculated, the sample solution was flowed only once and discarded after a single exposure to the laser and X-ray pulses, as the isomerization reaction to *cis*-AB is irreversible. In many solvents, *cis*-AB exhibits two- to three-times higher absorption than *trans*-AB at 400 nm (ref. ^21^). Thus, the generation of *cis*-AB under ambient light could potentially interfere with obtaining an unaltered signal from *trans*-AB. To address this, the preparation of *trans*-AB was performed in a darkroom, and the TRXL experiments were also conducted in the absence of ambient light. Furthermore, the absorption spectrum of *trans*-AB under ambient light showed no detectable changes within 5 h. For the *trans*-AB TRXL experiments, we used *trans*-AB solutions prepared within 5 h.

To obtain reference data representing the response of bulk solvent, a separate fs-TRXL measurement was conducted with neat methanol at a pump fluence of 5.3 mJ mm^−2^. The neat methanol was heated via two-photon absorption of the 400-nm pump pulse, depositing energy equivalent to single-photon excitation at 200 nm. All chemicals, including the solvent, were sourced from commercial suppliers and used as received without further purification.

The X-ray scattering patterns were measured using a Rayonix MX225-HS detector operated in 6 × 6 binning mode, resulting in an effective pixel size of 234 µm and a frame size of 960 × 960 pixels. The sample-to-detector distance was set to 40.4 mm, providing a *q* range of 1.0 Å^−1^ to 7.0 Å^−1^. For the *trans*-AB solution, TRXL data were collected at 140 nominal time delays from −0.5 ps to 100 ps (uniform 0.05 ps steps to 2.0 ps, followed by logarithmic spacing of approximately 50 points per decade), with approximately 250 laser-on and 250 laser-off single-pulse images per delay per run. To achieve a sufficient signal-to-noise ratio (SNR) for data analysis, we acquired 18 runs for the target sample, *trans*-AB, and 4 runs for the neat solvent. In total, around 4,500 laser-on scattering images per time delay (with an equal number of laser-off images) were accumulated, resulting in fs-TRXL data with satisfactory SNRs.

The molar absorptivity of *trans*-AB at 400 nm was measured to be 292 M^−1^ cm^−1^ under the experimental conditions. To confirm that the photoisomerization is initiated by a single-photon process at our experimental fluence (1.5 mJ mm^−2^), we examined the pump-fluence dependence of the signal using femtosecond transient absorption experiments in which the pump fluence was varied up to 1.7 mJ mm^−2^. The signal amplitude at 2 ps exhibited a linear relationship with pump fluence (not shown). This linearity confirms that multi-photon excitation or ionization processes are negligible under our experimental conditions.

### Data processing

Supplementary Figs. 1 and 2 detail the entire data-processing workflow, from raw images to the final structural refinement, explicitly showing each separation and correction step. The spatial overlap between the pump and probe pulses was established by adjusting the optical laser pointing to maximize the transient signal amplitude. The temporal overlap between the two pulses was ensured before data collection using an yttrium aluminium garnet (YAG) crystal, by monitoring the X-ray-induced change in its optical transmittance to identify the coincidence of the two pulses. No dedicated timing tool was used in this experiment. This YAG measurement was used to determine only the nominal time zero of the recorded delays; the precise time zero was refined afterwards, during data processing, in the two stages described later. Before all subsequent processing steps, each recorded diffraction image was corrected by subtracting a dark image (background) acquired under X-ray beam-off conditions, followed by corrections for polarization and solid angle effects. The polarization correction accounts for the linearly polarized nature of XFEL X-rays, which causes azimuthally anisotropic scattering intensity not related to sample anisotropy. Specifically, the measured intensity (*I*_measured_) was corrected for polarization as *I*_polar_ = *I*_measured_ × 1/[1 − sin^2^(2*θ*)cos^2^(*φ*)], where *φ* is the azimuthal angle relative to the X-ray polarization direction and 2*θ* is the scattering angle. The solid angle correction compensates for the reduced solid angle subtended by detector pixels at higher scattering angles in a flat detector geometry. This compensation requires an intensity correction with the formula: *I*_corrected_ = *I*_polar_ × 1/cos^3^(2*θ*). These corrections were applied using standard formulas implemented in the pyFAI package^43^.

Each corrected two-dimensional scattering image was then processed to obtain one-dimensional scattering curves as a function of *q*, the magnitude of the momentum transfer vector (*q* = 4*π*sin*θ*/*λ*, where *λ* is the X-ray wavelength). For each single-shot image, we obtained either (1) azimuthal scattering curves, *S*_azi_(*q*, *t*)^single^, through azimuthal averaging using the ‘integrate1d’ function from pyFAI, or (2) separated isotropic and anisotropic scattering curves, *S*_iso_(*q*, *t*)^single^ and *S*_aniso_(*q*, *t*)^single^, via the anisotropic decomposition method^36,40,44–47^ using the ‘integrate2d’ function from pyFAI. The isotropic component contains information on changes in interatomic distances independent of molecular orientation, whereas the anisotropic component reflects orientational dynamics of the molecules. The detailed procedure for the anisotropic decomposition method is provided in the literature^36,40,44–47^. This two-dimensional-to-one-dimensional conversion was applied identically to every detector image, regardless of whether it was recorded laser-on or laser-off; each image yields one isotropic and one anisotropic curve, and the laser-on/off distinction enters only at the subsequent difference and pairing step (below).

Before calculating difference scattering curves, each scattering curve was normalized by its area under the curve (AUC). Specifically, *S*_azi_(*q*, *t*)^single^ and *S*_iso_(*q*, *t*)^single^ were normalized by dividing by the AUC within the normalization range of *q* = 4–6 Å^−1^, while *S*_aniso_(*q*, *t*)^single^ was normalized by the AUC of the corresponding *S*_iso_(*q*, *t*). For each laser-on (pumped) measurement, we then calculated difference scattering curves by subtracting an appropriately paired laser-off (unpumped) signal. Rather than using simple time-sequential pairing, we used an AUC-based pairing method to minimize systematic errors. Although normalization is applied to account for shot-to-shot fluctuations in beam intensity and sample thickness, detector nonlinearity (that is, imperfect linearity between incident photon count and recorded signal intensity) can introduce systematic artefacts when subtracting laser-on and laser-off images with substantially different signal amplitudes. To suppress these artefacts, each laser-on curve was subtracted by its matched laser-off curve, the one with the most similar AUC value, thereby minimizing the influence of detector response nonlinearity on the resulting difference signal. This procedure yielded single-shot difference scattering signals, Δ*S*_[x]_(*q*, *t*)^single^ ([x] = azi, iso or aniso), for each time delay *t*.

To improve the SNR, multiple single-shot difference curves measured at the same time delay were averaged in two stages: (1) within each run and (2) across runs. Before averaging, an outlier rejection procedure was applied to ensure data quality. For each single-shot measurement, we calculated the AUC of the absolute difference signal (∫|Δ*S*(*q*, *t*)^single^|d*q*) over the *q* range of 1.0–7.0 Å^−1^. For the azimuthal data, the AUC was computed from Δ*S*_azi_(*q*, *t*)^single^. For the isotropic and anisotropic data, where each shot yields a paired isotropic and anisotropic curve, the AUC was computed from the isotropic difference, Δ*S*_iso_(*q*, *t*)^single^; shots identified as outliers were then excluded from both the isotropic and their paired anisotropic averages, with the anisotropic curves not filtered separately. Single-shot curves with AUC values exceeding an empirically determined threshold were identified as outliers and excluded from subsequent averaging. This procedure typically retained 95–99% of the collected data, removing only extreme outliers arising from beam instability or sample flow irregularities. The outlier-filtered difference curves were first averaged within each run to obtain the run-averaged Δ*S*_[x]_(*q*, *t*)^run^ and its associated standard error of the mean, *σ*_[x]_(*q*, *t*)^run^. The standard error of the mean was calculated as:

1 $${\sigma }_{[{\rm{x}}]}{(q,t)}^{\mathrm{run}}={\sigma }_{[{\rm{x}}]}{(q,t)}^{\mathrm{single}}/\sqrt{{N}_{\mathrm{pair}}}$$

Here *σ*_[x]_(*q*, *t*)^single^ is the standard deviation of the single-shot difference curves within that run for the specific time delay and *q* value, and *N*_pair_ is the number of difference scattering curves within that run that survived the outlier rejection.

The run-averaged curves from the 18 runs of the *trans*-AB TRXL data, all acquired on the same set of 140 nominal time delays (−0.5 ps to 100 ps), were then averaged across runs into a single dataset (across-run-averaged). First, the nominal time delays were referenced to the experimental time zero by subtracting a single, run-independent preliminary offset, *t*_0,pre_, which was determined from the across-run-averaged anisotropic signal before time-pooling, giving the preliminary pump–probe delays. The 18 runs differed slightly in the overall amplitude of their difference signals, reflecting small run-to-run variations in the experimental conditions, such as the sample (liquid jet) thickness and the position and intensity of the X-ray and laser pulses. To place them on a common amplitude scale, each run was first characterized by a single amplitude: the integrated magnitude of its isotropic difference signal, ∫|*q*Δ*S*_iso_|d*q* over *q* = 1–7 Å^−1^ averaged over the 90–100 ps time delays. The run with the largest amplitude was taken as the reference. Each run-averaged curve was then multiplied by a scaling factor equal to the reference amplitude divided by that run’s own amplitude, so that run-to-run amplitude fluctuations were removed. The corresponding uncertainty, *σ*_[x]_(*q*, *t*)^run^, was scaled by the same factor. The *t*_0,pre_-corrected, scaled curves were then binned onto a common analysis grid of 137 time delays (spanning −0.35 ps to 100 ps: uniform 0.05 ps spacing up to 2.0 ps, then approximately logarithmic spacing of approximately 50 points per decade). Specifically, each run’s *t*_0,pre_-corrected delay was assigned to the nearest delay of the analysis time grid, and the contributions from all runs at each grid delay were then averaged with weights proportional to the *N*_pair_ of the run-averaged curve (an image-count-weighted average). This across-run averaging onto the analysis time grid is distinct from the pooling along the time axis applied later (below), and gave the across-run-averaged difference scattering curves Δ*S*_iso_(*q*, *t*)^raw^, Δ*S*_aniso_(*q*, *t*)^raw^ and Δ*S*_azi_(*q*, *t*)^raw^ (Extended Data Fig. 1a,c,e); their uncertainties *σ*_[x]_(*q*, *t*)^raw^ were propagated from the *σ*_[x]_(*q*, *t*)^run^ according to standard error propagation rules.

For quantitative analysis, these across-run-averaged curves were placed on an absolute scale, normalized to the scattering intensity of a single solvent molecule, following standard methods in the TRXL community^36,38,40,45,46,48–61^. The total scattering curve of the solvent was calculated from the pair distribution functions *g*(*r*) obtained from molecular dynamics simulations, giving the scattering intensity in electron units (e.u.), and normalized by the number of solvent molecules in the molecular dynamics simulation box to yield the intensity in e.u. per solvent molecule. A scaling factor was determined by comparing the normalized experimental total scattering curve with this simulated curve. This use of the simulated scattering curve of the pure solvent to calibrate the experimental solution data is justified because, at the low solute concentration (50 mM), the experimental total scattering intensity of the solution arises almost exclusively from the bulk solvent. This single scaling factor was applied identically to all three experimental difference scattering curves, Δ*S*_iso_(*q*, *t*)^raw^, Δ*S*_aniso_(*q*, *t*)^raw^ and Δ*S*_azi_(*q*, *t*)^raw^, and to their uncertainties *σ*_[x]_(*q*, *t*)^raw^, expressing the signals in e.u. per solvent molecule. This normalization process enables the extraction of quantitative information regarding the reaction dynamics, including the concentration of solute molecules participating in the isomerization pathways.

The PEPC step then removes the solvent contribution from the signal, propagating the raw-data uncertainty *σ*_[x]_(*q*, *t*)^raw^ into the solvent-independent data, Δ*S*_[x]_^⊥^(*q*, *t*_*k*_), whose per-point standard error is *σ*_[x]_^⊥^(*q*, *t*_*k*_). For the subsequent quantitative analysis, these solvent-independent data at adjacent time delays were pooled along the time axis to further improve the SNR. Rather than an unweighted two-point moving average, the difference scattering curves at each pair of neighbouring time delays (*t*_*k*_ and *t*_*k*+1_) were averaged with weights proportional to the number of images contributing to each delay (an image-count-weighted mean of the signals). Each pooled time delay was assigned the corresponding image-count-weighted mean of the two time delays. This time-pooling reduced the 137 original time delays on the analysis grid (spanning from −0.35 ps to 100 ps) to 136 pooled time delays spanning about −0.33 ps to 98 ps. The same image-count time-pooling was also applied to the pre-PEPC anisotropic data, Δ*S*_aniso_(*q*, *t*)^raw^, which was then used to determine the refined time zero and the IRF, as described below. The isotropic curves in Figs. 2 and 3 are the time-pooled dataset, whereas the curves in Extended Data Figs. 1 and 2 are shown before time-pooling. Throughout this paper, difference curves are presented as *q*Δ*S*(*q*, *t*) for visualization in the figures, to emphasize high-*q* features associated with solute structural changes. This *q* weighting was applied only for display and not used in quantitative analysis.

The experimental time zero was accounted for in two stages. The preliminary offset above (*t*_0__,__pre_) served only to reference the nominal delays to time zero before merging. The refined time zero (*t*_0__,__ref_) and the IRF were then determined, after merging and time-pooling, by fitting the time-pooled, pre-PEPC anisotropic signal. Near time zero, the solvent produces a coherent response, the optical Kerr effect, a sharp feature that marks the arrival of the pump pulse and therefore provides an accurate temporal reference for the time zero and the IRF. Because PEPC removes the solvent contribution, it would also remove this feature. The fit therefore uses the anisotropic data before PEPC, where the feature is preserved. The refined time zero is consistent with *t*_0__,__pre_ to within about 15 fs (well below the IRF). These *t*_0__,__ref_ and IRF were then held fixed as parameters in the kinetic analysis of the time-pooled, PEPC-treated isotropic (Fig. 3) and anisotropic (Extended Data Fig. 3) signals.

From these isotropic, time-pooled, solvent-independent data, Δ*S*_iso_^⊥^(*q*, *t*_*k*_), the kinetic analysis involving SVD and kinetics-constrained analysis was performed first, followed by structural refinement. Fitting the RSVs obtained from SVD yielded time constants used to set up the kinetic model. The time-dependent concentration profiles of the species were calculated from the kinetic model and then used by the kinetics-constrained analysis to extract the SADSs for the structural refinement.

The standard error of the RSVs enters the analysis at two stages: (1) the fit of the RSVs and (2) the subsequent estimation of the uncertainties of the resulting time constants. This standard error is denoted *σ*_*n*_(*t*_*k*_) for the *n*th RSV at time delay *t*_*k*_. The two stages depend on *σ*_*n*_(*t*_*k*_) in different ways. The fit is sensitive only to the relative magnitudes of *σ*_*n*_(*t*_*k*_) across the time points, as these relative magnitudes determine the relative weights of the data points and hence the location of the *χ*^2^ minimum, that is, the best-fit time constants. A uniform rescaling of *σ*_*n*_(*t*_*k*_) multiplies the entire *χ*^2^ by a constant and therefore leaves the location of its minimum, and hence the fitted time constants, unchanged. The uncertainties of the time constants, by contrast, are obtained by displacing each time constant from its best-fit value until the *χ*^2^ increases by a fixed amount, and the required displacement is taken as the uncertainty. Such a displacement worsens the agreement between the RSVs and the model, so that the residuals, and with them the *χ*^2^, increase. Because the *χ*^2^ weights the residuals by 1/*σ*_*n*_(*t*_*k*_)^2^, the rate of this increase is governed by the absolute magnitude of *σ*_*n*_(*t*_*k*_): a smaller *σ*_*n*_(*t*_*k*_) produces a faster increase, and hence a smaller displacement to the threshold. The resulting uncertainty therefore depends on the absolute magnitude of *σ*_*n*_(*t*_*k*_), and not merely on its relative magnitudes. We therefore used two estimates of *σ*_*n*_(*t*_*k*_): an analytic estimate *σ*_*n*_^ana^(*t*_*k*_) for weighting the fit, and a bootstrap estimate *σ*_*n*_^boot^(*t*_*k*_) for estimating the uncertainties.

The model fitted to each RSV is a sum of exponential functions convolved with the IRF, and each RSV value is weighted by 1/[*σ*_*n*_^ana^(*t*_*k*_)]^2^. The analytic estimate *σ*_*n*_^ana^(*t*_*k*_) was obtained by propagating the per-point error of the data, *σ*_[x]_^⊥^(*q*, *t*_*k*_), through the time-pooling and the SVD. It reproduces the correct relative magnitudes of *σ*_*n*_(*t*_*k*_), and therefore yields the correct time constants, but it underestimates the absolute magnitude of *σ*_*n*_(*t*_*k*_), because it does not fully account for the correlation introduced between neighbouring time points by the two-point time-pooling (Supplementary Method 1).

The bootstrap estimate *σ*_*n*_^boot^(*t*_*k*_), by contrast, does capture this correlation. It was obtained by generating repeated synthetic datasets, each formed by adding random noise to the data at the level of the per-point error *σ*_[x]_^⊥^(*q*, *t*_*k*_), and re-running the time-pooling and the SVD on each (Supplementary Method 1). Using *σ*_*n*_^boot^(*t*_*k*_) as the standard error, the uncertainties of the time constants were then obtained by the profile-likelihood analysis, with each time constant displaced until the *χ*^2^ increased by Δ*χ*^2^ = 1 and the remaining parameters re-optimized at each displacement (Supplementary Methods 1 and 4). The standard error of the SADSs, denoted *σ*_*n*_(*q*_*j*_) for the *n*th SADS at the *j*th *q* point *q*_*j*_, was obtained by an analogous bootstrap (Supplementary Methods 2 and 3). In the structural refinement, the structural parameters were determined by minimizing a *χ*^2^ in which the difference in the SADSs at each *q*_*j*_ was divided by its standard error *σ*_*n*_(*q*_*j*_) and squared. Their 1*σ* uncertainties were obtained by the same profile-likelihood criterion (Δ*χ*^2^ = 1). The *σ*_*n*_(*q*_*j*_) are shown as the error bars on the SADSs (Fig. 3f), and the profile-likelihood limits as the error bars on the refined structural parameters (Fig. 4a).

### Obtaining solvent-independent TRXL data

TRXL signals generally consist of three main components: (1) the signal arising from changes in the solute itself (solute term, Δ*S*_solute_(*q*)), (2) the signal resulting from changes in the solvent–solute interatomic distances (cage term, Δ*S*_cage_(*q*)), and (3) the signal from the response of the bulk solvent (solvent term, Δ*S*_solvent_(*q*)), which includes contributions from changes in the temperature and density of the bulk solvent induced by heat dissipation from the photoexcited solute molecules, as well as the rearrangement of solvent molecules due to the optical Kerr effect^46^. The sum of the solute and cage terms is referred to as solute-related terms (Δ*S*_solute-related_(*q*) = Δ*S*_solute_(*q*) + Δ*S*_cage_(*q*)), and the kinetics of Δ*S*_solute-related_(*q*) reflect the behaviour of the solute molecules. In contrast, Δ*S*_solvent_(*q*) may exhibit different kinetics associated with the hydrodynamic response of bulk solvent and optical Kerr effect of the bulk solvent^46^. To accurately analyse the kinetics of the solute molecules, it is important to eliminate the influence of the solvent term from the TRXL signals.

As shown in Fig. 2, more than 90% of the TRXL signal of azobenzene is attributed to the solvent signal. This large contribution from the solvent makes it crucial to accurately account for and subtract this component to extract the solute-related kinetics. To address this, the PEPC was used^41^. The core concept of this approach is to project the data into a transformed space where the contribution of Δ*S*_solvent_(*q*) becomes zero, thereby effectively screening out the influence of Δ*S*_solvent_(*q*). We denote the data projected into this space using the superscript ⊥ symbol. In this transformed space, the solvent contribution is mathematically expressed as Δ*S*^⊥^_solvent_(*q*) = 0. As a result, the PEPC-treated data simplifies to Δ*S*^⊥^(*q*) = Δ*S*^⊥^_solute_(*q*) + Δ*S*^⊥^_cage_(*q*) + Δ*S*^⊥^_solvent_(*q*) = Δ*S*^⊥^_solute_(*q*) + Δ*S*^⊥^_cage_(*q*) = Δ*S*^⊥^_solute-related_(*q*), leaving only the solute and cage contributions. By projecting the data into a space orthogonal to the solvent terms, this transformation isolates the solute-related terms, enabling the accurate extraction of solute-specific kinetics and structural information, as detailed in previous work^41,60,62^. For the PEPC treatment, we used heating signals and artefact components obtained from a separate experiment with a neat solvent as known reference components, allowing for the removal of their contributions from the data. By analysing the resulting PEPC-treated data, we were able to isolate the kinetics of the solute molecules. The solvent contributions were effectively screened out from the original TRXL data (Extended Data Fig. 1) to obtain the PEPC-treated data presented in Extended Data Fig. 2, which we refer to as the solvent-independent data. The term ‘solvent-independent data’ is equivalent to the term ‘solvent-contribution-free data’ used in a previous study^62^; both describe TRXL data that has been projected into a subspace orthogonal to the solvent response. To obtain the reference components representing the solvent response for this PEPC procedure, we performed a separate fs-TRXL measurement of neat methanol under two-photon heating conditions. The LSVs extracted directly from the SVD of this neat solvent data were used as the reference components without further modification. These components sufficiently capture the signal variations corresponding to the solvent response in the azobenzene solution, enabling effective removal of solvent contributions through orthogonal projection. Both Δ*S*_iso_(*q*, *t*)^raw^ and Δ*S*_aniso_(*q*, *t*)^raw^ from the neat solvent dataset were independently subjected to SVD. The reference components were selected by individually inspecting each LSV and RSV pair against two criteria: (1) autocorrelation coefficient of the RSVs ≥ 0.5, ensuring temporal consistency, and (2) singular values well above the noise plateau, ensuring the components represent genuine signal variations rather than noise. Through this evaluation, 7 LSVs (LSVs 1–6 and 9) were selected for Δ*S*_iso_(*q*, *t*)^raw^, and 4 LSVs (LSVs 1–4) were selected for Δ*S*_aniso_(*q*, *t*)^raw^ (Supplementary Figs. 4 and 5). LSV_4_ for Δ*S*_iso_(*q*, *t*)^raw^ was included despite a lower overall autocorrelation because it exhibited a dominant contribution near time zero, capturing the initial ultrafast response. By separately projecting the Δ*S*_iso_(*q*, *t*)^raw^ and Δ*S*_aniso_(*q*, *t*)^raw^ of the *trans*-AB solution data onto the subspaces orthogonal to their respective reference components, PEPC effectively isolates the signal components strictly independent of the solvent response, thereby retaining only the signal components associated with solute structural changes that are orthogonal to the solvent response. The resulting solvent-independent data (Δ*S*^⊥^(*q*, *t*)) are presented in Extended Data Fig. 2. During this PEPC procedure, the standard errors of the PEPC-treated data, *σ*^⟂^(*q*, *t*), were calculated by propagating the standard error of the mean of the original TRXL data (*σ*(*q*, *t*)^raw^) and the uncertainties associated with the reference solvent components. Specifically, as PEPC is a linear projection operation, the covariance matrix of the projected data was computed using standard error propagation rules to derive *σ*^⟂^(*q*, *t*), ensuring that the uncertainties in both the raw experimental data and the solvent basis set were rigorously accounted for. We note that the oscillations observed in the low-*q* region of some SADS curves are a mathematical consequence of the PEPC procedure, which projects the data into a space orthogonal to the solvent response, and therefore should not be interpreted on their own as direct structural fingerprints of the solute. A flow chart illustrating the data analysis pipeline is shown in Supplementary Fig. 2.

### Structure analysis

We refined the structures of I_1_, I_2_ and *cis*-AB by minimizing *χ*_*ν*_^2^, which quantifies the discrepancy between the experimental and simulated SADSs. As described in Supplementary Method 5, simulated SADSs, SADS_*n*_′(*q*)s, were calculated based on the structures of four species: *trans*-AB, I_1_, I_2_ and *cis*-AB. For *trans*-AB, the structure derived from quantum chemical calculations (CASPT2) was used without further refinement. For I_1_ and I_2_, structural refinement was performed using a motion-based approach, considering four key structural motions as degrees of freedom: N–N rotation (ROT), inversion (INV), N–N bond-length change (NND) and C–N torsion (CNT). ROT describes the out-of-plane rotation of one phenyl ring about the N–N bond axis. This motion is quantified by the CNNC dihedral angle (∠CNNC), which defines the twisting of the molecule around the azo linkage. To model this, one phenyl group was rotated by a specified angle relative to the N–N axis, thereby introducing a controllable change in torsional strain. In INV, the CNN angles of both phenyl rings (∠CNN_1_ and ∠CNN_2_) are allowed to vary independently, with each ∠CNN treated as a separate fitting parameter during refinement. The concerted inversion of azobenzene, which has been extensively proposed in theoretical studies (Extended Data Table 2), can be described as a special case of INV, in which both ∠CNNs increase simultaneously and to the same extent, accompanied by a widening separation of the two rings. The NND motion is quantified simply by the N–N bond length (*r*_NN_). The CNT motion describes changes in the ∠CCNN dihedral angles, parameterized by two ∠CCNN angles. This internal coordinate change can be realized either by torsion of the phenyl rings about their respective C–N bond axes or by concerted displacement of the azo group (bicycle-pedal motion); both scenarios produce identical ∠CCNN changes and are therefore indistinguishable in the internal coordinate description. Each motion acts exclusively on a distinct structural degree of freedom (∠CNNC, ∠CNN, *r*_NN_ and ∠CCNN, respectively) and preserves the geometric reference frames of the others. Although CNT has received relatively little attention in previous studies of azobenzene isomerization, it was included to capture ∠CCNN changes that may accompany the structural rearrangement. Detailed definitions of these motions are provided in Supplementary Method 14. The *cis*-AB structure was refined separately by a direct two-parameter adjustment starting from the CASPT2 reference (see below).

For I_1_ and I_2_, we used SSPM, in which all possible combinations of the four motions are systematically evaluated and ranked by the BIC to identify the most parsimonious model consistent with the data (Supplementary Method 12). The intermediate structures I_1_ and I_2_ were constructed by applying the four elementary structural motions (ROT, INV, NND and CNT) defined above to their respective precursors (CASPT2-calculated *trans*-AB for I_1_, and the TRXL-refined I_1_ for I_2_). The structures of the intermediates generated by applying these four atomic motions were used to calculate the solute-only and cage scattering signals. The structural parameters corresponding to these four motions were optimized through *χ*_*ν*_^2^ minimization to ensure the best agreement between the simulated SADS′(*q*) and experimental SADS(*q*). The I_1_ structure was generated by applying motions to the CASPT2-calculated *trans*-AB geometry, whereas the I_2_ structure was generated by applying motions to the TRXL-refined I_1_ geometry. For *cis*-AB, rather than performing an SSPM scan, a direct two-parameter refinement (∠CNNC and ∠CCNN) was carried out starting from the CASPT2-calculated *cis*-AB geometry with Tikhonov regularization towards the CASPT2 reference (Supplementary Method 12.7). In all cases, the structural parameters and scaling coefficients were optimized simultaneously through nonlinear least-squares minimization (Supplementary Methods 3 and 12).

The structural refinement was carried out iteratively to achieve self-consistency among the solute atomic charges, the molecular dynamics simulations and the TRXL-refined geometry. In the first iteration, natural population analysis (NPA) atomic charges were computed for the CASPT2-calculated structures and used to parameterize GROMACS molecular dynamics simulations, from which the cage terms were obtained and applied in the structural refinement. The NPA charges were computed at different levels of theory according to the electronic state of the species: B3LYP-D3BJ/def2-TZVP for the ground-state species (*trans*-AB and *cis*-AB), and state-specific CASSCF(10,8)/aug-cc-pVDZ corrected by strongly contracted NEVPT2 (SC-NEVPT2) for the S_1_ intermediates (I_1_ and I_2_); in all cases, solvation was described using an implicit solvent model, the solvation model based on density. In each subsequent iteration, the NPA atomic charges were recomputed from the geometry refined in the preceding iteration, updated cage terms were generated and the structure was refined again. This cycle was repeated until the structural parameters showed negligible change between consecutive iterations, confirming convergence. Full details are provided in Supplementary Methods 8 and 9.

SADS_*n*_′(*q*)s calculated from the refined structures of I_1_, I_2_ and *cis*-AB demonstrated excellent agreement with the corresponding experimental SADS_*n*_(*q*)s (Fig. 3f). For *cis*-AB, the refinement confirmed that the solution-phase structure of *cis*-AB deviates only mildly from the CASPT2-calculated structure, showing a relatively small improvement in *χ*_*ν*_^2^ from 1.20 (CASPT2-calculated) to 0.97 (TRXL-refined), corresponding to a 19% reduction. This indicates both the sufficient accuracy of the CASPT2 structures of *trans*-AB and *cis*-AB, and the high quality of the TRXL data. In contrast, the refinement substantially improved the fits for I_1_ and I_2_, reducing the *χ*_*ν*_^2^ values from 0.46 to 0.24 for SADS_1_ (47% reduction) and from 0.79 to 0.33 for SADS_2_ (58% reduction). This indicates that the CASPT2-calculated structures of I_1_ and I_2_ deviate from their TRXL-refined counterparts to a greater extent than in the case of *cis*-AB. The structures of I_1_, I_2_ and *cis*-AB obtained from (1) CASPT2 calculations and (2) structural refinement using fs-TRXL data are compared in Extended Data Table 3, and a detailed comparison of these intermediate structures is provided in Supplementary Note 6.

It is worth noting that some of the *χ*_*ν*_^2^ values are near or well below unity. This is a well-known characteristic of X-ray scattering data analysis, arising from the fact that the effective number of independent information channels is fundamentally smaller than the number of measured *q* points. According to the Shannon sampling theorem, the independent information content of a scattering curve is governed by the Shannon number, *N*_s_ ≈ (*q*_max_ − *q*_min_) × *D*_max_/*π*, where *q*_max_ and *q*_min_ are the upper and lower limits of the measured *q* range, respectively, and *D*_max_ is the maximum interatomic distance contributing to the scattering signal. For a solute molecule such as azobenzene (*D*_max_ ≈ 12–15 Å), *N*_s_ is estimated to be approximately 25 for the *q* range of 1–7 Å^−1^ used in this study. However, in solution-phase scattering experiments, the signal also includes bulk solvent response and cage contributions, which extend to distances much larger than the solute dimensions (>30–60 Å). This effectively increases the independent information content to *N*_s_ ≈ 60–120. Because the distance range over which these solvent contributions affect the scattering signal is not precisely defined, the effective *D*_max_, and thus the true Shannon number, cannot be uniquely determined for solution scattering data. As adjacent *q* points carry redundant structural information beyond the Shannon limit, defining the degrees of freedom from the number of *q* points (*n*_*q*_ − *d*_f_, with the small number of fit parameters *d*_f_ « *n*_*q*_; Supplementary Method 3), rather than from the far smaller number of independent information channels (*N*_eff_ ≈ 110), results in systematically small *χ*_*ν*_^2^ values. Therefore, the observed *χ*_*ν*_^2^ values below unity do not indicate overfitting but rather reflect this inherent property of solution scattering data. A rigorous determination of *N*_eff_ using a BIC-based polynomial analysis is described in Supplementary Method 13. Moreover, one important caveat is that directly comparing the absolute *χ*_*ν*_^2^ values obtained for different SADSs (for example, comparing the *χ*_*ν*_^2^ of SADS_1_ with that of SADS_3_) can be misleading. This stems from the fact that the *χ*_*ν*_^2^ statistic is normalized by the uncertainties (*σ*_*n*_(*q*_*j*_)) of the extracted SADS, and in linear least-squares analysis, including the fitting of RSVs from SVD, the uncertainty of a signal component *S*_*k*_ is approximately inversely proportional to the norm of its concentration profile *C*_*k*_(*t*):

2 $${\sigma }_{{S}_{k}}\propto 1/\sqrt{\sum _{t}{C}_{k}{(t)}^{2}}$$

This relationship implies that a species with a higher concentration and longer duration (that is, a larger Σ*C*^2^) will therefore have a much smaller uncertainty ${\sigma }_{{S}_{k}}$ and correspondingly amplified *χ*_*ν*_^2^ values. In our dataset, the *cis*-AB species persists up to the latest measured time delay without decay, giving it the largest integrated concentration profile among all species. As a result, the *σ* associated with SADS_3_ (*cis*-AB) is systematically much smaller than those for the transient intermediates SADS_1_ and SADS_2_. Consequently, *χ*_*ν*_^2^ values for SADS_3_ (or fits dominated by it) are mathematically amplified and tend to appear larger than those of SADS_1_ and SADS_2_, even for strictly comparable fit qualities. Therefore, comparisons of *χ*_*ν*_^2^ values among candidate structural models for the same species should be the primary criterion for model assessment, rather than cross-species absolute comparisons.

### Systematic scan for parsimonious motions

To identify the minimal set of structural motions sufficient to describe each intermediate, we performed an SSPM. This procedure systematically evaluates all 15 possible combinatorial subsets of the four basis motions (4 single, 6 pairs, 4 triples, 1 quadruple) by fitting the simulated SADS from each candidate structure against the experimental SADS. Details are given in Supplementary Method 12. Model selection was performed using a *χ*^2^-based BIC, defined as BIC(*χ*^2^) = *χ*^2^ + (*k* + 4) × ln(*N*_eff_), where *k* is the number of structural motion parameters and the four additional parameters account for the mean-square displacement parameter *σ*^2^, which accounts for thermal broadening of interatomic distances, the scaling coefficient, and the two baseline coefficients *a*_*n*_ and *b*_*n*_, and *N*_eff_ is the effective number of independent data points determined by polynomial-order optimization (Supplementary Method 13). The model with the lowest BIC was selected as the optimal description; when two models were statistically indistinguishable (ΔBIC < 2), the more parsimonious model was preferred following the guideline of ref. ^63^. The structural parameters, symmetry constraints and BIC values for all candidate models are tabulated in Supplementary Table 2.

### Basis-motion analysis

**Extended Data Fig. 7:**
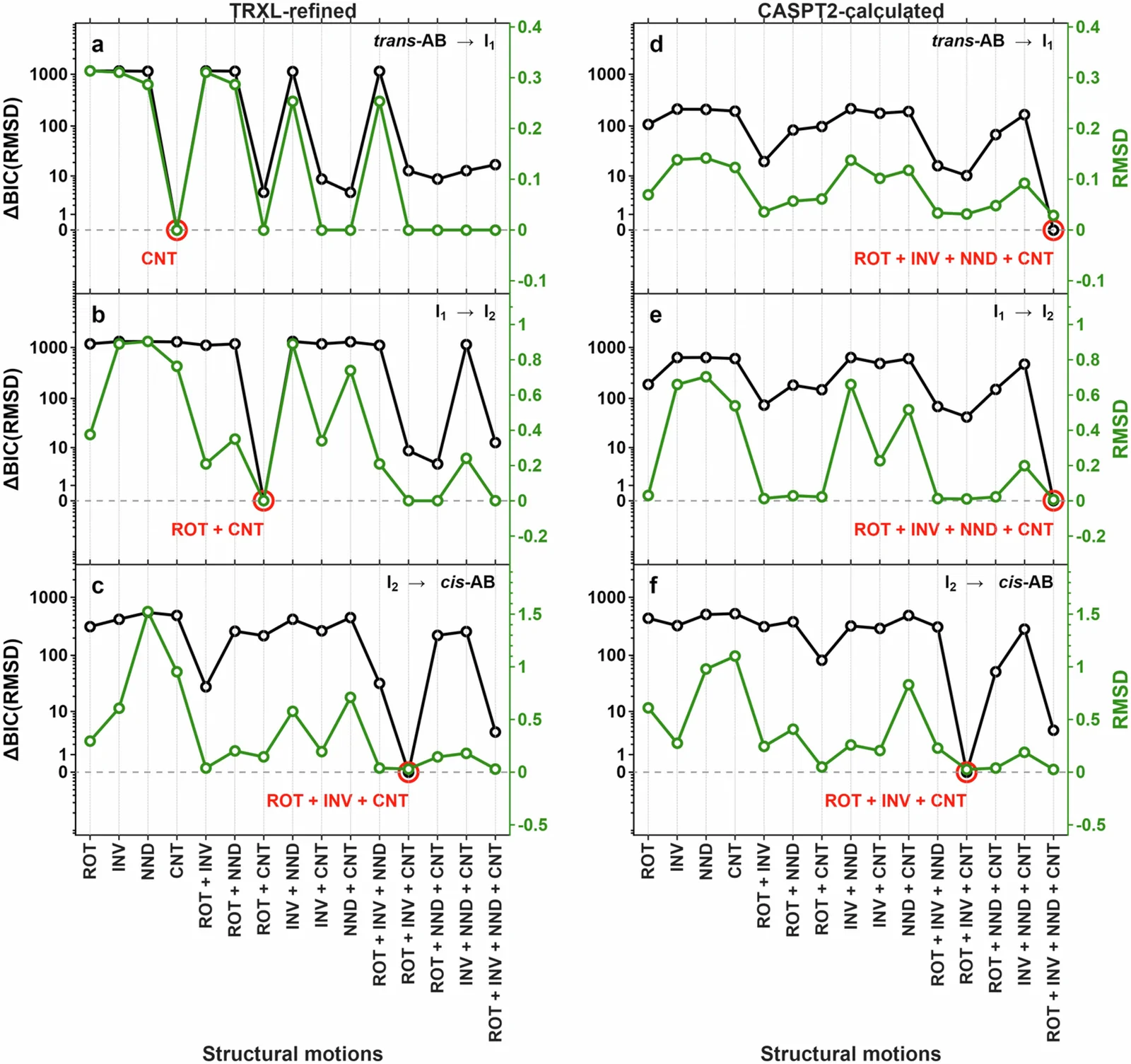
Basis-motion analysis (BMA) to unveil the motions responsible for structural transitions. **a–c**, Results for TRXL-refined structures: (**a**) *trans*-AB → I_1_, (**b**) I_1_ → I_2_ and (**c**) I_2_ → *cis*-AB. **d–f**, Results for CASPT2-calculated structures: (**d**) *trans*-AB → I_1_, (**e**) I_1_ → I_2_ and (**f**) I_2_ → *cis*-AB. In all panels, the right *y*-axis shows RMSD values (green) between the atomic coordinates of the target structure and the transformed structure obtained by applying a specific motion or combination of motions, and the left *y*-axis shows ΔBIC(RMSD) values (black), defined as the difference in BIC(RMSD) relative to the best (lowest-BIC) model. BIC(RMSD) serves as the sole criterion for model selection; ΔBIC(RMSD) = 0 corresponds to the optimal model. In each panel, the model with the lowest BIC is indicated by a red circle.

To identify the motions governing structural transitions between pairs of three-dimensional molecular structures, we developed BMA. Given an initial structure A and a target structure B, BMA evaluates how closely B can be reproduced by applying restricted combinations of the four basis motions to A. The similarity is quantified by the Kabsch-aligned root-mean-square deviation (RMSD) of atomic coordinates. All 15 combinatorial models were systematically evaluated with multi-phase global optimization, and an RMSD-based BIC was used for model selection (Supplementary Method 14). The RMSD and ΔBIC(RMSD) values from BMA analysis for TRXL-refined structures and CASPT2-calculated structures are shown in Extended Data Fig. 7a–c and 7d–f, respectively, and summarized in Supplementary Tables 3 and 4, respectively.

### Motional contribution analysis

To quantify the importance of each motion within an identified multi-motion optimal model, we developed a MoCA framework. The contribution of each motion was evaluated using a leave-one-out approach, which measures the increase in the optimized RMSD (or *χ*^2^) when a specific motion is excluded from the model while all remaining motions are re-optimized. This provides a robust measure of the functional necessity of each motion for reproducing the target structure, independent of the order in which the motions are applied (Supplementary Method 15). The MoCA results for the best models of the *trans*-AB → I_1_, I_1_ → I_2_, and I_2_ → *cis*-AB transitions are presented in Supplementary Table 5.

### Ab initio calculations

We performed multi-reference quantum chemistry calculations to determine the molecular structures of key species involved in the *trans*-to-*cis* photoisomerization of *trans*-AB. Before geometry optimizations, the underlying energetic landscape of the S_0_ and S_1_ states was explored by constructing two-dimensional potential energy surfaces obtained from rigid scans along the CNNC dihedral angle and the symmetric CNN bending coordinate, while keeping all remaining internal coordinates fixed (Supplementary Fig. 7). These coordinates provide a physically transparent description of the photoisomerization pathway and allow straightforward identification of the *trans* minimum and the two S_1_ local minima, as well as the approximate location of the *cis* minimum. These include the two ground-state structures, *trans*- and *cis*-AB, as well as the two intermediates in the S_1_(nπ*) state, denoted as I_1_ and I_2_. Although previous studies^7,22^ reported two minima on the S_1_ state surface, the optimized geometries of these minima have not been thoroughly discussed. To address this gap, we carried out full geometry optimizations for the four key structures directly at the extended multistate CASPT2 (XMS-CASPT2) level, using the BAGEL package (version 1.2.0)^64^, which provides fully analytic XMS-CASPT2 nuclear gradients^65,66^; these make unconstrained geometry optimizations at this level of theory feasible for a molecule of this size.

The active space for these calculations included ten electrons distributed across eight orbitals, comprising two *π* and two π* orbitals from the phenyl rings, *π* and π* orbitals from the azo group, and the lone-pair orbitals on the nitrogen atoms. The CASSCF reference was optimized using a state-averaged CASSCF approach including the two lowest singlet states (S_0_ and S_1_(nπ*)). Previous studies have shown that substantial CASPT2 corrections are necessary to accurately describe the low-lying excited-state manifold of azobenzene, particularly for states such as S_1_(nπ*) and S_2_(ππ*). To incorporate these essential corrections, we used the extended multistate version of the perturbative method (XMS-CASPT2), which effectively accounts for interactions among the CASSCF states. An imaginary shift of 0.2 a.u., a standard value for CASPT2 excited-state calculations, was used to prevent numerical instabilities caused by intruder states (accidental near-degeneracies) in the perturbative treatment^67^. Furthermore, symmetry constraints were disabled throughout the calculations, including geometry optimizations, to avoid potential bias. All calculations were performed in the gas phase using the aug-cc-pVDZ basis set with the cc-pVDZ-JKFIT auxiliary basis for density fitting. Solvent effects were not included in these geometry optimizations, but were incorporated in the atomic partial charge calculations for the cage-term calculation (Supplementary Methods 8 and 9). Supplementary Note 5 compares the CASPT2-calculated structures with previously reported theoretical structures.

### Ethics statement

This study did not involve human participants, animals or cell lines, and no ethical approval was required.

## Online content

Any methods, additional references, Nature Portfolio reporting summaries, source data, extended data, supplementary information, acknowledgements, peer review information; details of author contributions and competing interests; and statements of data and code availability are available at https://doi.org/10.1038/s41586-026-11068-4.

## Supplementary information

Supplementary Information Supplementary Methods 1–15, Notes 1–12, Figs 1–8, Tables 1–8 and References.

Supplementary Data 1 Atomic coordinates of all reported structures, comprising the TRXL-refined structures of I_1_, I_2_ and *cis*-AB and the CASPT2-calculated structures of *trans*-AB, I_1_, I_2_ and *cis*-AB, given in ångströms, compiled in a single document. The XYZ files are also provided separately in Supplementary Data 2–8, comprising seven structures in total.

Supplementary Data 2–8 Supplementary Data files 2–8 comprising seven individual atomic-coordinate datasets for the TRXL-refined and CASPT2-calculated structures of I_1_, I_2_, *cis*-AB and *trans*-AB. Each individual dataset is provided as an .xyz file. Supplementary Data 2 contains atomic coordinates of the TRXL-refined I_1_ intermediate, given in ångströms. Supplementary Data 3 contains atomic coordinates of the TRXL-refined I_2_ intermediate, given in ångströms. Supplementary Data 4 contains atomic coordinates of the TRXL-refined *cis*-AB, given in ångströms. Supplementary Data 5 contains atomic coordinates of the CASPT2-calculated *trans*-AB, given in ångströms. Supplementary Data 6 contains atomic coordinates of the CASPT2-calculated I_1_ intermediate, given in ångströms. Supplementary Data 7 contains atomic coordinates of the CASPT2-calculated I_2_ intermediate, given in ångströms. Supplementary Data 8 contains atomic coordinates of the CASPT2-calculated *cis*-AB, given in ångströms.

Supplementary Video 1 Structural trajectory of the *trans*-to-*cis* isomerization of azobenzene determined by TRXL (an oblique view selected to illustrate the overall structural changes), presented in the minimum-displacement frame as shown in Fig. 4b.

Supplementary Video 2 Structural trajectory of the *trans*-to-*cis* isomerization of azobenzene determined by TRXL (front, top and side views), presented in the minimum-displacement frame as shown in Fig. 4b.

Supplementary Video 3 Structural trajectory of the *trans*-to-*cis* isomerization of azobenzene determined by TRXL (an oblique view selected to illustrate the overall structural changes), presented in the N–N fixed frame where only the phenyl rings move while the azo group remains stationary, as illustrated in Supplementary Fig. 8.

Supplementary Video 4 Structural trajectory of the *trans*-to-*cis* isomerization of azobenzene determined by TRXL (front, top and side views), presented in the N–N fixed frame as illustrated in Supplementary Fig. 8.

Supplementary Video 5 Overlay comparison of the minimum-displacement and N–N fixed frames for the *trans*-to-*cis* isomerization of azobenzene (an oblique view selected to illustrate the overall structural changes).

Supplementary Video 6 Overlay comparison of the minimum-displacement and N–N fixed frames for the *trans*-to-*cis* isomerization of azobenzene (front, top and side views).

Peer Review File

## Source data

Source Data Fig. 2

Source Data Fig. 3

Source Data Fig. 4

Source Data Extended Data Fig. 1

Source Data Extended Data Fig. 2

Source Data Extended Data Fig. 3

Source Data Extended Data Fig. 4

Source Data Extended Data Fig. 5

## Figures and Tables

**Extended Data Table 1:**
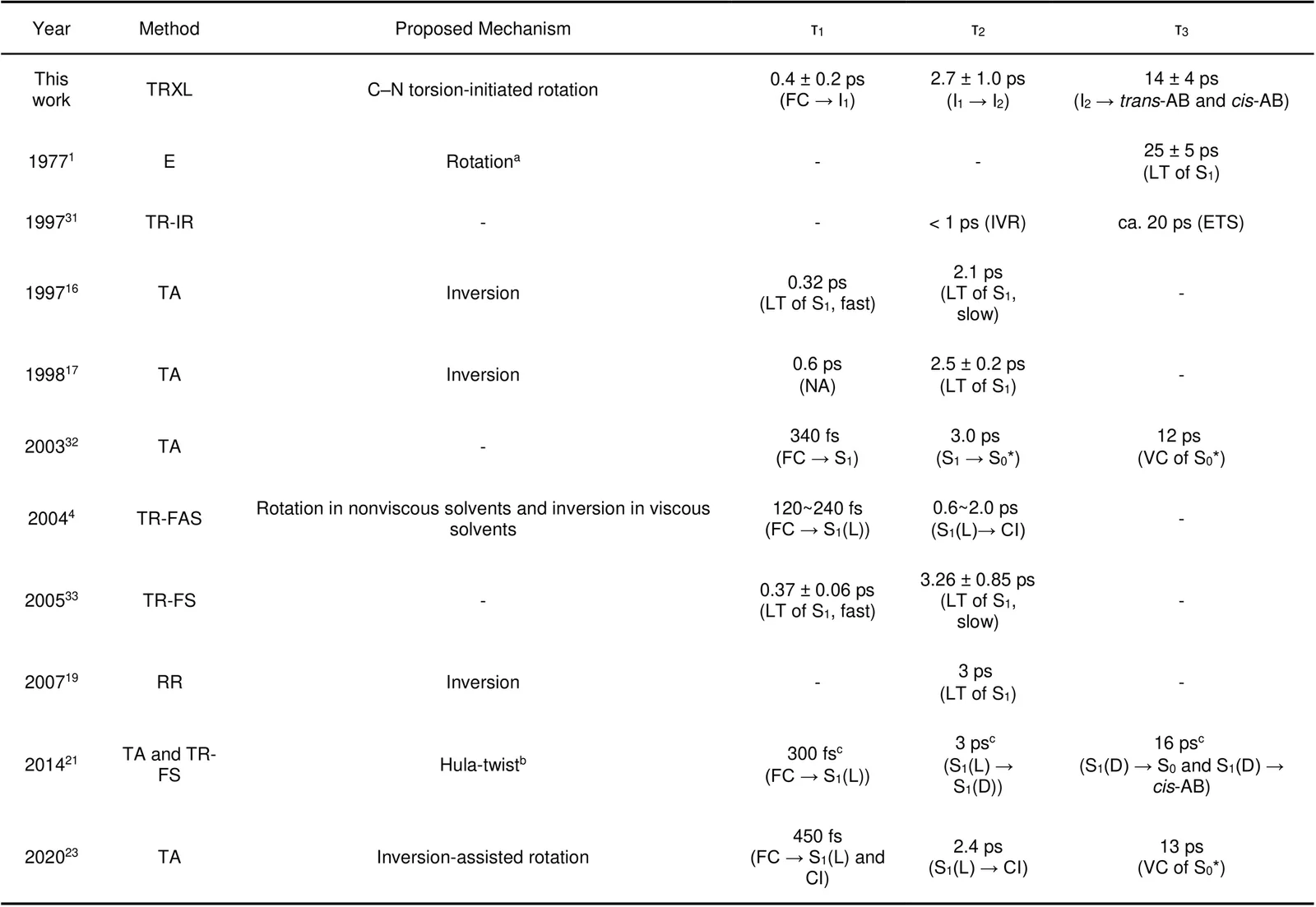
Summary of experimental studies on the photoisomerization of *trans*-AB reported in the literature

This table lists studies where *trans*-AB was excited to the S_1_(nπ*) state, as in the present work. Our study is listed first, followed by other studies in chronological order. For each study, the employed experimental methods, proposed mechanisms, reported time constants with associated errors (where available), and the assignments of these time constants (if provided) are indicated. Abbreviations used in this table are explained in the footnote below.

E = Emission spectroscopy, TR-IR = Time-resolved infrared spectroscopy, TA = Transient absorption spectroscopy, TR-FAS = Time-resolved fluorescence anisotropy spectroscopy, RR = Resonance Raman spectroscopy, TR-FS = Time-resolved fluorescence spectroscopy.

FC = Franck–Condon state, I_1_ = First intermediate, I_2_ = Second intermediate, LT = Lifetime, S_1_ = First singlet excited state of *trans*-azobenzene, IVR = Intramolecular vibrational energy redistribution, ETS = Energy transfer to solvent, NA = Not assigned, S_0_ = Ground state of *trans*-azobenzene, S_0_* = Vibrationally hot S_0_, VC = Vibrational cooling, CI = Conical intersection between the S_1_ and S_0_ surfaces, S_1_(L) = Local minimum on the S_1_ surface, S_1_(D) = Dark intermediate on the S_1_ surface.

^a^In the original paper, this mechanism was referred to as “torsion” instead of “rotation”.

^b^The hula-twist mechanism was proposed primarily based on the observation that the time constants of isomerization kinetics remained unaffected by solvent viscosity, as demonstrated in experiments across various solvents, rather than on direct observation of molecular motion. The limited influence of solvent viscosity on the isomerization time constant suggests that the solute molecule’s volume remains relatively constant during the process, indicating a volume-conserving pathway.

^c^Among the previous studies, both the observed time constants and their assignments reported in this study show the closest agreement with those from the present TRXL work.

**Extended Data Table 2:**
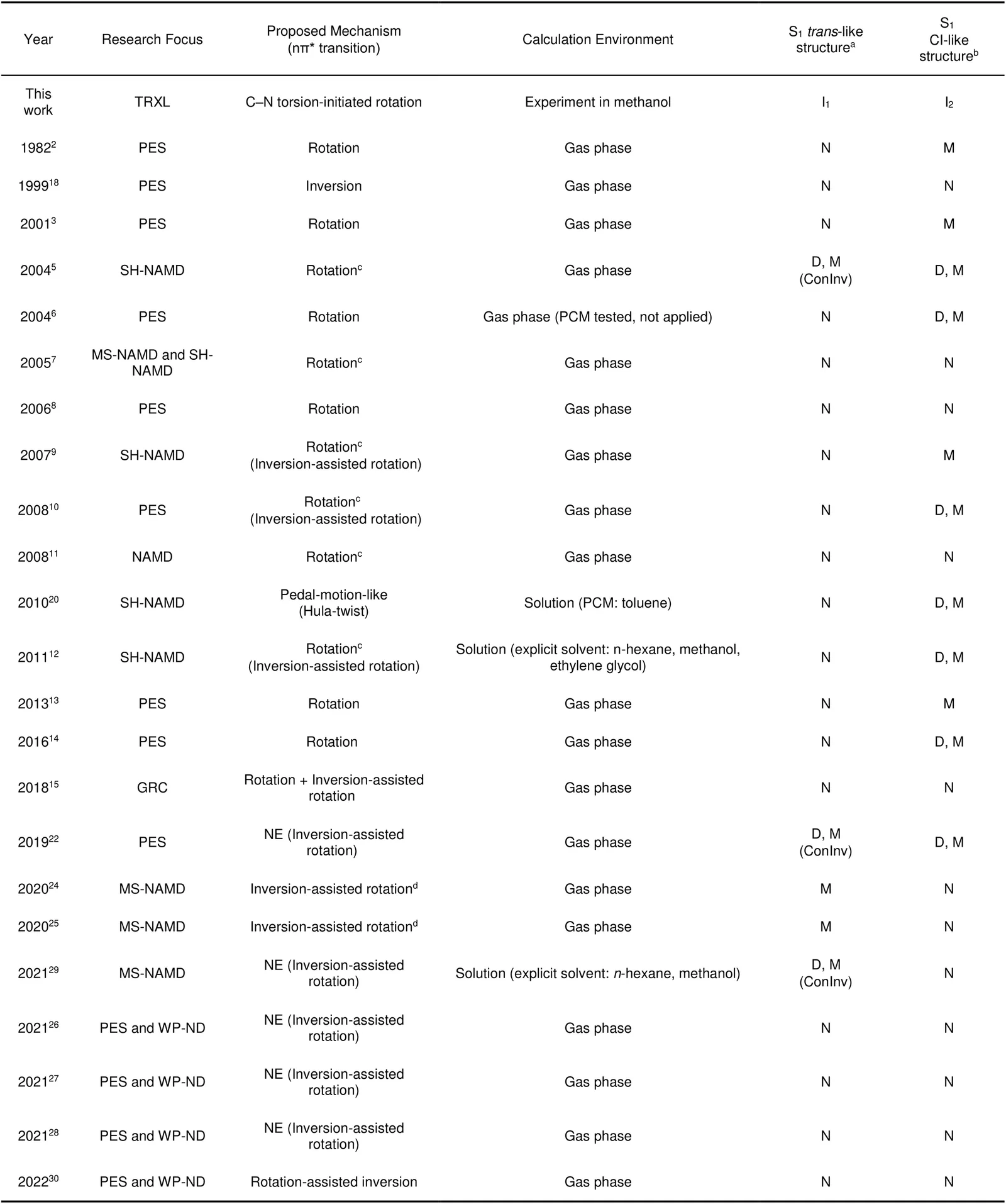
Summary of theoretical studies on the photoisomerization of *trans*-AB

For comparison, the mechanism identified through our TRXL experiment is listed first, followed by those proposed in previous theoretical studies in chronological order. The proposed mechanisms from each study are listed, with our interpretation indicated in parentheses.

CI-like = (conical intersection–like), PES = Potential energy surface calculation, MS-NAMD = Multiple spawning Nonadiabatic Molecular Dynamics (NAMD) simulation, SH-NAMD = Surface hopping NAMD, NAMD = Nonadiabatic Molecular Dynamics simulation, GRC = Global Reaction Coordinate calculation, WP-ND = Nonadiabatic wave packet dynamics, PCM = Polarizable Continuum Model, NE = No explicit mention of the mechanism, D = Detected, M = Mentioned, N = Neither detected nor mentioned, ConInv = concerted inversion.

^a^This structure corresponds to I_1_ captured by TRXL.

^b^This structure corresponds to I_2_ captured by TRXL.

^c^In the original paper, this mechanism was referred to as “torsion” instead of “rotation”.

^d^In the original paper, this mechanism was referred to as “inversion-assisted torsion” instead of “inversion-assisted rotation”.

**Extended Data Table 3:**
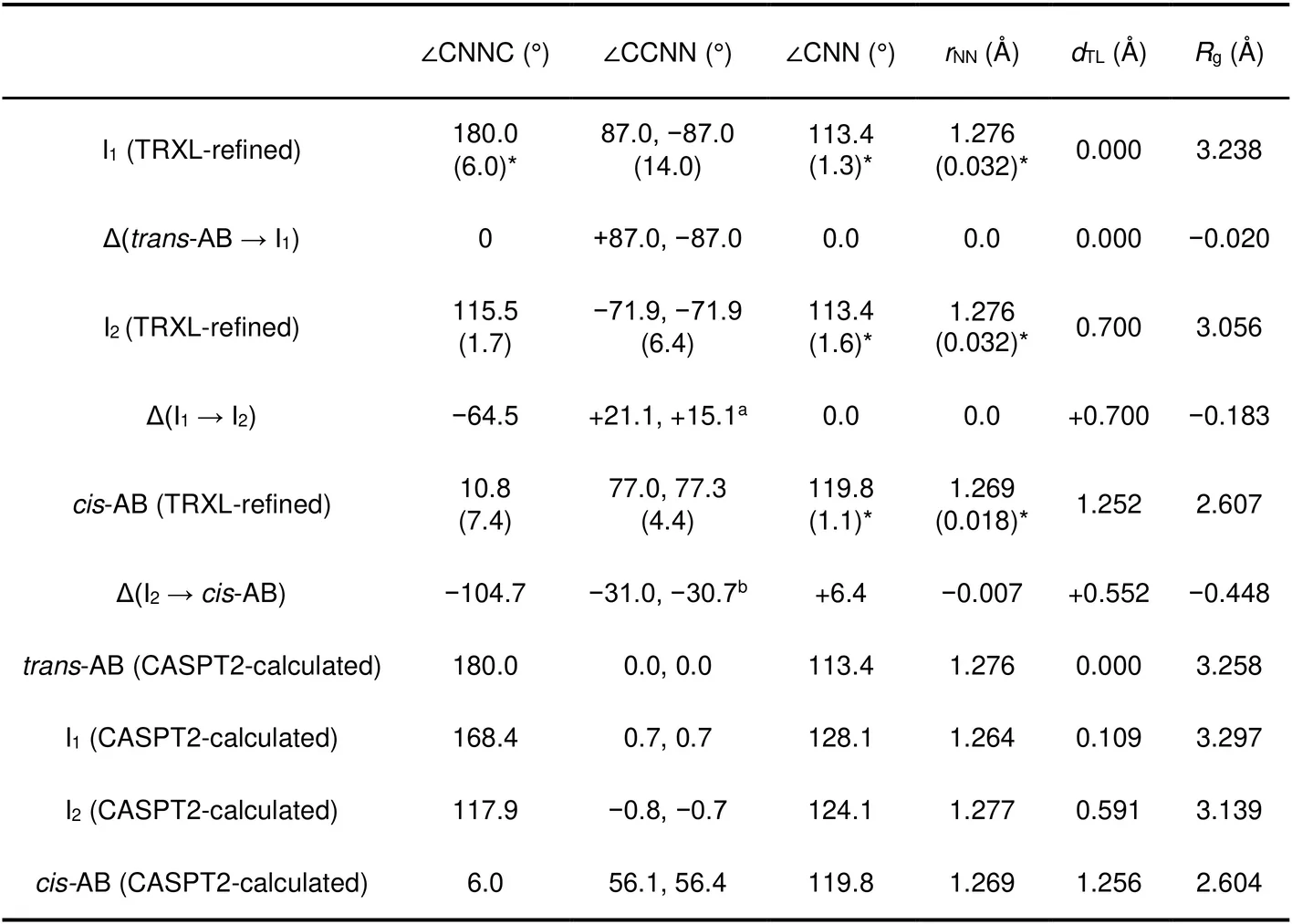
Structural parameters of *trans*-AB, I_1_, I_2_ and *cis*-AB

Structural parameters of *trans*-AB, I_1_, I_2_ and *cis*-AB determined from TRXL fitting are compared with those from CASPT2 calculations. Values in parentheses denote 1*σ* uncertainties estimated by profile likelihood analysis (Δ*χ*^2^ = 1 boundary). For each parameter, the profile likelihood was obtained by scanning the parameter of interest while re-optimizing all other free parameters. The two points at which *χ*^2^ exceeds its minimum by 1 are both referenced to the same minimum of the profile, and the uncertainty is taken from the larger of the two displacements. Where a crossing point was retained on only one side, only its magnitude taken from that side is reported; this applies only to ∠CCNN of *cis*-AB (Supplementary Method 4). The ∠CCNN columns list the two dihedral angles corresponding to the two ortho-carbon atoms. The changes in structural parameters for the TRXL-refined structures are shown for each transition (rows starting with Δ).

*Parameter held fixed during fitting. The quoted uncertainty represents the profile likelihood sensitivity: the parameter was scanned with all free parameters re-optimized at each scan point, and the quoted uncertainty value is the displacement from the reference minimum of the resulting profile to its Δ*χ*^2^ = 1 crossing point, following the convention described in Supplementary Method 4. Because the parameter was not refined, the reference minimum need not coincide with the value at which the parameter was held fixed; the uncertainty value measures how tightly the data would constrain the parameter if it were released.

^a^Each phenyl ring possesses local *C*_2_ symmetry, with two equivalent *ortho* carbon positions relative to the C–N bond. Consequently, ∠CCNN = *α* and ∠CCNN = *α* + 180° describe the same ring orientation, giving ∠CCNN a periodicity of 180°. The shortest-path rotation from I_1_ to I_2_ is therefore +21.1° for Ring 1 (87.0° → −71.9° ≡ 108.1°, Δ = +21.1°), not the apparent −71.9 − 87.0 = −158.9°. (Ring 2: −87.0° → −71.9°, Δ = +15.1°).

^b^By the same periodicity described in a, Ring 1: −71.9° ≡ 108.1° → 77.0°, Δ = −31.0°; Ring 2: −71.9° ≡ 108.1° → 77.3°, Δ = −30.7°.

## Data Availability

The atomic coordinates of the CASPT2-calculated and TRXL-refined structures are provided as Supplementary Data 1–8. All other data supporting the findings of this study, including the complete raw diffraction images, are too large to be deposited in a publicly available repository but are available from the corresponding author upon request. Source data are provided with this paper.

## Code availability

The complete data-processing and analysis workflow (preprocessing, PEPC, SVD and kinetic analysis, molecular dynamics solvent cage, and SSPM, BMA and MoCA structural analysis) is available as a Code Ocean capsule at https://doi.org/10.24433/CO.0502770.v1 (ref. ^68^). Any additional code or scripts supporting the findings are available from the corresponding author upon request.

## References

[1] Struve, W. S. Emission from the 1 (n, *π**) state of azobenzene: spectrum and ultrashort decay time. *Chem. Phys. Lett.* **46**, 15–19 (1977).

[2] Monti, S., Orlandi, G. & Palmieri, P. Features of the photochemically active state surfaces of azobenzene. *Chem. Phys.* **71**, 87–99 (1982).

[3] Ishikawa, T., Noro, T. & Shoda, T. Theoretical study on the photoisomerization of azobenzene. *J. Chem. Phys.* **115**, 7503–7512 (2001).

[4] Chang, C.-W., Lu, Y.-C., Wang, T.-T. & Diau, E. W.-G. Photoisomerization dynamics of azobenzene in solution with S_1_ excitation: a femtosecond fluorescence anisotropy study. *J. Am. Chem. Soc.* **126**, 10109–10118 (2004). 10.1021/ja049215p 15303887

[5] Ciminelli, C., Granucci, G. & Persico, M. The photoisomerization mechanism of azobenzene: a semiclassical simulation of nonadiabatic dynamics. *Chem. Eur. J.* **10**, 2327–2341 (2004). 10.1002/chem.200305415 15112222

[6] Diau, E. W.-G. A new trans-to-cis photoisomerization mechanism of azobenzene on the S1 (n, *π**) surface. *J. Phys. Chem. A* **108**, 950–956 (2004).

[7] Toniolo, A., Ciminelli, C., Persico, M. & Martínez, T. J. Simulation of the photodynamics of azobenzene on its first excited state: comparison of full multiple spawning and surface hopping treatments. *J. Chem. Phys.* **123**, 234308 (2005). 10.1063/1.2134705 16392921

[8] Crecca, C. R. & Roitberg, A. E. Theoretical study of the isomerization mechanism of azobenzene and disubstituted azobenzene derivatives. *J. Phys. Chem. A* **110**, 8188–8203 (2006). 10.1021/jp057413c 16805507

[9] Granucci, G. & Persico, M. Excited state dynamics with the direct trajectory surface hopping method: azobenzene and its derivatives as a case study. *Theor. Chem. Acc.* **117**, 1131–1143 (2007).

[10] Conti, I., Garavelli, M. & Orlandi, G. The different photoisomerization efficiency of azobenzene in the lowest n*π** and ππ* singlets: the role of a phantom state. *J. Am. Chem. Soc.* **130**, 5216–5230 (2008). 10.1021/ja710275e 18335990

[11] Yuan, S., Dou, Y., Wu, W., Hu, Y. & Zhao, J. Why does *trans*-azobenzene have a smaller isomerization yield for *ππ** excitation than for n*π** excitation? *J. Phys. Chem. A* **112**, 13326–13334 (2008). 10.1021/jp806227x 19053550

[12] Cusati, T., Granucci, G. & Persico, M. Photodynamics and time-resolved fluorescence of azobenzene in solution: a mixed quantum-classical simulation. *J. Am. Chem. Soc.* **133**, 5109–5123 (2011). 10.1021/ja1113529 21401136

[13] Harabuchi, Y., Ishii, M., Nakayama, A., Noro, T. & Taketsugu, T. A multireference perturbation study of the NN stretching frequency of trans-azobenzene in n*π** excitation and an implication for the photoisomerization mechanism. *J. Chem. Phys.* **138**, 064305 (2013). 10.1063/1.4790611 23425469

[14] Casellas, J., Bearpark, M. J. & Reguero, M. Excited-state decay in the photoisomerisation of azobenzene: a new balance between mechanisms. *ChemPhysChem* **17**, 3068–3079 (2016). 10.1002/cphc.201600502 27398810

[15] Tavadze, P. et al. A machine-driven hunt for global reaction coordinates of azobenzene photoisomerization. *J. Am. Chem. Soc.* **140**, 285–290 (2018). 10.1021/jacs.7b10030 29235856

[16] Nägele, T., Hoche, R., Zinth, W. & Wachtveitl, J. Femtosecond photoisomerization of *cis*-azobenzene. *Chem. Phys. Lett.* **272**, 489–495 (1997).

[17] Lednev, I. K. et al. Femtosecond time-resolved UV–visible absorption spectroscopy of *trans*-azobenzene: dependence on excitation wavelength. *Chem. Phys. Lett.* **290**, 68–74 (1998).

[18] Cattaneo, P. & Persico, M. An ab initio study of the photochemistry of azobenzene. *Phys. Chem. Chem. Phys.* **1**, 4739–4743 (1999).

[19] Stuart, C. M., Frontiera, R. R. & Mathies, R. A. Excited-state structure and dynamics of *cis*- and *trans*-azobenzene from resonance Raman intensity analysis. *J. Phys. Chem. A* **111**, 12072–12080 (2007). 10.1021/jp0751460 17985852

[20] Böckmann, M., Doltsinis, N. L. & Marx, D. Nonadiabatic hybrid quantum and molecular mechanic simulations of azobenzene photoswitching in bulk liquid environment. *J. Phys. Chem. A* **114**, 745–754 (2010). 10.1021/jp910103b 19928885

[21] Quick, M. et al. Photoisomerization dynamics and pathways of *trans*- and *cis*-azobenzene in solution from broadband femtosecond spectroscopies and calculations. *J. Phys. Chem. B* **118**, 8756–8771 (2014). 10.1021/jp504999f 24983924

[22] Aleotti, F. et al. Multidimensional potential energy surfaces resolved at the RASPT2 level for accurate photoinduced isomerization dynamics of azobenzene. *J. Chem. Theory Comput.* **15**, 6813–6823 (2019). 10.1021/acs.jctc.9b00561 31647648

[23] Otolski, C. J., Raj, A. M., Ramamurthy, V. & Elles, C. G. Spatial confinement alters the ultrafast photoisomerization dynamics of azobenzenes. *Chem. Sci.* **11**, 9513–9523 (2020). 10.1039/d0sc03955a PMC8162038 34094217

[24] Yu, J. K., Bannwarth, C., Hohenstein, E. G. & Martínez, T. J. Ab initio nonadiabatic molecular dynamics with hole–hole Tamm–Dancoff approximated density functional theory. *J. Chem. Theory Comput.* **16**, 5499–5511 (2020). 10.1021/acs.jctc.0c00644 32786902

[25] Yu, J. K., Bannwarth, C., Liang, R., Hohenstein, E. G. & Martínez, T. J. Nonadiabatic dynamics simulation of the wavelength-dependent photochemistry of azobenzene excited to the n*π** and *ππ** excited states. *J. Am. Chem. Soc.* **142**, 20680–20690 (2020). 10.1021/jacs.0c09056 33228358

[26] Gu, B. et al. Photoisomerization transition state manipulation by entangled two-photon absorption. *Proc. Natl Acad. Sci. USA* **118**, e2116868118 (2021). 10.1073/pnas.2116868118 PMC8617409 34799455

[27] Keefer, D. et al. Imaging conical intersection dynamics during azobenzene photoisomerization by ultrafast X-ray diffraction. *Proc. Natl Acad. Sci. USA* **118**, e2022037118 (2021). 10.1073/pnas.2022037118 PMC7826416 33436412

[28] Keefer, D. et al. Diffractive imaging of conical intersections amplified by resonant infrared fields. *J. Am. Chem. Soc.* **143**, 13806–13815 (2021). 10.1021/jacs.1c06068 34402612

[29] Liang, R. First-principles nonadiabatic dynamics simulation of azobenzene photodynamics in solutions. *J. Chem. Theory Comput.* **17**, 3019–3030 (2021). 10.1021/acs.jctc.1c00105 33882676

[30] Rouxel, J. R. et al. Coupled electronic and nuclear motions during azobenzene photoisomerization monitored by ultrafast electron diffraction. *J. Chem. Theory Comput.* **18**, 605–613 (2022). 10.1021/acs.jctc.1c00792 35073085

[31] Hamm, P., Ohline, S. M. & Zinth, W. Vibrational cooling after ultrafast photoisomerization of azobenzene measured by femtosecond infrared spectroscopy. *J. Chem. Phys.* **106**, 519–529 (1997).

[32] Satzger, H. et al. Fluorescence spectra of *trans*- and *cis*-azobenzene–emission from the Franck–Condon state. *Chem. Phys. Lett.* **372**, 216–223 (2003).

[33] Pancur, T. et al. Femtosecond fluorescence up-conversion spectroscopy of a rotation-restricted azobenzene after excitation to the S_1_ state. *Phys. Chem. Chem. Phys.* **7**, 1985–1989 (2005). 10.1039/b419236b 19787903

[34] Ihee, H. et al. Ultrafast X-ray diffraction of transient molecular structures in solution. *Science* **309**, 1223–1227 (2005). 10.1126/science.1114782 16020695

[35] Ihee, H. Visualizing solution-phase reaction dynamics with time-resolved X-ray liquidography. *Acc. Chem. Res.* **42**, 356–366 (2009). 10.1021/ar800168v 19117426

[36] Biasin, E. et al. Femtosecond X-ray scattering study of ultrafast photoinduced structural dynamics in solvated [Co(terpy)_2_]^2+^. *Phys. Rev. Lett.* **117**, 013002 (2016). 10.1103/PhysRevLett.117.013002 27419566

[37] Choi, E. H., Lee, Y., Heo, J. & Ihee, H. Reaction dynamics studied via femtosecond X-ray liquidography at X-ray free-electron lasers. *Chem. Sci.* **13**, 8457–8490 (2022). 10.1039/d2sc00502f PMC9337737 35974755

[38] Nimmrich, A. et al. Solvent-dependent structural dynamics in the ultrafast photodissociation reaction of triiodide observed with time-resolved X-ray solution scattering. *J. Am. Chem. Soc.* **145**, 15754–15765 (2023). 10.1021/jacs.3c00484 PMC10375522 37163700

[39] Powers-Riggs, N. E. et al. Characterization of deformational isomerization potential and interconversion dynamics with ultrafast X-ray solution scattering. *J. Am. Chem. Soc.* **146**, 13962–13973 (2024). 10.1021/jacs.4c00817 38727611

[40] Mara, M. W. et al. Characterizing ultrafast intersystem crossing pathways in molecular Pt dimers using time-resolved wide-angle X-ray scattering. *J. Phys. Chem. Lett.* **16**, 7120–7126 (2025). 10.1021/acs.jpclett.5c01245 40609039

[41] Ki, H., Gu, J., Cha, Y., Lee, K. W. & Ihee, H. Projection to extract the perpendicular component (PEPC) method for extracting kinetics from time-resolved data. *Struct. Dyn.* **10**, 034103 (2023). 10.1063/4.0000189 PMC10306411 37388296

[42] Rajagopal, S., Schmidt, M., Anderson, S., Ihee, H. & Moffat, K. Analysis of experimental time-resolved crystallographic data by singular value decomposition. *Acta Crystallogr. D* **60**, 860–871 (2004). 10.1107/S0907444904004160 15103131

[43] Ashiotis, G. et al. The fast azimuthal integration Python library: pyFAI. *J. Appl. Crystallogr.* **48**, 510–519 (2015). 10.1107/S1600576715004306 PMC4379438 25844080

[44] Biasin, E. et al. Anisotropy enhanced X-ray scattering from solvated transition metal complexes. *J. Synchrotron Rad.* **25**, 306–315 (2018). 10.1107/S1600577517016964 29488907

[45] Choi, E. H. et al. Filming ultrafast roaming-mediated isomerization of bismuth triiodide in solution. *Nat. Commun.* **12**, 4732 (2021). 10.1038/s41467-021-25070-z PMC8342516 34354075

[46] Ki, H. et al. Optical Kerr effect of liquid acetonitrile probed by femtosecond time-resolved X-ray liquidography. *J. Am. Chem. Soc.* **143**, 14261–14273 (2021). 10.1021/jacs.1c06088 34455778

[47] Heo, J. et al. Determining the charge distribution and the direction of bond cleavage with femtosecond anisotropic X-ray liquidography. *Nat. Commun.* **13**, 522 (2022). 10.1038/s41467-022-28168-0 PMC8792042 35082327

[48] Kim, T. K. et al. Spatiotemporal reaction kinetics of an ultrafast photoreaction pathway visualized by time-resolved liquid X-ray diffraction. *Proc. Natl Acad. Sci. USA* **103**, 9410–9415 (2006). 10.1073/pnas.0601958103 PMC1478163 16772380

[49] Christensen, M. et al. Time-resolved X-ray scattering of an electronically excited state in solution. Structure of the ^3^A_2u_ state of tetrakis-μ-pyrophosphitodiplatinate (II). *J. Am. Chem. Soc.* **131**, 502–508 (2009). 10.1021/ja804485d 19140790

[50] Canton, S. E. et al. Visualizing the non-equilibrium dynamics of photoinduced intramolecular electron transfer with femtosecond X-ray pulses. *Nat. Commun.* **6**, 6359 (2015). 10.1038/ncomms7359 PMC4366532 25727920

[51] Kim, K. H. et al. Direct observation of bond formation in solution with femtosecond X-ray scattering. *Nature* **518**, 385–389 (2015). 10.1038/nature14163 25693570

[52] Van Driel, T. B. et al. Atomistic characterization of the active-site solvation dynamics of a model photocatalyst. *Nat. Commun.* **7**, 13678 (2016). 10.1038/ncomms13678 PMC5133712 27892472

[53] Choi, E. H. et al. Structural dynamics of bismuth triiodide in solution triggered by photoinduced ligand-to-metal charge transfer. *J. Phys. Chem. Lett.* **10**, 1279–1285 (2019). 10.1021/acs.jpclett.9b00365 30835478

[54] Kjær, K. S. et al. Finding intersections between electronic excited state potential energy surfaces with simultaneous ultrafast X-ray scattering and spectroscopy. *Chem. Sci.* **10**, 5749–5760 (2019). 10.1039/c8sc04023k PMC6568243 31293761

[55] Kong, Q. Y. et al. Initial metal–metal bond breakage detected by fs X-ray scattering in the photolysis of Ru_3_(CO)_12_ in cyclohexane at 400 nm. *Photochem. Photobiol. Sci.* **18**, 319–327 (2019). 10.1039/c8pp00420j 30628601

[56] Leshchev, D. et al. Sub-nanosecond secondary geminate recombination in mercury halides HgX_2_ (X = I, Br) investigated by time-resolved X-ray scattering. *J. Chem. Phys.* **151**, 054310 (2019).

[57] Kim, J. G. et al. Mapping the emergence of molecular vibrations mediating bond formation. *Nature* **582**, 520–524 (2020). 10.1038/s41586-020-2417-3 32581378

[58] Biasin, E. et al. Direct observation of coherent femtosecond solvent reorganization coupled to intramolecular electron transfer. *Nat. Chem.* **13**, 343–349 (2021). 10.1038/s41557-020-00629-3 33589787

[59] Ki, H. et al. Photoactivation of triosmium dodecacarbonyl at 400 nm probed with time-resolved X-ray liquidography. *Chem. Commun.* **58**, 7380–7383 (2022). 10.1039/d2cc02438a 35695475

[60] Lee, Y. et al. Cerium photocatalyst in action: structural dynamics in the presence of substrate visualized via time-resolved X-ray liquidography. *J. Am. Chem. Soc.* **145**, 23715–23726 (2023). 10.1021/jacs.3c08166 PMC10623567 37856865

[61] Leshchev, D. et al. Revealing excited-state trajectories on potential energy surfaces with atomic resolution in real time. *Angew. Chem. Int. Ed.* **62**, e202304615 (2023). 10.1002/anie.202304615 37114904

[62] Kim, D. et al. Excited-state structural dynamics of the cubane-type metal cluster [Cu_4_I_4_(py)_4_] explored by time-resolved X-ray liquidography. *Adv. Sci.* **12**, 2414970 (2025). 10.1002/advs.202414970 PMC11984917 39951326

[63] Kass, R. E. & Raftery, A. E. Bayes factors. *J. Am. Stat. Assoc.* **90**, 773–795 (1995).

[64] Shiozaki, T. BAGEL: brilliantly advanced general electronic-structure library. *W**iley Interdiscip. Rev. Comput. Mol. Sci.* **8**, e1331 (2018).

[65] MacLeod, M. K. & Shiozaki, T. Communication: automatic code generation enables nuclear gradient computations for fully internally contracted multireference theory. *J. Chem. Phys.* **142**, 051103 (2015). 10.1063/1.4907717 25662628

[66] Vlaisavljevich, B. & Shiozaki, T. Nuclear energy gradients for internally contracted complete active space second-order perturbation theory: multistate extensions. *J. Chem. Theory Comput.* **12**, 3781–3787 (2016). 10.1021/acs.jctc.6b00572 27388038

[67] Forsberg, N. & Malmqvist, P.-Å. Multiconfiguration perturbation theory with imaginary level shift. *Chem. Phys. Lett.* **274**, 196–204 (1997).

[68] Kim, J. et al. X-ray liquidography decodes complex motions in azobenzene isomerization*. Code Ocean* 10.24433/CO.0502770.v1 (2026). PMC13668673 42816611

