# Supplementary material for: X-ray liquidography decodes complex motions in azobenzene isomerization
Source: Nature. 2026 Sep 30;658(8136):658–65. doi: 10.1038/s41586-026-11068-4 (PMC13668673; doi:10.1038/s41586-026-11068-4)
Supplement: Supplementary file 1 [file 41586_2026_11068_MOESM10_ESM.pdf]

nature portfolio

Peer Review File

X-ray liquidography decodes complex motions in azobenzene isomerisation

Corresponding Author: Professor Hyotcherl Ihee

Any redactions in this file are there to maintain patient confidentiality, the confidentiality of unpublished data, or to remove third-party material.

This file contains all reviewer reports in order by version, followed by all author rebuttals in order by version.

Version 0:

Reviewer comments:

Referee #1

(Remarks to the Author)
See attached document.

Referee #2

(Remarks to the Author)
Summary of the key results

In the study 'Complex motions in trans-to-cis isomerization of azobenzene decoded by femtosecond X-ray liquidography', Kim et al. report on the trans-to-cis photoisomerization process of azobenzene. Through pump-probe experiments with laser excitation at 400 nm to induce the nπ* transition from S0 to S1 and structurally sensitive X-ray probe, the authors are able to resolve two intermediate states in the photoisomerization of trans-to-cis azobenzene. Trans-azobenzene excited to the Franck-Condon state relaxes to intermediate 1 (I1) with a time constant of around 0.4 ps, this is followed by a transition into a second intermediate (I2) with a time constant around 2.4 ps and final population of cis-azobenzene with a time constant around 13.7 ps.

By testing sets of motions derived from classical mechanisms proposed for the photoisomerization of trans-to-cis azobenzene in literature, the authors refine theoretical CASPT2-calculated structures of I1 and I2 by chi-squared fitting to species-associated difference scattering (SADS) curves calculated from the experimental data. Based on a systematic scan for parsimonious motions and basis-motion analysis, the authors determine that the trans-to-cis isomerization of azobenzene can be explained by out-of-plane hula-twist (OHT) for trans-azo to I1 and OHT and rotation (ROT) for I1 to I2 and I2 to cis-azobenzene.

Originality and significance

The study introduces a novel application of time-resolved X-ray liquidography (TRXL) to a small organic molecule, providing direct structural insight into the trans-to-cis photoisomerization mechanism of azobenzene in methanol solution. The authors advance the understanding of this reaction by identifying two intermediates, refining their structure, and elucidating the parsimonious structural motions underlying the photoisomerization process.
If the authors can address the points raised below, this work demonstrates that time-resolved X-ray scattering can yield detailed structural information on photoreaction dynamics of small organic molecules in solution. It represents an important step toward establishing TRXL as a technique for such investigations and highlights the type of insights that can be obtained, potentially paving the way for future studies of photoactive organic molecules.
While the study is novel and potentially impactful, the authors should address the following comments, questions, and

suggestions before the manuscript can be considered for publication in Nature.

Data & methodology

TRXL is an established technique for studying photoreactions, particularly in molecules containing heavy atoms. The authors convincingly demonstrate that the technique can be extended to molecules without heavy atoms. The data quality appears good, and the authors are able to separate the dominant solvent contribution from solute and cage contributions, showing that structural changes in azobenzene can be resolved. However, based on the current manuscript, it remains unclear whether the structure of intermediate states and the isomerization pathways can be successfully refined.

The methods and analysis involve numerous separation and correction steps on both the experimental and simulation sides, making the workflow difficult to follow. In several sections, the manuscript does not sufficiently describe these processes. A figure summarizing the sequence of treatments and corrections applied to experimental and simulated data—both before and during analysis—would greatly improve clarity. This is particularly important given that the authors report a novel application of TRXL to a small organic molecule in solution; referencing procedures from previous studies on heavy-atom systems may not be sufficient.

The overall presentation quality is good, but some inconsistencies and contradictions were noted. The authors should carefully revise and proofread the Methods section and Supplementary Information. Specific comments are provided in the "Appropriate use of statistics and treatment of uncertainties" and "Suggested Improvements" sections of this report.

Appropriate use of statistics and treatment of uncertainties

Some aspects of the use of statistics and treatment of uncertainties require clarification:
- The authors report an instrument response function of approximately 170 fs, which presumably limits the temporal resolution. However, they specify uncertainties of 20–50 fs, and a time constant of around 110 fs for the spatiotemporal analysis of the anisotropic fs-TRXL data for trans-azobenzene. Could the authors clarify how these values are consistent with the stated instrument response?
- The authors employ chi-squared statistics using σ, the standard error of the mean for ΔS at time delay t. Please provide details on how σ was calculated.
- While the use of chi-squared statistics may be appropriate, the reported chi-squared values appear quite large. The manuscript does not specify the number of data points, the number of fitting parameters in the structural refinement, or the degrees of freedom. Without this information, the reported chi-squared values and their differences appear arbitrary, making it difficult to assess the quality of the fits or the significance of differences between models. The authors should report these details and present chi-squared values in a way that allows more intuitive evaluation, for example reduced chi-squared. Including a table summarizing the fitting parameters for each model would be helpful.
- The authors describe a systematic scan for parsimonious motions to identify minimal structural changes. However, it is unclear whether the statistical significance of different motions or combinations of motions was tested. For example, was an F-test or information criteria such as AIC or BIC applied?

Conclusions: robustness, validity, reliability

The strength of the conclusions depends on the clarity and rigor of the methodology and statistical analysis. At present, the authors must address the comments and questions outlined in this review before the robustness, validity, and reliability of the reported conclusions can be fully assessed.

Of particular importance is a detailed discussion of the experimental conditions, specifically the high pump power and excitation energy. The authors should clarify whether multi-photon excitation of azobenzene is a plausible scenario under these conditions. Additionally, the authors should clarify whether the methanol solvent was directly heated during the azobenzene measurements and, if so, discuss how this might influence the observed dynamics and the relative contributions of solvent, solute, and cage signals. Furthermore, the manuscript should consider how vibrational modes activated through thermal and kinetic energy in the excited state affect the interpretation and relevance of the identified parsimonious motions. Finally, the main article must clearly state that these motions represent parsimonious motions and that other motions might be possible. Specific recommendations, including references to relevant sections of the manuscript and Supplementary Information, are provided below.

Suggested improvements

To enhance clarity, reproducibility, and overall impact, the following specific revisions are recommended. These suggestions address issues related to methodological transparency, data presentation, referencing, and consistency throughout the manuscript.

Main text

- Page 3: The authors state that the 400 nm laser pulse activated the nπ* transition. Given the high pump fluence, could azobenzene undergo multi-photon excitation to a higher-lying electronic state or even ionization? This possibility should be addressed or clarified in the manuscript.
- Page 3: The authors refer to Extended Data Fig. 2, but the purpose of this reference is unclear. Including a brief description of what the reader can observe in this figure would improve clarity and help contextualize its relevance.
- Page 3: The authors state that "the solute and cage contributions are negligible." They later report "clear time-dependent features reflecting contributions from solute structural dynamics" (page 4). This wording appears contradictory and must be explained.
- Page 7: The authors state that "the refined I1 and I2 structures exhibited substantially improved agreement with experiment." The authors should quantify this improvement.
- Page 7: The authors state that "The structural fitting alone cannot characterize the motion responsible for I2 → cis-azo and extract the overall sequence of structural transformations for the CASPT2-calculated structures." Could the authors add an explanation of why the motion responsible for I2 → cis-azo cannot be characterized from the structural fitting alone? The authors report a TRXL-refined cis-azo structure, but no information on the structural fitting of this structure is given. The authors should include this information.
- Page 8: The authors state that they "concluded that the trans-to-cis isomerization of azobenzene is best explained by a sequential mechanism: OHT for trans-azo → I1, followed by OHT and ROT for both I1 → I2 and I2 → cis-azo." This may be overstating their observation. The isomerization could be equally well—or better—explained by more complex motions. The authors should consider clarifying that these represent the most parsimonious motions identified in their analysis, rather than implying exclusivity.
- 400 nm excitation lies at the blue edge of the nπ* absorption band, potentially imparting additional thermal and kinetic energy to the solute in the excited state. They also discuss how thermal and kinetic energy may activate vibrational modes in their treatment of Debye–Waller factors in the Supplementary Methods. The authors should clarify whether this additional energy could enable a more complex set of motions during the reaction, and whether this would impact the relevance of the identified parsimonious motions in describing the data.

Figures

Fig. 2
- The relative signal contributions in Fig. 2 were "quantified by calculating the area under their respective curve". Was this then summed over all times or do the relative contributions refer to a specific time? How does the relative signal contribution evolve as a function of time? Is the solvent contribution dominating the signal by more than 90% at all times?
- The authors conducted a separate fs-TRXL measurement with neat methanol. The neat solvent was heated via two-photon absorption of the 400 nm pump pulse. Do the authors also observe direct heating of the solvent in the azobenzene measurements, and how would this influence the conclusions?

Fig. 3
- Is it correct that Fig. 3a shows the solute + cage contribution, while Fig. 3b is calculated from the solute contribution alone? Could this be clarified?
- Fig 3f displays smooth oscillations at low q values for the TRXL-refined data of SADS1 and the CASPT2-calucated data for SADS2. The authors should discuss what gives rise to these smooth oscillations and explain their significance.

Methods

Data collection
- The authors should add more details to this section. Was a timing tool used? Were any corrections applied to the recorded images? Were any images rejected prior to data processing and analysis and by which criteria?
- The authors should consider adding a table summarizing key experimental parameters. This would improve clarity and allow readers to quickly access essential details.

Data processing and analysis
- The section is repetitive, confusing, and lacks important details. The authors state that "The TRXL data were analyzed using established TRXL data analysis methods" but do not provide direct citations or specify which methods were used. I strongly encourage the authors to revise and expand this section, including figures that illustrate key data processing steps.

Obtaining solvent-contribution-free TRXL data
- The authors state that "For the PEPC treatment, we used heating signals and an artifact component obtained from a separate experiment with a neat solvent as known reference components, allowing for the removal of their contributions from the data." This process should be explained in more detail, and the components should be shown in a figure. What is the shape of the artifact component, and what does it arise from?
- The authors state that "To achieve a more accurate representation of the solvent-heating response of the azobenzene solution, the heating signal obtained from a separate experiment using a neat solvent was not used in its original form. Instead, it was corrected to account for potential differences in the shape of the difference scattering signal corresponding to

the hydrodynamic response of the azobenzene solution and the neat solvent. This correction ensures a more accurate representation of the solvent-heating effects specific to the azobenzene system. The correction procedure applied in this study is described in detailed in the previous work44,46,56-59." This statement requires more explanation. From a quick review of some cited references (e.g., references 44, 46, and 57), it is unclear which correction the authors are referring to. Could the authors clarify the nature of this correction or explicitly link it to the relevant references?

Structural parameters for structure refinement
- The authors state that "For trans-azo and cis-azo, the structures derived from quantum chemical calculations (CASPT2) were used without further refinement of structural parameters. In other words, these structures were not refined." The authors also mention minimal adjustment of cis-azo in the main text and present a TRXL-refined SADS3 in Fig. 3f and a cis-azo structure from TRXL in Extended Data Fig. 8. This appears inconsistent. Could the authors clarify the extent of structural refinement and reconcile these statements to avoid confusion?
- The authors state that "It should be noted that the agreement between the experimental data (SADS3) and the simulation is excellent (Fig. 3f), indicating both the sufficient accuracy of the CASPT2 structures of trans-azo and cis-azo, and the high quality of the TRXL data. In contrast, the agreement for SADS1 and SADS2 is less satisfactory". Please quantify these agreements.
- The authors should consider including a table that provides an overview of the different motion mechanisms, the degrees of freedom associated with each mechanism and combination of mechanisms, and the corresponding (reduced) chi-squared values. This would allow readers to easily compare models and assess the quality of fit.
- The authors mention that "The structures of the intermediates generated by applying these atomic motions were used to calculate the solute-only and cage scattering signals. The structural parameters used to generate the intermediate structures were optimized to ensure that the SADS′(q)s obtained from these structures accurately represent the SADS(q)s". Could the authors clarify whether they calculated a set of structures with varying parameters and compared these to the data, or whether the structural parameters were directly optimized during the chi-squared fitting process?

Ab-initio calculations
- The authors state that "An imaginary shift of 0.2 a.u. was introduced to prevent the inclusion of intruder states during the calculations." Could the authors provide more information on this and explain what is meant by an "imaginary shift"?

Extended Data Figures

Extended Data Fig. 1 and 2
- Panel e shows an anisotropy feature near 1.5 Å^{-1} that persists for over 10 ps. This feature is absent in the solvent-contribution-free TRXL data, suggesting it originates from an anisotropic solvent-related process. Could the authors clarify or speculate on the possible mechanism behind this feature?
- The color scale in Extended Data Fig. 1e appears inconsistent in comparison to Extended Data Fig. 2e. Can the authors verify that the color scales are accurate?

Extended Data Fig. 4
- The authors identified three significant components. Based on the reported measures (singular values and autocorrelations), could the authors provide a quantitative rationale for selecting three components rather than a different number?

Extended Data Fig. 5
- The authors should expand the caption. While the Supplementary Methods provide an interpretation of the SADS in real space, the caption should also include a brief discussion of the key features displayed in the figure to aid immediate understanding.

Extended Data Fig. 6
- The authors should either consider including an additional statistical measure or provide more detail on the reported chi-squared statistics. As currently presented, the chi-squared values cannot be meaningfully evaluated by the reader.

Supplementary Methods

Singular value decomposition (SVD) and kinetic analysis of isomerization kinetics
- Could the authors clarify what is meant by "the top three singular-value-weighted RSVs were globally fitted"? How was the global fit applied to the three components?

Obtaining species-associated difference scattering curves
- Could the authors include the explicit equation used to calculate the concentration of trans-azo forming I1 from the reported alpha value?

Chi-square as a fitting criterion for the structural analysis of SADSs
- Could the authors include a figure of the theoretical signals before and after PEPC treatment?
- The authors mention performing a baseline correction during chi-squared fitting because the SADS may contain residual

baseline features. Could the authors clarify what these residual features arise from? Since this procedure is applied to a novel dataset, it would also be helpful to provide more detail on the data analysis steps and visualize key parts of the process, rather than referring to studies involving strongly scattering heavy atoms.
- The authors determine the optimal alpha values by minimizing the chi-squared statistic. Could the authors clarify how alpha depends on the structural models? Do the alpha values vary significantly across different structures, or are they largely consistent?
- Was the noise present in the simulated SADS accounted for in the chi-square fitting? What is its impact on the structural refinement?

Error estimation for structural parameters
- Please clarify the meaning of a 1-unit increase in the chi-squared value in the context of the study.

Debye equation for simulating the scattering signal from solute structures
- The authors introduce Debye-Waller factors to account for broadening caused by vibrational motion in azobenzene. They consider Debye-Waller factors for the refinement of structures I1 and I2. Since cis-azo is populated during the photoreaction and presumably vibrationally hot, could the authors comment on whether the assumption that cis-azo is a structurally stable ground-state molecule holds at the investigated time scales

Molecular dynamics (MD) simulations for simulating cage terms
- The authors state that “The force field parameters and atomic charges for the solvent molecules were adopted from previous studies.” Could the authors provide more detail on these parameters or specify which previous studies were used as sources?
- The authors state that they used the refined molecular structures of the I1 and I2 intermediates for natural bond orbital analysis. Could the authors clarify what is meant by “refined molecular structures”? Does this refer to experimentally refined structures? If so, could the authors explain why experimentally refined structures were used in the simulations, which were presumably intended to enable the fitting of the experimental data?

Validating the kinetic model through linear combination fitting analysis
- The authors validate the kinetic model through linear combination fitting and conclude that it accurately describes the structural dynamics. Given that this validation relies on SADS derived from the same kinetic model, could the authors comment on whether it is expected that the model could not be recovered? How do they ensure that this does not introduce circular reasoning?

Supplementary Discussion

2 | Analysis of the anisotropic scattering signal
- The authors state that “I2 and cis-azo do not contribute detectable anisotropy signals. This, however, does not necessarily mean the absence of anisotropy in I2 and cis-azo. Rather, it may be that the substantial rotational motions accompanying the I1 → I2 transition (ROT + OHT) lead to rapid rotational dephasing, reducing their anisotropy below the detection limit, since anisotropy signals arise from the initial alignment of molecules before rotational motions randomize their orientation.” This point is somewhat unclear. How does the rapid rotational dephasing and reduction of anisotropy below the detection limit differ from the absence of anisotropy in I2 and cis-azo? Could the authors clarify the distinction between these two scenarios?

3 | Systematic Scan for Parsimonious Motions (SSPM) to characterize structural motions
- The authors refer to “conventional SADS analyses.” Could the authors consider adding a reference to a study where such a conventional SADS analysis was performed, to provide context for readers?
- The authors state that they “could identify the smallest set of motions that yielded a sufficiently low χ^2 value”. Could the authors define what threshold or range they consider sufficiently low, and explain how this choice was justified?
- Out of curiosity, based on the analysis presented, would it be possible to estimate the relative contributions of individual mechanisms to the overall motion in cases where two or more mechanisms are involved? Could the authors comment on whether such a decomposition is feasible within their current framework?

4 | Basis-Motion Analysis (BMA) to unveil the motions responsible for structural transitions and 5 | Discussion on the basis motions analyzed by BMA
- The authors use BMA to evaluate how well a given motion applied to structure A can reproduce structure B and to identify which motions drive the observed structural transitions. For the TRXL-refined geometries, which were optimized using these motions, could the authors comment on whether it is expected that the same motions appear prominently in the BMA analysis? How do they ensure that this observation is not simply a consequence of the refinement procedure?
- The authors report an RMSD of 0.244 Å for I1 to I2 for the CASPT2 results, but also that the largest RMSD among these steps is around 0.03 Å. The authors should verify that the reported values are correct.

6 | Comparison between CASPT2 and previously reported theoretical structures
- Could the authors include the resulting S_1 potential energy surface from the CASPT2 calculations? Visualizing this surface would help readers understand the energetic landscape.

9 | Comparison of the results from spectroscopy studies and TRXL results

- The authors state that “It is important to note that the assignments made by previous studies are not entirely incorrect; rather, the structural dynamics we observed suggest that the trans-to-cis isomerization of azobenzene involves contributions from both out-of-plane hula-twist and rotation.” Could the authors expand on this statement and elaborate on what aspects of the previous studies they consider correct and which aspects they believe require revision or clarification?

Supplementary Figure

Supplementary Fig. 1
- The caption does not agree with the reported results or the caption in Fig. 4.

General comments and questions

Several general issues and clarifications would improve the clarity and consistency of the manuscript:
- The authors should ensure that the axes on their figures are accurate, labels on the y-axis appear shifted in multiple figures.
- Cross-references between sections and to figures should be checked for correctness.
- The manuscript and Supplementary Information refer to PEPC-treated data and solvent-contribution-free data. Are these distinct or equivalent? This should be clarified.
- While the structural investigation of different motion mechanisms is intriguing, do the authors anticipate that future TRXL studies of organic molecules could track structural changes directly in the time-resolved data, rather than relying on systematic scans for parsimonious motions and basis-motion analysis to identify structural transitions?

References

The references are generally appropriate; however, in several sections of the Methods and Supplementary Information, the authors refer to “established” or “well-established” methods without providing adequate citations. In some cases, only one or two references are given, often self-citations. For methods described as established, multiple references from different research groups should be included to substantiate this claim. Additionally, the authors should provide direct citations to the relevant studies in Extended Data Tables 1 and 2 to facilitate access for readers interested in these sources.

Clarity and context

Clarity and context in the main text of the manuscript are satisfactory.

Referee #3

(Remarks to the Author)
I co-reviewed this manuscript with one of the reviewers who provided the listed reports.

Version 1:

Reviewer comments:

Referee #1

(Remarks to the Author)
I have sent these comments to the editorial office. Two of my students spent a considerable amount of time testing the code and checking the materials uploaded. The authors should upload the average raw diffraction data (one diffraction pattern per time delay + reference) and their complete workflow for reproducibility checks.

Additional comments from the students in my team:

Major Complaints

We are lacking raw experimental data and a significant part of the pipeline. In Methods of the paper they talk about transforming raw data into a specific cleaned state so that analysis can be done. The code provided only provides structural analysis, for SSPM and BMA, and nothing else. While the code as given runs without major issues, it is hard to judge properly without the full pipeline and raw experimental data.

For example, I would like to see the PEPC implementation, in particular. I know they discussed this in focus in a previous paper, but they did not release any code for it that time. Given that this is the first time they are using it for experimental data

and it is such a powerful and novel method, I would like to see how the code works. If they have it released it somewhere (I have looked for it), I believe it should be listed prominently given it is a significant part of the preprocessing.
Similarly for PCA, SVD, solvent cage work that was done with MD, etc. We are missing all code and experimental from before the techniques described in sections Data processing and Obtaining solvent-independent TRXL data.

Minor Complaints

The capsule release does not clearly identify which specific scripts, outputs, and shipped artifacts correspond to each manuscript-facing result. In particular, the package exposes multiple overlapping BMA/MoCA output paths and iteration-labeled structure/cage files without a concise public manifest linking them to the reported tables and figures.

From the released materials alone, I could not find the code corresponding to the profile-likelihood uncertainty procedure described in Supplementary Method 4. The only visible structural-uncertainty routine is a commented-out Hessian-based helper path, which is a local quadratic approximation rather than the parameter-fixing/re-optimization profile-likelihood procedure described in the supplement.

The released I1 to It SSPM script does not clearly separate the unconstrained I2 parameterization from the symmetry-reduced BIC accounting. As a result, the public code can present an I2 fit with independent structural parameters while still applying a symmetry-reduced parameter count, which makes the manuscript-facing I2 comparison harder to audit.

A bit nitpicky, but their label system as written in README.txt does not correspond to their code. They state that they have used iteration 5 for their results, but their provided data files for structural information are not iteration 5. We have an iteration 1, two iteration 4s for the intermediaries, etc. It's unclear if these files are not up-to-date or if the readme is wrong or miswritten.

Sincerely,
German Sciaini

(Remarks on code availability)
I provided comments above.

Referee #2

(Remarks to the Author)
The revised manuscript X-ray liquidography decodes complex motions in azobenzene isomerization by Kim et al. is greatly improved in terms of clarity, consistency, and overall presentation. My previous comments and suggestions have been addressed satisfactorily, and I now recommend acceptance for publication in Nature after the following minor issues have been addressed.

Requests for clarification
Supplementary Information, page 12 (baseline correction):
The authors state that the baseline correction parameters a_i and b_i were treated as free parameters and optimized simultaneously with the solute structural parameters. However, in reporting the number of free parameters, only the structural motion parameters and two additional parameters (mean-square displacement and scaling coefficient) are included. Could the authors clarify why the baseline correction parameters are not counted among the free parameters?
Methods section (lines 649–667) and Supplementary Method 13:
The authors note that some reduced chi-squared values are well below unity and attribute this to the effective number of independent information channels being smaller than the number of measured q-points. Would reducing the number of q-points (e.g., through binning along the q-axis) mitigate this issue?

Minor Revision Suggestions
Main Text
Lines 143–152: The authors may consider referring to a figure that visualizes the structural parameters of the four basic motion types (e.g., Fig. 4), to aid the reader.
Line 817 (Extended Data Fig. 1): Please verify the references to figure panels.
Lines 874–875 (Extended Data Fig. 6): Ensure consistency between color descriptions and the figure.
Lines 890, 893 (Extended Data Fig. 7): Ensure consistency between color descriptions and the figure.
Extended Data Table 1: Update the time constants and associated uncertainties in the “This work” row to reflect the revised values.
Supplementary Information
Page 39 (Supplementary Note 1): Clarify the reference to three versus four isomerization mechanisms.
Page 44 (Supplementary Note 4): The text mentions five dominant candidate motions, including OHT, which is not reported elsewhere in the revised manuscript; tables and figures indicate four motions. Please ensure consistency.
Page 56: Duplicate sentence: “If I1 had perfect C2h symmetry…”

(Remarks on code availability)
I was able to run the code on the Code Ocean platform. However, I did not install or execute the code locally, nor did I test it on datasets other than those provided with the manuscript. A README file is included with instructions for installation and

execution.

Referee #3

(Remarks to the Author)
I co-reviewed this manuscript with one of the reviewers who provided the listed reports.

(Remarks on code availability)

Version 2:

Reviewer comments:

Referee #1

(Remarks to the Author)
The authors have successfully addressed all major concerns, and the manuscript is nearly ready for publication, pending a few comments from my students that can be treated as minor (attached below).

We appreciate the authors accommodating all of our requests. In particular, the code is much better structured now and significantly easier to follow.

Best regards,
German

Comments from Student 1:

Things for the authors to check:

The manuscript reported +/- 4.7° uncertainty bars of the cis-AB ∠CCNN, which appears to be derived from the profile-likelihood calculation. The code looks at the region $\Delta\chi 2 \leq 1$, around each minimum to determine a two-sided error range. However, it apparently does not account for when the inter-basin barrier is below the $\Delta\chi 2 = 1$ metric. As a result, the code combines the error widths of two minima, with a barrier around $\Delta\chi 2 \approx 0.8$ above the local basin, at approximately -21.8° and the lower one at -54°. The right-hand error of 4.65° is consistent with the -21.8° minimum, but the left-hand error of 2.64° appears to be the result of the deeper minimum at -54°, thus hybridizing the error bars. The authors should correct this profile interval calculation and clarify which minimum and errors were intended.

Supplementary Method 1 states the auto-correlations should be greater than 0.8 for the first three RSVs and less than 0.5 for the fourth. However, recalculations from the deposited RSVs are 0.9875, 0.9042, 0.7100, and 0.6907, hence the third value is less than 0.8 and the fourth greater than 0.5. There may be some error in the reproduction methodology which found the auto-correlations using Hkifuncs.m using data from PCAstr.mat, though the values seem to match what is stored in pepcSVDStr.Viso.

While the capsule does provide the atomic coordinates of the intermediate structures for both I1 and I2, it does not provide XMS-CASPT2 input files, output logs, gradients, and convergence metrics. However, the Methods section provides details regarding the XMS-CASPT2 computation settings and the rigid CASPT2 PES scans are shown in Supplementary Figure 7. While ORCA inputs are present, these relate to fixed geometry charge calculations rather than XMS-CASPT2 geometry optimizations. The authors should provide the necessary records to fully reproduce the reported structures.

The Tikhonov regularized λ constant seems inconsistent, and is stated to be $\lambda = 7.5$ in Supplementary Method 12.4 and 12.7, but takes a value of approximately 4.74 in the code. The deposited penalty and RMSD are mutually consistent with $\lambda = 4.74$, so there appears to be a discrepancy between the code and output versus the penalty supplied in Supplementary information, which is consistent with $\lambda = 7.5$.

The current structural SSPM uses 511 q-points, but uses an Neff of 110 for the BIC penalty via a Chebyshev polynomial fit, consisting of three SADS and seven solvent LSVs. This is a reasonable method, but this value seems almost entirely dependent on the solvent singular vectors, which contribute over 99.999% to the pooled residual sum of squares at $K = 110$. The authors should justify the relative weighting used and demonstrate how the BIC changes with Neff.

Additional comments from Student 2:

- Pro: They have provided averaged data for the anisotropic, isotropic, and azimuthal components for the solvent and solution. This averaged data is not actually an input for the analysis. This is clarified in data/averaged/README.md, where instead the shot-pairing data that is obtained from processing the single-shot data is given, where they give one example.

- Pro: The code is much better organized, with files and folders more clearly labeled, as well as RE-ADMEs for every major section, a global one, and MANIFEST.md, and additional commenting. Very good job.

- Con: There is a mixing of the global vs. relative file paths, with more confusion brought from how Code Ocean handles data files. This does not affect running due to a helper script, and is therefore not a top priority, but should be clarified if possible.
o E.g. for 02_svd_kinetics we have local relative path.
 E.g. we have local file paths stated in 02_svd_kinetics as 02_svd_kinetics/data/Shot_pairring_241207/ and the global read-me as02_svd_kinetics/, Shot_pairring_241207.m (minor typo here, should be fixed).
o But for 05_sspm_bma, the data is not in the relative data folder but the global one.
 In 05_sspm_bma the file paths are ../data/sads_svd/PCAStr.mat and similar.
 This could be in part due to being accessed by different components instead of just one, but I'm not 100% sure because looking at the table indata/README.md in the final col-umn where it clarifies placement in manuscript and what component accesses it, I can-not see a pattern for each type of data storage.
o I have to ask the question: Why is data storage handled differently here? Is there an intentional design reason, or perhaps related to different people writing, writing certain components later than others, etc.
 There is room for disagreement here on whether it is worthwhile to change and whether it should be changed in the first place.
o This is made more confusing by how everything on Code Ocean is stored in data/...
 This does make sense if you understand Code Ocean/Git requirements, but in combina-tion with the inconsistency above it makes it very annoying to track data locations.
 It means that for about half the project components on Code Ocean, you need check the individual readme for the relative path and then the data/ readme to find its actual path when referencing data.
• (I assume the Code Ocean will be linked when the paper is published, so it will not just be me having to parse the code situation.)

- In my opinion, this point should be mentioned as a warning in the global readme (where most people would start), even just as a reference like "For essential clarification on data locations in local vs. Code Ocean, see data/README.md"), because it is easy to miss if you jump straight to looking at a module. It would fit well within the Locally section, but exact placement is their choice.
o Even without changing anything about the code, this addition to documentation would improve things immensely.

(Remarks on code availability)
Details were above provided

Open Access This Peer Review File is licensed under a Creative Commons Attribution 4.0 International License, which permits use, sharing, adaptation, distribution and reproduction in any medium or format, as long as you give appropriate credit to the original author(s) and the source, provide a link to the Creative Commons license, and indicate if changes were made.
In cases where reviewers are anonymous, credit should be given to 'Anonymous Referee' and the source.
The images or other third party material in this Peer Review File are included in the article’s Creative Commons license, unless indicated otherwise in a credit line to the material. If material is not included in the article’s Creative Commons license and your intended use is not permitted by statutory regulation or exceeds the permitted use, you will need to obtain permission directly from the copyright holder.

To view a copy of this license, visit https://creativecommons.org/licenses/by/4.0/

Responses to the Comments from Referee #1

The authors present experimental insights into the photoinduced trans-to-cis isomerization of azobenzene in methanol using time-resolved X-ray scattering. They employ a recently developed data analysis protocol—called projection to extract the perpendicular component (PEPC)—which enables the removal of solvent background, a significant challenge in experiments of this type. If the method developed by the authors indeed allows for resolving structural changes in such a weakly scattering molecule within a solvent environment, this would represent a noteworthy advancement, and I therefore support its publication in Nature.

→ We thank the referee for the encouraging summary and for supporting the publication of our work.

Moreover, while the authors refer specifically to femtosecond time-resolved X-ray liquidography (fs-TRXL), their analytical approach appears to be broadly applicable. In principle, it could also be adapted to femtosecond electron diffraction, with appropriate adjustments to sample concentration.

→ We appreciate the referee's insight regarding the broader applicability of our PEPC approach to other techniques such as femtosecond electron diffraction. Indeed, the underlying principle of orthogonal projection against solvent reference components is technique-independent and could be adapted to femtosecond electron diffraction or other scattering-based methods, provided that appropriate solvent reference data are available.

Should the data analysis protocol prove to be as effective as demonstrated, it would constitute a significant breakthrough. In that case, I strongly encourage the authors to make their raw data publicly available. This would allow the broader scientific community to engage with, test, and build upon their approach.

→ We completely agree with the referee on the importance of open science. To enable the broader scientific community to validate and build upon our approach, we have deposited the processed one-dimensional curves as source data for all relevant figures (main text Figures, Extended Data Figures, and Supplementary Figures) and the key analysis code on the Code Ocean platform. These deposited datasets and code are sufficient to independently reproduce and validate the data analysis presented in this work. Due to the extreme size of the complete set of raw 2D diffraction images (exceeding 12 terabytes), which is too large to be accommodated by publicly available repositories, these specific files will be made available upon reasonable request.

The sample consisted of a 50 mM solution of trans-azobenzene dissolved in methanol, which was flowed through a 100-μm capillary. This concentration appears relatively low compared to previous work, making the success of their PEPC approach and fs-TRXL experiments even more compelling.

→ We thank the referee for recognizing the technical difficulty of the experiment and the effectiveness of our approach in extracting reliable structural information from low signal-to-noise ratio data derived from low-concentration samples.

To achieve a sufficient signal-to-noise ratio for data analysis, the authors conducted 21 runs for the target sample (trans-azo) and 5 runs for the neat solvent. Data from these runs were averaged and used for subsequent analysis. I recommend that these averaged datasets be made publicly available through a suitable repository.

→ We have deposited the averaged datasets (for both the target sample and neat solvent) as source data for all relevant figures (main text Figures, Extended Data Figures, and Supplementary Figures), along with the relevant data analysis code and software, on Code Ocean. A corresponding Code and Software Checklist has also been submitted. Accordingly, we have updated the Data Availability Statement to reflect this commitment.

We note that the number of measurement runs reported in the original manuscript contained a typographical error: the correct numbers are 18 runs for the *trans*-azobenzene solution and 4 runs for the neat solvent. This has been corrected in the revised manuscript.

Abstract: Isomerization, which alters molecular geometry without changing Perhaps replace 'composition' by 'connectivity'…

→ We appreciate the suggestion, but we note that some isomerization processes such as molecular switching involving ring-closing involve changes in connectivity. In the revised manuscript, this sentence was removed to reduce the total word count and bring the manuscript within the journal's prescribed limit.

Check: … a combined inversion-assisted rotation motions …

→ We thank the referee for the careful reading. The sentence containing this typo has been removed in the revised manuscript as part of the substantial revision of the mechanistic discussion.

Page 3: … the solute, cage (term, remove), and solvent terms.

→ We have removed the redundant word "(term)" from the sentence on Page 3.

Page 5: For quantitative analysis, the first three RSVs were globally fitted with a sum of exponential functions sharing common time constants across all three RSVs. This yielded three exponential time constants: 0.40 ± 0.03 ps, 2.44 ± 0.02 ps and 13.8 ± 0.05 ps (Fig. 3c). The observation of three distinct time constants is consistent with a three-step sequential model widely adopted in previous studies14,15,17 regardless of the identities of the species involved. Guided by this framework, we assigned the three time constants (0.40 ± 0.03 ps, 2.44 ± 0.02 ps, and 13.8 ± 0.05 ps; Fig. 3c).

Avoid repetition so close.

→ We have rephrased the paragraph on Page 5 to remove the repetitive listing of the time constants. We note that the errors associated with the time constants have been updated following the revision of the sigma values. The resulting error estimates now fall within a more reasonable range. We have replaced the relevant paragraph with the following one:

"For quantitative analysis, the first three RSVs were globally fitted with a sum of exponential functions sharing common time constants across all three RSVs, yielding three distinct time constants: $\tau_1 = 0.4 \pm 0.2$ ps, $\tau_2 = 2.5 \pm 0.9$ ps and $\tau_3 = 14 \pm 3$ ps (Fig. 3c). The observation of three distinct time constants is consistent with a three-step sequential model widely adopted in previous studies[14,15,17], despite differences in the specific assignment of the intermediates (Supplementary Note 8). Guided by this framework (Fig. 3d), we assigned these time constants to the sequential transitions: the first intermediate (I_1) forms from the Franck–Condon (FC) state with τ_1, then converts to the second intermediate (I_2) with τ_2, and I_2 relaxes to *cis*-azo with τ_3."

Page 7: The refinement confirmed that the cis-azo structure required only minimal adjustment and remained nearly identical to the CASPT2-calculated structure (Extended Data Table 3), while the refined I1 and I2 structures exhibited substantially improved agreement with experiment (Fig. 3f). These results establish the TRXL-refined structures as reliable representations of the transient intermediates.

What makes TRXL-refined structures reliable? Please clarify.

→ We acknowledge that our original statement was ambiguous. By 'reliable,' we meant that the TRXL-refined structures yielded significantly reduced χ_v^2 values compared to the initial CASPT2 models, thereby providing a statistically superior description of the experimental data while maintaining physically reasonable geometries. In the revised manuscript, we now quantify this improvement using reduced chi-square (χv^2) values. We have revised the relevant paragraph as follows:

"Refinement showed that the *cis*-azo structure required relatively minimal adjustment, remaining highly similar to the CASPT2-calculated structure (Extended Data Table 3), with a small χ_v^2 reduction of 21%. In contrast, the refined I_1 and I_2 structures exhibited substantially improved agreement with the experimental data, yielding χ_v^2 reductions of 41% and 55%, respectively, relative to the initial CASPT2-calculated structures (Fig. 3f)."

We note that the fit quality has improved significantly compared to our previous manuscript, owing to the incorporation of a newly identified motion (C–N bond torsion, CNT) and the application of Bayesian Information Criterion (BIC) analysis for model selection. A detailed account of these developments is provided in our response to the corresponding comments by Referee #2.

Page 10: How is volume change defined in the context of volume-conserving reaction pathway?

→ In the context of the historical 'volume-conserving' debate for azobenzene, volume change refers to the expansion of the molecular hydrodynamic envelope (swept volume) required to reach the transition state. A 'volume-conserving' pathway implies that this swept volume is minimized, explaining why the reaction rate is insensitive to solvent viscosity. To quantify this, we estimated the solvent swept volume for each structural transition using a grid-based approach (Supplementary Note 11 and Supplementary Table 6), comparing an N–N fixed frame (where only the phenyl rings move) with a minimum-displacement frame (where all atoms rearrange concertedly). The minimum-displacement pathway reduces the swept volume by 81% for *trans*-azo → I_1, 43% for I_1 → I_2, and 4% for I_2 → *cis*-azo relative to the N–N fixed pathway, demonstrating the volume-conserving character of the observed isomerization pathway. We have revised and expanded the relevant paragraph in the manuscript to include this quantitative analysis as follows.

"The TRXL-refined intermediates shed light on this apparent contradiction. I_1, and I_2 are more compact than gas-phase CASPT2-calculated counterparts, as reflected in smaller R_g (Fig. 4a; Extended Data Table 3; Supplementary Note 9), consistent with isomerization within a solvent cage favoring geometries that displace less solvent. The compactness of individual intermediates alone, however, does not ensure a volume-conserving pathway: the solvent volume swept during transitions is critical. To quantify this, we compared two limiting scenarios for each transition: an N–N fixed frame where only the phenyl rings move, and a minimum-displacement frame where all atoms rearrange concertedly (Supplementary Note 12 and Supplementary Table 6). The minimum-displacement pathway sweeps only 20, 35, and 110 Å^3 for *trans*-azo → I_1, I_1 → I_2, and I_2 → *cis*-azo transitions, respectively, compared with 105 Å^3, 62 Å^3, and 114 Å^3 for the intuitive N–N-fixed pathway, representing reductions of 81%, 43%, and 4%. The reduction is largest for *trans*-azo → I_1, where the near-90° ∠CCNN change is achieved via bicycle-pedal-like concerted displacement of the azo bridge rather than phenyl-ring sweeps (Supplementary Videos 3 and 4). Even for I_1 → I_2, where ROT becomes active, the accompanying CNT and the optimally pre-configured ∠CCNN of I_1 ensure that the phenyl rings do not sweep through large volumes of solvent. Although isotropic TRXL data provide interatomic distances but not absolute molecular orientations, the observed solvent-viscosity independence favors the volume-conserving scenario (Fig. 4b) over the ring-sweep alternative (Supplementary Fig. 8). The TRXL-derived pathway thus reconciles the observed solvent-viscosity independence with the substantial ∠CNNC changes required to reach the *cis*-azo product. The isomerization achieves a 170° cumulative ∠CNNC rotation from *trans*-azo to *cis*-azo not by large-amplitude motion of any single group, but by coordinated small displacements of all atoms, including active participation of the nitrogen atoms, minimizing solvent-cage disruption."

Page 16: What's the absorptivity of trans-azobenzene at 400 nm?

→ The molar absorptivity of *trans*-azobenzene at 400 nm is approximately 292 $M^{-1}cm^{-1}$. We have included this value in the Experimental Methods section as follows:

"The molar absorptivity of *trans*-azobenzene at 400 nm was measured to be 292 $M^{-1}cm^{-1}$ under the experimental conditions."

Page 17: ... The neat methanol was heated via two-photon absorption of the 400 nm pump pulse, corresponding to excitation at 200 nm… What was the pump fluence in this experiment? I guess that there shouldn't be much two-photon absorption at 1.5 mJ/cm2.

→ The pump fluence used for the neat methanol experiment was not explicitly stated in the original manuscript (due to an oversight). The fluence used for this measurement was 5.3 mJ/mm^2, which is sufficiently high to induce two-photon absorption for solvent heating, unlike the much lower fluence (1.5 mJ/mm^2) used for the azobenzene sample. We have now specified this value in the Methods section (Data collection):

"To obtain reference data representing the response of bulk solvent, a separate fs-TRXL measurement was conducted with neat methanol at a pump fluence of 5.3 mJ/mm^2. The neat methanol was heated via two-photon absorption…"

Page 18: S_iso depends on t and therefore is not static.

→ We thank the referee for the careful reading. We previously used the term 'static' to distinguish the absolute scattering signal from the difference signal, but we acknowledge that this terminology is contradictory given the explicit time-dependence of $S_{iso}(q, t)$. The sentence containing this term has been removed as part of the comprehensive revision of the "Data processing and analysis" section, which has been restructured and renamed to "Data processing" in the revised manuscript. Throughout the revised manuscript, we have replaced 'static' with 'total' to avoid ambiguity.

I find that the excitation fluences used are quite high and only about one order of magnitude below the commonly cited universal threshold for laser ablation (~1 J/cm2); see, for example our work in Nat. Commun. 5, 3863 (2014), and references therein. I regret that I did not raise this point in my original review, as fluences are typically reported in units of mJ/cm2, which may have led to my oversight.

I would suggest working with fluences not exceeding 10 mJ/cm² and increasing the concentration accordingly to maintain a sufficient signal level.

→ We appreciate the referee's concern regarding the excitation fluence. We note that the commonly cited ablation threshold (~1 J/cm^2) pertains primarily to solid surfaces or static samples, whereas our experiment employs a continuously flowing liquid jet (~3 m/s), which ensures that each laser pulse interacts with a fresh sample volume, thereby substantially mitigating cumulative damage effects. To verify that our measurement operates in the linear excitation regime, we performed fluence-dependent femtosecond transient absorption (fs-TA) experiments and confirmed a linear relationship between signal amplitude and pump fluence up to 1.7 mJ/mm^2 (as described in our response to Referee #2's comment on multi-photon excitation). The fluence of 1.5 mJ/mm^2 used in this study was necessary to achieve a sufficient signal-to-noise ratio for azobenzene, a weakly scattering molecule without heavy atoms at a low concentration (50 mM). Reducing the fluence to 10 mJ/cm^2 (0.1 mJ/mm^2) would decrease the signal by more than an order of magnitude, making reliable extraction of the solute structural signal impractical under the current experimental conditions.

Responses to the Comments from Referee #2

Summary of the key results

In the study 'Complex motions in trans-to-cis isomerization of azobenzene decoded by femtosecond X-ray liquidography', Kim et al. report on the trans-to-cis photoisomerization process of azobenzene. Through pump-probe experiments with laser excitation at 400 nm to induce the nπ transition from S0 to S1 and structurally sensitive X-ray probe, the authors are able to resolve two intermediate states in the photoisomerization of trans-to-cis azobenzene. Trans-azobenzene excited to the Franck-Condon state relaxes to intermediate 1 (I1) with a time constant of around 0.4 ps, this is followed by a transition into a second intermediate (I2) with a time constant around 2.4 ps and final population of cis-azobenzene with a time constant around 13.7 ps.*

By testing sets of motions derived from classical mechanisms proposed for the photoisomerization of trans-to-cis azobenzene in literature, the authors refine theoretical CASPT2-calculated structures of I1 and I2 by chi-squared fitting to species-associated difference scattering (SADS) curves calculated from the experimental data. Based on a systematic scan for parsimonious motions and basis-motion analysis, the authors determine that the trans-to-cis isomerization of azobenzene can be explained by out-of-plane hula-twist (OHT) for trans-azo to I1 and OHT and rotation (ROT) for I1 to I2 and I2 to cis-azobenzene.

→ We thank the referee for the thorough summary of our work. We note that, in the revised manuscript, the out-of-plane hula-twist (OHT) motion has been replaced by C–N bond torsion (CNT), as OHT was found to be not independent of the other basis motions. Details are provided in our responses below.

The study introduces a novel application of time-resolved X-ray liquidography (TRXL) to a small organic molecule, providing direct structural insight into the trans-to-cis photoisomerization mechanism of azobenzene in methanol solution. The authors advance the understanding of this reaction by identifying two intermediates, refining their structure, and elucidating the parsimonious structural motions underlying the photoisomerization process.

If the authors can address the points raised below, this work demonstrates that time-resolved X-ray scattering can yield detailed structural information on photoreaction dynamics of small organic molecules in solution. It represents an important step toward establishing TRXL as a technique for such investigations and highlights the type of insights that can be obtained, potentially paving the way for future studies of photoactive organic molecules.

While the study is novel and potentially impactful, the authors should address the following comments, questions, and suggestions before the manuscript can be considered for publication in Nature.

→ We appreciate the referee's recognition of the novelty and potential impact of our study. We have carefully addressed all the comments to improve the manuscript.

Data & methodology

TRXL is an established technique for studying photoreactions, particularly in molecules containing heavy atoms. The authors convincingly demonstrate that the technique can be extended to molecules without heavy atoms. The data quality appears good, and the authors are able to separate the dominant solvent contribution from solute and cage contributions, showing that structural changes in azobenzene can be resolved. However, based on the current manuscript, it remains unclear whether the structure of intermediate states and the isomerization pathways can be successfully refined.

→ We are grateful for the referee's positive assessment of the data quality and the solvent–solute separation. Regarding the concern about whether the intermediate structures and isomerization pathways can be successfully refined, we have made substantial improvements to both the structural refinement procedure and the fit quality in the revised manuscript. These improvements confirm that the TRXL data indeed contain sufficient structural information to refine the intermediate geometries and to identify the underlying isomerization motions. As the referee has kindly recapped the suggested improvements in a separate section ("Suggested improvements"), we have provided our detailed point-by-point responses in that section.

The methods and analysis involve numerous separation and correction steps on both the experimental and simulation sides, making the workflow difficult to follow. In several sections, the manuscript does not sufficiently describe these processes. A figure summarizing the sequence of treatments and corrections applied to experimental and simulated data—both before and during analysis—would greatly improve clarity. This is particularly important given that the authors report a novel application of TRXL to a small organic molecule in solution; referencing procedures from previous studies on heavy-atom systems may not be sufficient.

→ We agree that a visual summary is essential for clarity. We have added a comprehensive flow chart (Supplementary Fig. 1) that details the entire data processing workflow, from raw images to the final structural refinement, explicitly showing each separation and correction step. We have also added a flow chart illustrating the data analysis pipeline (Supplementary Fig. 2) and another flow chart for structural and motional analysis (Supplementary Fig. 3). Our detailed response can be found in the "Suggested improvements" section.

The overall presentation quality is good, but some inconsistencies and contradictions were noted. The authors should carefully revise and proofread the Methods section and Supplementary Information.

→ We have thoroughly revised and proofread the Methods section and Supplementary Information to ensure consistency and remove any contradictions. Our detailed response can be found in the "Suggested improvements" section.

Appropriate use of statistics and treatment of uncertainties

The authors report an instrument response function of approximately 170 fs, which presumably limits the temporal resolution. However, they specify uncertainties of 20–50 fs, and a time constant of around 110 fs for the spatiotemporal analysis of the anisotropic fs-TRXL data for trans-azobenzene. Could the authors clarify how these values are consistent with the stated instrument response?

→ We thank the referee for raising this important point. In the revised manuscript, we have reanalyzed the anisotropic data and now identify the previously reported ~110 fs component as an IRF-limited instantaneous rise rather than a resolved kinetic time constant. This component is attributed to the impulsive rotational response of the solute molecules induced by the linearly polarised pump pulse. The two resolved time constants from the anisotropic analysis are 1.9 ± 0.5 ps and 22 ± 2 ps, both well above the IRF width. We have clarified this in Supplementary Note 2 as follows:

"The kinetics extracted from the first RSV reveal an IRF-limited instantaneous rise followed by two characteristic time constants: 1.9 ± 0.5 ps and 22 ± 2 ps (Extended Data Fig. 3f). The instantaneous rise is prominent in the anisotropic signal but weak in the isotropic signal. [...] The instantaneous component is consistent with an impulsive rotational response of the solute molecules induced by the linearly polarised pump pulse, in which the interaction between the pump electric field and the anisotropic molecular polarisability exerts a torque on the solute, analogous to the optical Kerr effect but acting on the solute rather than the bulk solvent. This process reorients the solute molecules on a timescale faster than the IRF, producing a strong anisotropic signal through the change in orientational distribution."

The authors employ chi-squared statistics using σ, the standard error of the mean for ΔS at time delay t. Please provide details on how σ was calculated.

→ The detailed procedure for calculating σ, the standard error of the mean of ΔS at each time delay and q-value, is now described in the revised "Data processing" section of the Methods. Briefly, the standard error of the mean was first calculated within each measurement run from the standard deviation of single-shot difference curves divided by $\sqrt{N_{pair}}$, where N_{pair} is the number of difference curves that survived the outlier filtering within that run, and then propagated across all 18 runs according to standard error propagation rules. Please see our detailed response to the "Data processing and analysis" comment below for the complete revised text.

While the use of chi-squared statistics may be appropriate, the reported chi-squared values appear quite large. The manuscript does not specify the number of data points, the number of fitting parameters in the structural refinement, or the degrees of freedom. Without this information, the reported chi-squared values and their differences appear arbitrary, making it difficult to assess the quality of the fits or the significance of differences between models. The authors should report these details and present chi-squared values in a way that allows more intuitive evaluation, for example reduced chi-squared. Including a table summarizing the fitting parameters for each model would be helpful.

→ We appreciate the referee's suggestion. In the revised manuscript, we now report the reduced chi-square (χ_ν^2) values, defined as $\chi_\nu^2 = \chi^2 / (n_q - d_f)$, where n_q is the number of q-points ($n_q = 511$ for the q-range of 1–7 Å^{-1}) and $d_f = k + 2$ is the number of free parameters (k structural motion parameters plus the mean-square displacement parameter σ^2 and the scaling coefficient), as detailed in Supplementary Method 3. The fitting parameters, the number of active motion parameters (k), and the resulting χ^2 and $\Delta\mathrm{BIC}(\chi^2)$ values for each candidate model are summarized in Supplementary Table 2, and the corresponding χ_ν^2 values are discussed in Supplementary Note 3. Our detailed response can be found in the "Suggested improvements" section.

The authors describe a systematic scan for parsimonious motions to identify minimal structural changes. However, it is unclear whether the statistical significance of different motions or combinations of motions was tested. For example, was an F-test or information criteria such as AIC or BIC applied?

→ We sincerely thank the referee for this valuable suggestion. Following the referee's recommendation, we employed the Bayesian Information Criterion (BIC) to objectively assess the statistical significance of including additional parameters (corresponding to more complex motions) in our models. The BIC results support our identification of the minimal set of motions required to describe each intermediate, confirming that the selected structural models provide a statistically significant improvement in fit quality without over-parameterization. Our detailed response can be found in the "Suggested improvements" section.

Conclusions: robustness, validity, reliability

Of particular importance is a detailed discussion of the experimental conditions, specifically the high pump power and excitation energy. The authors should clarify whether multi-photon excitation of azobenzene is a plausible scenario under these conditions.

→ We appreciate the referee raising this important point. To verify that multi-photon excitation does not occur under our experimental conditions, we performed fluence-dependent femtosecond transient absorption (fs-TA) measurements and confirmed a linear relationship between signal amplitude and pump fluence up to 1.7 mJ/mm^2, which encompasses the fluence of 1.5 mJ/mm^2 used in this study. This confirms that the excitation

process is dominated by single-photon absorption. Our detailed response can be found in the "Suggested improvements" section.

Additionally, the authors should clarify whether the methanol solvent was directly heated during the azobenzene measurements and, if so, discuss how this might influence the observed dynamics and the relative contributions of solvent, solute, and cage signals.

→ At the wavelength (400 nm) and fluence (1.5 mJ/mm^2) used in the azobenzene experiments, direct absorption and heating of methanol is negligible. The observed solvent thermal response in the azobenzene solution arises solely from heat dissipation by the photoexcited azobenzene molecules, not from direct excitation of the solvent. In contrast, the separate neat methanol experiment was performed at a higher fluence (5.3 mJ/mm^2) to generate a strong bulk solvent response, which served as the reference signal for the PEPC treatment. Importantly, the PEPC method projects the data into a space orthogonal to the solvent response, yielding solvent-independent signals for analysis. Therefore, although direct two-photon heating of the solvent is negligible at 1.5 mJ/mm^2, even if such a contribution were present, it would not affect the solute dynamics extracted via the PEPC analysis. Our detailed response can be found in the "Suggested improvements" section.

Furthermore, the manuscript should consider how vibrational modes activated through thermal and kinetic energy in the excited state affect the interpretation and relevance of the identified parsimonious motions.

→ We account for vibrational broadening effects via the Debye-Waller factors when calculating simulated difference scattering curves. The σ^2 (mean-square displacement) parameters in the Debye-Waller factor describe the width of the interatomic distance distribution arising from thermal motions, while the parsimonious motions describe the evolution of the mean molecular geometry along the reaction coordinate. These two contributions are complementary: the refined structures represent the average geometry at each time point, while the σ^2 parameters capture the vibrational ensemble around that average. Therefore, the vibrational effects do not invalidate the relevance of the identified motions; rather, they are separately quantified as the broadening of the structural ensemble. To clarify that our structural analysis accounts for the potential involvement of excited-state vibrational modes and the resulting distribution broadening, and to elucidate how incorporating these effects via the Debye-Waller factor ensures the robustness of our motion assignment, we have expanded the discussion in Supplementary Method 6 titled "Debye equation for simulating the scattering signal from solute structures" as follows:

"By explicitly incorporating the DWF, our analysis effectively decouples the vibrational effects from the structural changes. In this framework, the refined structural parameters define the average molecular geometry, whereas the σ^2 parameters capture the width of the vibrational ensemble around that center. Therefore, the presence of vibrational broadening does not invalidate the identified parsimonious motions; rather, by explicitly accounting for it through the σ^2 parameter, the framework ensures that the refined structural parameters isolate the mean molecular geometry from the vibrational envelope surrounding it."

Indeed, the refined σ^2 values for I_1, I_2, and *cis*-azo (0.018, 0.010, and 0.000 Å², respectively) are small, indicating that the structural ensemble remains well-defined at each stage of the reaction and that the excess vibrational energy is effectively dissipated to the solvent. Our detailed response regarding the relevance of the identified parsimonious motions can be found in the "Suggested improvements" section.

Finally, the main article must clearly state that these motions represent parsimonious motions and that other motions might be possible.

→ We have revised the main text to explicitly acknowledge that the identified motions represent the most parsimonious set and that more complex trajectories cannot be ruled out. Specifically, we now state in the main text:

“Our primary conclusion regarding the dominant reaction coordinate, involving the C–N torsion and N–N rotation, is robustly supported by our systematic evaluation of alternative structural models, although the involvement of more complex structural coordinates unexplored in this work cannot be ruled out.”
Our detailed response can be found in the “Suggested improvements” section.

Suggested improvements

Main text

Page 3: The authors state that the 400 nm laser pulse activated the nπ transition. Given the high pump fluence, could azobenzene undergo multi-photon excitation to a higher-lying electronic state or even ionization? This possibility should be addressed or clarified in the manuscript.*

→ To ensure that the photoisomerization is initiated by a single-photon process and to rule out the potential contributions of multi-photon excitation or ionization, we performed femtosecond transient absorption (fs-TA) experiments with varying pump fluences. Specifically, we monitored the signal amplitude at 2 ps as a function of pump fluence up to 1.7 mJ/mm^2. The fluence-dependence plot (Fig. R1) shows a clear linear relationship between the signal amplitude and pump fluence, providing compelling evidence that the reaction dynamics are governed by single-photon excitation. We have added an explicit clarification in the manuscript as follows.

“To confirm that the photoisomerization is initiated by a single-photon process at our experimental fluence (1.5 mJ/mm^2), we examined the pump-fluence dependence of the signal using femtosecond transient absorption (fs-TA) experiments varying pump fluences up to 1.7 mJ/mm^2. The signal amplitude at 2 ps exhibited a linear relationship with pump fluence (not shown). This linearity confirms that multi-photon excitation or ionization processes are negligible under our experimental conditions.”

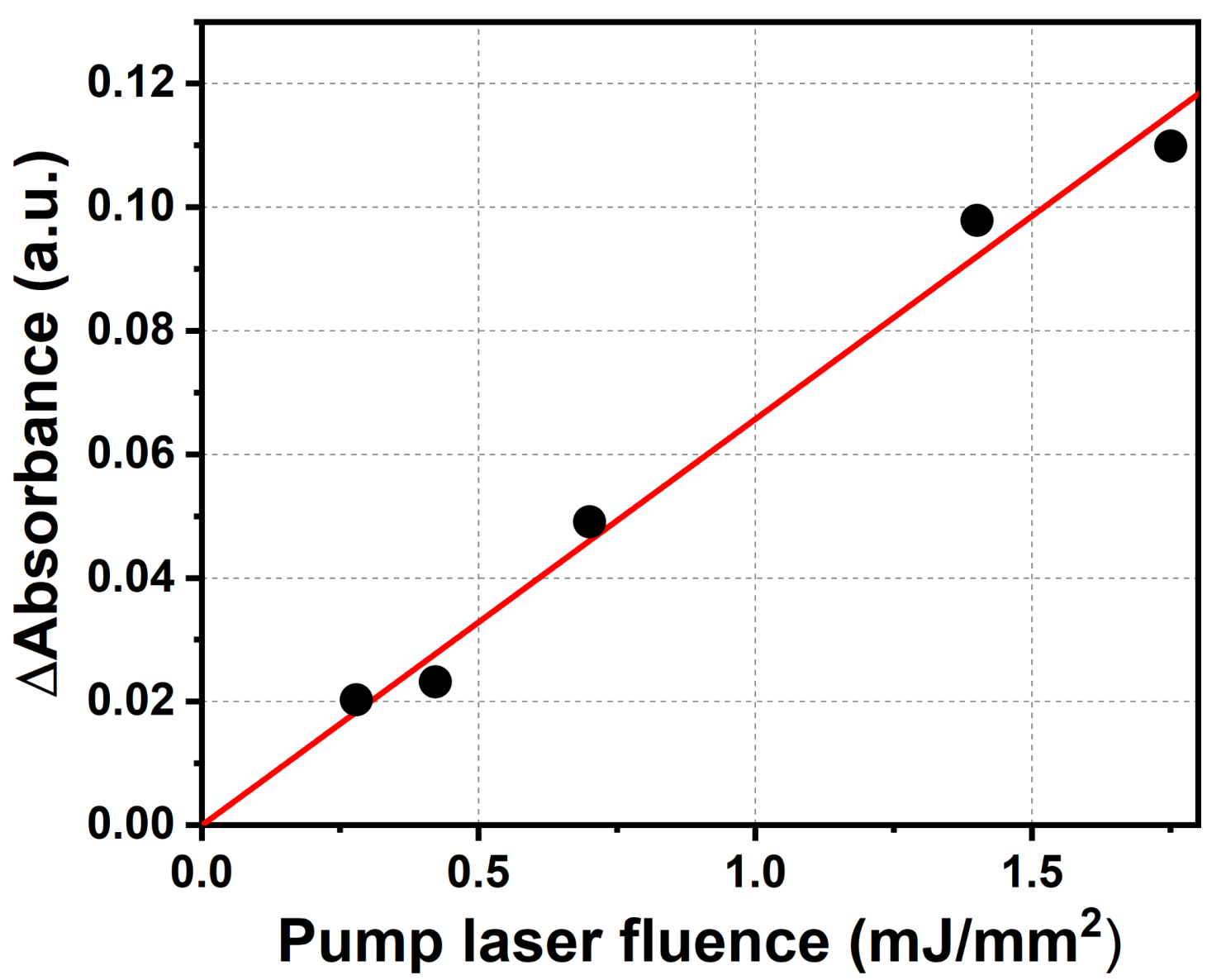

Fig. R1: Fluence dependence of the transient absorption signals of *trans*-azobenzene in methanol measured at a time delay of 2 ps and a probe wavelength of 540 nm. It demonstrates a linear dependence of the signal on pump fluence, confirming that multi-photon excitation or ionization processes are negligible under our experimental conditions.

Page 3: The authors refer to Extended Data Fig. 2, but the purpose of this reference is unclear. Including a brief description of what the reader can observe in this figure would improve clarity and help contextualize its relevance.

→ We appreciate the suggestion to improve clarity. Extended Data Fig. 2 plays a crucial role in validating our method by demonstrating that the PEPC analysis successfully isolates solute signals from the solvent background. Specifically, it shows that while the azobenzene solution exhibits clear time-dependent structural features, the neat solvent data are reduced to featureless noise after PEPC treatment. To make this clear to the reader, we have revised the text to explicitly describe the figure's content immediately upon citation as follows.
"The resulting solvent-independent curves multiplied by q, $q\Delta S^{\perp}_{iso}(q, t)$ and $q\Delta S^{\perp}_{aniso}(q, t)$, are shown in Extended Data Fig. 2. This comparison demonstrates the efficacy of the PEPC method: while the azobenzene data retains clear time-dependent structural features, the corresponding neat solvent data are effectively reduced to featureless noise, confirming the successful removal of the solvent background. Fig. 2b shows the decomposition of $q\Delta S^{\perp}_{iso}(q, t)$ into the solute, cage, and solvent terms."

Page 3: The authors state that "the solute and cage contributions are negligible." They later report "clear time-dependent features reflecting contributions from solute structural dynamics" (page 4). This wording appears contradictory and must be explained.
→ The statement on Page 3 ("negligible solute contribution") describes the raw difference scattering data ($q\Delta S_{iso}(q, t)^{raw}$), where the solute signal is overshadowed by the dominant solvent heating signal. In contrast, the statement on Page 4 ("clear time-dependent features") describes the PEPC-treated data ($q\Delta S^{\perp}_{iso}(q, t)$), which reveals the solute structural dynamics after the solvent background has been removed. To prevent ambiguity, we have revised the text on Page 3 as follows.
"The $q\Delta S_{iso}(q, t)^{raw}$ is nearly identical to the solvent term, whereas the solute and cage contributions are virtually negligible relative to the dominant solvent term."

We have also refined the corresponding sentence on Page 4 as follows.
"In contrast, $\Delta S^{\perp}_{iso}(q, t)$ from the azobenzene solution exhibits clear time-dependent features revealed by extracting the signal component independent of the solvent response, reflecting contributions from solute structural dynamics (azobenzene) despite their much smaller amplitude relative to solvent heating signals in the raw data. "

Page 7: The authors state that "the refined I1 and I2 structures exhibited substantially improved agreement with experiment." The authors should quantify this improvement.
→ We thank the referee for the suggestion. In the revised manuscript, to quantitatively demonstrate the improvement, we have calculated the percentage reduction in the reduced chi-square (χ_v^2) values, which accounts for the degrees of freedom. The refinement resulted in a significant improvement, with χ_v^2 decreasing by 41% for I_1 and 55% for I_2 compared to the initial CASPT2 models. We have revised the text to include these quantitative metrics. A more detailed discussion can be found in our response to one of the subsequent comments.
"Refinement showed that the *cis*-azo structure required relatively minimal adjustment, remaining highly similar to the CASPT2-calculated structure (Extended Data Table 3), with a small χ_v^2 reduction of 21%. In contrast, the refined I_1 and I_2 structures exhibited substantially improved agreement with the experimental data, yielding χ_v^2 reductions of 41% and 55%, respectively, relative to the initial CASPT2-calculated structures (Fig. 3f)."

Page 7: The authors state that "The structural fitting alone cannot characterize the motion responsible for I2 → cis-azo and extract the overall sequence of structural transformations for the CASPT2-calculated structures." Could the authors add an explanation of why the motion responsible for I2 → cis-azo cannot be

characterized from the structural fitting alone? The authors report a TRXL-refined cis-azo structure, but no information on the structural fitting of this structure is given. The authors should include this information.

→ We thank the referee for this comment. The reason why the structural fitting characterizes the motion for the intermediates but not for the final product lies in the fundamentally different refinement strategies employed. Although it is in principle possible to refine the *cis*-azo structure by applying structural motions starting from the I_2 intermediate (thereby identifying the I_2 → *cis*-azo motion), we chose to refine the structure of the final product directly to independently validate its structure, leveraging the availability of the calculated *cis*-azo geometry as a reliable starting point. Since the *cis*-azo is a stable ground-state isomer, its calculated structure is inherently more reliable than those of transient excited-state intermediates. Thus, refining its structure directly against the experimental data provides an independent structural determination, without assuming a specific connecting motion from I_2. In contrast, for I_1 and I_2, which are transient species with unknown geometries, we refined the structures by generating candidate geometries through the application of specific structural motions to their precursor structures. Specifically, the I_1 structure was determined by starting from the CASPT2-optimized *trans*-azo structure and optimizing the structural parameters associated with the candidate motions to minimize the deviation from the experimental $SADS_1$. Similarly, for I_2, the structural search was performed starting from both the *trans*-azo and the refined I_1 structures (as detailed in Extended Data Fig. 6). This process simultaneously optimized the geometry and characterized the motion connecting them. Consequently, the fitting procedure for *cis*-azo yields the optimized structure but does not inherently characterize the dynamic motion driving the transition from I_2, which is why the subsequent Basis-Motion Analysis (BMA), described in Supplementary Method 14, was necessary.

We apologize for the confusion, which stems from the fact that the detailed description of this *cis*-azo fitting procedure was inadvertently omitted in the original manuscript. We have added the following explanation in the main text. We have also added a new subsection (Supplementary Method 12.4) detailing the fitting procedure for *cis*-azo (detailed in one of the subsequent responses).

"For *cis*-azo, SSPM was not used. Instead, starting from the CASPT2-calculated *cis*-azo structure, we optimized its structural parameters to best fit $SADS_3$. The refinement strategy for *cis*-azo differed from that for the intermediates: it focused on determining the optimal structure of the final product, rather than characterizing the structural motion from I_2."

The detailed *cis*-azo fitting procedure is described in the newly added Supplementary Method 12.4, and our detailed response is provided in one of the subsequent comments below.

Page 8: The authors state that they "concluded that the trans-to-cis isomerization of azobenzene is best explained by a sequential mechanism: OHT for trans-azo → I1, followed by OHT and ROT for both I1 → I2 and I2 → cis-azo." This may be overstating their observation. The isomerization could be equally well—or better—explained by more complex motions. The authors should consider clarifying that these represent the most parsimonious motions identified in their analysis, rather than implying exclusivity.

→ We appreciate the referee for this important comment. The referee's suggestions to explore the possibility of additional motions, to employ the Bayesian Information Criterion (BIC) for assessing the statistical justification of the assigned motions, and to quantify the motional contributions proved highly valuable in guiding our revision. In addition, we improved the quality of the cage term by running MD simulations with a larger simulation box and longer simulation times. Aided by these developments, the fit qualities for I_1 and I_2 improved significantly compared to our previous manuscript, although the resulting structures did not deviate significantly from the previous ones. Specifically, we re-evaluated the statistical justification of the previous assignment (OHT alone) using BIC. The BIC analysis showed that the I_1 structure is statistically better described by the combination of ROT + OHT rather than OHT alone. More importantly, the resulting structure retained essentially the same CNNC dihedral (179.9° vs. 180.0°) and CNN angles (114.2° vs. 113.4°) as

trans-azo, with the only significant change occurring in the CCNN dihedral angles (0° to 72.6°). This indicated that the ROT + OHT combination effectively performed C–N bond torsions rather than the intended out-of-plane deformation of the CNNC framework, and the I_1 structure obtained from ROT + OHT could be equivalently generated by a single C–N bond torsion motion, which we term CNT. Previously, we did not include CNT because it has received little attention in previous studies and is not directly related to the mechanisms previously proposed (rotation, inversion, hula twist, or inversion-assisted rotation).

At this point, we realized that the OHT motion we employed was not entirely independent from the other motions. While ROT, INV, NND, and CNT are independent and can each be represented by a single structural parameter (for example, ROT by ∠CNNC and CNT by ∠CCNN), OHT involves changes across nearly all structural parameters associated with the other motions. Since this inter-dependence is not ideal for SSPM, we removed OHT from the motional pool. In fact, a combination of CNT, ROT, INV, and NND can produce an OHT motion when considering the internal degrees of freedom. With this new motional pool, the I_2 structure generated from the new I_1 also showed improved fit quality, although the resulting I_2 structure did not deviate significantly from the previous one. Once CNT was added to the motional pool, BIC analysis showed that the $I_1 \rightarrow I_2$ transition is best explained by ROT + CNT. In addition, we developed a method termed Motional Contribution Analysis (MoCA) to quantify the relative contributions of individual motions. A detailed description of MoCA is provided in our response to the referee's relevant comment. MoCA analysis of the $I_1 \rightarrow I_2$ transition revealed that ROT and CNT contribute 70% and 30%, respectively. We note that we have dropped the in-plane vs. out-of-plane distinction for hula twist in the revised manuscript, as the literature does not consistently separate them.

Additionally, we acknowledge that while the proposed mechanism (CNT → CNT + ROT) is the best description within the framework of these fundamental motions, it does not strictly exclude the possibility of more complex structural dynamics. Therefore, we have revised the manuscript to clarify that our proposed mechanism represents the most parsimonious explanation consistent with the experimental data, rather than claiming exclusivity.

"While the involvement of more complex structural coordinates, especially involving those not extensively explored here such as CCCN improper angles, CCN angles, and C–N distances, cannot be ruled out, the determined motional assignment provides a sufficient and statistically robust description of the experimental observations, within the framework of the minimal set of structural motions necessary to describe the data."

Additionally, we have added a new paragraph at the end of the "Structures of I_1 and I_2" section to address the inherent limitations of the current data quality while emphasizing the robustness of our mechanistic conclusions:

"Experimental uncertainties arising from the finite signal-to-noise ratio may influence the extent of motions determining the numerical values of the refined structural parameters, such as bond angles and dihedral angles of I_1 and I_2. While future higher-quality data might lead to quantitative adjustments, they are unlikely to alter the fundamental mechanistic assignment. Our primary conclusion regarding the dominant reaction coordinate, involving the C–N torsion and N–N rotation, is robustly supported by our systematic evaluation of alternative structural models, although the involvement of more complex structural coordinates unexplored in this work cannot be ruled out."

400 nm excitation lies at the blue edge of the nπ absorption band, potentially imparting additional thermal and kinetic energy to the solute in the excited state. They also discuss how thermal and kinetic energy may activate vibrational modes in their treatment of Debye–Waller factors in the Supplementary Methods. The authors should clarify whether this additional energy could enable a more complex set of motions during the reaction, and whether this would impact the relevance of the identified parsimonious motions in describing the data.*

→ We acknowledge that 400 nm excitation imparts substantial excess vibrational energy to the excited state. Considering that the nπ* band origin of *trans*-azo is located at ~520 nm (2.38 eV), whereas excitation at 400 nm corresponds to 3.10 eV, resulting in an excess vibrational energy of ~0.72 eV in the initially prepared excited state. However, our data provides compelling evidence suggesting that this energy is effectively dissipated in the solution phase and does not significantly distort the reaction coordinate into a complex set of motions. Specifically, if significant excess energy induced large-amplitude, randomized fluctuations traversing complex potential energy landscapes, we would expect a broad structural distribution for the resulting intermediates. However, the refined mean-square displacement (σ^2) values for I_1, I_2 and *cis*-azo are remarkably small (σ^2 = 0.018 ± 0.003, 0.010 ± 0.003, and 0.000 ± 0.006 Å^2, respectively). These quantitative values indicate that the structural ensemble is well-defined and chemically "cool," suggesting that the excess energy is efficiently dissipated to the solvent rather than driving randomized complex motions. Thus, the directed reaction coordinate (CNT for *trans*-azo → I_1 and ROT + CNT for I_1 → I_2) remains dominant. We have incorporated this reasoning into the manuscript as follows:

"...Furthermore, while the 400-nm excitation imparts excess vibrational energy, the small refined mean-square displacement (σ^2) values for I_1, I_2 and *cis*-azo (σ^2 = 0.018 ± 0.003, 0.010 ± 0.003, and 0.000 ± 0.006 Å^2, respectively) provide strong evidence that the structural ensemble remains well-defined. This suggests that the excess energy is effectively dissipated to the solvent rather than inducing large-amplitude randomized motions, thereby preserving the dominant reaction coordinate."

Figures

Fig. 2

: The relative signal contributions... were "quantified by calculating the area under their respective curve". Was this then summed over all times or do the relative contributions refer to a specific time? How does the relative signal contribution evolve as a function of time? Is the solvent contribution dominating the signal by more than 90% at all times?

→ The relative signal contributions were quantified by first integrating the absolute signal magnitudes over q at each time delay, then summing these values across all measured time delays. Specifically, for each time delay, we calculated the area under the curve in q-space using trapezoidal integration. These instantaneous values were then summed over all measured time delays to determine the total signal contribution of each component. This analysis reveals that the solute contribution is most prominent at very early time delays, before vibrational energy has fully dissipated into the solvent. Crucially, however, the solvent background remains so dominant that it accounts for at least 85% of the total signal intensity even at these earliest time delays. As energy dissipation proceeds and hydrodynamic heating grows, this dominance becomes even more absolute. This overwhelming dominance of the solvent background highlights the critical necessity of our PEPC method to extract the subtle solute dynamics.

The authors conducted a separate fs-TRXL measurement with neat methanol. The neat solvent was heated via two-photon absorption of the 400 nm pump pulse. Do the authors also observe direct heating of the solvent in the azobenzene measurements, and how would this influence the conclusions?

→ In the neat solvent experiment, we intentionally used a significantly higher pump fluence (5.3 mJ/mm^2) to induce two-photon absorption and generate a strong solvent signal for reference. However, in the azobenzene sample measurements, the pump fluence was kept sufficiently low (1.5 mJ/mm^2) to minimize multiphoton effects, and methanol is transparent to single-photon absorption at 400 nm. Consequently, direct solvent heating is negligible, and the observed solvent signal predominantly arises from vibrational energy transfer from the solute. Critically, the physical nature of the solvent response (hydrodynamic heating) determines the shape of its scattering signal in q-space. This spectral shape is identical regardless of whether the heat

originates from direct multiphoton absorption or vibrational energy transfer from the solute; only the signal amplitude scales with the amount of deposited energy. Therefore, the solvent components derived from the neat solvent data correctly capture the solvent signature in the azobenzene data. Since PEPC removes these components based on their shape in q-space, it effectively isolates the solute dynamics irrespective of the heating source, ensuring our conclusions remain unaffected.

Fig. 3

Is it correct that Fig. 3a shows the solute + cage contribution, while Fig. 3b is calculated from the solute contribution alone? Could this be clarified?

→ We have clarified in the figure legend that Fig 3a includes cage contributions, while Fig 3b represents the solute-only component. The revised figure caption is as follows.

"**a,b,** Solvent-independent experimental and simulated TRXL data, displayed as $q\Delta S^{\perp}(q)$ (reciprocal space) and $r^2\Delta S(r)$ (real space), respectively. **a,** Data displayed as $q\Delta S^{\perp}(q)$ in reciprocal space, including both solute and cage contributions. **b,** Data displayed as $r^2\Delta S(r)$ in real space, showing the solute-only component."

Fig 3f displays smooth oscillations at low q values for the TRXL-refined data of SADS1 and the CASPT2-calucated data for SADS2. The authors should discuss what gives rise to these smooth oscillations and explain their significance.

→ These low-q oscillations are a mathematical consequence of the PEPC procedure and do not carry specific physical meaning related to solute structural changes. Solvent heating contributions typically manifest as strong signals at low-q due to long-range structural changes associated with bulk solvent thermal expansion. The PEPC method extracts the signal component orthogonal to these solvent components. When such a dominant low-q component is removed via a projection, it can leave characteristic oscillatory features in the low-q region. However, these features are not structural signatures of the solute but rather a mathematical consequence of the projection process. We have added the following clarification at the end of the "Obtaining solvent-independent TRXL data" section in Methods:

"We note that the oscillations observed in the low-q region of some SADS curves are a mathematical consequence of the PEPC procedure, which projects the data into a space orthogonal to the solvent response, and therefore should not be interpreted on their own as direct structural fingerprints of the solute."

Methods

Data collection

The authors should add more details to this section. Was a timing tool used? Were any corrections applied to the recorded images? Were any images rejected prior to data processing and analysis and by which criteria?

→ We have added the following clarification to the revised "Data processing" section:

"No dedicated timing tool was employed in this experiment."

Details regarding image corrections, outlier rejection criteria, and the complete data processing workflow can be found in the comprehensive revision of the "Data processing" section described below.

The authors should consider adding a table summarizing key experimental parameters.

→ We have added Supplementary Table 1 summarizing the key experimental parameters for clarity. This table includes X-ray and pump laser specifications, sample conditions, detector configuration, and data acquisition statistics.

Supplementary Table 1 | Summary of key experimental parameters for fs-TRXL measurements

Parameter	Value
**X-ray (probe)**	
**Source**	PAL-XFEL
**Photon energy**	15.01 ± 0.03 keV (λ = 0.83 Å)
**Spot size**	20 μm × 20 μm (FWHM)
**Photon flux**	1×10^{12} (photons / pulse)
**Repetition rate**	60 Hz
**Optical laser (pump)**	
**Source**	fs Ti:sapphire laser (fundamental: 800 nm)
**Wavelength**	400 nm
**Spot size**	55 μm × 66 μm (H × V)
**Pulse duration**	~100 fs (FWHM)
**Laser fluence**	1.5 mJ/mm^2 (*trans*-azobenzene); 5.3 mJ/mm^2 (neat methanol)
**Repetition rate**	30 Hz
**Timing**	
**Time delays**	−0.35 ps to ~98 ps (137 time points)
**Instrument response function**	170 ± 49 fs (FWHM)
**Sample and detection**	
**Concentration**	50 mM (*trans*-azobenzene in methanol)
**Delivery system**	100 μm inner diameter capillary nozzle
**Vertical flow rate**	~3 m/s
**Detector**	Rayonix MX-225HS (6×6 binning mode)
**Available *q*-range**	1.0 $Å^{-1}$ to 7.0 $Å^{-1}$
**Number of scattering images per time delay**	18 runs × 250 images/run = 4,500 (*trans*-azobenzene); 4 runs × 250 images/run = 1,000 (neat methanol)
**Data retention ratio after outlier rejection**	95–99%

Data processing and analysis

The section is repetitive, confusing, and lacks important details. The authors state that "The TRXL data were analyzed using established TRXL data analysis methods" but do not provide direct citations or specify which methods were used. I strongly encourage the authors to revise and expand this section, including figures that illustrate key data processing steps.

→ We have comprehensively revised this section to address all concerns. The section title has been changed from "Data processing and analysis" to "Data processing" to more accurately reflect its scope, with analysis methods described separately in subsequent sections. We have expanded the sections to include:

1. Explicit method descriptions: All image correction procedures (polarization and solid angle corrections), AUC-based pairing, and outlier rejection criteria are now described in detail with specific citations to references.

2. Reorganized workflow: We eliminated repetition by restructuring the section into a clear, step-by-step workflow from raw images to final processed signals.

Additionally, to address the request for visual illustration of key processing and analysis steps, we have added three new figures (Supplementary Figs. 1–3) as an independent visual aid.

Supplementary Fig. 1 shows our data reduction pipeline, which can be summarized as follows:

- The complete sequence of processing steps (background correction → polarization/solid angle corrections → azimuthal integration/anisotropic decomposition → normalization → AUC-based pairing → differencing → outlier rejection → averaging → scaling)
- Decision points (e.g., azimuthal vs. anisotropic decomposition pathways)
- Key formulas and parameters at each step
- Data format transformations (2D images → 1D curves → difference signals)

Supplementary Fig. 2 shows our data analysis pipeline, which can be summarized as follows:

- The mathematical decomposition of isotropic data using SVD and kinetic modeling to isolate SADSs
- The iterative structural fitting, which minimizes the difference between experimental and theoretical SADSs incorporating both solute terms and solvent cage terms

Supplementary Fig. 3 shows our structural analysis pipeline, which can be summarized as follows:

- The rigorous statistical evaluation and refinement of intermediate structures utilizing SSPM and BIC
- The detailed decomposition and assignment of motions through BMA and MoCA

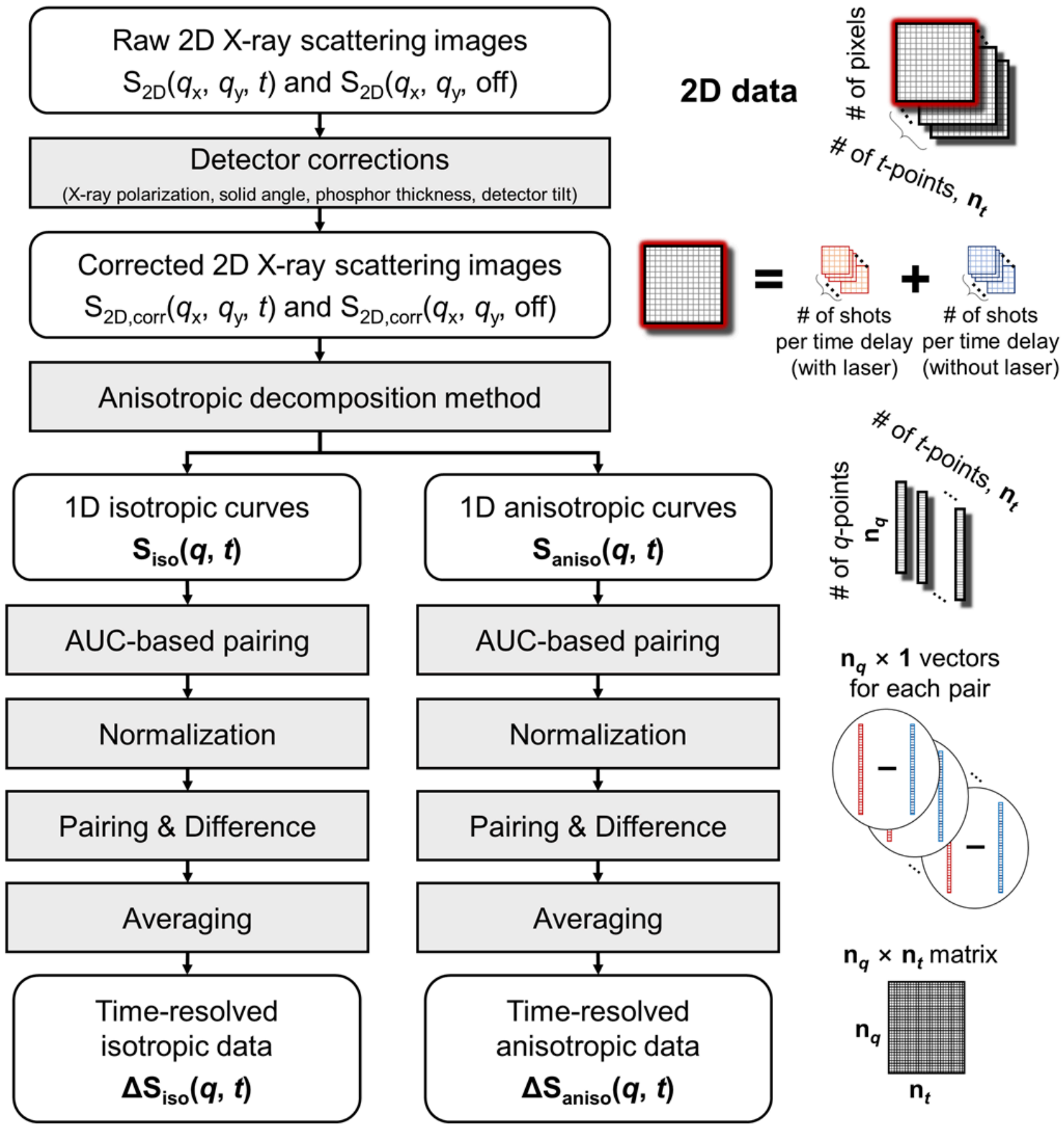

Supplementary Fig. 1 | Data reduction pipeline for time-resolved X-ray liquidography. The workflow illustrates the processing steps starting from the collection of raw 2D X-ray scattering images. These images undergo standard corrections (including polarization and solid angle corrections) to produce corrected 2D images, which are then decomposed into 1D isotropic and anisotropic curves using Legendre polynomial-based decomposition. The curves corresponding to "laser-on" and "laser-off" shots are matched based on an area-under-the-curve (AUC) criterion, and each curve is subsequently normalized individually. The difference curves calculated from these normalized pairs are then statistically averaged to yield the final time-resolved isotropic ($\Delta S_{iso}(q, t)$) and anisotropic ($\Delta S_{aniso}(q, t)$) difference scattering signals.

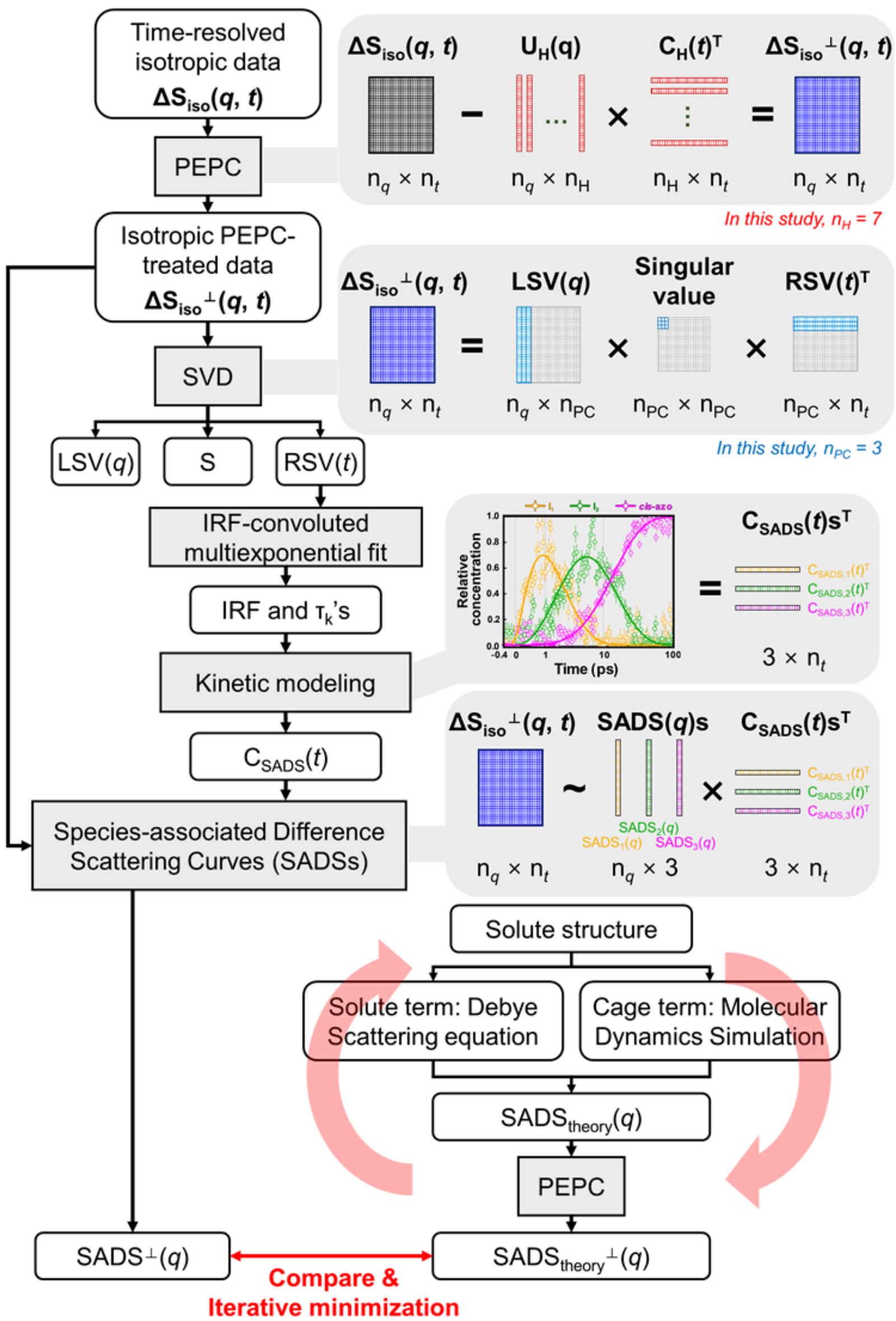

Supplementary Fig. 2 | Data analysis pipeline for time-resolved X-ray liquidography. The data framework encompasses both kinetic analysis and structural refinement. The difference TRXL data are processed using the projection to extract the perpendicular components (PEPC) method. Here, the number of solvent-related principal components (n_H) was independently determined from the TRXL data of neat methanol, and the corresponding left singular vectors (U_H) were employed for the PEPC. The PEPC-treated isotropic data are then analyzed via singular value decomposition (SVD) to identify the number of significant signal components (n_{PC}). In this study, we confirmed that $n_H = 7$ and $n_{PC} = 3$ best describe the TRXL data. Subsequently, a kinetic model is applied to extract species-associated difference scattering curves (SADSs) and their corresponding concentration profiles. Structural refinement is performed by combining a solute term derived from the Debye scattering equation and a cage term derived from molecular dynamics (MD) simulations. The final structural parameters are obtained through an iterative minimization process by comparing experimental SADSs with simulated SADSs until convergence is achieved.

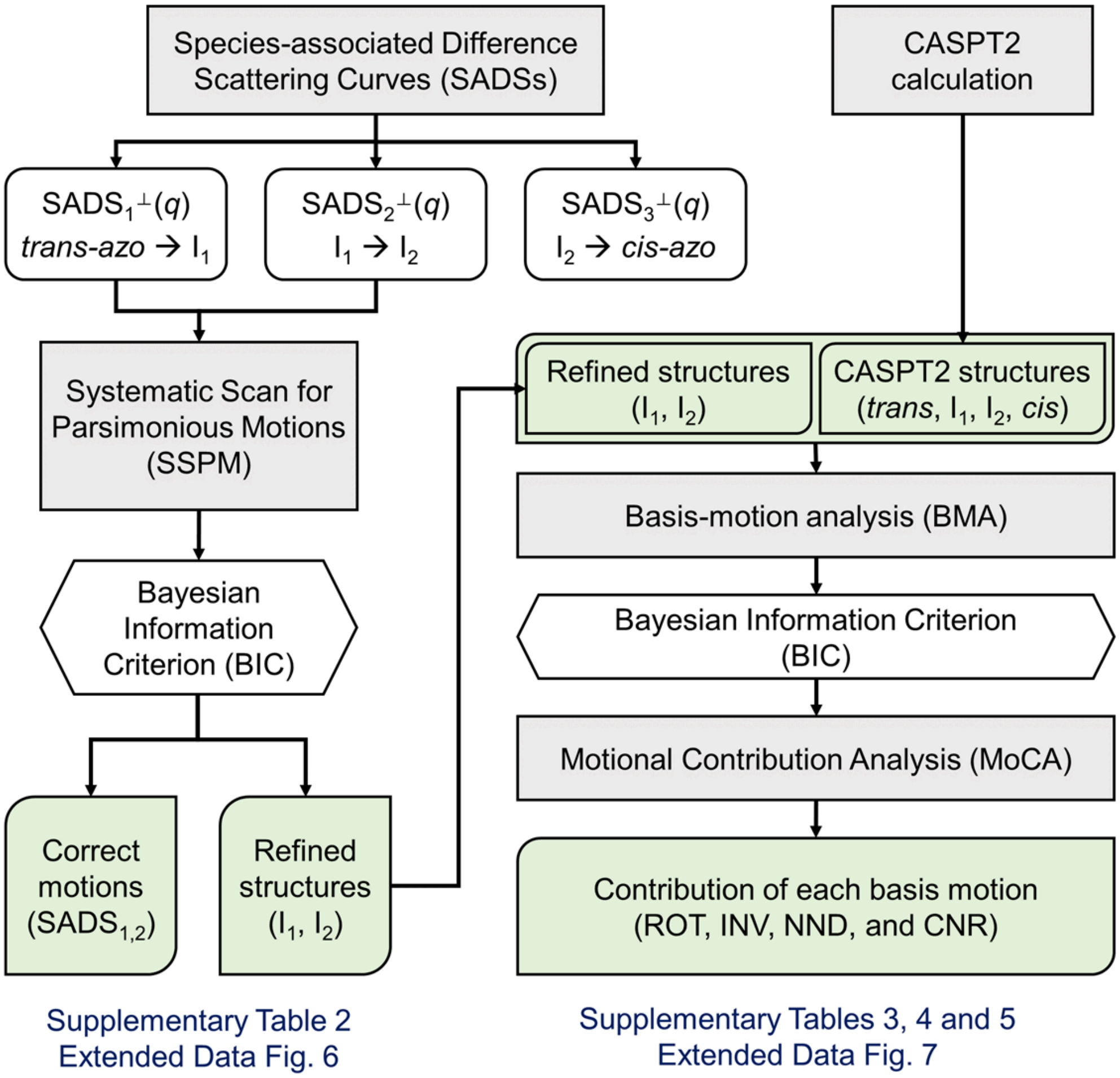

Supplementary Fig. 3 | A schematic for structural analysis, including SSPM, BIC, BMA, and MoCA. Workflow for extracting parsimonious structural motions from SADSs and quantifying their motional contributions. Three SADSs are assigned to sequential steps along the photoisomerization pathway (*trans*-azo to I_1, I_1 to I_2, and I_2 to *cis*-azo). A systematic scan for parsimonious motions (SSPM), evaluated using the Bayesian information criterion (BIC), identifies the minimal set of structural motions consistent with $SADS_1$ and $SADS_2$ and yields refined structures for I_1 and I_2. In parallel, reference geometries (*trans*-azo, I_1, I_2, *cis*-azo) from CASPT2 are analyzed together with the refined structures using basis-motion analysis (BMA). BIC-guided model selection and motional contribution analysis (MoCA) then quantify the relative contributions of the basis motions (ROT, INV, NND, and CNT) to each structural transition. The SSPM results are summarized in Supplementary Table 2 and Extended Data Fig. 6, and the related BMA and MoCA results are reported in Supplementary Tables 3–5 and Extended Data Fig. 7.

The revised "Data processing" section and newly added Supplementary Figs. 1 and 2 together provide a self-contained, non-repetitive description of our complete processing pipeline. The revised section is as follows.

"**Data processing**

Supplementary Fig. 1 details the entire data processing workflow, from raw images to the final structural refinement, explicitly showing each separation and correction step. The spatial overlap between the pump and probe pulses was established by adjusting the optical laser pointing to maximize the transient signal

amplitude. The temporal overlap (time-zero) was determined using a YAG crystal, where the X-ray-induced change in optical transmittance was monitored to identify the coincidence of the two pulses. No dedicated timing tool was employed in this experiment. Prior to all subsequent processing steps, each recorded diffraction image was corrected by subtracting a dark image (background) acquired under X-ray beam-off conditions, followed by corrections for polarization and solid angle effects. The polarization correction accounts for the linearly polarized nature of XFEL X-rays, which causes azimuthally anisotropic scattering intensity not related to sample anisotropy. Specifically, the measured intensity ($I_{measured}$) was corrected by the polarization factor, defined as $I_{polar} = I_{measured} \times 1/[1 - \sin^2(2\theta)\cos^2(\varphi)]$, where φ is the azimuthal angle relative to the X-ray polarization direction. The solid angle correction compensates for the reduced solid angle subtended by detector pixels at higher scattering angles in a flat detector geometry. This compensation requires an intensity correction with the formula: $I_{corrected} = I_{polar} \times 1 / \cos^3(2\theta)$. These corrections were applied using standard formulas implemented in the pyFAI package[ref].

Each corrected two-dimensional (2D) scattering image was then processed to obtain one-dimensional scattering curves as a function of q, the magnitude of the momentum transfer vector ($q = 4\pi\sin\theta / \lambda$, where λ is the X-ray wavelength and 2θ is the scattering angle). For each single-shot image, we obtained either (1) azimuthal scattering curves, $S_{azi}(q, t)^{single}$, through azimuthal averaging using the "integrate1D" function from pyFAI, or (2) separated isotropic and anisotropic scattering curves, $S_{iso}(q, t)^{single}$ and $S_{aniso}(q, t)^{single}$, via the anisotropic decomposition method[refs] using the "integrate2D" function from pyFAI. The isotropic component contains information on changes in interatomic distances independent of molecular orientation, while the anisotropic component reflects orientational dynamics of the molecules. The detailed procedure for the anisotropic decomposition method is provided in the literature[ref].

Before calculating difference scattering curves, each scattering curve was normalized by its area under the curve (AUC). Specifically, $S_{azi}(q, t)^{single}$ and $S_{iso}(q, t)^{single}$ were normalized by dividing by the AUC within the normalization range of q = 4–7 Å^{-1}, while $S_{aniso}(q, t)^{single}$ was normalized by the AUC of the corresponding $S_{iso}(q, t)$.

For each laser-on (pumped) measurement, we then calculated difference scattering curves by subtracting an appropriately paired laser-off (unpumped) signal. Rather than using simple time-sequential pairing, we employed an AUC-based pairing method to minimize systematic errors. Although normalization is applied to account for shot-to-shot fluctuations in beam intensity and sample thickness, detector nonlinearity (i.e., imperfect linearity between incident photon count and recorded signal intensity) can introduce systematic artifacts when subtracting laser-on and laser-off images with significantly different signal amplitudes. To suppress these artifacts, each laser-on curve was subtracted by its matched laser-off curve, the one with the most similar AUC value, thereby minimizing the influence of detector response nonlinearity on the resulting difference signal. This procedure yielded single-shot difference scattering signals, $\Delta S_{[x]}(q, t)^{single}$ ([x] = azi, iso, or aniso), for each time delay t.

To improve signal-to-noise ratio, multiple single-shot difference curves measured at the same time delay were averaged. Before averaging, an outlier rejection procedure was applied to ensure data quality. For each single-shot measurement, we calculated the AUC of the absolute azimuthal difference signal ($\int |\Delta S_{azi}(q, t)^{single}|\, dq$) over the q-range of 1.0–6.5 Å^{-1}. Single-shot curves with AUC values exceeding an empirically determined threshold were identified as outliers and excluded from subsequent averaging. This procedure typically retained 95–99% of the collected data, removing only extreme outliers arising from beam instability or sample flow irregularities. The data collection consisted of 18 independent measurement runs, with each run at each time delay comprising 250 laser-on images and 250 laser-off images. The outlier-filtered difference curves were first averaged within each run to obtain the run-averaged $\Delta S_{[x]}(q, t)^{run}$ and its associated standard error of the mean, $\sigma_{[x]}(q, t)^{run}$. The standard error of the mean was calculated as:

$$\sigma_{[x]}(q, t)^{run} = \sigma_{[x]}(q, t)^{single} / \sqrt{N_{pair}} \quad (1)$$

where $\sigma_{[x]}(q, t)^{single}$ is the standard deviation of the single-shot difference curves within that run for the specific time delay and q-value, and N_{pair} is the number of difference scattering curves within that run that survived the outlier filtering. A total of 18 independent measurement runs were performed. Prior to merging, each run-averaged curve was scaled by a relative weighting factor to ensure comparable signal amplitudes across runs; the corresponding $\sigma_{[x]}(q, t)^{run}$ values were scaled by the same factor. The scaled run-averaged curves were then averaged across all 18 runs to produce the final difference scattering curves, denoted as $\Delta S_{iso}(q, t)^{raw}$, $\Delta S_{aniso}(q, t)^{raw}$, and $\Delta S_{azi}(q, t)^{raw}$. The final standard error of the mean $\sigma_{[x]}(q, t)^{raw}$ was obtained by propagating the $\sigma_{[x]}(q, t)^{run}$ values according to standard error propagation rules. The $\Delta S_{[x]}(q, t)^{raw}$ data for *trans*-azo are shown in Extended Data Fig. 1a,c,e. Throughout this paper, difference curves are presented as $q\Delta S(q, t)$ for visualization in the figures, to emphasize high-q features associated with solute structural changes. This q-weighting was applied only for display and not used in quantitative analysis.

For quantitative analysis, a scaling procedure was implemented to normalize the signal amplitude to that of a single solvent molecule, following standard methods in the TRXL community[refs m7]. The experimental scattering intensities were calibrated to absolute units of electron units (e.u.) per solvent molecule using the following procedure. First, the total scattering curve of the solvent was calculated from the radial distribution function $g(r)$ obtained from molecular dynamics (MD) simulations, which yields the scattering intensity in electron units. This calculated curve was then normalized by the number of solvent molecules in the MD simulation box to yield the intensity in units of e.u. per solvent molecule. A scaling factor was determined by comparing the normalized experimental total scattering curve with this simulated curve in units of e.u. per solvent molecule. The use of the simulated scattering curve of the pure solvent to calibrate the experimental solution data is justified because the experimental total scattering intensity of the solution arises almost exclusively from the bulk solvent, given the low concentration of the solute (50 mM). Finally, this scaling factor was applied to all experimental difference scattering curves, $\Delta S_{[x]}(q, t)^{raw}$, allowing the experimental signals to be expressed in e.u. per solvent molecule. This quantitative normalization process enabled the extraction of precise information regarding the reaction dynamics, including the concentration of solute molecules participating in the isomerization pathways."

Obtaining solvent-contribution-free TRXL data

The authors state that "For the PEPC treatment, we used heating signals and an artifact component obtained from a separate experiment with a neat solvent as known reference components, allowing for the removal of their contributions from the data." This process should be explained in more detail, and the components should be shown in a figure. What is the shape of the artifact component, and what does it arise from?

→ We have added Supplementary Figs. 4 and 5, which display all reference solvent components used in the PEPC procedure. Supplementary Fig. 4 shows the isotropic reference components, and Supplementary Fig. 5 shows the anisotropic reference components. The solvent reference components were obtained from a separate fs-TRXL measurement of neat methanol subjected to two-photon absorption heating at a high pump fluence (5.3 mJ/mm^2). Both the $\Delta S_{iso}(q, t)^{raw}$ and $\Delta S_{aniso}(q, t)^{raw}$ from this neat solvent dataset were independently subjected to SVD analysis. As shown in Supplementary Figs. 4 and 5 (highlighted in yellow), seven major components from $\Delta S_{iso}(q, t)^{raw}$ were selected for the isotropic data and four components for the anisotropic data. The reference components were selected by individually inspecting each left and right singular vector (LSV and RSV) pair against two criteria: (i) autocorrelation coefficient of the RSVs ≥ 0.5, ensuring temporal consistency, and (ii) singular values significantly above the noise plateau (visible in Supplementary Figs. 4 and 5, panel c), ensuring the components represent genuine signal variations rather than noise. Through this component-by-component evaluation, we identified components 1–6 and 9 for $\Delta S_{iso}(q, t)^{raw}$ and components 1–4 for $\Delta S_{aniso}(q, t)^{raw}$. LSV_4 for $\Delta S_{iso}(q, t)^{raw}$ was included despite a lower overall autocorrelation because it exhibits a dominant contribution near time zero, capturing the initial ultrafast

response. These selected components serve as the reference basis for the PEPC procedure. It is important to note that these reference components are mathematical basis vectors that span the solvent response space, rather than individual signals with a strictly one-to-one correspondence to distinct physical phenomena. While Component 1 (LSV_1) for $\Delta S_{iso}(q, t)^{raw}$ is dominated by the thermal response ($(\partial S/\partial T)_\rho$) of the solvent, and LSV_1 for $\Delta S_{aniso}(q, t)^{raw}$ is dominated by the scattering signal arising from the optical Kerr effect of the bulk solvent, these and all other components represent mixed contributions from multiple physical origins (e.g., thermal expansion, optical Kerr effect, beam fluctuations, detector nonlinearity). Each component is simply a mathematical singular vector from the SVD analysis. By projecting the azobenzene solution data onto the subspace orthogonal to these basis vectors (separately for $\Delta S_{iso}(q, t)^{raw}$ and $\Delta S_{aniso}(q, t)^{raw}$), PEPC effectively isolates the signal components that are strictly independent of the solvent response, thereby retaining only the components of the solute structural signal that are orthogonal to the solvent response.

The original Methods section did not include a detailed description of the procedure for extracting solvent reference components. We have now added the following description in the revised manuscript:

"Both $\Delta S_{iso}(q, t)^{raw}$ and $\Delta S_{aniso}(q, t)^{raw}$ from the neat solvent dataset were independently subjected to singular value decomposition (SVD). The reference components were selected by individually inspecting each left and right singular vector (LSV and RSV) pair against two criteria: (i) autocorrelation coefficient of the RSVs ≥ 0.5, ensuring temporal consistency, and (ii) singular values significantly above the noise plateau, ensuring the components represent genuine signal variations rather than noise. Through this evaluation, seven LSVs (LSVs 1–6 and 9) were selected for $\Delta S_{iso}(q, t)^{raw}$, and four LSVs (LSVs 1–4) were selected for $\Delta S_{aniso}(q, t)^{raw}$ (Supplementary Figs. 4 and 5). LSV_4 for $\Delta S_{iso}(q, t)^{raw}$ was included despite a lower overall autocorrelation because it exhibits a dominant contribution near time zero, capturing the initial ultrafast response. By separately projecting the $\Delta S_{iso}(q, t)^{raw}$ and $\Delta S_{aniso}(q, t)^{raw}$ of the azobenzene solution data onto the subspaces orthogonal to their respective reference components, PEPC effectively isolates the signal components strictly independent of the solvent response, thereby retaining only the signal components associated with solute structural changes that are orthogonal to the solvent response."

The authors state that "To achieve a more accurate representation of the solvent-heating response of the azobenzene solution, the heating signal obtained from a separate experiment using a neat solvent was not used in its original form. Instead, it was corrected to account for potential differences in the shape of the difference scattering signal corresponding to the hydrodynamic response of the azobenzene solution and the neat solvent. This correction ensures a more accurate representation of the solvent-heating effects specific to the azobenzene system. The correction procedure applied in this study is described in detailed in the previous work44,46,56-59." This statement requires more explanation. From a quick review of some cited references (e.g., references 44, 46, and 57), it is unclear which correction the authors are referring to. Could the authors clarify the nature of this correction or explicitly link it to the relevant references?

→ We greatly appreciate the referee for bringing this to our attention. The description in the original manuscript regarding a "shape correction" of the heating signal was an error (a legacy description from a previous draft) and does not reflect the actual analysis procedure used in this study. In our final analysis, we did not apply any additional phenomenological shape correction to the solvent reference components. We found that the LSVs, derived directly from the SVD of the neat solvent data acquired under two-photon heating conditions, constitute a basis set that sufficiently spans the scattering signals corresponding to the solvent response in the azobenzene solution. Therefore, these LSVs were used directly as the reference components for the PEPC procedure without further modification.

We have removed the incorrect statement from the revised manuscript. The text has been updated to accurately reflect that the reference components obtained from the neat solvent SVD were used directly as the orthonormal basis set for the PEPC projection. The relevant paragraph has been revised as follows.

"The solvent contributions were effectively screened out from the original TRXL data (Extended Data Fig. 1) to obtain the PEPC-treated data presented in Extended Data Fig. 2, which we refer to as the solvent-independent data. The term "solvent-independent data" is equivalent to the term "solvent-contribution-free data" used in a previous study[64]; both describe TRXL data that has been projected into a subspace orthogonal to the solvent response. To obtain the reference components representing the solvent response for this PEPC procedure, we performed a separate fs-TRXL measurement of neat methanol under two-photon heating conditions. The left singular vectors (LSVs) extracted directly from the singular value decomposition (SVD) of this neat solvent data were used as the reference components without further modification. These components sufficiently capture the signal variations corresponding to the solvent response in the azobenzene solution, enabling effective removal of solvent contributions through orthogonal projection. Both $\Delta S_{iso}(q, t)^{raw}$ and $\Delta S_{aniso}(q, t)^{raw}$ from the neat solvent dataset were independently subjected to singular value decomposition (SVD). The reference components were selected by individually inspecting each left and right singular vector (LSV and RSV) pair against two criteria: (i) autocorrelation coefficient of the RSVs ≥ 0.5, ensuring temporal consistency, and (ii) singular values significantly above the noise plateau, ensuring the components represent genuine signal variations rather than noise. Through this evaluation, seven LSVs (LSVs 1–6 and 9) were selected for $\Delta S_{iso}(q, t)^{raw}$, and four LSVs (LSVs 1–4) were selected for $\Delta S_{aniso}(q, t)^{raw}$ (Supplementary Figs. 3 and 4). LSV_4 for $\Delta S_{iso}(q, t)^{raw}$ was included despite a lower overall autocorrelation because it exhibits a dominant contribution near time zero, capturing the initial ultrafast response. By separately projecting the $\Delta S_{iso}(q, t)^{raw}$ and $\Delta S_{aniso}(q, t)^{raw}$ of the azobenzene solution data onto the subspaces orthogonal to their respective reference components, PEPC effectively isolates the signal components strictly independent of the solvent response, thereby retaining only the signal components associated with solute structural changes that are orthogonal to the solvent response. The resulting solvent-independent data ($\Delta S^{\perp}(q, t)$) are presented in Extended Data Fig. 2. During this PEPC procedure, the standard errors of the PEPC-treated data, $\sigma^{\perp}(q, t)$, were calculated by propagating the standard error of the mean of the original TRXL data ($\sigma(q, t)^{raw}$) and the uncertainties associated with the reference solvent components. Specifically, since PEPC is a linear projection operation, the covariance matrix of the projected data was computed using standard error propagation rules to derive $\sigma^{\perp}(q, t)$, ensuring that the uncertainties in both the raw experimental data and the solvent basis set were rigorously accounted for."

Structural parameters for structure refinement

The authors state that "For trans-azo and cis-azo, the structures derived from quantum chemical calculations (CASPT2) were used without further refinement of structural parameters. In other words, these structures were not refined." The authors also mention minimal adjustment of cis-azo in the main text and present a TRXL-refined SADS3 in Fig. 3f and a cis-azo structure from TRXL in Extended Data Fig. 8. This appears inconsistent. Could the authors clarify the extent of structural refinement and reconcile these statements to avoid confusion?

→ We thank the referee for identifying the inconsistency in our description of the *cis*-azo refinement and for suggesting the quantification of the agreement. First, regarding the inconsistency: We acknowledge that the original description in the Methods section was inaccurate regarding the refinement procedure for *cis*-azo. While the CASPT2-calculated structure for *trans*-azo was used directly without refinement, the *cis*-azo structure underwent a further refinement against the TRXL data. Specifically, we used the CASPT2-calculated *cis*-azo structure as the starting geometry and allowed variations in the ∠CNNC and ∠CCNN angles during the fitting process. These two angles were selected because they exhibit the largest changes between the CASPT2-calculated *trans*-azo and *cis*-azo structures, whereas other structural parameters (∠CNN inversion angles, *r*NN, *r*CN, ∠CCCN improper angles, and ∠CCN angles) show only small changes (Extended Data Table 3). This refinement resulted in only minor adjustments to the initial CASPT2-calculated structure, which

confirmed that the structure deviates only minimally from the CASPT2-calculated structure. We have added new subsections (Supplementary Method 12.4 and Supplementary Method 12.7) providing the following details.

"**12.4 | Structural refinement of *cis*-azo (SADS3)**

The structure of *cis*-azo was determined by direct refinement against the experimental $SADS_3$, rather than by applying motions from the I_2 intermediate via SSPM (as done for I_1 and I_2 in Sections 12.5 and 12.6). This approach was adopted because: (i) *Cis*-azo is a ground-state (S_0) product for which CASPT2 provides a reliable reference geometry, unlike the excited-state intermediates I_1 and I_2 where multi-reference character complicates the theoretical prediction. (ii) The large conformational difference between I_2 (∠CNNC ≈ 120°) and *cis*-azo (∠CNNC ≈ 10°) makes the I_2 → *cis*-azo fitting landscape contain multiple local minima. An SSPM starting from I_2 would require extensive regularization and the result would be difficult to validate independently. (iii) The experimental $SADS_3$ directly encodes the *cis*-azo – *trans*-azo difference signal. Starting from the well-characterised CASPT2 *cis*-azo geometry and refining a minimal number of parameters provides the most robust and interpretable result.

Starting from the CASPT2-optimized *cis*-azo geometry, two structural parameters were refined: the ∠CNNC dihedral angle and the ∠CCNN angle. These two angles were selected because they exhibit the largest changes between the CASPT2-calculated *trans*-azo and *cis*-azo structures, whereas other structural parameters (the ∠CNN inversion angles, *r*NN, *r*CN, ∠CCCN improper angles, and ∠CCN angles) show only small changes (Extended Data Table 3) and were therefore fixed at their CASPT2 values during refinement. C_2 symmetry (with the C_2 axis perpendicular to N–N) was imposed, reflecting the symmetry of the equilibrium *cis*-azo structure ($∠CCNN_1$ = $∠CCNN_2$, one free parameter). The mean-square displacement parameter σ^2 was fixed at zero, because *cis*-azo is a long-lived ground-state product (formed with a time constant of 14 ps) that has sufficient time to relax vibrationally to thermal equilibrium before contributing to the measured $SADS_3$. Together with the scaling coefficient (= $\alpha \cdot \beta \cdot \gamma$), this gives a total of $k + 1 = 3$ free parameters ($k = 2$ motional parameters). The objective function was a Tikhonov-regularized χ^2:

$$F = \chi^2 + \lambda \cdot \mathrm{RMSD}^2(\mathbf{r}_{\mathrm{fitted}}, \mathbf{r}_{\mathrm{CASPT2}}) \qquad \text{(S21)}$$

Here, $\mathbf{r}_{\mathrm{CASPT2}}$ is the CASPT2-calculated *cis*-azo geometry, $\mathbf{r}_{\mathrm{fitted}}$ is the candidate *cis*-azo structure generated by applying the basis motions to $\mathbf{r}_{\mathrm{CASPT2}}$, and RMSD is computed over all 24 atoms after optimal Kabsch superposition. The regularization strength $\lambda = 1.0$ was determined by evidence maximization (Section 12.7). The *trans*-azo reference structure was fixed at its CASPT2 geometry throughout, and the simulated $SADS_3$ was computed as the difference between the Debye scattering curves of the candidate *cis*-azo and the reference *trans*-azo, with solvent cage terms included for both species. The simulated SADS was then subjected to the same PEPC projection as the experimental data to ensure that the comparison between experiment and simulation is performed on a consistent basis.

Starting from the zero-motion CASPT2 reference ($\chi^2 = 648.3$, $\chi_\nu^2 = 1.27$), the two-parameter refinement reduced χ^2 to 511.9 ($\chi_\nu^2 = 1.01$), a 21.0% improvement, with a concentration coefficient $\alpha \cdot \beta \cdot \gamma = (3.261 \pm 0.048) \times 10^{-4}$. The refined structure deviates from the CASPT2 reference primarily in the ∠CCNN angle (Δ∠CCNN = 22.4° ± 10.0°), with a modest change in ∠CNNC (Δ∠CNNC = 4.6° ± 7.4°). The RMSD from the CASPT2 reference (1.32 Å) is consistent with a structural refinement rather than a wholesale rearrangement, confirming that the CASPT2-calculated *cis*-azo structure serves as a reliable starting point. The full set of refined structural parameters is listed in Extended Data Table 3."

"**12.7 | Tikhonov regularisation for the $SADS_3$ *cis*-azo refinement**

The *cis*-azo geometry was determined through the refinement against $SADS_3$ described in Section 12.4; no separate I_2 → *cis*-azo SSPM was performed. This refinement employed Tikhonov regularisation toward the CASPT2 reference geometry. Because *cis*-azo is a ground-state (S_0) product, its equilibrium geometry is well described by CASPT2. In general, CASPT2 geometries can be less reliable for excited-state

species, where multi-reference character, conical-intersection proximity, and non-adiabatic dynamics complicate the calculation; however, these issues do not arise for a ground-state molecule such as *cis*-azo. The regularization term penalizes large deviations from this well-characterized reference, preventing the optimizer from settling into unphysical local minima while still allowing the experimental scattering data to refine the structure where the data warrant it. The validity of this approach is further supported by the refinement results (Section 12.4), which showed that the CASPT2 geometry requires only a two-parameter adjustment to match the experimental $SADS_3$ with a χ^2 improvement of 21.0%. In contrast, no regularisation was applied to the *trans*-azo → I_1 and I_1 → I_2 transitions (Sections 12.5 and 12.6), as the corresponding intermediates (I_1 and I_2) reside on the S_1 excited-state surface, where multi-reference character and conical-intersection proximity render CASPT2 geometries less reliable as structural priors.

The regularisation strength λ controls the balance between fidelity to the experimental data (χ^2) and proximity to the CASPT2 reference structure ($RMSD^2$). An optimal λ should be large enough to exclude unphysical solutions, but small enough to allow the data to drive the structural determination. We determined λ by evidence maximisation (Bayesian) approach following MacKay [ref], in which the optimal λ maximizes the marginal likelihood (evidence) P(D|λ), approximated via the Laplace approximation:

$$\ln P(D|\lambda) \approx -1/2 \cdot F(\hat{\theta}) + (k_{total} / 2) \cdot \ln(2\pi) - 1/2 \cdot \ln(\det\mathbf{H}) + (m / 2) \cdot \ln(\lambda / 2\pi) \qquad (S22)$$

$F(\hat{\theta})$ is the regularised objective function evaluated at the optimal parameters $\hat{\theta}$, **H** is the Hessian matrix of F at $\hat{\theta}$, k_{total} is the total number of free parameters ($k_{total} = k + 2$), and m is the number of regularized structural parameters. The Hessian was computed numerically by finite differences. Scanning λ over 25 logarithmically spaced values from 10^{-8} to 10^4 Å^{-2} followed by two rounds of local refinement (5 points each), the evidence exhibited a clear maximum at λ = 1.0 Å^{-2}, which was adopted for all subsequent analyses. At the optimal λ = 1.0, the regularisation penalty contributes only $\lambda \cdot RMSD^2_{cis}$ = 1.7 to the total objective function F = 513.6, representing less than 0.4% of the total cost. This confirms that the regularisation acts as a mild constraint rather than dominating the fit: the experimental data remain the primary driver of the structural determination, while the CASPT2 prior prevents the optimizer from exploring unphysical regions of parameter space. The resulting *cis*-azo geometry deviates from the CASPT2 reference by RMSD = 1.32 Å, consistent with a modest but statistically significant structural adjustment driven by the experimental scattering data."

The authors state that "It should be noted that the agreement between the experimental data (SADS3) and the simulation is excellent (Fig. 3f), indicating both the sufficient accuracy of the CASPT2 structures of trans-azo and cis-azo, and the high quality of the TRXL data. In contrast, the agreement for SADS1 and SADS2 is less satisfactory". Please quantify these agreements.

→ We thank the referee for this suggestion. We have now quantified the agreement using reduced chi-square (χ_v^2) values. Our initial statement that the agreement between the experimentally derived SADSs and the calculated ones from the CASPT2 structures was "excellent" for $SADS_3$ versus "less satisfactory" for $SADS_1$ and $SADS_2$ was based on the extent to which structural refinement improved the fit quality. Specifically, structural refinement reduced χ_v^2 by only 21% for $SADS_3$ (*cis*-azo), indicating that the initial CASPT2 structure was already very close to the optimal geometry. In contrast, the refinement yielded substantial reductions in χ_v^2 for $SADS_1$ (I_1) and $SADS_2$ (I_2), improving the fit by 41% and 55%, respectively. Nevertheless, we acknowledge that our original statement, which emphasized excellent agreement exclusively for $SADS_3$, could be misleading. Considering these points, we have revised the relevant paragraph in the Methods section to address both issues simultaneously. The revised text is as follows.

"As a first step toward structural validation, we compared the experimentally derived SADSs with their simulated counterparts from the CASPT2-calculated structures (Fig. 3f). To explore whether structures with better agreement with the experimental data exist, we then refined the structures of I_1, I_2 and *cis*-azo by

minimizing the reduced chi-square (χ_v^2), which quantifies the difference between the experimental and simulated SADSs, using the structural degrees of freedom associated with the candidate isomerization motions."

"$SADS_n'(q)$s calculated from the refined structures of I_1, I_2, and *cis*-azo demonstrated excellent agreement with the corresponding experimental $SADS_n(q)$s (Fig. 3f). For *cis*-azo, the refinement confirmed that the solution-phase structure of *cis*-azo deviates only mildly from the CASPT2-calculated structure, showing a relatively small improvement in χ_v^2 from 1.27 (CASPT2-calculated) to 1.01 (TRXL-refined), corresponding to a 21% reduction. This indicates both the sufficient accuracy of the CASPT2 structures of *trans*-azo and *cis*-azo, and the high quality of the TRXL data. In contrast, the refinement significantly improved the fits for I_1 and I_2, reducing the χ_v^2 values from 0.50 to 0.30 for $SADS_1$ (41% reduction) and from 0.66 to 0.30 for $SADS_2$ (55% reduction). This indicates that the CASPT2-calculated structures of I_1 and I_2 deviate from their TRXL-refined counterparts to a greater extent than in the case of *cis*-azo. The structures of I_1, I_2, and *cis*-azo obtained from i) CASPT2 calculations and ii) structural refinement using fs-TRXL data, are compared in Extended Data Table 3, and a detailed comparison of these intermediate structures is provided in Supplementary Note 6.

It is worth noting that some of the χ_v^2 values are near or well below unity. This is a well-known characteristic of X-ray scattering data analysis, arising from the fact that the effective number of independent information channels is fundamentally smaller than the number of measured q-points. According to the Shannon sampling theorem, the independent information content of a scattering curve is governed by the Shannon number, $N_s \approx (q_{\max} - q_{\min}) \times D_{max} / \pi$, where $q_{\max}$ and $q_{\min}$ are the upper and lower limits of the measured q-range, respectively, and D_{max} is the maximum interatomic distance contributing to the scattering signal. For a solute molecule such as azobenzene ($D_{max} \approx$ 12–15 Å), N_s is estimated to be approximately 25 for the q-range of 1–7 $Å^{-1}$ used in this study. However, in solution-phase scattering experiments, the signal also includes bulk solvent response and cage contributions, which extend to distances significantly larger than the solute dimensions (> 30–60 Å). This effectively increases the independent information content to $N_s \approx$ 60–120. Because the distance range over which these solvent contributions affect the scattering signal is not precisely defined, the effective D_{max}, and thus the true Shannon number, cannot be uniquely determined for solution scattering data. Since adjacent q-points carry redundant structural information beyond the Shannon limit, using the total number of q-points as the degrees of freedom in the χ_v^2 calculation results in systematically small values. Therefore, the observed χ_v^2 values below unity do not indicate overfitting but rather reflect this inherent property of solution scattering data. A rigorous determination of N_{eff} using a BIC-based polynomial analysis is described in Supplementary Method 13. Moreover, one important caveat is that directly comparing the absolute χ_v^2 values obtained for different SADS (e.g., comparing the χ_v^2 of $SADS_1$ with that of $SADS_3$) can be misleading. This stems from the fact that the χ_v^2 statistic is normalized by the uncertainties (σ) of the extracted SADS, and in linear least-squares analysis (including SVD), the uncertainty of a signal component S_k is inversely proportional to the norm of its concentration profile $C_k(t)$:

$$\sigma_{S_k} \propto 1/\sqrt{\sum_t \left(C_k(t)^2\right)} \quad (2)$$

This relationship implies that a species with a higher concentration and longer duration (i.e., a larger ΣC^2) will therefore have a much smaller uncertainty σ_{S_k} and correspondingly amplified χ_v^2 values. In our dataset, the *cis*-azo species persists up to the latest measured time delay without decay, giving it the largest integrated concentration profile among all species. As a result, the σ associated with $SADS_3$ (*cis*-azo) is systematically much smaller than those for the transient intermediates $SADS_1$ and $SADS_2$. Consequently, χ_v^2 values for $SADS_3$ (or fits dominated by it) are mathematically amplified and tend to appear larger than those of $SADS_1$ and $SADS_2$, even for strictly comparable fit qualities. Therefore, comparisons of χ_v^2 values among candidate

structural models for the same species should be the primary criterion for model assessment, rather than cross-species absolute comparisons."

The authors should consider including a table that provides an overview of the different motion mechanisms, the degrees of freedom associated with each mechanism and combination of mechanisms, and the corresponding (reduced) chi-squared values. This would allow readers to easily compare models and assess the quality of fit.

→ We thank the referee for this excellent suggestion. We have added Supplementary Table 2, which systematically compares χ^2 values and BIC values for all possible combinations of the four elementary structural motions (ROT, INV, NND, and CNT) across the structural transitions (*trans*-azo → I_1 and I_1 → I_2). Definitions and implementations of the structural parameters used to describe the ROT, INV, NND, and CNT motions are provided in the "Structure analysis" subsection in the Methods section.

Supplementary Table 2 | Structural motions and their combinations evaluated in the SSPM analysis. For each combination, the table lists the fitting parameters, the number of active motion parameters (k), and the resulting χ^2 and ΔBIC(χ^2) values. The χ^2 and BIC(χ^2)values correspond to the SSPM analysis presented in Extended Data Fig. 6, where various combinations of structural motions are evaluated to determine the simplest structural models consistent with the experimental data. For each structural transition indicated by the arrow (→, indicating a transition from the initial structure to the target structure), the χ^2 and BIC(χ^2) values corresponding to the best structural model are highlighted in bold.

Structural motions	Fitting parameters*	SADS1(q): *trans*-azo → I_1			SADS$_2$(q): I_1 → I_2		
		$k^{\#}$	χ^2	ΔBIC(χ^2)	$k^{\#}$	χ^2	ΔBIC(χ^2)
ROT	∠CNNC (°), σ^2 (Å^2), c	1	280.8	129.9	1	177.5	16.5
INV	∠CNN_1 (°), ∠CNN_2 (°), σ^2 (Å^2), c	1	236.6	85.7	2	216.5	60.2
NND	rNN (Å), σ^2 (Å^2), c	1	277.9	127.0	1	241.2	80.2
CNT	∠$CCNN_1$ (°), ∠$CCNN_2$ (°), σ^2 (Å^2), c	**1**	**150.9**†	**0.0**†	2	256.6	100.3
ROT + INV	∠CNNC (°), ∠CNN_1 (°), ∠CNN_2 (°), σ^2 (Å^2), c	2	236.6	90.4	3	176.7	25.1
ROT + NND	∠CNNC (°), rNN (Å), σ^2 (Å^2), c	2	277.9	131.7	2	177.1	20.8
ROT +	∠CNNC (°),	2	150.9	4.7	3	**151.6**†	**0.0**†

CNT	∠CCNN$_1$ (°), ∠CCNN$_2$ (°), σ^2 (Å^2), *c*						
INV + NND	∠CNN$_1$ (°), ∠CNN$_2$ (°), *r*NN (Å), σ^2 (Å^2), *c*	2	231.3	85.1	3	215.7	64.1
INV + CNT	∠CNN$_1$ (°), ∠CNN$_2$ (°), ∠CCNN$_1$ (°), ∠CCNN$_2$ (°), σ^2 (Å^2), *c*	2	150.9	4.7	4	199.8	52.9
NND + CNT	*r*NN (Å), ∠CCNN$_1$ (°), ∠CCNN$_2$ (°), σ^2 (Å^2), *c*	2	149.5	3.3	3	236.3	84.7
ROT + INV + NND	∠CNNC (°), ∠CNN$_1$ (°), ∠CNN$_2$ (°), *r*NN (Å), σ^2 (Å^2), *c*	3	231.3	89.8	4	176.7	29.8
ROT + INV + CNT	∠CNNC (°), ∠CNN$_1$ (°), ∠CNN$_2$ (°), ∠CCNN$_1$ (°), ∠CCNN$_2$ (°), σ^2 (Å^2), *c*	3	150.9	9.4	5	142.5	0.3
ROT + NND + CNT	∠CNNC (°), *r*NN (Å), ∠CCNN$_1$ (°), ∠CCNN$_2$ (°), σ^2 (Å^2), *c*	3	149.5	8.0	4	149.8	2.9
INV + NND + CNT	∠CNN$_1$ (°), ∠CNN$_2$ (°), *r*NN (Å), ∠CCNN$_1$ (°), ∠CCNN$_2$ (°), σ^2 (Å^2), *c*	3	149.5	8.0	5	198.6	56.4
ROT + INV + NND + CNT	∠CNNC (°), ∠CNN$_1$ (°), ∠CNN$_2$ (°), *r*NN (Å), ∠CCNN$_1$ (°), ∠CCNN$_2$ (°), σ^2 (Å^2), *c*	4	149.5	12.7	6	142.5	5.0

* Fitting parameter definitions:
∠CNNC (°): The CNNC dihedral angle describing torsional rotation around the N–N bond, used to parameterize the rotational (ROT) motion of the azo group.
$\angle CNN_1$ and $\angle CNN_2$ (°): The C–N–N bond angles at the two phenyl-azo junctions, independently describing inversion (INV) motions on each side of the azo group.
*r*NN (Å): The N–N bond length, used to represent the N–N bond stretching motion (NND).
$\angle CCNN_1$ and $\angle CCNN_2$ (°): The dihedral angles describing the relative twist of each phenyl ring with respect to the CNN plane about the respective C–N bond, used to parameterize the CNT motion.
σ^2 ($Å^2$): The mean-squared displacement parameter introduced through a Debye–Waller factor to account for thermal broadening of interatomic distances in the calculated scattering curves.
c: The scaling coefficient, which relates the experimentally observed SADS amplitude to the unit-concentration simulated SADS (see Section 12.3). For $SADS_1$, $c = \alpha$; for $SADS_2$, $c = \alpha \cdot \beta$.
[#] k denotes the number of active motion parameters, excluding σ^2 and c. The total number of fitting parameters is therefore $k + 2$. For the $SADS_1$ analysis, C_i symmetry was imposed, constraining $\angle CNN_1 = \angle CNN_2$ and $\angle CCNN_1 = -\angle CCNN_2$, so that INV and CNT each contribute one free parameter rather than two. For the $SADS_2$ analysis, no symmetry constraint was imposed, and all parameters were varied independently. The BIC was computed as $\mathrm{BIC}(\chi^2) = \chi^2 + (k + 2) \cdot ln(N_{eff})$, where $N_{\mathrm{eff}} = 110$ is the effective number of independent data points determined by polynomial-order optimization via BIC minimisation (see Supplementary Method 13).
[†]The best model for each transition was selected based on the minimum $\mathrm{BIC}(\chi^2)$. For the $I_1 \rightarrow I_2$ transition, CNR + INV + OHT ($k = 5$) yielded the lowest BIC (222.3), but CNR + ROT ($k = 3$, BIC = 223.7, ΔBIC = 1.4) achieves a comparable fit with fewer parameters and was therefore selected as the best model. For the *trans*-azo → I_1 transition, CNT ($k = 1$, $\mathrm{BIC}(\chi^2) = 165.0$) is the best model; the next-ranked model, NND + CNT ($k = 2$, $\mathrm{BIC}(\chi^2) = 168.3$, $\Delta\mathrm{BIC}(\chi^2) = 3.3$), does not achieve a statistically significant improvement, confirming that the CNT motion alone is sufficient to describe this transition. For the $I_1 \rightarrow I_2$ transition, ROT + CNT ($k = 3$, $\mathrm{BIC}(\chi^2) = 175.1$) yields the lowest $\mathrm{BIC}(\chi^2)$; the next-ranked model, ROT + INV + CNT ($k = 5$, $\mathrm{BIC}(\chi^2) = 175.4$, $\Delta\mathrm{BIC}(\chi^2) = 0.3$), indicates that INV does not provide a statistically justified improvement. ROT ($k = 1$, $\mathrm{BIC}(\chi^2) = 191.6$, $\Delta\mathrm{BIC}(\chi^2) = 16.5$) is substantially worse, confirming that CNT is a necessary component for this transition.

The authors mention that "The structures of the intermediates generated by applying these atomic motions were used to calculate the solute-only and cage scattering signals. The structural parameters used to generate the intermediate structures were optimized to ensure that the SADS'(q)s obtained from these structures accurately represent the SADS(q)s". Could the authors clarify whether they calculated a set of structures with varying parameters and compared these to the data, or whether the structural parameters were directly optimized during the chi-squared fitting process?
→ We thank the referee for requesting this clarification. The structural parameters were iteratively optimized through a direct chi-square minimization fitting process, rather than calculating a pre-defined set of structures for comparison. Specifically, we employed a nonlinear least-squares optimization algorithm that iteratively adjusted the structural parameters to minimize the discrepancy between the simulated and experimental SADSs. We have revised the manuscript to explicitly state this procedure:
"The structural parameters corresponding to these four motions were optimized through reduced chi-square minimization to ensure the best agreement between the simulated SADS′(q) and experimental SADS(q)."
The complete fitting procedure, including parameter bounds, scaling factor determination, symmetry constraints, and the extended seven-motion validation, is described in the newly added Supplementary Method 12.

Ab-initio calculations

The authors state that "An imaginary shift of 0.2 a.u. was introduced to prevent the inclusion of intruder states during the calculations." Could the authors provide more information on this and explain what is meant by an "imaginary shift"?

→ We thank the referee for requesting clarification on this technical detail. In CASPT2 calculations, intruder states can arise when a reference state becomes accidentally near-degenerate with external configurations in the perturbative expansion, leading to singularities and numerical instabilities. An imaginary level shift is a standard regularization technique that suppresses these unphysical singularities by adding an imaginary term to the denominator of the perturbation expression. This effectively damps the contributions from near-degenerate intruder states while leaving the energies of physically relevant states (which are well-separated from the singularities) essentially unaffected. The value of 0.2 a.u. employed in this work is a standard choice in the literature for excited-state calculations, providing a robust balance between stability and accuracy.

We have added the following explanation and citation to the "*Ab initio* calculations" section of the revised manuscript:

"An imaginary shift of 0.2 a.u., a standard value for CASPT2 excited-state calculations, was employed to prevent numerical instabilities caused by intruder states (accidental near-degeneracies) in the perturbative treatment.[61]"

Reference 61: N. Forsberg and P.-Å. Malmqvist, *Chem. Phys. Lett.* **274**, 196–204 (1997).

In addition, for the sake of transparency, a careful rereading of the manuscript prompted by the referee's comment led us to identify a few points where the description of the *ab initio* methodology could be clarified. In particular, the original wording did not make it sufficiently explicit that all reported stationary structures were fully optimized at the XMS-CASPT2 level, rather than at the CASSCF level. Moreover, an oversight in the description of the state-averaging protocol used in the CASSCF reference was corrected. These points have now been addressed in the revised "*Ab initio* calculations" section to improve clarity and ensure full methodological consistency. The following relevant sentences were added to the revised manuscript.

"To address this gap, we carried out full geometry optimizations for the four key structures directly at the extended multistate CASPT2 (XMS-CASPT2) level. "

and

"The CASSCF reference was optimized using a state-averaged (SA) CASSCF approach including the two lowest singlet states (S_0 and S_1(nπ*))."

Extended Data Figures

Extended Data Fig. 1 and 2

Panel e shows an anisotropy feature near 1.5 Å^{-1} that persists for over 10 ps... Could the authors clarify or speculate on the possible mechanism behind this feature?

→ The persistent anisotropy feature observed near 1.5 Å^{-1} (Extended Data Fig. 1e and 2e) arises from two physically distinct but spectrally indistinguishable sources: (i) the impulsive rotational response of the solute induced by the linearly polarised pump pulse, analogous to the optical Kerr effect but acting on the solute rather than the bulk solvent, and (ii) the photoselective depletion (anisotropic hole) of the *trans*-azo ground-state population. Both mechanisms alter the orientational distribution of the *trans*-azo ensemble and therefore produce the same *q*-dependent scattering profile. The kinetic analysis of the anisotropic signal (Extended Data Fig. 3f) yields an IRF-limited instantaneous rise followed by two characteristic time constants: 1.9 ± 0.5 ps and 22 ± 2 ps. The instantaneous rise is prominent in the anisotropic signal but weak in the isotropic signal, consistent with an impulsive reorientation that changes the orientational distribution without

altering the intramolecular structure. We note that the bulk solvent optical Kerr effect does not contribute here, as it was independently measured and removed through the PEPC procedure. SVD analysis identifies only a single significant anisotropic component despite the coexistence of the ground-state hole and the excited-state species evolving toward I_1. This is a direct consequence of the structural similarity between I_1 and *trans*-azo: the *trans*-azo → I_1 transition involves only CNT, leaving the azo backbone unchanged, so the hole and I_1 contributions are nearly identical in *q*-space and partially cancel. The subtle rise at 1.9 ps is attributed to the I_1 → I_2 transition, which removes this cancelling I_1 contribution. The signal fully vanishes with a time constant of 22 ± 2 ps, assigned to the rotational dephasing of the ground-state *trans*-azo ensemble. No detectable anisotropy from I_2 or *cis*-azo is observed, which we attribute to the large-amplitude ROT motion (70% LOO contribution) that randomizes the orientational distribution upon I_2 formation.

We have expanded the discussion in Supplementary Note 2 to describe these mechanisms in detail, including the distinction between the impulsive rotational response and the photoselective depletion, and their kinetic signatures.

The color scale in Extended Data Fig. 1e appears inconsistent in comparison to Extended Data Fig. 2e. Can the authors verify that the color scales are accurate?

→ We appreciate the careful reading. The color bars in Extended Data Fig. 2 contained an error, which has been corrected in the revised manuscript. Furthermore, to ensure consistency throughout the manuscript, we have unified the units of signal intensity in all relevant plots to electron units (e.u.) per solvent molecule. We added the following paragraph explaining the conversion to e.u.

"The experimental scattering intensities were calibrated to absolute units of electron units (e.u.) per solvent molecule using the following procedure. First, the total scattering curve of the solvent was calculated from the radial distribution function *g*(*r*) obtained from molecular dynamics (MD) simulations, which yields the scattering intensity in electron units. This calculated curve was then normalized by the number of solvent molecules in the MD simulation box to yield the intensity in units of e.u. per solvent molecule. A scaling factor was determined by comparing the normalized experimental total scattering curve with this simulated curve in units of e.u. per solvent molecule. The use of the simulated scattering curve of the pure solvent to calibrate the experimental solution data is justified because the experimental total scattering intensity of the solution arises almost exclusively from the bulk solvent, given the low concentration of the solute (50 mM). Finally, this scaling factor was applied to all experimental difference scattering curves, $\Delta S_{[x]}(q, t)^{raw}$, allowing the experimental signals to be expressed in e.u. per solvent molecule."

Extended Data Fig. 4

The authors identified three significant components. Based on the reported measures (singular values and autocorrelations), could the authors provide a quantitative rationale for selecting three components rather than a different number?

→ We selected three significant components based on quantitative criteria involving singular values and the temporal autocorrelation of the RSVs. Specifically, the first three components exhibit singular values significantly above the noise plateau and high autocorrelation coefficients (> 0.8), indicating robust, non-random signal contributions. In contrast, the fourth component shows a sharp drop in autocorrelation (< 0.5) and a singular value approaching the noise level, suggesting it is dominated by noise. We have added a detailed quantitative justification to Supplementary Method 1 ("Singular value decomposition (SVD) and kinetic analysis of isomerization kinetics") of the revised manuscript as follows:

"Based on the singular values and autocorrelation coefficients C(U) and C(V), we identified three significant components. Specifically, the first three components exhibited singular values well above the noise plateau and high autocorrelation coefficients (C(V) > 0.8), indicating robust non-random signal contributions. In contrast,

the fourth component showed a marked decrease in autocorrelation (C(V) < 0.5) and a singular value approaching the noise level, suggesting it is dominated by noise (Extended Data Fig. 4c). These support the involvement of three structurally distinct reaction species (intermediates and/or products) in the isomerization process. ”

Extended Data Fig. 5

The authors should expand the caption. While the Supplementary Methods provide an interpretation of the SADS in real space, the caption should also include a brief discussion of the key features displayed in the figure to aid immediate understanding.

→ We have added a new section (Supplementary Note 12) explaining Extended Data Fig. 5. Specifically, we now clarify that while Fig. 3f presents the *q*-space data including both solute and solvent cage contributions, Extended Data Fig. 5 presents the real-space (*r*-space) comparison focusing strictly on the solute-only signal.

Revised caption:

“**Extended Data Fig. 5 | Solute-only SADSs in *r*-space.** Experimental (black curves) and simulated (red curves) SADSs are presented as $r^2 SADS_{solute}(r)$ in *r*-space. In the difference RDFs, negative amplitudes indicate interatomic distances depleted relative to *trans*-azo, while positive amplitudes indicate newly formed distances. A detailed discussion is provided in Supplementary Note 12.”

Added section:

“**Supplementary Note 12 | Solute-only SADSs in real space**

Experimental and simulated SADSs for the solute-only term in real space (*r*-space), $r^2 SADS_{solute}(r)$, are presented in Extended Data Fig. 5. This *r*-space representation serves as a complementary validation to the *q*-space data shown in Fig. 3f. While the *q*-space comparison in Fig. 3f includes contributions from both the solute and the cage terms, the *r*-space comparison presented here compares the experimental data with the calculated signal from the solute structure alone (excluding the cage term). In these difference RDFs, negative amplitudes indicate a loss of atomic pair density at the corresponding distance compared to *trans*-azo, while positive amplitudes indicate an increase in atomic pair density at the corresponding distance.

The *r*-space signal for I_1 is the weakest among the three SADSs, consistent with the relatively small structural change from *trans*-azo to I_1, which primarily involves a change in the ∠CCNN dihedral angle while the overall molecular framework remains largely intact. The difference RDF does not exhibit a single dominant feature but instead shows weak negative amplitudes at both short (~2.4 Å) and long (~9 Å) distances, with modest positive amplitudes at intermediate distances (~3–5 Å). This pattern reflects the breaking of molecular planarity: the loss of the planar *trans* geometry redistributes interatomic distances that were previously at the extremes (the shortest and longest intramolecular distances in the planar configuration) toward intermediate values.

The *r*-space signal for I_2 shows a clear positive feature at shorter distances (~3–5 Å) and a negative feature at longer distances (~7–9 Å). This clear separation indicates that the molecular structure becomes more compact upon the $I_1 \rightarrow I_2$ transition, with the two phenyl rings approaching each other. The depletion of long-range interatomic distances (negative peak) and the concurrent formation of shorter distances (positive peak) together reflect the progressive folding of the molecular backbone as the CNNC dihedral angle decreases.

The *r*-space features observed in $SADS_2$ become even more pronounced in $SADS_3$. Both the positive peak at ~3.5–5 Å and the negative peak at ~7 Å significantly increase in amplitude compared to $SADS_2$, indicating that the structural difference from *trans*-azo becomes substantially larger upon the $I_2 \rightarrow$ *cis*-azo transition.

The progressive increase in signal magnitude from $SADS_1$ to $SADS_2$ to $SADS_3$ is consistent with the progressive decrease in R_g along the isomerization pathway (Extended Data Table 3). The overall agreement

between experiment and simulation confirms that the extracted experimental SADSs accurately represent the intrinsic structural changes of the azobenzene molecule, effectively isolated from the bulk solvent contribution.”

Extended Data Fig. 6

The authors should either consider including an additional statistical measure or provide more detail on the reported chi-squared statistics. As currently presented, the chi-squared values cannot be meaningfully evaluated by the reader.

→ We thank the referee for this constructive comment. To enable meaningful evaluation of the fit quality, we applied the Bayesian Information Criterion (BIC), which augments the χ^2 goodness-of-fit with an explicit complexity penalty proportional to the number of free parameters, thereby favouring the most parsimonious model consistent with the data. In each candidate model, the total number of free parameters is $k + 2$, where k is the number of active structural (motional) parameters and the two additional parameters are the mean-square displacement parameter σ^2 and the scaling coefficient. The full statistical framework is described in Supplementary Method 13. Applying this criterion, the BIC-best model for the *trans*-azo → I_1 transition is CNT alone ($k + 2 = 3$, $\chi^2 = 150.9$, $\chi_v^2 = 0.297$, BIC(χ^2) = 165.0); all models extending CNT by adding ROT, INV, NND, or their combinations yielded larger BIC values (Supplementary Table 2). For the I_1 → I_2 transition, the BIC-best model is ROT + CNT ($k + 2 = 5$, BIC(χ^2) = 175.1), while the full four-motion model ($k + 2 = 8$, BIC(χ^2) = 180.1, ΔBIC(χ^2) = 5.0) showed no significant improvement, confirming that neither NND nor INV is statistically required. The complete SSPM and BIC results, including all candidate models and their χ^2 and BIC(χ^2) values, are tabulated in Supplementary Table 2 and discussed in Supplementary Note 3. Extended Data Fig. 6 was replotted to reflect the updated values.

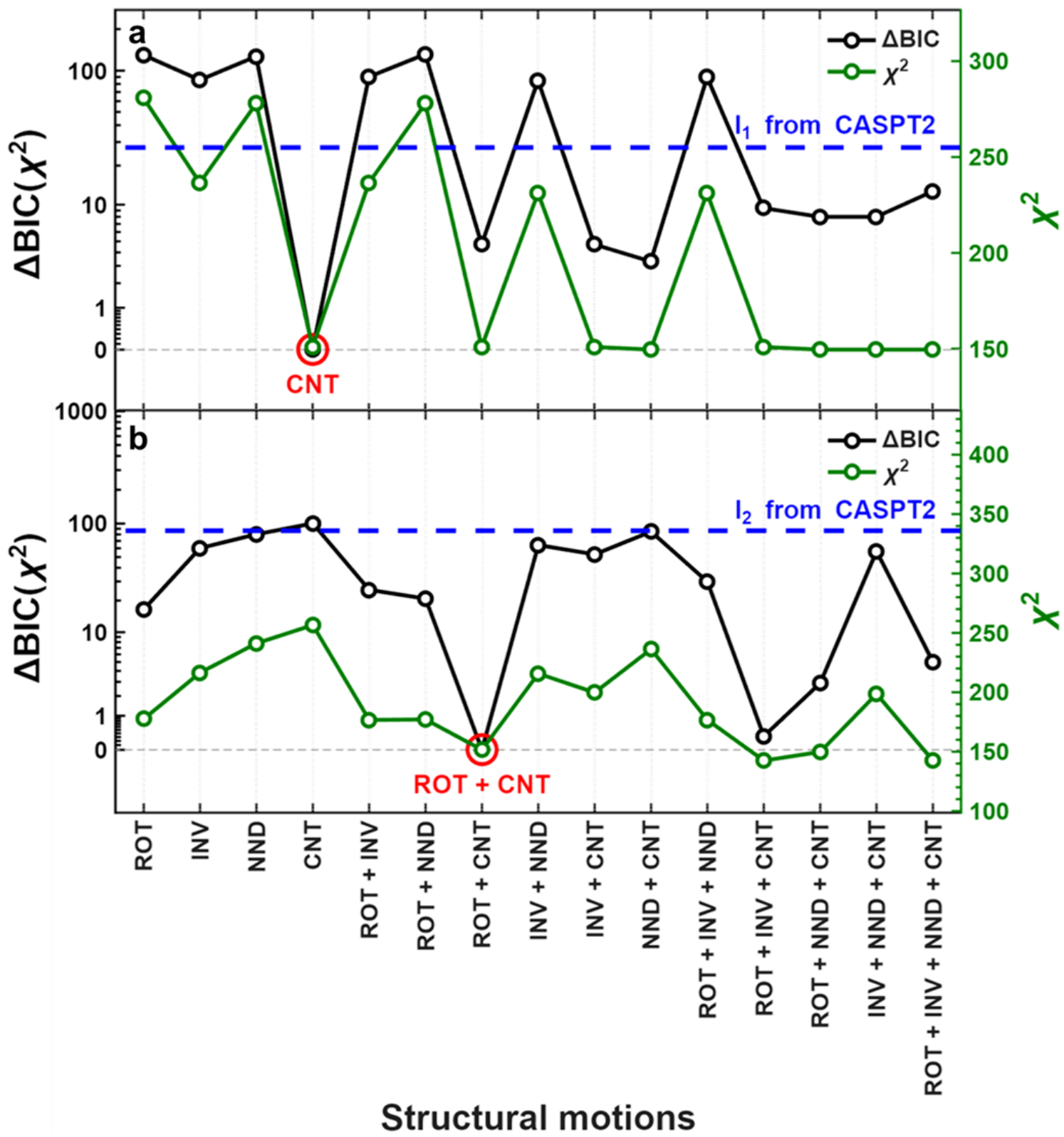

Extended Data Fig. 6 | Selection of parsimonious structural motions using SSPM (Systematic Scan for Parsimonious Motions). SSPM identifies the simplest combination of structural motions consistent with the experimental data by evaluating all possible combinatorial subsets of the four basis motions and ranking them using the Bayesian Information Criterion (BIC). **a**, SSPM results for I_1 starting from *trans*-azo. **b**, SSPM results for I_2 starting from I_1. In all panels, the right *y*-axis shows χ^2 values (black) for visual reference, and the left *y*-axis shows $\Delta BIC(\chi^2)$ values (red), defined as the difference in BIC relative to the best (lowest-BIC) model. BIC serves as the sole criterion for model selection; $\Delta BIC = 0$ corresponds to the optimal model, and larger values indicate progressively less favourable models. The *x* axis denotes different combinations of structural motions. The four basis motions considered are C–N bond torsion (CNT), N–N bond rotation (ROT), inversion (INV), and N–N bond-length change (NND), evaluated individually and in all possible combinations (15 models in total). The dashed horizontal lines represent the χ^2 values calculated from the CASPT2-calculated structures of I_1 and I_2. The BIC-optimal models are highlighted with circles. I_1 is most parsimoniously described by CNT alone, and I_2 is most parsimoniously described by ROT + CNT.

Supplementary Methods

Singular value decomposition (SVD) and kinetic analysis of isomerization kinetics

Could the authors clarify what is meant by "the top three singular-value-weighted RSVs were globally fitted"? How was the global fit applied to the three components?

→ We clarified that the global fitting procedure was performed by minimizing the total χ^2_{total} , which is defined as the sum of the χ^2 values for each of the top three RSVs (χ^2_n). This approach ensures that a single set of global time constants optimally describes all RSV components simultaneously. We have added the explicit mathematical definitions to Supplementary Method 1 of the revised manuscript.

"The top three RSVs were globally fitted using the sum of three exponential functions to extract kinetic information about the isomerization process. Specifically, the global fitting was performed by minimizing the total chi-square χ^2_{total}, which is defined as the sum of the chi-square values for each of the top three RSVs χ^2_n:

$$\chi^2_{total} = \sum_{n=1}^{3} \chi^2_n \qquad \text{(S1)}$$

χ^2_n represents the chi-square fit quality for the nth RSV:

$$\chi^2_n = \sum_k [(RSV_n^{exp}(t_k) - RSV_n^{calc}(t_k))/\sigma_n(t_k)]^2 \qquad \text{(S2)}$$

Here, $RSV_n^{exp}(t_k)$ is the experimental value of the nth RSV at time delay t_k, and $\sigma_n(t_k)$ is the standard error of $RSV_n^{exp}(t_k)$. The standard error $\sigma_n(t_k)$ was obtained by propagating the standard error of the mean of the experimental difference scattering data. Specifically, for error estimation, the relationship between the experimental data and the RSVs was modeled as a weighted linear problem: at each time delay, the difference scattering curve $\Delta S_{iso}^{\perp}(q, t_k)$ was represented as a linear combination of the LSVs, where the expansion coefficients correspond to the RSV values weighted by their corresponding singular values. Using this linear model, the covariance matrix of the RSV values was calculated within the standard weighted least-squares formalism, properly accounting for the experimental uncertainties ($\sigma_{iso}^{\perp}(q, t_k)$) at each q-point. The diagonal elements of this covariance matrix provide the standard errors $\sigma_n(t_k)$ of the RSV values. $RSV_n^{calc}(t_k)$ denotes the theoretically calculated curve using a multi-exponential model convolved with the instrument response function (IRF):

$$RSV_n^{calc}(t_k) = (\sum_{j=1}^{3} A_{nj} \cdot exp(-t/\tau_j) \cdot H(t)) \otimes IRF(t) \qquad \text{(S3)}$$

In this equation, the term A_{nj} represents the amplitude of the jth exponential function specifically for the nth RSV, serving as a local parameter optimized independently for each RSV. τ_j represents the time constant of the corresponding exponential function, treated as a global parameter shared across all RSVs (n = 1, 2, 3) to ensure a consistent kinetic model. $H(t)$ is the Heaviside step function ($H(t)$ = 1 for $t \geq$ 0, 0 otherwise), ensuring that the signal onset occurs only after photoexcitation. The symbol ⊗ denotes a convolution operation. Finally, $IRF(t)$ is the instrument response function (IRF), modeled as a Gaussian function with a full width at half maximum (FWHM) of approximately 170 fs."

Obtaining species-associated difference scattering curves

Could the authors include the explicit equation used to calculate the concentration of trans-azo forming I1 from the reported alpha value?

→ We have added the explicit equation to Supplementary Method 12. The concentration of *trans*-azo forming I_1 is calculated from the optimized scaling factor α via the relation $c(I_1) = \alpha \times (\rho / m)$, where ρ is the solvent density (≈ 792 g/L) and m is the solvent molecular weight (≈ 32.04 g/mol). With the optimized $\alpha = (3.672 \pm 0.635) \times 10^{-4}$, this yields $c(I_1) = 9.1 \pm 1.6$ mM.

Chi-square as a fitting criterion for the structural analysis of SADSs

Could the authors include a figure of the theoretical signals before and after PEPC treatment?

→ In the revised manuscript, we have added Supplementary Fig. 6, which presents the theoretical scattering curves calculated from the optimized structures before and after the application of the PEPC treatment. As PEPC projects the signal onto a subspace orthogonal to the solvent, the shape of the solute signal is mathematically modified. This figure illustrates exactly how the projection transforms the theoretical signal. By comparing the PEPC-treated experimental data with these PEPC-projected theoretical curves (rather than the raw theoretical curves), we ensure a mathematically consistent and rigorous structural refinement. We

added the following relevant sentence to Supplementary Method 3 titled "Chi-square as a fitting criterion for the structural analysis of SADSs".

"A comparison between the $\mathrm{SADS}_i'(q)$ and their PEPC-projected counterparts ($\mathrm{SADS}_i'^{\perp}(q)$) is presented in Supplementary Fig. 6 to explicitly illustrate how the projection transforms the theoretical signal."

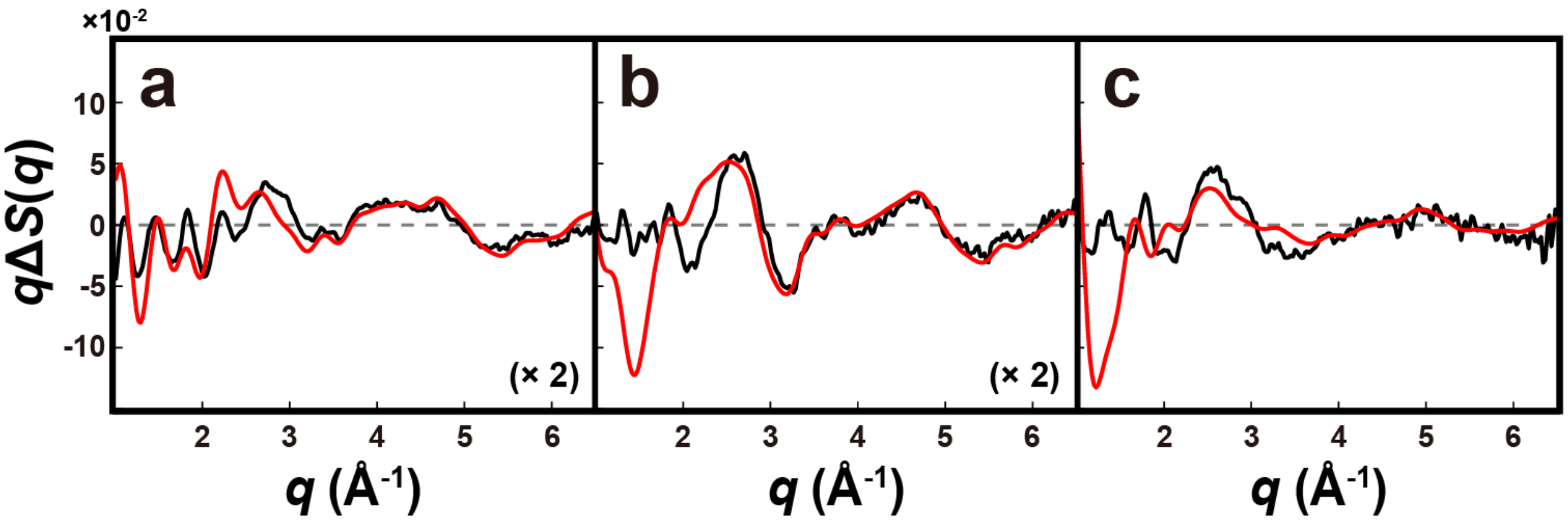

Supplementary Fig. 6 | Comparison of theoretical difference scattering curves before and after PEPC treatment. a–c, Comparison of the raw theoretically calculated species-associated difference scattering curves ($\mathrm{SADS}_n'(q)$s, red lines) and the corresponding PEPC-treated theoretical curves ($\mathrm{SADS}_n'^{\perp}(q)$s, black lines) for the refined structures: (**a**) I_1, (**b**) I_2, and (**c**) *cis*-azo. The PEPC operator projects the signal onto a subspace orthogonal to the dominant solvent scattering components, mathematically modifying the shape of the solute signal. The structural refinement minimizes the discrepancy between the experimentally extracted PEPC-treated SADS and these PEPC-projected theoretical curves, ensuring a mathematically consistent scaling within the solvent-free subspace. For clarity, selected panels are displayed with a vertical scaling factor of 2 as indicated.

The authors mention performing a baseline correction during chi-squared fitting because the SADS may contain residual baseline features. Could the authors clarify what these residual features arise from? Since this procedure is applied to a novel dataset, it would also be helpful to provide more detail on the data analysis steps and visualize key parts of the process, rather than referring to studies involving strongly scattering heavy atoms.

→ We have expanded Supplementary Method 3 to clarify the physical origin of these baseline features and the mathematical justification for their correction. The residual baseline features arise primarily from fluctuations in the liquid jet thickness and position, which alter the liquid-to-air scattering ratio and create broad, low-frequency residuals in q-space. The validity of correcting these artifacts with a low-order polynomial baseline is grounded in the Debye equation: since the self-scattering term cancels in the difference signal, the structural information resides in the inherently oscillatory interference term ($\sin(qr_{ij})/qr_{ij}$), which is unaffected by a smooth baseline. The baseline correction term $B_i(q)$ is now explicitly incorporated into the chi-square definition (equation S12), and the reduced chi-square is defined in equation S13 with all free parameters clearly specified. Furthermore, to provide the requested visualization of the analysis steps, we have added a comprehensive data processing flowchart (Supplementary Fig. 1) that illustrates the entire workflow, including the baseline correction step.

The authors determine the optimal alpha values by minimizing the chi-squared statistic. Could the authors clarify how alpha depends on the structural models? Do the alpha values vary significantly across different structures, or are they largely consistent?

→ The scaling factor α and the structural parameters are interdependent and therefore optimized simultaneously within each SADS fit, as described in Supplementary Method 12. The value of α determines the absolute magnitude of the target SADS that the structural model must reproduce: a different α value requires the structural model to match a different signal intensity, thereby influencing the optimal structural parameters, and conversely, a different structural model necessitates a different α value to best match the experimental signal. This interdependence is explicitly reflected in equation (S11), where α enters through the scaled SADS (e.g., $SADS_1^{\perp *}(q) = \alpha \cdot SADS_1^{\perp}(q)$). In practice, the optimized α values are largely consistent across all models that include CNT (the BIC-selected motion), ranging from 3.63 to 3.67 × 10^{-4} (corresponding to 9.0–9.1 mM), while models lacking CNT yield higher α values (up to 5.53 × 10^{-4}, corresponding to 13.7 mM) with correspondingly poorer fit quality. Overall, the concentration of *trans*-azo forming I_1 ranges from 9.0 to 13.7 mM across all candidate models. This demonstrates that the reaction yield determination is robust among statistically competitive models.

Error estimation for structural parameters

Please clarify the meaning of a 1-unit increase in the chi-squared value in the context of the study.

→ In chi-square statistics, an increase in the non-reduced chi-square by 1 from its minimum ($\Delta\chi^2 = 1$) corresponds to the 1σ confidence interval (68.3% probability) for a single parameter of interest. In the original manuscript, we estimated uncertainties using the Hessian (curvature) matrix at the χ^2 minimum, which relies on a parabolic approximation of the χ^2 surface. In the revised manuscript, we replaced this with the profile likelihood method, which provides a more rigorous treatment. For each structural parameter θ_i, the profile likelihood $\chi^2_{\text{profile}}(\theta_i)$ was constructed by fixing θ_i at a series of values while re-optimizing all remaining free parameters at each scan point. The confidence interval bounds were then determined from the values of θ_i at which $\chi^2_{\text{profile}}(\theta_i) - \chi^2_{\text{min}} = 1$. This approach does not rely on a parabolic approximation, naturally accounts for parameter correlations, and permits asymmetric error bars. The detailed procedure is described in Supplementary Method 4.

Debye equation for simulating the scattering signal from solute structures

The authors introduce Debye-Waller factors to account for broadening caused by vibrational motion in azobenzene. They consider Debye-Waller factors for the refinement of structures I1 and I2. Since cis-azo is populated during the photoreaction and presumably vibrationally hot, could the authors comment on whether the assumption that cis-azo is a structurally stable ground-state molecule holds at the investigated time scales.

→ We appreciate the referee for raising this valid concern. We justify the assumption that *cis*-azo can be treated as a structurally stable, vibrationally relaxed ground-state molecule based on kinetic considerations and the absence of spectral signatures indicative of vibrational cooling. In polar protic solvents such as methanol, vibrationally hot molecules typically dissipate excess vibrational energy to the solvent on a timescale of ~10–20 ps. Our kinetic analysis reveals that *cis*-azo is formed with a time constant of 14 ± 3 ps. This timescale places the formation of *cis*-azo in a regime where substantial vibrational cooling is expected to have already occurred or is occurring rapidly. Consistent with this, we do not observe time-dependent features in the experimental data characteristic of vibrational cooling. Considering the functional form of the Debye-Waller factor ($e^{-\sigma^2 q^2/2}$, equation (S17 in Supplementary Methods), vibrational cooling would manifest as distinct intensity changes, particularly in the high-q region. However, such features are not observed in the experimental data after ~10 ps, supporting the conclusion that *cis*-azo is largely thermalized upon formation. To verify this assumption, we additionally tested treating σ^2 for *cis*-azo as a free fitting parameter. The optimization yielded $\sigma^2 \approx 0.010$ Å^2, with a modest χ^2 improvement ($\Delta\chi^2 \approx 32$ out of a total $\chi^2 \approx 512$, corresponding to ~6%). The fitted value itself is not large. Combining the kinetic expectation of vibrational

cooling prior to observation with the small magnitude of the optimized σ^2, we fixed $\sigma^2 = 0$ for *cis*-azo in the final structural refinement. We have revised the relevant paragraph in Supplementary Method 6 as follows:

"In the structural refinement, the σ^2 value for ground-state trans-azo was fixed at 0. For cis-azo, σ^2 was also fixed at 0, as the late-forming product (τ = 14 ± 3 ps) has sufficient time for vibrational cooling prior to observation. In contrast, the refinement yielded σ^2 values of 0.018 ± 0.003 Å^2 for I_1 and 0.010 ± 0.003 Å^2 for I_2, indicating modest vibrational broadening in these intermediates. By explicitly incorporating the DWF, our analysis effectively decouples the vibrational effects from the structural changes. In this framework, the refined structural parameters define the average molecular geometry, whereas the σ^2 parameters capture the width of the vibrational ensemble around that center. Therefore, the presence of vibrational broadening does not invalidate the identified parsimonious motions; rather, by explicitly accounting for it through the σ2 parameter, the framework ensures that the refined structural parameters isolate the mean molecular geometry from the vibrational envelope surrounding it."

Molecular dynamics (MD) simulations for simulating cage terms

The authors state that "The force field parameters and atomic charges for the solvent molecules were adopted from previous studies." Could the authors provide more detail on these parameters or specify which previous studies were used as sources?

→ We appreciate the referee for this request. In the revised manuscript, the MD simulation methodology has been substantially updated to improve the accuracy and reproducibility of the cage-term calculations. Specifically, we employed the OPLS-AA force field with a six-site all-atom (AA) model for methanol. To ensure that the original source of these parameters is accurately identified, we have included the citation for the original study detailing this force field (*ACS Omega*, **2018**, *3*, 17566.), in addition to the recent application of this model (*Nat. Commun.* **2022**, *13*, 522.) which was already cited in the original version of the manuscript. The key changes from the original methodology are as follows: (i) The simulation software was changed from MOLDY to GROMACS, enabling more rigorous treatment of long-range interactions via Particle Mesh Ewald electrostatics and NPT ensemble simulations with the C-rescale barostat. (ii) The force field for the solute was changed from UFF to OPLS-AA, ensuring consistency with the solvent force field. (iii) Atomic partial charges were computed via natural population analysis (NPA) at state-specific levels of electronic structure theory (B3LYP for ground-state species, SS-CASSCF for excited-state intermediates), replacing the previous NBO analysis (Supplementary Method 8). (iv) The system size was increased from 512 to ~1,995 solvent molecules. (v) The sampling was extended from a single 1 ns trajectory to 30 independent 1 ns production runs per species, yielding 30 ns of cumulative sampling and enabling robust convergence assessment. The complete details of the updated methodology are provided in Supplementary Methods 8 and 9.

The authors state that they used the refined molecular structures of the I1 and I2 intermediates for natural bond orbital analysis. Could the authors clarify what is meant by "refined molecular structures"? Does this refer to experimentally refined structures? If so, could the authors explain why experimentally refined structures were used in the simulations, which were presumably intended to enable the fitting of the experimental data?

→ We thank the referee for this question. We clarify the terminology and rationale below.

The term "refined molecular structures" refers to the TRXL-refined geometries of I_1 and I_2, which were obtained by fitting the experimental scattering data as described in the manuscript. These structures serve two purposes in the MD simulations: (i) they provide the input geometries for computing state-specific atomic partial charges via natural population analysis (NPA), and (ii) they define the fixed solute geometry in the MD simulations. The resulting NPA charges and solute geometry together parametrise the MD simulations, from which the solute–solvent pair distribution functions (and hence the cage terms) are extracted. To ensure that

this procedure involves no logical circularity, we employed a self-consistent iterative framework. In the initial iteration, the MD simulations were performed using the CASPT2-optimized structures and NPA charges computed for those geometries. The resulting cage terms were used in the TRXL structural refinement to yield updated solute geometries. In subsequent iterations, NPA charges were recomputed on the refined structures, and the MD simulations, cage-term calculations, and structural refinement were repeated until convergence was achieved. This iterative approach explicitly accounts for the interdependence between the solute structure, atomic charges, and solvent cage organisation, and the observed convergence confirms that the final geometry and its corresponding cage term define a self-consistent result.

We note that, as part of this revision, the MD simulation methodology has been substantially improved: the simulations are now performed using GROMACS with the OPLS-AA force field for both solute and solvent (replacing the previously used MOLDY with the UFF force field for the solute), NPA charges replace the previously used NBO charges, and the system size and sampling have been increased to ~12,000 atoms and 30 ns cumulative sampling per species. These improvements, together with the self-consistent iterative framework, are described in detail in the revised Supplementary Methods 8 and 9.

Validating the kinetic model through linear combination fitting analysis

The authors validate the kinetic model through linear combination fitting and conclude that it accurately describes the structural dynamics. Given that this validation relies on SADS derived from the same kinetic model, could the authors comment on whether it is expected that the model could not be recovered? How do they ensure that this does not introduce circular reasoning?

→ We appreciate the referee for raising this important methodological question. The SADSs are indeed extracted using the kinetic model, and the linear combination fitting (LCF) analysis uses these SADS as input. However, the LCF serves as a critical internal-consistency check, precisely because the two steps constrain different aspects of the decomposition. In KCA, the population dynamics are strictly fixed according to the kinetic model (rate equations), and the algorithm extracts the SADS that best reproduce the experimental data. In LCF, the SADS are fixed, and the population coefficients are optimized independently at each time delay with no kinetic constraints and no coupling between successive time points. The LCF thus yields 137 × 3 = 411 independently determined coefficients (three species at each of 137 time delays). These coefficients are then compared with the population profiles predicted by the kinetic model, whose time constants were independently determined from the global fit of the RSVs (Supplementary Method 1). Because the kinetic model predictions and the LCF coefficients are derived through entirely separate procedures, one from the time-domain RSV fitting, the other from unconstrained decomposition at each time delay, their agreement constitutes a non-trivial validation. If the kinetic model were incorrect, the KCA-extracted SADS would be distorted to compensate for the incorrectly assumed population dynamics. When these distorted SADS are used in LCF, the independently determined coefficients would systematically deviate from the kinetic model predictions. For example, if the true mechanism involved parallel pathways (A→B and A→C) but was incorrectly modelled as sequential (A→B→C), the LCF-derived concentration profiles would show unphysical features such as negative populations or premature appearance of species. Similarly, if the model assumed an incorrect time constant, the LCF coefficients would show systematic time-dependent deviations from the kinetic model predictions. The excellent agreement observed in our data (Fig. 3e) confirms that our kinetic model accurately captures the temporal evolution of the species without forcing the data into an incompatible framework.

Supplementary Discussion

2 | Analysis of the anisotropic scattering signal

The authors state that "I2 and cis-azo do not contribute detectable anisotropy signals. This, however, does not necessarily mean the absence of anisotropy in I2 and cis-azo. Rather, it may be that the substantial rotational motions accompanying the I1 → I2 transition (ROT + OHT) lead to rapid rotational dephasing, reducing their anisotropy below the detection limit, since anisotropy signals arise from the initial alignment of molecules before rotational motions randomize their orientation." This point is somewhat unclear. How does the rapid rotational dephasing and reduction of anisotropy below the detection limit differ from the absence of anisotropy in I2 and cis-azo? Could the authors clarify the distinction between these two scenarios?

→ We thank the referee for raising the question of anisotropic scattering contributions. In the revised manuscript, we have added a dedicated analysis of the anisotropic scattering signal in Supplementary Note 2. SVD analysis of the anisotropic difference scattering data reveals only a single significant component, which is attributed to the photoselective ground-state depletion (hole) of *trans*-azo and the structurally similar I_1 intermediate. Notably, no additional anisotropy component is detected from I_2 or *cis*-azo, despite their distinct molecular geometries. We attribute this absence to the large-amplitude ROT motion that dominates the $I_1 \rightarrow I_2$ transition (70% by motional contribution analysis), which is expected to randomize the molecular orientation of I_2 upon its formation. Since the structural refinement is performed on the isotropic component of the difference scattering signal, which is separated from the anisotropic component during data reduction (Supplementary Fig. 1), the anisotropic contributions do not affect the extracted SADS or the structural conclusions of this study.

3 | Systematic Scan for Parsimonious Motions (SSPM) to characterize structural motions

The authors refer to "conventional SADS analyses." Could the authors consider adding a reference to a study where such a conventional SADS analysis was performed, to provide context for readers?

→ We appreciate the referee for this suggestion. To provide appropriate context for the "conventional SADS analysis," we have added references to representative studies (*Chem. Sci.*, **2021**, *12*, 2114–2120; *J. Am. Chem. Soc.*, **2023**, *145*, 23715–23726; *Chem. Commun.*, **2022**, *58*, 7380–7383.), where such analysis was performed. These studies demonstrate the standard approach where SADS are extracted based on kinetic models, and the subsequent structural analysis is performed without considering specific parsimonious motions as employed in our SSPM method.

The authors state that they "could identify the smallest set of motions that yielded a sufficiently low χ^2 value". Could the authors define what threshold or range they consider sufficiently low, and explain how this choice was justified?

→ We acknowledge that the original criterion ("sufficiently low χ^2") lacks rigorous statistical justification. To address this in the revision, we have implemented the BIC analysis to essentially identify the smallest set of motions that satisfactorily describe the data, as detailed in our responses to the relevant comments. This statistical test provides an objective criterion for determining the necessary number of basis motions, ensuring that we do not overfit or underfit the experimental data. The detailed BIC analysis results are described in our response to the comment on Extended Data Fig. 6 above.

Out of curiosity, based on the analysis presented, would it be possible to estimate the relative contributions of individual mechanisms to the overall motion in cases where two or more mechanisms are involved? Could the authors comment on whether such a decomposition is feasible within their current framework?

→ We thank the referee for this stimulating question. Yes, such a quantitative decomposition is fully feasible within our framework. To address this, we developed a new analysis method termed Motional Contribution Analysis (MoCA), which evaluates the relative contribution of each basis motion using a leave-one-out (LOO) approach. Specifically, for a structural transition described by a set of *n* basis motions, MoCA measures the

increase in the optimized RMSD when each individual motion is removed from the combination while re-optimizing the remaining motions. The fractional LOO contribution of each motion then quantifies its functional necessity within the assigned combination. The detailed methodology is described in Supplementary Method 15.

Applying MoCA to the TRXL-refined structures yields the following decomposition: 100% CNT for the *trans*-azo → I_1 transition, 70% ROT + 30% CNT for I_1 → I_2, and 53% ROT + 40% INV + 7% CNT for I_2 → *cis*-azo (Supplementary Note 4; Supplementary Table 5). These results provide a quantitative picture of how the dominant motion evolves along the isomerization pathway.

4 | Basis-Motion Analysis (BMA) to unveil the motions responsible for structural transitions and 5 | Discussion on the basis motions analyzed by BMA

The authors use BMA to evaluate how well a given motion applied to structure A can reproduce structure B and to identify which motions drive the observed structural transitions. For the TRXL-refined geometries, which were optimized using these motions, could the authors comment on whether it is expected that the same motions appear prominently in the BMA analysis? How do they ensure that this observation is not simply a consequence of the refinement procedure?

→ Yes, as the referee correctly noted, it is expected by construction that the same motions appear prominently in the BMA analysis for the *trans*-azo → I_1 and I_1 → I_2 transitions, because the TRXL-refined structures were optimized within the subspace defined by the SSPM-selected motions. For these transitions, the primary value of BMA lies not in identifying which motions are involved, but in quantifying their relative contributions through MoCA (e.g., 70% ROT + 30% CNT for I_1 → I_2), which SSPM alone does not provide. In contrast, since SSPM was not applied to $SADS_3$ (*cis*-azo), the motions responsible for the I_2 → *cis*-azo transition were determined independently through BMA and MoCA. Furthermore, the same BMA analysis was applied to the CASPT2-calculated structures, which were obtained entirely independently of the SSPM refinement. The comparison between the two sets of BMA results (Supplementary Note 4; Supplementary Table 5) therefore provides a genuinely independent assessment of how the motional composition of the isomerization pathway differs between experiment and theory.

The authors report an RMSD of 0.244 Å for I1 to I2 for the CASPT2 results, but also that the largest RMSD among these steps is around 0.03 Å. The authors should verify that the reported values are correct.

→ We thank the referee for the careful reading. The inconsistency between the reported RMSD of 0.244 Å for the I_1 → I_2 step and the statement "the largest RMSD remains small (~0.03 Å)" was indeed an error in the previous version. In the revised manuscript, the BMA analysis has been substantially revised: the OHT (out-of-plane hydrogen twist) basis motion has been removed, and CNT (C–N bond torsion) has been newly included. BIC-based model selection has also been applied to objectively determine the optimal motion combination for each step (Supplementary Note 4). With the revised analysis, all CASPT2 BMA residuals are now ≤ 0.03 Å (0.0283, 0.0073, and 0.0262 Å for the three steps, respectively), and the inconsistency no longer exists.

6 | Comparison between CASPT2 and previously reported theoretical structures

Could the authors include the resulting S_1 potential energy surface from the CASPT2 calculations? Visualizing this surface would help readers understand the energetic landscape.

→ We have included the CASPT2 S_1 potential energy surface in Supplementary Information (Supplementary Fig. 7). The potential energy surfaces were computed as reduced-dimensional scans along the CNNC dihedral angle and the CNN angles, with the scanned coordinates varied in a rigid manner. These coordinates provide a clear and intuitive representation of the photoisomerization pathway, allowing straightforward identification of

the *trans* minimum, the two S_1 minima (I_1 and I_2) discussed in the text and reoptimized at the full CASPT2 level, as well as the approximate location of the *cis* minimum. The description of this calculation has been added in the "*Ab initio* calculations" section with the following description.

"Prior to geometry optimizations, the underlying energetic landscape of the S_0 and S_1 states was explored by constructing two-dimensional potential energy surfaces obtained from rigid scans along the CNNC dihedral angle and the symmetric CNN bending coordinate, while keeping all remaining internal coordinates fixed (Supplementary Fig. 7). These coordinates provide a physically transparent description of the photoisomerization pathway and allow straightforward identification of the *cis* and *trans* minima, as well as local minima on the S_1 surface."

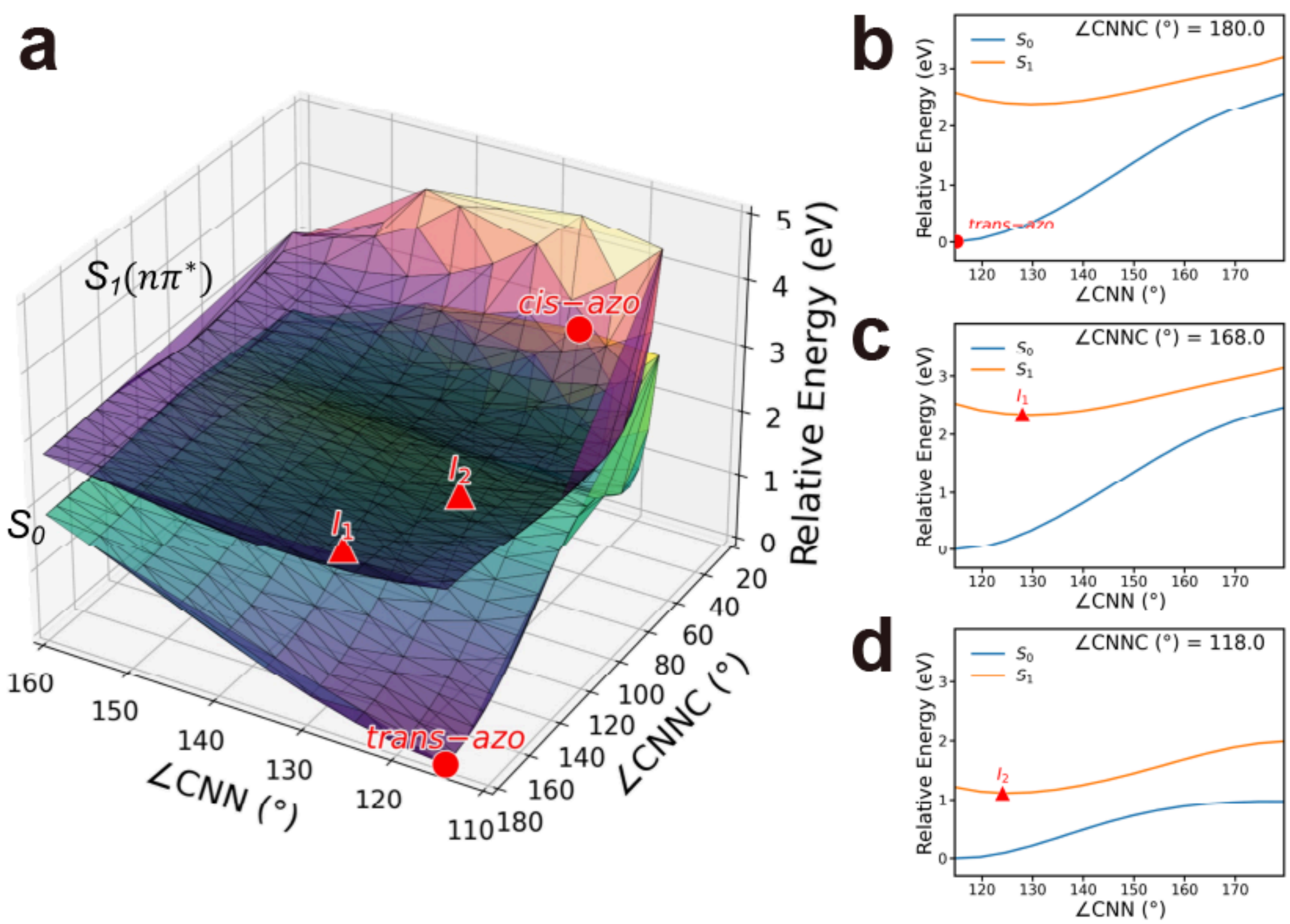

Supplementary Fig. 7 | CASPT2 potential energy surfaces of azobenzene along CNNC and CNN angles. Two-dimensional potential energy surfaces (PESs) of azobenzene computed at the XMS-CASPT2 level for the ground (S_0) and first excited (S_1, nπ*) electronic states. **a,** Three-dimensional side view of the PESs obtained from rigid scans along the CNNC dihedral angle and the symmetric CNN angle, with all remaining degrees of freedom kept fixed. This representation enables a direct visualization of the *trans*-azo minimum on the S_0 surface and of the two S_1 (nπ*) minima, I_1 and I_2. The *cis*-azo minimum cannot be reached within the present rigid scans, which do not allow concerted rotation of the phenyl rings; the corresponding angular region is nevertheless indicated for completeness. Red filled circles mark optimized ground-state stationary points, while red filled triangles denote the I_1 and I_2 minima on the S_1 surface. **b–d,** One-dimensional cuts of the two-dimensional PESs along the CNN bending angle at fixed CNNC dihedral angle, corresponding to the *trans*-azo ground-state minimum (**b**), I_1 (**c**), and I_2 (**d**) minima on the S_1 surface.

9 | Comparison of the results from spectroscopy studies and TRXL results

The authors state that "It is important to note that the assignments made by previous studies are not entirely incorrect; rather, the structural dynamics we observed suggest that the trans-to-cis isomerization of azobenzene involves contributions from both out-of-plane hula-twist and rotation." Could the authors expand on this statement and elaborate on what aspects of the previous studies they consider correct and which aspects they believe require revision or clarification?

→ We have expanded the discussion to explicitly state which spectroscopic assignments align with our findings and where they differ, providing a unified mechanistic picture. We clarified that while previous assignments of "rotation" or "inversion" might seem contradictory, they likely captured specific components of the complex coupled motion we observed. For instance, studies supporting "rotation" likely detected the large ∠CNNC change, which is indeed a major component of our observed pathway. Studies supporting "inversion" may have been sensitive to the ∠CNN changes that contribute to the later stage of the isomerization (I_2 → *cis*-azo, where INV accounts for 40% of the motional contribution), rather than the early steps. Crucially, none of the previous studies identified CNT as a distinct early-stage motion, which our TRXL data reveal to be the dominant coordinate in the *trans*-azo → I_1 step. This represents the principal aspect requiring revision in the existing mechanistic picture. Our TRXL results reconcile these views by revealing that the actual mechanism involves a sequential interplay of CNT and ROT, with INV becoming relevant only in the final step. We added the following discussion to the revised "Comparison of observed isomerization pathway with spectroscopic assignments" section (now Supplementary Note 8).

"It is important to note that the assignments made by previous studies are not entirely incorrect. Our structural analysis suggests that these seemingly conflicting assignments rather represent partial views of the full reaction coordinate. Specifically, studies that assigned the mechanism to "rotation" likely detected the large rotational motion associated with the ∠CNNC change, which is indeed a major component of our observed pathway. Similarly, studies supporting the "inversion" mechanism may have been sensitive to the ∠CNN changes that contribute to the later stage of the isomerization (I_2 → *cis*-azo, where INV accounts for 40% of the motional contribution), rather than the early steps. The structural dynamics we observed suggest that the *trans*-to-*cis* isomerization of azobenzene is not solely driven by rotation but also involves significant contributions from C–N torsion associated with ∠CCNN changes, particularly in the initial step toward I_1, whereas changes in ∠CNN are not apparent in the early steps (*trans*-azo → I_1 and I_1 → I_2)."

Supplementary Figure

Supplementary Fig. 1

The caption does not agree with the reported results or the caption in Fig. 4.

→ We thank the referee for identifying this inconsistency. In the revised manuscript, we have removed the original Supplementary Fig. 1, as its utility was highly limited. In its place, a new Supplementary Fig. 1 has been added, which illustrates the data reduction pipeline (see our response to the "Data processing and analysis" comment below).

General comments and questions

The authors should ensure that the axes on their figures are accurate, labels on the y-axis appear shifted in multiple figures.

→ We thank the referee for this observation. To ensure clarity and avoid any potential rendering issues, we have re-exported all figures for the revised manuscript and confirmed that all axis labels are properly positioned.

Cross-references between sections and to figures should be checked for correctness.

→ We thank the referee for this suggestion. We have thoroughly reviewed all cross-references throughout the manuscript and Supplementary Information and confirmed their accuracy. Any incorrect references have been corrected in the revised version. Several incorrect figure/section references have been identified and corrected. Some examples are as follows.

"Based on the kinetic model (shown in Extended Data Fig. 3d,e)" → "Based on the kinetic model (shown in Fig. 3d,e)"

"Based on the kinetic model (shown in Extended Data Fig. 3d,e), $m_i(t)$ can be expressed as follows" → "Based on the kinetic model (shown in Fig. 3d,e), $m_i(t)$ can be expressed as follows"

The manuscript and Supplementary Information refer to PEPC-treated data and solvent-contribution-free data. Are these distinct or equivalent? This should be clarified.

→ We thank the referee for pointing out this potential confusion. These terms refer to the same data. "PEPC-treated data" and "solvent-contribution-free data" both describe the TRXL data after applying the projection to extract the perpendicular component (PEPC) procedure, which projects the data onto a subspace orthogonal to the solvent response, thereby isolating the solvent-independent photoinduced contributions. To improve clarity and accuracy, we have replaced the term "solvent-contribution-free" with "solvent-independent" throughout the revised manuscript. We now consistently use "PEPC-treated data" or "solvent-independent TRXL data" to refer to the processed data. The terminology has been clarified in the Methods section as follows.

"The solvent contributions were effectively screened out from the original TRXL data (Extended Data Fig. 1) to obtain the PEPC-treated data presented in Extended Data Fig. 2, which we refer to as the solvent-independent data. The term "solvent-independent data" is equivalent to the term "solvent-contribution-free data" used in a previous study[64]; both describe TRXL data that has been projected into a subspace orthogonal to the solvent response."

While the structural investigation of different motion mechanisms is intriguing, do the authors anticipate that future TRXL studies of organic molecules could track structural changes directly in the time-resolved data, rather than relying on systematic scans for parsimonious motions and basis-motion analysis to identify structural transitions?

→ We appreciate this forward-looking question. At present, the signal-to-noise ratio (SNR) of TRXL data imposes practical limitations on how much structural information can be uniquely extracted from the SADS without additional constraints. Moreover, X-ray solution scattering inherently encodes one-dimensional pair distance distributions, so that recovering three-dimensional molecular geometries inevitably requires some degree of structural modelling, regardless of data quality. SSPM addresses both limitations by systematically evaluating well-defined structural coordinates, thereby reducing parameter degeneracy and improving the robustness of structural refinement. Looking forward, with advances in X-ray sources (e.g., high-repetition-rate XFELs) and detectors, the improved SNR and time resolution may enable more direct extraction of structural parameters from time-resolved scattering data with fewer model assumptions. For example, our BIC-based model selection identifies CNT alone as the statistically optimal description of the *trans*-azo → I_1 transition, although the actual motion may involve minor contributions from other coordinates (e.g., ROT) that cannot be confidently resolved with the current data quality. With improved SNR, the BIC framework would become sensitive to these smaller contributions, enabling finer decomposition of the motional landscape while retaining the statistical rigour that prevents over-interpretation. However, we note that SSPM will likely remain valuable even in such regimes because it provides physically interpretable reaction coordinates (e.g., rotation vs. inversion), which are crucial for connecting structural dynamics to mechanistic understanding. Direct

parameter extraction would tell us which atoms moved where, but SSPM tells us which mechanism drove the motion.

References

The references are generally appropriate; however, in several sections of the Methods and Supplementary Information, the authors refer to "established" or "well-established" methods without providing adequate citations. In some cases, only one or two references are given, often self-citations. For methods described as established, multiple references from different research groups should be included to substantiate this claim. Additionally, the authors should provide direct citations to the relevant studies in Extended Data Tables 1 and 2 to facilitate access for readers interested in these sources.

→ We thank the referee for this important observation. We have thoroughly reviewed all instances where we used the terms "established" or "well-established" in the Methods and Supplementary Information sections. Where only one or two citations (particularly self-citations) were provided, we have expanded the references to include multiple independent studies from different research groups, ensuring that the characterization as "established" is properly substantiated. Additionally, we have revised Extended Data Tables 1 and 2 to include direct citations to all relevant studies, making it easier for readers to access the original sources. In total, a substantial number of new references from independent research groups have been added throughout the revised manuscript and Supplementary Information to substantiate the methodological claims.

Responses to the Comments from Referee #3

I co-reviewed this manuscript with one of the reviewers who provided the listed reports.

→ We sincerely thank the reviewer for their thoughtful and constructive report. We have addressed the comments raised by Referee #2 in our responses above.

Responses to the Comments from Referee #1

I have sent these comments to the editorial office. Two of my students spent a considerable amount of time testing the code and checking the materials uploaded. The authors should upload the average raw diffraction data (one diffraction pattern per time delay + reference) and their complete workflow for reproducibility checks.

→ We are grateful to the referee and to the two students for the careful and substantial effort they spent testing our code and materials; their feedback has directly improved the reproducibility of our deposit. We have addressed both requests and, beyond them, have organized the deposit so that the workflow can be reproduced at three levels of detail.

(i) Two-dimensional detector images. The complete set is approximately 3.2 TB and cannot be hosted publicly, so it is available upon reasonable request. So that the conversion from two-dimensional images to one-dimensional curves can itself be demonstrated and tested, we have included a representative real detector image together with the calibration geometry, the detector mask, and the integration script (pyFAI azimuthal integrate2d (azimuthal regrouping), from which the isotropic and anisotropic components are obtained by a Legendre P_0/P_2 decomposition). This 2D-to-1D conversion is applied identically to every image, whether laser-on or laser-off; the laser-on/off distinction enters only at the subsequent per-delay pairing-and-averaging step described in (ii).

(ii) Single-shot and per-run one-dimensional curves. For one representative run, we have deposited all single-shot isotropic and anisotropic curves, so that the laser-on/off pairing, the area-under-curve normalization, and the outlier rejection that produce that run's averaged curves can be reproduced. For every run, we have additionally deposited the per-run averaged curves at each time delay, so that the subsequent averaging across runs, which yields the final averaged data used in the analysis, can likewise be reproduced.

(iii) Averaged one-dimensional curves obtained by averaging all images from multiple runs via steps (i) and (ii), which serve as the reproducible entry point: one curve per time delay plus the neat-solvent reference (the averaged isotropic and anisotropic difference-scattering curves for both the azobenzene solution and the neat solvent). From these curves onward, the entire analysis, including the PEPC treatment, the SVD and kinetic fitting, and the structural analysis, is reproducible with the deposited code. These averaged data were also provided as Source Data with our previous submission: the raw (before-PEPC) averaged curves as the Source Data for Extended Data Figure 1, the corresponding PEPC-treated curves as the Source Data for Extended Data Figure 2, and the decomposed solute, cage, and solvent curves as the Source Data for Figure 2.

The specific code modules that process the data at each of these stages are described in our responses to the points below. We have also revised the "Data processing" section of the Methods to be fully explicit, so that each step is described in the text and corresponds to the deposited code: the pooling of adjacent time delays and the resulting analysis time grid, the area-under-the-curve normalization and outlier-rejection ranges, and the determination of the time-zero offset and the instrument response function from the anisotropic signal.

Major Complaints

We are lacking raw experimental data and a significant part of the pipeline. In Methods of the paper they talk about transforming raw data into a specific cleaned state so that analysis can be done. The code provided only provides structural analysis, for SSPM and BMA, and nothing else. While the code as given runs without major issues, it is hard to judge properly without the full pipeline and raw experimental data.

For example, I would like to see the PEPC implementation, in particular. … Given that this is the first time they are using it for experimental data and it is such a powerful and novel method, I would like to see how the code works. … I believe it should be listed prominently given it is a significant part of the preprocessing.

Similarly for PCA, SVD, solvent cage work that was done with MD, etc. We are missing all code and experimental from before the techniques described in sections Data processing and Obtaining solvent-independent TRXL data.

→ We address these three related points together, as they all concern the completeness of the deposited pipeline. We have substantially expanded our Code Ocean capsule so that, in addition to the previously deposited structural (SSPM/BMA/MoCA) analysis, it now contains the complete data-processing and analysis workflow, from the raw detector images through to the structural analysis. The averaged one-dimensional curves serve as the reproducible entry point for the downstream analysis, and the capsule additionally documents the upstream front-end that produces those curves from the raw detector images. The capsule provides the following components, each in its own directory (the directory name is given in parentheses):

(i) Preprocessing, raw images to one-dimensional curves (00_preprocessing/): a documented, runnable example of the image-to-curve front-end (detector-image correction; pyFAI integrate2d, giving the mean intensity in bins of q and azimuthal angle; and, at each q, a Legendre P_0/P_2 decomposition over the azimuthal angle into isotropic and anisotropic curves), applied to a representative detector image (the same conversion applies to every image, laser-on or laser-off). This is the stage described in the "Data processing" section of the Methods.

(ii) PEPC (01_pepc/): PEPC, which projects the scattering data onto a subspace orthogonal to the solvent response to obtain the solvent-independent component, is now provided as a dedicated, documented module (pepc_v2.m), together with a short runnable example (example_pepc.m) that applies it to the deposited experimental data and isolates the solvent-independent component, which is far weaker than the dominant solvent contribution. In the previous capsule this projection was embedded in a large file of general-purpose utility functions and was therefore hard to find; it has now been extracted into this clearly labeled, self-contained module.

(iii) Run-merging, PEPC, SVD, and kinetic analysis (02_svd_kinetics/): the run-averaging that combines the per-run curves, the PEPC treatment applied once to the run-averaged data (the same projection provided as a standalone module above), the singular value decomposition, and the global multi-exponential, IRF-convolved kinetic fitting that yields the reported time constants. A single driver (run_pipeline.m) runs the full sequence and reproduces the reported time constants from the deposited per-run isotropic and anisotropic difference curves.

(iv) MD solvent-cage (03_md_cage/): the GROMACS inputs for the azobenzene-in-methanol simulation and, as the runnable entry point, the script (cage/compute_cage_5term.py) that computes the cage term from the solute–solvent radial distribution functions using 5-term Waasmaier-Kirfel X-ray form factors. The MD uses the solute partial charges obtained by natural population analysis in 04_orca_npa/, computed with the SMD(methanol) implicit-solvation model.

(v) ORCA / NPA solute charges (04_orca_npa/): the ORCA input files for the excited-state intermediates I_1 and I_2, computed at the SS-CASSCF(10,8)/aug-cc-pVDZ level with the SMD(methanol) implicit-solvation model, and the resulting NPA partial charges (extracted with JANPA from the SC-NEVPT2 density evaluated on top of the SS-CASSCF reference) used as the solute charges in the MD. The ground-state atomic partial charges of *trans*-azobenzene and *cis*-azobenzene, computed at the B3LYP-D3BJ/def2-TZVP level with the same solvation model, are also provided. (ORCA and JANPA are external packages and are not redistributed here.)

(vi) Structural analysis (05_sspm_bma/): the SSPM/BMA/MoCA code, now accompanied by the profile-likelihood scripts that compute the reported parameter uncertainties (which were not part of the previous capsule and have been added in response to Referee 1's comment) and a public manifest (see the minor points below).

Together, these modules cover the full workflow, from the raw detector images through to the structural analysis, addressing the gap that Referee 1 identified. The upstream image-to-curve front-end is demonstrated on a representative real detector image, because the complete raw 2D image set (approximately 3.2 TB) cannot be hosted publicly and is available upon request; the downstream analysis is fully reproducible from the deposited averaged one-dimensional curves, which serve as the reproducible entry point.

Minor Complaints

The capsule release does not clearly identify which specific scripts, outputs, and shipped artifacts correspond to each manuscript-facing result. In particular, the package exposes multiple overlapping BMA/MoCA output paths and iteration-labeled structure/cage files without a concise public manifest linking them to the reported tables and figures.

→ We have added a public manifest (MANIFEST.md) that maps each deposited script, data file, and generated output to the specific manuscript item it produces (the Fig. 3f SADS, Extended Data Table 3, Extended Data Figs 6 and 7, Supplementary Tables 2–7, and the reported time constants). Overlapping or intermediate BMA/MoCA outputs have been moved to a clearly marked intermediate/ folder, and every structure and cage file is labeled with its iteration in the manifest.

From the released materials alone, I could not find the code corresponding to the profile-likelihood uncertainty procedure described in Supplementary Method 4. The only visible structural-uncertainty routine is a commented-out Hessian-based helper path, which is a local quadratic approximation rather than the parameter-fixing/re-optimization profile-likelihood procedure described in the supplement.

→ We thank the referee for pointing out that the profile-likelihood code was missing from the deposited capsule, which retained only the secondary Hessian-based helper. This was an inadvertent omission, which we have corrected: we have now added the profile-likelihood scripts that produced the reported uncertainties (one for each refined species: profile_likelihood_I1_v2.m, profile_likelihood_I2.m, and profile_likelihood_cis.m), implementing exactly the parameter-fixing-and-re-optimization procedure ($\Delta\chi^2 = 1$) described in Supplementary Method 4. The Hessian-based routine is retained only as an optional fast cross-check and is not the source of the reported uncertainties.

The released I1 to It SSPM script does not clearly separate the unconstrained I2 parameterization from the symmetry-reduced BIC accounting. As a result, the public code can present an I2 fit with independent structural parameters while still applying a symmetry-reduced parameter count, which makes the manuscript-facing I2 comparison harder to audit.

→ We thank the referee for this careful check, and we agree that in the previous capsule this symmetry setting was hard to notice: it was controlled by a single flag whose default did not correspond to the configuration used for the SSPM analysis of the $I_1 \rightarrow I_2$ transition in the manuscript. To make it explicit, we have surfaced this as a clearly documented flag (useSymmetry) at the top of the script, and the BIC report now states, for every model, both the symmetry setting used and the active number of free parameters. We emphasize that the corresponding degrees of freedom were already applied consistently in our analysis, because this flag sets both the fit constraints and the BIC parameter count together. For the $I_1 \rightarrow I_2$ step, the I_2 structure was fit with independent parameters ($\angle CNN_1/\angle CNN_2$ and $\angle CCNN_1/\angle CCNN_2$ allowed to differ), so the BIC counts the

C–N torsion as two parameters and the selected ROT + CNT model has $k = 3$ structural parameters (ROT = 1, C–N torsion = 2); including the mean-square-displacement, scaling-coefficient, and the two baseline coefficients, this gives $d_f = k + 4 = 7$ free parameters ($\chi^2 = 168.5$). For the *trans* → I_1 step, performed under C_i symmetry, the C–N torsion counts as a single parameter and the selected CNT model has $k = 1$ and $d_f = k + 4 = 5$ ($\chi^2 = 123.1$). By toggling this flag, the reader can now run either the symmetry-constrained fit or the unconstrained (independent-parameter) fit and see the corresponding parameter count and BIC directly.

A bit nitpicky, but their label system as written in README.txt does not correspond to their code. They state that they have used iteration 5 for their results, but their provided data files for structural information are not iteration 5. We have an iteration 1, two iteration 4s for the intermediaries, etc. It's unclear if these files are not up-to-date or if the readme is wrong or miswritten.
→ We thank the referee for noting this inconsistency in our file labeling. It arose from a blanket "iteration 5" statement in the README, compounded by a zero-indexed numbering scheme (in which "iteration n" denotes the $(n + 1)$th refinement round) that was easy to misread. We have reconciled the README, the deposited structure and cage files, and the manuscript results so that each species is represented by its true converged iteration, stated under a clearly defined numbering convention and labeled accordingly in the manifest; stale intermediate files have been removed.

Responses to the Comments from Referee #2

The revised manuscript X-ray liquidography decodes complex motions in azobenzene isomerization by Kim et al. is greatly improved in terms of clarity, consistency, and overall presentation. My previous comments and suggestions have been addressed satisfactorily, and I now recommend acceptance for publication in Nature after the following minor issues have been addressed.
→ We thank the referee for the positive assessment and the recommendation to accept. We have addressed each of the remaining minor points below.

Requests for clarification
Supplementary Information, page 12 (baseline correction):
The authors state that the baseline correction parameters a_i and b_i were treated as free parameters and optimized simultaneously with the solute structural parameters. However, in reporting the number of free parameters, only the structural motion parameters and two additional parameters (mean-square displacement and scaling coefficient) are included. Could the authors clarify why the baseline correction parameters are not counted among the free parameters?
→ We are grateful to the referee for spotting this subtle, easily overlooked point. The referee is correct. When we reported the number of free parameters, both in Supplementary Method 3 (Eq. S13) and in the definition of the BIC, we did not include the two baseline coefficients a_n and b_n that appear in the term $B_n(q)$ (Eq. S12). These two coefficients are optimized during the fit, in exactly the same way as the mean-square-displacement parameter and the scaling coefficient, and so they should be counted among the free parameters. We have corrected the omission.

Within a single model, all candidate structures have the same number of free parameters. Therefore, accounting for the two baseline coefficients changes the reduced chi-square values uniformly and does not affect the refined structure. Between models, selection is based on the BIC. Because the two baseline coefficients are common to all models, they add the same constant to every BIC value and therefore do not affect model selection or any of the fitting results.

Beyond this shift caused by counting the two baseline coefficients described above, the reported reduced chi-square and BIC values differ slightly from those in the previous version (for example, the reduced chi-square for the TRXL-refined *cis*-AB structure changed from 1.01 to 0.97; all key parameters are summarized in a table in the "Additional Changes" section of this point-by-point response). These differences arise not from parameter counting but from improvements in the propagation of experimental uncertainties, as described in the "Data processing" section of the Methods. The revised texts are as follows:
Supplementary Method 3 (definition of d_f below Eq. S13).
Original: "Here, $n_q = 511$ is the number of q-points and $d_f = k + 2$ is the number of free parameters (k is the number of structural motion parameters and the two additional parameters account for the mean-square displacement parameter σ^2 and the scaling coefficient)."
Revised: "Here, $n_q = 511$ is the number of q-points and $d_{f,n}$ is the number of free parameters in the fit to the n-th SADS: the k structural motion parameters, the scaling coefficient, the mean-square-displacement parameter σ^2 where it is refined, and the two baseline coefficients a_n and b_n of $B_n(q)$. For the SSPM fits of I_1 and I_2, $d_{f,n} = k + 4$; for the refinement of the *cis*-AB structure, in which σ^2 is fixed, $d_{f,n} = k + 3$. For a given motion combination, $d_{f,n}$ enters the reduced chi-square only as a constant normalization, so the refined structure is independent of this count."

Methods, main text (model selection).
Original: "Model selection was performed using a χ^2-based Bayesian Information Criterion (BIC), defined as $\mathrm{BIC}(\chi^2) = \chi^2 + (k + 2) \cdot \ln(N_{eff})$, where k is the number of structural motion parameters and the two additional parameters account for the mean-square displacement parameter σ^2 which accounts for thermal broadening of interatomic distances and the scaling coefficient, …"
Revised: "Model selection was performed using a χ^2-based BIC, defined as $\mathrm{BIC}(\chi^2) = \chi^2 + (k + 4) \cdot \ln(N_{eff})$, where k is the number of structural motion parameters and the four additional parameters account for the mean-square displacement parameter σ^2 which accounts for thermal broadening of interatomic distances, the scaling coefficient, and the two baseline coefficients a_n and b_n, …"

Supplementary Method 13 (Eq. S30).
Original: "In the present SSPM analysis, the total number of model parameters is $k + 2$, where k is the number of active structural (motional) parameters and the two additional parameters are the σ^2 (the Debye–Waller mean-square displacement) and the scaling coefficient. Substituting this into the general BIC gives:

$$BIC_{\chi^2} = \chi^2 + (k + 2) \cdot \ln(N) \qquad \text{(S30).”}$$

Revised: "In the present SSPM analysis, the total number of model parameters is $k + 4$, where k is the number of active structural (motional) parameters and the four additional parameters are the σ^2 (the Debye–Waller mean-square displacement), the scaling coefficient, and the two baseline coefficients a_n and b_n. Substituting this into the general BIC gives:

$$BIC_{\chi^2} = \chi^2 + (k + 4) \cdot \ln(N) \qquad \text{(S30).”}$$

Methods section (lines 649–667) and Supplementary Method 13:
The authors note that some reduced chi-squared values are well below unity and attribute this to the effective number of independent information channels being smaller than the number of measured q-points. Would reducing the number of q-points (e.g., through binning along the q-axis) mitigate this issue?
→ We thank the referee for this insightful question. While binning along the q-axis would indeed raise the reduced chi-square toward unity by reducing the oversampling, we deliberately retained the full q-resolution.

As detailed in the Methods and Supplementary Method 13, sub-unity reduced chi-squared values are an inherent consequence of oversampling rather than an indication of overfitting. Due to the Shannon limit, the independent information content of solution scattering is significantly smaller than the number of measured *q*-points. We rigorously account for this by determining the effective number of independent points (N_{eff} = 110) via a BIC-based procedure and incorporating it directly into our model selection. Consequently, while binning would not alter any reported results or model-selection outcomes, it would discard the fine *q*-sampling required by our forward model and PEPC projection, and degrade the independent per-pixel error statistics. We therefore retained the full *q*-sampling.

Minor revision suggestions: Main Text

Lines 143–152: The authors may consider referring to a figure that visualizes the structural parameters of the four basic motion types (e.g., Fig. 4), to aid the reader.

→ We thank the referee for the helpful suggestion. At the point where the four basic structural motions are introduced (Main Text, lines 143–152), we now refer the reader to Fig. 4, which visualizes their structural parameters. The sentence is revised as follows:

Original: "To capture such flexibility, we represented these mechanisms using combinations of four basic structural motions: N–N rotation (ROT), inversion (INV), N–N bond-length change (NND), and C–N torsion (CNT)."

Revised: "To allow such flexibility, we represented these mechanisms using combinations of four basic structural motions, each defined by a characteristic structural parameter shown in Fig. 4a: N–N rotation (ROT), inversion (INV), N–N bond-length change (NND), and C–N torsion (CNT)."

Line 817 (Extended Data Fig. 1): Please verify the references to figure panels.

→ We are grateful to the referee for prompting us to check the panel references of Extended Data Fig. 1. The scaling note referred to "panels b and e," but panel e shows the azobenzene-solution anisotropic signal, not the neat solvent; the scaled neat-solvent panels are b (azimuthal) and d (isotropic). We have corrected the reference, as follows:

Original: "The neat solvent data in panels b and e were scaled so that the signal at 100 ps matches that of the azobenzene solution data, and the same scaling factor was applied to panel f."

Revised: "The neat solvent data in panels b and d were scaled so that the signal at 100 ps matches that of the azobenzene solution data, and the same scaling factor was applied to panel f."

Lines 874–875 (Extended Data Fig. 6): Ensure consistency between color descriptions and the figure.

Lines 890, 893 (Extended Data Fig. 7): Ensure consistency between color descriptions and the figure.

→ We are grateful to the referee for pointing out the color mismatch in these captions. In both figures, the right-axis quantity (χ^2 in Extended Data Fig. 6; RMSD in Extended Data Fig. 7) is plotted in green, the left-axis ΔBIC is plotted in black, the CASPT2 reference is a blue dashed line, and the BIC-optimal model is marked with a red circle. The original captions incorrectly described the left axis as red. We have corrected the color descriptions in both captions as follows:

Extended Data Fig. 6, Original: "…the right *y*-axis shows χ^2 values (black) for visual reference, and the left *y*-axis shows ΔBIC(χ^2) values (red)…"

Extended Data Fig. 6, Revised: "…the right *y*-axis shows χ^2 values (green) for visual reference, and the left *y*-axis shows ΔBIC(χ^2) values (black)…"

Extended Data Fig. 7, Original: "…the right *y*-axis shows RMSD values (black)… and the left *y*-axis shows ΔBIC(RMSD) values (red)…"

Extended Data Fig. 7, Revised: "…the right y-axis shows RMSD values (green)… and the left y-axis shows ΔBIC(RMSD) values (black)…"

Extended Data Table 1: Update the time constants and associated uncertainties in the "This work" row to reflect the revised values.
→ We thank the referee for noting this. We have updated the "This work" row of Extended Data Table 1 to reflect the revised time constants and uncertainties, consistent with the main text and Fig. 3c.

Minor revision suggestions: Supplementary Information
Page 39 (Supplementary Note 1): Clarify the reference to three versus four isomerization mechanisms.
→ We thank the referee for noting this inconsistency. The title of Supplementary Note 1 refers to "three mechanisms", but its opening sentence stated "four distinct mechanisms" while listing only three: rotation, inversion, and hula-twist. These three are the representative classical mechanisms illustrated in Fig. 1a, and their combinations (for example, inversion-assisted rotation) are referred to separately. We have corrected the opening sentence to "three", consistent with the title, the three mechanisms listed, and Fig. 1a.

Page 44 (Supplementary Note 4): The text mentions five dominant candidate motions, including OHT, which is not reported elsewhere in the revised manuscript; tables and figures indicate four motions. Please ensure consistency.
→ We thank the referee for noting this inconsistency. OHT was a leftover from an earlier draft; the analysis uses the four basis motions (ROT, INV, NND, and CNT). We have removed OHT from Supplementary Note 4 and from the footnote of Extended Data Table 2. The sentence in Supplementary Note 4 is revised as follows:
Original: "…focusing on five dominant, candidate internal motions, CNT, ROT, INV, NND, and OHT, and…"
Revised: "…focusing on four dominant, candidate internal motions, ROT, INV, NND, and CNT, and…"

Page 56: Duplicate sentence: "If I1 had perfect C2h symmetry…"
→ We thank the referee for noting this duplication. We have removed the redundant occurrence of the sentence "If I_1 had perfect C_{2h} symmetry…" on page 56.

Remarks on code availability
I was able to run the code on the Code Ocean platform. However, I did not install or execute the code locally, nor did I test it on datasets other than those provided with the manuscript. A README file is included with instructions for installation and execution.
→ We thank the referee for confirming that the deposited code runs on the Code Ocean platform. In this revision, we have expanded the Code Ocean capsule to include the complete workflow, and we have improved the README and manifest, as detailed in our responses to Referee 1.

Responses to the Comments from Referee #3

I co-reviewed this manuscript with one of the reviewers who provided the listed reports.
→ We thank the referee for co-reviewing the manuscript; the points raised in the joint report are addressed in our responses above.

Additional Changes

(i) **Adoption of Standardized Abbreviation (*trans*/*cis*-AB):** We standardized the abbreviation of azobenzene to "AB" (i.e., *trans*-AB and *cis*-AB) globally across the manuscript, figures, and tables. As this update is purely notational and does not affect any scientific results or conclusions, these frequent text replacements have been left unmarked in the tracked-changes version so that the tracked changes remain legible.

(ii) **Refinement of Data Processing and Reanalysis:** During this revision, while ensuring consistency between the manuscript and the Code Ocean implementation, we refined the data-processing procedure, including the propagation of the experimental uncertainties, and repeated the entire analysis. Crucially, while these improvements slightly update the fit-dependent values across the manuscript and Supplementary Information, all original discussions and scientific conclusions remain strictly unchanged. The revised procedure is detailed in the "Data processing" section of Methods and in Supplementary Methods 1 and 3; the resulting numerical changes are summarized in the table below rather than marked individually in the tracked-changes version so that the substantive revisions remain easy to follow.

Comparison of reported values between the previous submission and the present revision. All qualitative conclusions are identical; the quantitative values changed only slightly, all of them within the reported uncertainties.

Quantity	Previous submission	Present revision	Comment
Reaction mechanism and motional assignment			
Reaction pathway	*trans*-AB → I_1 → I_2 → *cis*-AB	*trans*-AB → I_1 → I_2 → *cis*-AB	unchanged
Motional contribution, *trans*-AB → I_1	CNT 100%	CNT 100%	unchanged
Motional contribution, I_1 → I_2	ROT 70%, CNT 30%	ROT 67%, CNT 33%	assignment unchanged
Motional contribution, I_2 → *cis*-AB	ROT 53%, INV 40%, CNT 7%	ROT 56%, INV 39%, CNT 5%	assignment unchanged
Best model, I_2 → *cis*-AB (BMA)	ROT + INV + CNT	ROT + INV + CNT	unchanged
Kinetics			
Instrument response function (FWHM)	170 fs ± 49 fs	169 fs ± 5 fs	within uncertainty
τ_1	0.4 ± 0.2 ps	0.4 ± 0.2 ps	unchanged
τ_2	2.5 ± 0.9 ps	2.7 ± 1.0 ps	within uncertainty
τ_3	14 ± 3 ps	14 ± 4 ps	central value unchanged
Branching			
α (I1 scaling, $\times 10^{-4}$)	3.672 ± 0.635	3.655 ± 0.629	within uncertainty
β (I_1 → I_2)	1.00 ± 0.24	1.00 ± 0.19	central value unchanged

γ ($I_2 \rightarrow$ *cis*-AB)	0.89 ± 0.15	0.91 ± 0.07	within uncertainty
Structural parameters (TRXL-refined)			
I_1 ∠CCNN	±87.7° ± 10.3°	±87.0° ± 13.9°	within uncertainty
I_2 ∠CNNC	118.4° ± 2.1°	115.5° ± 1.7°	consistent within uncertainties
I_2 ∠CCNN	−74.9° ± 6.2°	−71.9° ± 6.3°	within uncertainty
cis-AB ∠CNNC	10.6° ± 7.4°	10.8° ± 7.5°	within uncertainty
cis-AB ∠CCNN	78.5°, 78.8° ± 10.0°	77.0°, 77.3° ± 4.7°	within uncertainty
Fitting quality (TRXL-refined), χ_ν^2			
I_1 ($SADS_1$)	0.30	0.24	comparable fit quality
I_2 ($SADS_2$)	0.30	0.33	comparable fit quality
cis-AB ($SADS_3$)	1.01	0.97	comparable fit quality

(iii) **Enhanced Visualization of Molecular Motions:** We have improved Fig. 4b, Supplementary Fig. 8, and Supplementary Videos 1–6 to better illustrate the molecular motions. To more effectively convey the structural changes, the subpanels of I_1, I_2, and *cis*-AB now include the preceding structure, with the transition depicted via a sequence of interpolated structures. Furthermore, red arrows have been incorporated to explicitly highlight the trajectory of motion where the directionality might otherwise be complex. These changes are not marked in the tracked-changes version of the manuscript to keep the remaining tracked changes easy to follow.

(iv) **Standardization of Mathematical Notation and Editorial Refinements:** Following ISO 80000-2 guidelines, we have standardized all mathematical notations by setting physical quantities in italic type and operators, functions, and units in upright type. We have also ensured consistent labeling and corrected minor typographical errors. These refinements apply globally across the main text, figures, tables, Extended Data, and Supplementary Information. These changes are not marked in the tracked-changes version of the manuscript so that the remaining tracked changes remain legible.

(v) **Inclusion of a Supplementary List of Abbreviations:** To further facilitate readability and serve as a convenient reference, we have added a “List of Abbreviations” to the Supplementary Information. This section explicitly defines all key abbreviations, symbols, and mathematical notations employed throughout the manuscript.

(vi) **General Editorial and Textual Refinements:** Finally, we have made several minor editorial revisions throughout the manuscript to enhance clarity and internal consistency. These include standardizing notations and cross-references, as well as clarifying specific descriptions in the Methods and Supplementary Information. Importantly, none of these revisions alter any reported data, results, or scientific conclusions.

Responses to the Comments from Referee #1

I have sent these comments to the editorial office. Two of my students spent a considerable amount of time testing the code and checking the materials uploaded. The authors should upload the average raw diffraction data (one diffraction pattern per time delay + reference) and their complete workflow for reproducibility checks. The authors have successfully addressed all major concerns, and the manuscript is nearly ready for publication, pending a few comments from my students that can be treated as minor (attached below).
We appreciate the authors accommodating all of our requests. In particular, the code is much better structured now and significantly easier to follow.
→ We are grateful to the referee and the two students for the careful and substantial effort they devoted to testing our code and examining the deposited materials. Their feedback has directly improved the reproducibility of our deposit, and we are glad that the reorganized code proved easier to follow. Each of the remaining comments is addressed below.

Comments from Student 1:
Things for the authors to check:
The manuscript reported +/- 4.7° uncertainty bars of the cis-AB ∠CCNN, which appears to be derived from the profile-likelihood calculation. The code looks at the region $\Delta\chi 2 \leq 1$, around each minimum to determine a two-sided error range. However, it apparently does not account for when the inter-basin barrier is below the $\Delta\chi 2 = 1$ metric. As a result, the code combines the error widths of two minima, with a barrier around $\Delta\chi 2 \approx 0.8$ above the local basin, at approximately -21.8° and the lower one at -54°. The right-hand error of 4.65° is consistent with the -21.8° minimum, but the left-hand error of 2.64° appears to be the result of the deeper minimum at -54°, thus hybridizing the error bars. The authors should correct this profile interval calculation and clarify which minimum and errors were intended.
→ We thank the referee for this careful diagnosis, which is correct: the previous version of the code combined crossing points referenced to different minima, because it did not test whether a further minimum intervened between the reference minimum and a crossing point. The intended minimum is the one near −21.8°, which lies within the ±30° bound imposed in the refinement; the minimum near −54° is outside the bound and is not an alternative solution of the refinement. We have corrected the procedure. The revised convention that selects the reference minimum and the crossing points is now explained in Supplementary Method 4, and the specific case of ∠CCNN of *cis*-AB is described in Supplementary Method 12.4. The quoted uncertainty of ∠CCNN of *cis*-AB is now 4.4°, measured from the reference minimum at −21.6° to the retained crossing point at −17.2°. All uncertainties in Extended Data Table 3 were recomputed with the corrected procedure, and the table was updated accordingly; eight of the listed uncertainty values were slightly changed. The refined structural parameters themselves are unchanged.

Supplementary Method 1 states the auto-correlations should be greater than 0.8 for the first three RSVs and less than 0.5 for the fourth. However, recalculations from the deposited RSVs are 0.9875, 0.9042, 0.7100, and 0.6907, hence the third value is less than 0.8 and the fourth greater than 0.5. There may be some error in the reproduction methodology which found the auto-correlations using Hkifuncs.m using data from PCAstr.mat, though the values seem to match what is stored in pepcSVDStr.Viso.
→ We thank the referee for the careful reading and for independently reproducing the autocorrelation analysis. The autocorrelations recomputed by the referee (C(V) = 0.9875, 0.9042, 0.7100 and 0.6907 for the first four components) are exactly the values presented in Extended Data Fig. 4c and in the corresponding Source Data. There is no error in the referee's reproduction method. The discrepancy lies solely in the accompanying

description in Supplementary Method 1: the statement that C(**V**) > 0.8 for the first three components and < 0.5 for the fourth does not reflect the actual values. We have revised Supplementary Method 1 to state the criterion that was actually decisive, namely whether the RSV shows a resolvable kinetic feature. This correction affects only the wording of Supplementary Method 1. The deposited data, Extended Data Fig. 4, and the conclusion that three significant components are present remain unchanged.

While the capsule does provide the atomic coordinates of the intermediate structures for both I1 and I2, it does not provide XMS-CASPT2 input files, output logs, gradients, and convergence metrics. However, the Methods section provides details regarding the XMS-CASPT2 computation settings and the rigid CASPT2 PES scans are shown in Supplementary Figure 7. While ORCA inputs are present, these relate to fixed geometry charge calculations rather than XMS-CASPT2 geometry optimizations. The authors should provide the necessary records to fully reproduce the reported structures.

→ We are grateful to the referee for this meticulous audit. The criticism is well-founded: the Code Ocean capsule included the final coordinates but not the primary computational records. The capsule now includes, for every reported structure, the complete BAGEL input, the full output logs with per-cycle analytic gradients and convergence tables, the optimization trajectories, and the final orbitals, together with a README mapping each reported value to the corresponding files.

We should also clarify that every XMS-CASPT2 geometry optimization was performed with BAGEL, which provides fully analytic nuclear gradients for internally contracted (X)MS-CASPT2 (MacLeod and Shiozaki, *J. Chem. Phys.* **142**, 051103 (2015); Vlaisavljevich and Shiozaki, *J. Chem. Theory Comput.* **12**, 3781 (2016)). The ORCA inputs in the capsule belong to fixed-geometry single-point charge calculations at the optimized geometries. We note that although ORCA was not used for the optimizations, the CASSCF-based single-point calculations were meticulously set up to be fully consistent with the BAGEL ones, utilizing the same basis set and active space. The computational settings are stated in the Methods, where this point is now clarified.

In the logs deposited on Code Ocean, the gradient and energy criteria are fully satisfied (well below their respective thresholds) for all four optimizations (*trans*-AB, *cis*-AB, I_1, and I_2), triggering the normal and successful termination of the program. The maximum-displacement criterion, which BAGEL evaluates in internal coordinates, remains partly unsatisfied solely because the soft phenyl torsions permit motion along nearly flat directions of the potential energy surface after the gradient and the energy have converged; a stationary point is established by the gradient and the energy. The deposited script verify_convergence.py reads all three criteria from the logs and confirms this status for each optimization.

The Tikhonov regularized λ constant seems inconsistent, and is stated to be λ= 7.5 in Supplementary Method 12.4 and 12.7, but takes a value of approximately 4.74 in the code. The deposited penalty and RMSD are mutually consistent with λ =4.74, so there appears to be a discrepancy between the code and output versus the penalty supplied in Supplementary information, which is consistent with λ =7.5.

→ We thank the referee for identifying this inconsistency. The constant λ recorded in the deposited code was wrong: it read 4.7434 $Å^{-2}$ instead of 7.5 $Å^{-2}$. The refinement reported in the manuscript used λ = 7.5 $Å^{-2}$, as stated in Supplementary Methods 12.4 and 12.7; no refinement was carried out at 4.7434 $Å^{-2}$, and the deposited refined structure of *cis*-AB is consistent with λ = 7.5 $Å^{-2}$. In the previously deposited output, however, the penalty term for this structure had been recomputed with the wrong constant, which is why the deposited penalty and RMSD appeared mutually consistent with 4.7434 $Å^{-2}$. We have corrected the constant and the mislabeled output in the capsule uploaded to Code Ocean. We have also added the code and output of the evidence-maximization scan that determines λ (05_sspm_bma/lambda_evidence/) to that capsule; the scan returns λ = 7.5 $Å^{-2}$ and reproduces the reported refinement. No value reported in the manuscript changes as a

result of this correction.

The current structural SSPM uses 511 q-points, but uses an Neff of 110 for the BIC penalty via a Chebyshev polynomial fit, consisting of three SADS and seven solvent LSVs. This is a reasonable method, but this value seems almost entirely dependent on the solvent singular vectors, which contribute over 99.999% to the pooled residual sum of squares at K =110. The authors should justify the relative weighting used and demonstrate how the BIC changes with Neff.

→ The referee's observation is correct: at $K = 110$, the seven solvent singular vectors account for 99.9998% of the pooled residual sum of squares, and the determination of N_{eff} is therefore governed mainly by the solvent curves. This dominance reflects amplitude: the solvent curves are normalized to unit length, while the three SADS retain the absolute scale of the experimental data, and their amplitude is smaller by a factor of about 1,800.

To examine the influence of this imbalance, we repeated the determination of N_{eff}, varying both the set of curves entering the fit and their relative weights. The seven solvent vectors alone return the same value, $N_{eff} = 110$. The three SADS alone return $N_{eff} = 183$. We also varied only the weights, keeping all ten curves. In one case, each curve was rescaled so that its root mean square amplitude equals one. This rescaling returns $N_{eff} = 91$. In the other case, the three SADS were rescaled together, so that their total sum of squares equals that of the seven solvent curves. This rescaling also returns $N_{eff} = 91$. We then examined how the model selection of SSPM depends on the value of N_{eff}. The results, which have been added to Supplementary Method 13 of the revised manuscript, show that the selected models for both *trans*-AB → I_1 and I_1 → I_2 are unchanged over the entire range of N_{eff} examined.

Additional comments from Student 2:

- Pro: They have provided averaged data for the anisotropic, isotropic, and azimuthal components for the solvent and solution. This averaged data is not actually an input for the analysis. This is clarified in data/averaged/README.md, where instead the shot-pairing data that is obtained from processing the single-shot data is given, where they give one example.

- Pro: The code is much better organized, with files and folders more clearly labeled, as well as RE-ADMEs for every major section, a global one, and MANIFEST.md, and additional commenting. Very good job.

→ We thank the referee for this generous assessment. The reading is exactly as intended: the averaged curves are reference data, and the shot-pairing output is the input to the analysis, as stated in data/averaged/README.md. We are pleased that the reorganized code and its documentation made the deposit easier to navigate.

- Con: There is a mixing of the global vs. relative file paths, with more confusion brought from how Code Ocean handles data files. This does not affect running due to a helper script, and is therefore not a top priority, but should be clarified if possible.

→ The referee's description is accurate. Data locations are written in three forms in the deposit: module-local paths (data/ inside a module), repository-root paths (../data/), and the Code Ocean mount (/data). The helper script the referee mentions, setup_data_links.sh, maps the three onto one another, which is why the mixture does not affect execution. We have clarified the mapping through documentation, as described below.

o E.g. for 02_svd_kinetics we have local relative path.

E.g. we have local file paths stated in 02_svd_kinetics as 02_svd_kinetics/data/Shot_pairring_241207/ and the global read-me as02_svd_kinetics/, Shot_pairring_241207.m (minor typo here, should be fixed).

→ The misspelling has been corrected to Shot_pairing throughout the code tree, including the global README. The subfolder shot_pairring/ in the Data Asset and the Analyzer:Shot_pairring_241207 tag recorded in each run's Specification.txt were deliberately left unchanged. Every deposited log and provenance entry refers to the data by these two strings. Renaming them would therefore leave those records pointing at names that no longer exist. setup_data_links.sh maps the two spellings onto each other, and the README at the root of the Data Asset now states this explicitly.

o But for 05_sspm_bma, the data is not in the relative data folder but the global one.
In 05_sspm_bma the file paths are ../data/sads_svd/PCAStr.mat and similar.
This could be in part due to being accessed by different components instead of just one, but I'm not 100% sure because looking at the table indata/README.md in the final col-umn where it clarifies placement in manuscript and what component accesses it, I can-not see a pattern for each type of data storage.
→ The referee's inference is correct. The intended convention was that data read by several modules, such as the SADS and singular-vector data under ../data/sads_svd/ which both 01_pepc and 05_sspm_bma read, are referenced from the repository root, whereas data used by a single module, such as the shot-pairing curves of 02_svd_kinetics, are kept module-local. This convention was, however, not applied uniformly as the modules were written at different stages of the project, and this is why no clean pattern is visible in the table of data/README.md. Rather than leaving the reader to infer a pattern, we now state the actual location of every dataset explicitly in a single table, as described in the responses to the following comments.

o I have to ask the question: Why is data storage handled differently here? Is there an intentional design reason, or perhaps related to different people writing, writing certain components later than others, etc.
There is room for disagreement here on whether it is worthwhile to change and whether it should be changed in the first place.
→ Both explanations the referee suggests are correct. There was an intentional convention, the one described above, and the modules were written at different stages of the project, which is why the convention was not carried through uniformly. We considered unifying the paths in this revision. Rewriting them, however, would change every deposited output, log, and provenance record that references them, and the reproducibility of the entire capsule would then have to be re-verified. As the referee notes, there is room for disagreement here; we have instead documented the actual locations, as described in the response to the next comment, which resolves the practical difficulty while leaving the deposited records untouched.

o This is made more confusing by how everything on Code Ocean is stored in data/...
This does make sense if you understand Code Ocean/Git requirements, but in combina-tion with the inconsistency above it makes it very annoying to track data locations.
It means that for about half the project components on Code Ocean, you need check the individual readme for the relative path and then the data/ readme to find its actual path when referencing data.
• (I assume the Code Ocean will be linked when the paper is published, so it will not just be me having to parse the code situation.)
→ The referee's understanding is correct: the placement of all data under /data is a requirement of Code Ocean, which keeps the versioned code tree at /code and attaches the data as a read-only Data Asset mounted at /data. To remove the need to consult two READMEs, the global README now contains a new subsection, "Where the data live, and how paths resolve". It explains how the paths written in the code resolve in a local checkout and on Code Ocean, and it lists the complete mapping in a single table: the path used by the code, the Data Asset subfolder behind it, and the module that reads it. A data location can therefore be resolved from this one table, without consulting the README of the Data Asset. The same table appears in that README as

well, with the manuscript item that each dataset supports, for readers who reach the capsule from the published paper.

- In my opinion, this point should be mentioned as a warning in the global readme (where most people would start), even just as a reference like "For essential clarification on data locations in local vs. Code Ocean, see data/README.md"), because it is easy to miss if you jump straight to looking at a module. It would fit well within the Locally section, but exact placement is their choice.
o Even without changing anything about the code, this addition to documentation would improve things immensely.
→ We have adopted this suggestion. The “Locally” section of the global README now contains the pointer the referee proposes, directing the reader to the subsection described above before any module is run on its own. Since that subsection sits in the global README itself, the reader who starts there finds the pointer and the full mapping in the same file. We agree that documentation, rather than a change to the code, was the appropriate remedy, and we thank the referee for the concrete suggestion.
