# Supplementary material for: X-ray liquidography decodes complex motions in azobenzene isomerization
Source: Nature. 2026 Sep 30;658(8136):658–65. doi: 10.1038/s41586-026-11068-4 (PMC13668673; doi:10.1038/s41586-026-11068-4)
Supplement: Supplementary file 2 [file 41586_2026_11068_MOESM1_ESM.pdf]

nature portfolio
https://doi.org/10.1038/s41586-026-11068-4

# Supplementary information

# X-ray liquidography decodes complex motions in azobenzene isomerization

In the format provided by the authors and unedited

**Supplementary Information for**

# X-ray liquidography decodes complex motions in azobenzene isomerization

Jungmin Kim[1,2∇], Hosung Ki[1,2∇], Seonggon Lee[1,2], Alekos Segalina[1,2], Yunbeom Lee[1,2], Hyotcherl Ihee[1,2*]

[1]Department of Chemistry, Korea Advanced Institute of Science and Technology (KAIST), Daejeon, 34141, Republic of Korea

[2]Center for Advanced Reaction Dynamics (CARD), Institute for Basic Science (IBS), Daejeon, 34141, Republic of Korea

[∇]These authors contributed equally to this work.

*Corresponding author. Email: hyotcherl.ihee@kaist.ac.kr

# Table of Contents


**List of Abbreviations ........ 5**

A. Experimental techniques and light source ........ 5

B. General quantities and metrics ........ 5

C. Scattering-data reduction and decomposition ........ 5

D. Scattering and analysis variables and symbols ........ 6

E. Structural refinement and statistical analysis ........ 7

F. Geometric symbols ........ 7

G. Structural coordinates and basis motions ........ 8

H. Quantum chemistry and molecular dynamics ........ 8

I. Chemical species and electronic states ........ 9

J. Software (data processing and computation) ........ 10

**Supplementary Methods ........ 11**

Supplementary Method 1 | Singular value decomposition (SVD) and kinetic analysis of isomerization kinetics ........ 11

Supplementary Method 2 | Obtaining species-associated difference scattering curves ... 15

Supplementary Method 3 | Chi-square as a fitting criterion for the structural analysis of SADSs ........ 19

Supplementary Method 4 | Error estimation for structural parameters ........ 21

Supplementary Method 5 | Calculation of simulated SADSs ........ 25

Supplementary Method 6 | Debye scattering equation for simulating the scattering signal from solute structures ........ 26

Supplementary Method 7 | Equation for simulating the anisotropic scattering signal from solute structures ........ 27

Supplementary Method 8 | Atomic charge calculations for MD simulations ........ 28

Supplementary Method 9 | MD simulations for simulating cage terms ........ 30

Supplementary Method 10 | Fourier sine transform of SADSs in *q*-space to retrieve structural information in *r*-space ........ 33

Supplementary Method 11 | Validating the kinetic model through linear combination fitting analysis ........ 34

Supplementary Method 12 | Systematic Scan for Parsimonious Motions (SSPM) to characterize structural motions ........ 36

Supplementary Method 13 | $\chi^2$-Based BIC for SSPM and $N_{\mathrm{eff}}$ Determination ........ 45

Supplementary Method 14 | Basis-Motion Analysis: Algorithm, Alignment, and RMSD-Based BIC ........ 50

Supplementary Method 15 | Motional Contribution Analysis (MoCA) ........................... 55

**Supplementary Notes ........................................................................ 57**

Supplementary Note 1 | Explanation of three mechanisms for *trans*-to-*cis* isomerization proposed in the literature ........................................................................ 57

Supplementary Note 2 | Analysis of the anisotropic scattering signal ............................ 57

Supplementary Note 3 | Discussion on the SSPM results ............................................... 60

Supplementary Note 4 | Discussion on the basis motions analysed by BMA and MoCA ........................................................................ 63

Supplementary Note 5 | Comparison of present CASPT2-calculated structures with previously reported theoretical structures ........................................................ 64

Supplementary Note 6 | Comparison of quantum chemically predicted and TRXL-refined intermediate structures ........................................................................ 66

Supplementary Note 7 | Comparison of observed isomerization pathway with theoretical predictions ........................................................................ 69

Supplementary Note 8 | Comparison of observed isomerization pathway with spectroscopic assignments ........................................................................ 71

Supplementary Note 9 | Quantitative assessment of volume-conserving character using radius of gyration ........................................................................ 72

Supplementary Note 10 | Comparison of TRXL and spectroscopy results on the determination of the *cis*/*trans* product fractions ............................................................ 73

Supplementary Note 11 | Cartesian realization of internal coordinate changes ............... 75

Supplementary Note 12 | Solute-only SADSs in real space ............................................ 78

**Supplementary Figures ........................................................................ 80**

Supplementary Fig. 1 | Data reduction pipeline for time-resolved X-ray liquidography. 80

Supplementary Fig. 2 | Data analysis pipeline for time-resolved X-ray liquidography. .. 81

Supplementary Fig. 3 | A schematic for structural analysis, including SSPM, BIC, BMA, and MoCA. ........................................................................ 83

Supplementary Fig. 4 | Results of SVD analysis of the isotropic component of the neat methanol dataset for extracting solvent reference components for PEPC treatment. ...... 84

Supplementary Fig. 5 | Results of SVD analysis of the anisotropic component of the neat methanol dataset for extracting solvent reference components for PEPC treatment. ...... 85

Supplementary Fig. 6 | Comparison of theoretical difference scattering curves before and after PEPC treatment. ........................................................................ 86

Supplementary Fig. 7 | CASPT2 potential energy surfaces of azobenzene along CNNC and CNN angles. ........................................................................ 87

Supplementary Fig. 8 | Three-dimensional molecular structures of *trans*-AB, $I_1$, $I_2$, and *cis*-AB obtained from TRXL, aligned in the N–N fixed frame. ...................................... 88

**Supplementary Tables** ........ **89**

Supplementary Table 1 | Summary of key experimental parameters for fs-TRXL measurements ........ 89

Supplementary Table 2 | Structural motions and their combinations evaluated in the SSPM analysis. ........ 91

Supplementary Table 3 | RMSD and ΔBIC(RMSD) values from BMA analysis for TRXL-refined structures. ........ 94

Supplementary Table 4 | RMSD and ΔBIC(RMSD) values from BMA analysis for CASPT2-calculated structures. ........ 96

Supplementary Table 5 | MoCA results for the best models of the *trans*-AB → $I_1$, $I_1$ → $I_2$, and $I_2$ → *cis*-AB transitions. ........ 98

Supplementary Table 6 | Dependence of the SSPM model selection on $N_{eff}$ ........ 99

Supplementary Table 7 | Solvent swept volume for each structural transition. ........ 100

Supplementary Table 8 | RMSD between the initial and final structures of each transition. ........ 101

**References** ........ **102**

## List of Abbreviations

### A. Experimental techniques and light source

| Abbreviation | Definition |
|---|---|
| fs-TA | Femtosecond transient absorption |
| fs-TRXL | Femtosecond time-resolved X-ray liquidography |
| TA | Transient absorption |
| TRXL | Time-resolved X-ray liquidography |
| XFEL | X-ray free-electron laser |
| YAG | Yttrium aluminium garnet (crystal used to establish the approximate temporal and spatial overlap of the optical pump and X-ray probe pulses) |

### B. General quantities and metrics

| Abbreviation | Definition |
|---|---|
| AUC | Area under the curve |
| FWHM | Full width at half maximum |
| IRF | Instrument response function |
| SNR | Signal-to-noise ratio |

### C. Scattering-data reduction and decomposition

| Abbreviation | Definition |
|---|---|
| DWF | Debye–Waller factor |
| FST | Fourier sine transform |
| LSV | Left singular vector |
| PDF | Pair distribution function |
| PEPC | Projection to extract the perpendicular component |
| RDF | Radial distribution function |
| RSV | Right singular vector |
| SADS | Species-associated difference scattering curve |

| | |
|---|---|
| SVD | Singular value decomposition |

## D. Scattering and analysis variables and symbols

| Abbreviation | Definition |
|---|---|
| $q$ | Magnitude of the momentum transfer vector ($q = 4\pi\sin\theta/\lambda$, where $\lambda$ is the X-ray wavelength and $2\theta$ is the scattering angle) |
| $t$ | Time delay between the optical pump and the X-ray probe |
| $r$ | Interatomic distance in real space |
| $\perp$ (superscript) | Perpendicular component of the scattering signal, extracted by PEPC |
| $S(q, t)$ | Scattering curve as a function of $q$ and time delay $t$ |
| $S_{\mathrm{iso}}(q, t)$ | Isotropic scattering curve |
| $S_{\mathrm{aniso}}(q, t)$ | Anisotropic scattering curve |
| $S_{\mathrm{azi}}(q, t)$ | Azimuthal scattering curve |
| $\Delta S(q, t)$ | Difference scattering curve (laser-on minus laser-off); the superscript "raw" denotes the curve before PEPC and $\perp$ denotes the perpendicular component after PEPC |
| $\Delta S_{\mathrm{iso}}(q, t)$ | Isotropic difference scattering curve |
| $\Delta S_{\mathrm{aniso}}(q, t)$ | Anisotropic difference scattering curve |
| $\Delta S_{\mathrm{azi}}(q, t)$ | Azimuthal difference scattering curve |
| $q\Delta S(q, t)$ | Difference scattering curve multiplied by $q$, plotted to enhance the high-$q$ features associated with solute structural changes |
| $R(r)$ | Radial distribution function in real space |
| $\Delta R(r)$ | Difference radial distribution function in real space, displayed as $r^2\Delta R(r)$ |
| $g_{ij}(r)$ | Site-site pair distribution function between atom sites $i$ and $j$, obtained from molecular dynamics simulations |
| $n_q$ | Number of $q$ points (rows of the SVD data matrix **A**) |
| $n_t$ | Number of time points / delays (columns of the SVD data matrix **A**) |
| $n_{\mathrm{H}}$ | Number of solvent-related principal components (determined for PEPC) |
| $n_{\mathrm{PC}}$ | Number of significant signal components identified by SVD |

| | |
|---|---|
| $\sigma_{[x]}(q, t)$ | Standard error of the mean of the quantity x ([x] = azi, iso, or aniso), obtained by standard error propagation |
| $\tau_1$, $\tau_2$, $\tau_3$ | Time constants of the kinetic model (common exponential time constants shared across the three RSVs) |
| $\chi^2$ | Chi-square goodness-of-fit metric |
| $\chi_v^2$ | Reduced chi-square ($\chi^2$ per degree of freedom) |
| $\sigma^2$ | Mean-square atomic displacement in the Debye–Waller factor, $e^{(-\sigma^2 q^2/2)}$ |

## E. Structural refinement and statistical analysis

| Abbreviation | Definition |
|---|---|
| BIC | Bayesian information criterion |
| BMA | Basis-motion analysis |
| KCA | Kinetics-constrained analysis |
| LCF | Linear combination fitting |
| LOO | Leave-one-out |
| MoCA | Motional contribution analysis |
| RMS | Root mean square |
| RMSD | Root-mean-square deviation (Kabsch-aligned) |
| RSS | Residual sum of squares |
| SSPM | Systematic scan for parsimonious motions |

## F. Geometric symbols

| Abbreviation | Definition |
|---|---|
| ∠CCCN | Improper angle quantifying pyramidalization at nitrogen (PYR) |
| ∠CCN | C–C–N bond angle |
| ∠CCNN | Dihedral measuring the twist of each phenyl ring out of the CNN plane (modified by CNT) |
| ∠CNN | Bending angle of the C–N bond relative to the N–N bond at each nitrogen (modified by INV) |

| | |
|---|---|
| ∠CNNC | Dihedral angle measuring the relative orientation of the two phenyl rings about the N–N bond (modified by ROT) |
| $\Delta r_{CN}$ | C–N bond-length change (CND) |
| $r_{NN}$ | N–N bond length (modified by NND) |

## G. Structural coordinates and basis motions

| Abbreviation | Definition |
|---|---|
| CND | C–N bond elongation, parameterized by $\Delta r_{CN}$ |
| CNT | C–N torsion; twist of each phenyl ring about the C–N axis (∠CCNN) |
| $d_{TL}$ | Shortest distance between the line connecting the two N atoms and the line connecting the two bonded C atoms; quantifies the out-of-plane displacement |
| INV | Inversion; independent variation of the two ∠CNN bending angles |
| NND | N–N bond-length change; the N–N distance ($r_{NN}$) |
| PYR | Pyramidalization at the nitrogen atom, quantified by the ∠CCCN improper angle |
| ROT | N–N rotation; change in the CNNC dihedral angle (∠CNNC) |

## H. Quantum chemistry and molecular dynamics

| Abbreviation | Definition |
|---|---|
| CASPT2 | Complete active space second-order perturbation theory |
| CASSCF | Complete active space self-consistent field |
| CC3 | Approximate coupled-cluster singles-doubles-triples method (used for benchmarking) |
| CI | Conical intersection (between the $S_1$ and $S_0$ surfaces) |
| ConInv | Concerted inversion |
| DRACO | Dynamic radii adjustment for continuum solvation |
| FC | Franck–Condon (state / region) |
| LINCS | Linear constraint solver (constraint algorithm in MD simulation) |
| MD | Molecular dynamics |

| | |
|---|---|
| NAMD | Nonadiabatic molecular dynamics |
| NPA | Natural population analysis |
| NPT | Isothermal–isobaric ensemble (constant number, pressure, temperature) |
| NVT | Canonical ensemble (constant number, volume, temperature) |
| OPLS-AA | Optimized potentials for liquid simulations, all-atom (force field) |
| PCM | Polarizable continuum model |
| PES | Potential energy surface |
| PME | Particle mesh Ewald (method for electrostatic interactions) |
| RASPT2 | Restricted active space second-order perturbation theory |
| RIJCOSX | Resolution-of-identity approximation with chain-of-spheres exchange |
| SC-NEVPT2 | Strongly contracted N-electron valence state second-order perturbation theory |
| SMD | Solvation model based on density |
| SS-CASSCF | State-specific complete active space self-consistent field |
| XMS-CASPT2 | Extended multi-state CASPT2 |

## I. Chemical species and electronic states

| **Abbreviation** | **Definition** |
|---|---|
| AB | Azobenzene |
| *cis*-AB | *cis*-Azobenzene |
| ETS | Energy transfer to solvent |
| GS | Ground state |
| $I_1$ | First intermediate |
| $I_2$ | Second intermediate |
| IVR | Intramolecular vibrational energy redistribution |
| $S_0$ | Ground state of *trans*-azobenzene |
| $S_0$* | Vibrationally hot S0 |
| $S_1$ | First singlet excited state of *trans*-azobenzene |

| | |
|---|---|
| $S_1$(D) | Dark intermediate on the $S_1$ surface |
| $S_1$(L) | Local minimum on the $S_1$ surface |
| *trans*-AB | *trans*-Azobenzene |
| VC | Vibrational cooling |

## J. Software (data processing and computation)

| Abbreviation | Definition |
|---|---|
| GROMACS | GROningen MAchine for Chemical Simulations; molecular-dynamics simulation package |
| JANPA | Open-source cross-platform implementation of natural population analysis on the Java platform |
| MATLAB | MATrix LABoratory; numerical computing environment (used for optimization, e.g., fminsearch) |
| MOLDEN | Pre- and post-processing program for molecular and electronic structures (used for electron-density export) |
| ORCA | General-purpose quantum-chemistry program package |
| pyFAI | Python Fast Azimuthal Integration; Python library for azimuthal integration (caking) of X-ray scattering images into q and azimuthal-angle bins, providing the basis for the isotropic and anisotropic (Legendre $P_0$/$P_2$) decomposition |

# Supplementary Methods

## Supplementary Method 1 | Singular value decomposition (SVD) and kinetic analysis of isomerization kinetics

We used SVD to examine the time-dependent behaviour of the TRXL data and to determine the number of significant components. In SVD, an $n_q$ (number of $q$ points) × $n_t$ (number of time-delay points) data matrix **A** is decomposed as $\mathbf{A}=\mathbf{USV^T}$, where **U** is the $n_q \times n_t$ matrix of left singular vectors (LSV), **S** is the $n_t \times n_t$ diagonal matrix of singular values, and $\mathbf{V^T}$ is the transpose of the $n_t \times n_t$ matrix of right singular vectors (RSV). The $n$-th LSV and RSV are referred to as $\mathrm{LSV}_n$ and $\mathrm{RSV}_n$, respectively. The LSVs represent the time-independent signal components in $q$-space, **S** contains the singular values that represent the relative contribution of each LSV to the data, and the RSVs describe the time-dependent changes in the contribution of the corresponding LSV. A more detailed explanation of the SVD analysis can be found elsewhere[1].

In this work, SVD was applied to several different scattering datasets. The analysis of the solvent-independent isotropic data, $\Delta S^{\perp}_{\mathrm{iso}}(q, t)$, is presented in this section. The corresponding analysis of the solvent-independent anisotropic data is presented in Supplementary Note 2. The raw anisotropic data of the sample were used to determine the IRF and time zero (Methods). The neat-solvent data, both isotropic and anisotropic, were used to obtain the solvent reference for the PEPC treatment (Methods). The SVD of the solvent-independent isotropic difference scattering curve, $\Delta S_{\mathrm{iso}}{}^{\perp}(q, t)$, yields **U**, **S**, and $\mathbf{V^T}$, satisfying the relationship $\Delta S^{\perp}_{\mathrm{iso}}(q, t) = \mathbf{USV^T}$. The autocorrelation values of the columns of **U** and **V**, denoted as C(**U**) and C(**V**), were then calculated to distinguish signal components (high autocorrelation) from those dominated by noise (low autocorrelation).

Based on the singular values and autocorrelation coefficients C(**U**) and C(**V**), we assessed the significance of each component. The first two components clearly represent signal: their autocorrelation coefficients are the highest (C(**V**) ≈ 0.99 and 0.90), and their RSVs vary smoothly and systematically with time delay. For the third and fourth components, however, the two measures give nearly equal, intermediate values: C(**V**) ≈ 0.71 and 0.69, with singular values equally close (Extended Data Fig. 4c). Rather than accepting both components as significant or discarding both as noise, we examined a further property: whether the RSV shows a resolvable kinetic feature. The RSV of the third component exhibits a smooth rise and decay

characteristic of a kinetic process. In contrast, the time dependence of the RSVs of the fourth and subsequent components consists of rapid fluctuations without any such feature, and is barely distinguishable from the random fluctuations independent of photoexcitation observed at negative time delays (Extended Data Fig. 4a). Three significant components were therefore identified, supporting the involvement of three structurally distinct reaction species (intermediates and/or products) in the isomerization process. The top three RSVs were globally fitted using the sum of exponential functions sharing common time constants to extract the time constants of the isomerization process. The fit minimized the total chi-square, $\chi^2_{\text{total}}$, which is defined as the sum of the chi-square values for the top three RSVs $\chi^2_n$:

$$\chi^2_{\text{total}} = \sum_{n=1}^{3} \chi^2_n \tag{S1}$$

$\chi^2_n$ represents the chi-square fit quality for the $n$-th RSV:

$$\chi^2_n = \sum_k \left[\left(\text{RSV}_n^{\text{exp}}(t_k) - \text{RSV}_n^{\text{calc}}(t_k)\right) / \sigma_n(t_k)\right]^2 \tag{S2}$$

Here, $\text{RSV}_n^{\text{exp}}(t_k)$ is the experimental $n$-th RSV at time delay $t_k$, $\text{RSV}_n^{\text{calc}}(t_k)$ denotes its theoretical counterpart (see below), and $\sigma_n(t_k)$ is the standard error of $\text{RSV}_n^{\text{exp}}(t_k)$.

This standard error is needed for two distinct purposes. The first is to weight the data points in the fit that determines the time constants. The second is to quantify the uncertainty of those time constants. A different estimate of $\sigma_n(t_k)$ is used for each purpose: an analytic estimate, $\sigma_n^{\text{ana}}(t_k)$, for the first, and a bootstrap estimate, $\sigma_n^{\text{boot}}(t_k)$, for the second. The two estimates, and the reason they differ, are described below. Both estimates are based on the same quantity: the per-point uncertainty $\sigma_{\text{iso}}^{\perp}(q, t_k)$ of the solvent-independent difference scattering data before the time-pooling. This uncertainty was obtained by propagating the run-level standard error $\sigma_{[\text{x}]}(q, t)^{\text{run}}$ (Methods, "Data processing", Eq. (1)) through the across-run merging and the PEPC treatment. The two estimates differ in how this pre-pooling uncertainty is then propagated through the time-pooling and the SVD.

For the fit, $\sigma_n^{\text{ana}}(t_k)$ was obtained by analytic error propagation. Two linear operations map the data to the RSVs. The first is the two-point time-pooling, which averages adjacent time points. The second is the SVD, which projects the data at each time onto the LSVs to give each RSV value as a linear combination over $q$. Because both operations are linear, the RSVs

depend linearly on the data. The per-point data error $\sigma_{iso}^{\perp}(q, t_k)$ was therefore propagated analytically through the two-point time-pooling and the SVD to yield the error $\sigma_n^{ana}(t_k)$ of the RSVs. This analytic estimate reproduces the correct relative magnitudes of $\sigma_n(t_k)$, and therefore yields the correct time constants. It does, however, under-estimate the absolute magnitude of $\sigma_n(t_k)$, because it does not fully capture the correlation that the two-point time-pooling introduces between neighbouring time points.

The parametric bootstrap estimate $\sigma_n^{boot}(t_k)$, which is used to estimate the uncertainties, was not used here as the fit weight. The weight should reflect the error of each RSV value at a fixed set of LSVs, which is what the analytic propagation gives. A bootstrap, by contrast, re-runs the SVD on every realization, so the LSVs also vary between realizations. The resulting bootstrap estimate $\sigma_{\mathrm{n}}^{boot}(t_k)$ is inflated by this basis variation and no longer reflects the per-point data noise alone. Using it as the weight would distort the relative magnitudes of $\sigma_n(t_k)$ and shift the fitted time constants. The analytic estimate $\sigma_{\mathrm{n}}^{ana}(t_k)$ affects the fit only through the relative weighting of the points, and the fitted time constants are unchanged by an overall rescaling of $\sigma_n(t_k)$; its over-inflation of the absolute chi square therefore does not shift the fitted time constants.

For the uncertainty of the time constants, however, this over-estimation of the chi square does matter. The $\sigma_{\mathrm{n}}^{ana}(t_k)$ is smaller than the true error by roughly $\sqrt{2}$, because it treats the correlated, neighbouring time points as independent; used in the profile-likelihood, it would over-inflate the chi-square and make the reported uncertainty of the time constants too small (its $\Delta\chi^2 = 1$ limit would correspond to only $\Delta\chi^2 \approx 0.5$ for the true error). The basis variation via a parametric bootstrap, in contrast, provides here a legitimate part of the uncertainty rather than a distortion. We therefore used the $\sigma_{\mathrm{n}}^{boot}(t_k)$, which restores the correct error magnitude and includes this basis variation, as the $\sigma$ in the profile-likelihood. To do this, we generated $N = 500$ bootstrap realizations of the measurement, as follows. We started from the measured, unsmoothed difference scattering data $\Delta S_{iso}^{\perp}(q, t_k)$, that is, the data before the two-point time-pooling. To make one realization, we added to every data point an independent random number, drawn from a Gaussian distribution whose standard deviation was that point's own uncertainty $\sigma_{iso}^{\perp}(q, t_k)$; the result is a synthetic dataset that looks like the measurement perturbed by one fresh draw of noise of the correct size. We then processed this synthetic dataset through exactly the same steps as the real data, first the two-point time-pooling and then the SVD, so that it produced its own set of RSVs. Adding the noise before the pooling is essential here: the pooling

averages each pair of neighbouring time points and so mixes their noise, and by injecting the noise first we let it be mixed in the same way as the real experimental noise, which reproduces the correlations that the pooling introduces. Repeating this independently $N$ = 500 times (enough for the results to converge), the 500 values of $\mathrm{RSV}_n(t_k)$ at each $n$ and $t_k$ form a distribution whose standard deviation is $\sigma_n^{\mathrm{boot}}(t_k)$. This $\sigma_n^{\mathrm{boot}}(t_k)$ was then used as the $\sigma$ in the profile-likelihood analysis that gives the 1$\sigma$ uncertainties of the time constants ($\Delta\chi^2 = 1$).

The theoretical counterpart $\mathrm{RSV}_n^{\mathrm{calc}}(t_k)$, introduced in Eq. (S2), was calculated using a multi-exponential function convolved with the instrument response function (IRF):

$$\mathrm{RSV}_n^{\mathrm{calc}}(t_k) = \left( \left( \begin{array}{c} A_{n0} \cdot \exp(-t/\tau_0) + \sum_{j=1}^{3} A_{nj} \cdot \exp\left(-t/\tau_j\right) \\ + A_{n4} \cdot \exp(-t/\tau_4) \end{array} \right) \cdot H(t) \right) \otimes \mathrm{IRF}(t) \qquad \text{(S3)}$$

Here $\tau_0$ = 20 fs is the fixed instrument-limited onset and $\tau_4$ = 1 μs represents a long-lived (non-decaying) offset (effectively constant over the measured window); $\tau_1$, $\tau_2$, $\tau_3$ are the three free time constants interpreted as the kinetic time scales. In this equation, the term $A_{nj}$ represents the amplitude of the $j$-th exponential function specifically for the $n$-th RSV, serving as a local parameter optimized independently for each RSV. $\tau_j$ ($j$ = 1, 2, 3) represents the time constant of the corresponding exponential function, treated as a global parameter shared across all RSVs ($n$ = 1, 2, 3) to ensure a consistent kinetic model. $H(t)$ is the Heaviside step function ($H(t)$ = 1 for $t \geq 0$, 0 otherwise), ensuring that the signal onset occurs only after photoexcitation. The symbol ⊗ denotes a convolution operation. Finally, IRF($t$) is the instrument response function (IRF), modelled as a Gaussian function with a full width at half maximum (FWHM) of approximately 169 fs; both the IRF width and the time-zero offset ($t_{0,\mathrm{ref}}$) were determined from a fit of this multi-exponential model to the first RSV (denoted as $\mathrm{RSV}_1$) of the time-pooled, pre-PEPC anisotropic difference scattering signal, and were then held fixed when the same model was fitted to the first three RSVs ($\mathrm{RSV}_1$, $\mathrm{RSV}_2$, and $\mathrm{RSV}_3$) of the isotropic difference scattering signal. Fitting with one or two exponentials did not yield satisfactory results, while fitting with four exponentials resulted in overfitting. The fixed onset ($\tau_0$) and offset ($\tau_4$) terms are not interpreted as kinetic time constants of the reaction; only the three free time constants $\tau_1$, $\tau_2$, and $\tau_3$ are interpreted as the time scales of the chemical processes. The resulting three exponential time constants, 0.4 ± 0.2 ps, 2.7 ± 1.0 ps and 14 ± 4 ps, were used to construct kinetic models for the analysis of species-associated difference scattering curves (SADSs),

described in the subsequent section.

**Supplementary Method 2 | Obtaining species-associated difference scattering curves**

Based on the kinetic model (shown in Fig. 3d,e), which was derived from the time constants and number of intermediates identified in the SVD analysis, we constructed experimental SADSs. To allow for the possibility that the intermediates may return to *trans*-AB without proceeding to the isomerization pathway, we extended the kinetic framework following the sequential model consistently adopted in previous studies[2-4]. We note that the kinetic model identifies the number of intermediates and their lifetimes, but the structural identity of each intermediate (i.e., what molecular geometry $I_1$ and $I_2$ correspond to) is determined separately through the structural refinement described below (see also Supplementary Method 12). The branching yields obtained from this structural refinement (Supplementary Method 12.3) show that the $I_1 \rightarrow I_2$ conversion proceeds with unity yield within experimental uncertainty (100 ± 19%), while the $I_2 \rightarrow$ *cis*-AB yield is 91 ± 7%, indicating that the pathway is predominantly sequential with at most a minor fraction of $I_2$ returning directly to the ground state (see Supplementary Note 10 for a discussion of the *trans*/*cis* product ratio).

The SADS for the *n*-th species, denoted as $\text{SADS}_n(q)$, is defined as the difference between the X-ray scattering curve of the *n*-th species and that of the ground state, *trans*-AB, expressed as $\text{SADS}_n(q) = S_n(q) - S_{trans}(q)$. Here, $S_n(q)$ is the X-ray scattering curve from the *n*-th species, while $S_{\text{trans}}(q)$ is the scattering curve from *trans*-AB. The photoisomerization process of *trans*-AB involves three distinct species, resulting in three SADS components: $\text{SADS}_1(q)$ corresponds to intermediate 1 ($I_1$), $\text{SADS}_2(q)$ to intermediate 2 ($I_2$), and $\text{SADS}_3(q)$ to the final product, *cis*-azobenzene (*cis*-AB).

To extract SADSs from the experimental data, we applied kinetics-constrained analysis (KCA). The process of extracting SADS is composed of the following steps. First, SADSs and the TRXL data have the following relationship.

$$\Delta S(q,\, t) = \sum_n \text{SADS}_n(q) \cdot m_n(t) \qquad \text{(S4)}$$

$\Delta S(q,\ t)$ represents the difference scattering curves, $\text{SADS}_n(q)$ corresponds to the SADS associated with the *n*-th species, and $m_n(t)$ represents the time-dependent concentration of the

$n$-th species. In our analysis, we utilized PEPC-treated difference scattering curves rather than raw difference scattering curves. Therefore, the corresponding equation is updated to reflect this, as follows: $\mathrm{SADS}_n(q)^{\perp} = (S_n(q) - S_{trans}(q))^{\perp} = S^{\perp}{}_n(q) - S^{\perp}{}_{trans}(q)$. The following relationship holds for the PEPC-treated difference scattering curves.

$$\Delta S^{\perp}(q,\, t) = \sum_n \mathrm{SADS}_n^{\perp}(q) \cdot m_n(t) \tag{S5}$$

$\Delta S^{\perp}(q, t)$ represents the PEPC-treated difference scattering curve, and $\mathrm{SADS}_n{}^{\perp}(q)$ represents the PEPC-treated SADS associated with the $n$-th species. This equation can be expressed in matrix form as follows.

$$\begin{aligned} \Delta S^{\perp}(q,\, t) &= \left[\Delta S^{\perp}(q,\, t_1), \Delta S^{\perp}(q,\, t_2), \ldots, \Delta S^{\perp}(q,\, t_{n_t})\right] \\ &= \left[\sum_n \mathrm{SADS}_n^{\perp}(q) \cdot m_n(t_1), \ldots, \sum_n \mathrm{SADS}_n^{\perp}(q) \cdot m_n(t_{n_t})\right] \\ &= \left[\mathrm{SADS}_1^{\perp}(q), \mathrm{SADS}_2^{\perp}(q), \ldots, \mathrm{SADS}_{n_i}^{\perp}(q)\right] \cdot \left[m_1(t), m_2(t), \ldots, m_{n_i}(t)\right]^{\mathrm{T}} \\ &= \mathbf{X}(q)\mathbf{M}(t)^{\mathrm{T}} \end{aligned} \tag{S6}$$

Here, $n_i$ is the number of species (in this case, three), and $\mathbf{X}(q)$ is a matrix containing the $\mathrm{SADS}_n{}^{\perp}(q)$ components, corresponding to the first term on the right side of the third line in equation (S6). Similarly, $\mathbf{M}(t)$ is a matrix representing the time profiles of the relative contributions, corresponding to the second term on the right side in the same line. Transposing the last line in equation (S6) results in the following equation in the well-known form of AX = B.

$$(\mathbf{X}(q)\mathbf{M}(t)^{\mathrm{T}})^{\mathrm{T}} = \mathbf{M}(t)\mathbf{X}(q)^{\mathrm{T}} = \left(\Delta S^{\perp}(q,\, t)\right)^{\mathrm{T}} \tag{S7}$$

The $\mathrm{SADS}_n{}^{\perp}(q)$s can be obtained by solving equation (S7) for $\mathbf{X}(q)$. Of the remaining terms in equation (S7), $\Delta S^{\perp}(q, t)$ is known, but $\mathbf{M}(t)$ is not yet determined. Based on the kinetic model (shown in Fig. 3d,e), $\mathrm{m}_n(t)$ can be expressed as follows.

$$\begin{aligned} m_1(t) &= \alpha \cdot (\exp(-t/\tau_2) - \exp(-t/\tau_1)) \\ m_2(t) &= \alpha \cdot \beta \cdot (\exp(-t/\tau_3) - \exp(-t/\tau_2)) \\ m_3(t) &= \alpha \cdot \beta \cdot \gamma \cdot (1 - \exp(-t/\tau_3)) \end{aligned} \tag{S8}$$

Here, $\alpha$ is the molar ratio, relative to the solvent molecules, of photoexcited *trans*-AB molecules that undergo *trans*-AB → $I_1$ transition; $\beta$ is the fraction of $I_1$ molecules that proceed to $I_2$; and $\gamma$ is the fraction of $I_2$ molecules that transition to *cis*-AB. The time constants $\tau_1$, $\tau_2$,

and $\tau_3$ in Eq. (S8) are not fitted here; they were fixed to the values obtained from the global fit of the first three RSVs in Supplementary Method 1 (0.4, 2.7 and 14 ps). The scaling factors $\alpha$, $\beta$ and $\gamma$, in contrast, are unknown at this stage and are the quantities determined by the analysis that follows.

To extract $\mathrm{SADS}_n{}^{\perp}(q)$s without prior knowledge of $\alpha$, $\beta$ and $\gamma$, we employed the following approach. Equation (S8) can be modified as follows.

$$\begin{aligned} m_1(t) &= \alpha \cdot m_1^*(t) \\ m_2(t) &= \alpha \cdot \beta \cdot m_2^*(t) \\ m_3(t) &= \alpha \cdot \beta \cdot \gamma \cdot m_3^*(t) \end{aligned} \tag{S9}$$

Then, considering that three species are involved in this system, substituting equation (S9) into equation (S6) yields:

$$\begin{aligned} \Delta S^{\perp}(q,\, t) = &\ [\mathrm{SADS}_1^{\perp}(q), \mathrm{SADS}_2^{\perp}(q), \mathrm{SADS}_3^{\perp}(q)] \cdot [m_1(t), m_2(t), m_3(t)]^{\mathrm{T}} \\ = &\ [\mathrm{SADS}_1^{\perp *}(q), \mathrm{SADS}_2^{\perp *}(q), \mathrm{SADS}_3^{\perp *}(q)] \cdot [m_1^*(t), m_2^*(t), m_3^*(t)]^{\mathrm{T}} \\ = &\ \mathbf{X}^*(q)\mathbf{M}^*(t)^{\mathrm{T}} \end{aligned} \tag{S10}$$

In this expression, $\mathbf{X}^*(q)$ is a matrix comprising $\mathrm{SADS}_n{}^{\perp}(q)$ components scaled by the unknown factors $\alpha$,$\beta$ and $\gamma$, corresponding to the first term on the right side of the third line in equation (S10). Specifically, the components $\mathrm{SADS}_n{}^{\perp *}(q)$ of the $\mathbf{X}^*(q)$ matrix are expressed as follows:

$$\begin{aligned} \mathrm{SADS}_1^{\perp *}(q) &= \alpha \cdot \mathrm{SADS}_1^{\perp}(q) \\ \mathrm{SADS}_2^{\perp *}(q) &= \alpha \cdot \beta \cdot \mathrm{SADS}_2^{\perp}(q) \\ \mathrm{SADS}_3^{\perp *}(q) &= \alpha \cdot \beta \cdot \gamma \cdot \mathrm{SADS}_3^{\perp}(q) \end{aligned} \tag{S11}$$

The matrix $\mathbf{M}^*(t)$, composed of $\mathrm{m}_n{}^*(t)$ components, is known, as defined in equation (S10). Consequently, in equation (S10), both $\Delta S^{\perp}(q,\ t)$ and $\mathbf{M}^*(t)$ are known, enabling the straightforward determination of $\mathbf{X}^*(q)$. Our strategy involves first calculating $\mathbf{X}^*(q)$ and subsequently extracting the scaling factors ($\alpha$, $\beta$ and $\gamma$) through structural analysis of these scaled SADSs.

It should be noted that the experimental and simulated SADS presented in the main text and figures correspond specifically to the scaled PEPC-treated SADS, denoted as $\mathrm{SADS}_n{}^{\perp *}(q)$, or its simulated counterpart, $\mathrm{SADS}_n{}'^{\perp *}(q)$, unless indicated otherwise.

Here we note that within each SADS fit, the relevant scaling factors and the structural parameters were optimized simultaneously, because they are interdependent: the scaling factors determine the absolute scale of the target SADS, thereby influencing the optimal structural

parameters, while the structural parameters dictate the scattering intensity profile required to match this scale, thereby influencing the optimal reaction yields. The fitting of the three SADSs was carried out sequentially to progressively constrain these scaling factors, as described below.

The refinement began with $SADS_3$ (corresponding to *cis*-AB minus *trans*-AB). Because $SADS_3{}^{\perp *}(q) = \alpha \cdot \beta \cdot \gamma \cdot SADS_3{}^{\perp}(q)$ (equation (S11)), fitting $SADS_3$ simultaneously determines the refined *cis*-AB structure and the product of the three scaling factors $\alpha \cdot \beta \cdot \gamma$. Starting from the CASPT2-calculated *cis*-AB structure, we optimized its ∠CCNN and ∠CNNC parameters against the experimental $SADS_3$, yielding a refined structure and a well-determined value of $\alpha \cdot \beta \cdot \gamma = (3.339 \pm 0.062) \times 10^{-4}$. Since the branching fractions satisfy $0 \leq \beta \leq 1$ and $0 \leq \gamma \leq 1$, the inequality $\alpha \cdot \beta \cdot \gamma \leq \alpha \cdot \beta \leq \alpha$ holds, providing physically motivated bounds for the subsequent fits. The SSPM for the *trans*-AB → $I_1$ transition ($SADS_1$) was then performed with $\alpha$ as a free parameter subject to the constraint $\alpha \geq \alpha \cdot \beta \cdot \gamma$, yielding the refined $I_1$ structure and $\alpha = (3.655 \pm 0.629) \times 10^{-4}$. Subsequently, the SSPM for the $I_1 \rightarrow I_2$ transition ($SADS_2$) was performed with $\alpha \cdot \beta$ as a free parameter bounded by $\alpha \cdot \beta \cdot \gamma \leq \alpha \cdot \beta \leq \alpha$, yielding the refined $I_2$ structure and $\alpha \cdot \beta = (3.655 \pm 0.287) \times 10^{-4}$. From these, the individual branching fractions were determined as $\beta = (\alpha \cdot \beta)/\alpha = (1.00 \pm 0.19)$ and $\gamma = (\alpha \cdot \beta \cdot \gamma)/(\alpha \cdot \beta) = (0.91 \pm 0.07)$.

The resulting optimized $\alpha$ value corresponds to an excited-state concentration of 9.0 ± 1.6 mM, calculated from the solvent density ($\rho \approx 792$ g/L) and molecular weight ($m \approx 32.04$ g/mol) via the relation of $c(I_1) = \alpha \times (\rho / m)$, where $c(I_1)$ is the concentration of *trans*-AB that forms $I_1$. This equation indicates that approximately 9.0 mM of *trans*-AB (out of 50 mM total) forms $I_1$ upon photoexcitation. The optimized branching factors, $\beta = (1.00 \pm 0.19)$ and $\gamma = (0.91 \pm 0.07)$, give the yields of the two subsequent steps: essentially all of the $I_1$ proceeds to $I_2$ (with a time constant of 2.7 ps), and about 91% of the resulting $I_2$ molecules subsequently convert to *cis*-AB (with a time constant of 14 ps). The sub-unity central value of $\gamma$ suggests that a minor fraction (about 9%) of the $I_2$ population may return to the *trans*-AB ground state via a competing pathway, although $\gamma$ is also consistent with unity within the experimental uncertainty. A detailed discussion of the branching yields, including a decomposition into intrinsic forward and backward rate constants and a comparison with spectroscopic quantum yield measurements, is provided in Supplementary Note 10.

**Supplementary Method 3 | Chi-square as a fitting criterion for the structural analysis of SADSs**

The structural parameters of each intermediate were optimized by minimizing the discrepancy between the simulated SADS, $SADS_n'(q)$, calculated from the structures, and the experimental SADSs, $SADS_n(q)$, with the aim of determining the optimal molecular structures. The apostrophe symbol (′) is used to denote "simulated." Ideally, the comparison should be made between $SADS_n(q)$ and $SADS_n'(q)$; however, the experimental SADSs that can actually be extracted from the measured data are derived from PEPC-treated experimental curves, corresponding to $SADS_n^{\perp}(q_j)$ rather than $SADS_n(q)$. Therefore, the structural fitting was performed by comparing $SADS_n^{\perp}(q_j)$ and $SADS_n'^{\perp}(q_j)$ instead. This approach has been well established and validated in previous studies[5-7]. A comparison between the $SADS_n'(q)$ and their PEPC-projected counterparts ($SADS_n'^{\perp}(q)$) is presented in Supplementary Fig. 6 to explicitly illustrate how the projection transforms the theoretical signal. We note that the simulated SADS curves shown in Fig. 3f appear noisy in $q$-space rather than smooth, because the PEPC treatment propagates noise from the experimentally derived solvent components into the simulated curves (see Supplementary Method 5 for details).

The fitting process involved minimizing the reduced chi-square score function, which quantifies the difference between the simulated and experimental SADSs. For the first step to calculate the reduced chi-square, the chi-square value for the $n$-th SADS is defined as:

$$\chi_n^2 = \sum_{j=1}^{n_q} \left[ \left( \mathrm{SADS}_n^{\perp *}(q_j) - \left( \mathrm{SADS}_n'^{\perp *}(q_j) + B_n(q_j) \right) \right) / \sigma_n(q_j) \right]^2 \qquad \text{(S12)}$$

$SADS_n'^{\perp *}(q_j)$ represents the simulated, PEPC-treated SADS multiplied by the scaling factors, as in equation (S11) for the experimental counterparts. For example, $SADS_1'^{\perp *}(q_j) = \alpha \cdot SADS_1'^{\perp}(q_j)$, $SADS_2'^{\perp *}(q_j) = \alpha \cdot \beta \cdot SADS_2'^{\perp}(q_j)$, and $SADS_3'^{\perp *}(q_j) = \alpha \cdot \beta \cdot \gamma \cdot SADS_3'^{\perp}(q_j)$. $B_n(q_j)$ represents the baseline correction term for the $n$-th SADS. We used a linear baseline model in $q\Delta S(q)$ ($q \cdot B_n(q) = a_n \cdot q + b_n$), corresponding to $B_n(q) = a_n + b_n/q$ in the $S(q)$ domain. This residual baseline has two origins. First, fluctuations in the liquid jet thickness and position during the measurement alter the liquid-to-air scattering ratio and create broad, low-frequency residuals in $q$-space. Second, the solvent reference used in the PEPC treatment contains a residual baseline of the same kind, because this baseline is present in the neat-solvent data and passes into the reference when it is obtained by SVD. The PEPC treatment then carries this

baseline into the solvent-independent signal. Because the amount of solvent removed by the PEPC treatment varies with time delay, this second contribution, and in particular its slope, varies with time delay as well. Since the SADSs are weighted differently over time delay through their concentration profiles, each contains a different residual baseline, and a separate baseline $B_n(q)$ is therefore fitted for each SADS. The validity of this correction is grounded in the Debye scattering equation (Supplementary Method 6). Since the atomic form factors are smooth and the self-scattering term (first term) cancels out in the difference signal, the molecular difference signal is dominated by the interference term (second term), which is inherently oscillatory due to the $\sin(qr_{ij})/qr_{ij}$ dependence. A lower-order polynomial baseline effectively captures the smooth, non-structural artefacts without distorting these characteristic high-frequency oscillations that determine the structural parameters. The parameters $a_n$ and $b_n$ were treated as free parameters and optimized simultaneously with the solute structural parameters. Further details of the baseline correction are described elsewhere[5]. $\sigma_n(q_j)$ represents the standard error of $\mathrm{SADS}_n^{\perp *}(q_j)$. The SADSs are obtained by the KCA of the difference scattering data based on the matrix formulation in equation (S7) of the Supplementary Methods, where the signal matrix is expressed as the matrix product of $\mathbf{X}^*(q)$ (the matrix containing the SADS vectors) and $\mathbf{M}^*(t)^\mathrm{T}$ (the transpose of the matrix containing the concentration profiles); in practice, at each $q$-point the time trace $\Delta S_\mathrm{iso}^{\perp}(q, t_k)$ is fitted as a linear combination of the species concentration profiles, with the $\mathrm{SADS}_n^{\perp *}(q_j)$ as the coefficients to be determined. The standard errors $\sigma_n(q_j)$ were estimated from parametric bootstrap realizations analogous to those used for the standard errors σn of the RSVs (Supplementary Method 1), except that the final step extracts the SADSs instead of the RSVs. Each RSV is a component of the data along the time axis, defined over the 136 pooled time delays, whereas each SADS is a component along the $q$ axis, defined over the 511 $q$-points; because the SADSs are resolved over many more points, a larger ensemble of $N = 1{,}000$ realizations was used, so that $\sigma_n(q_j)$ is well-converged at every $q$-point. For each realization, independent Gaussian noise of standard deviation $\sigma_\mathrm{iso}^{\perp}(q, t_k)$ was added to the raw (unsmoothed) difference scattering data, and the two-point time-pooling was applied, exactly as for the RSVs. The SADSs of that realization were then obtained by the KCA, using the species concentration profiles calculated from the time constants obtained from the fit of the RSVs (Supplementary Method 1). For each $n$ and $q_j$, the standard deviation of $\mathrm{SADS}_n^{\perp *}(q_j)$ across the $N$ realizations was taken as $\sigma_n(q_j)$. The reduced chi-square for the fit to the $n$-th SADS is then defined as:

$$\chi^2_{\nu,n} = \chi^2_n / \left(n_q - d_{\mathrm{f},n}\right) \tag{S13}$$

Here, $n_q$ = 511 is the number of $q$-points and $d_{f,n}$ is the number of free parameters in the fit to the $n$-th SADS: the $k$ structural motion parameters, the scaling coefficient, the mean-square-displacement parameter $\sigma^2$ where it is refined, and the two baseline coefficients $a_n$ and $b_n$ of $B_n(q)$. For the SSPM fits of $I_1$ and $I_2$, $d_{f,n} = k + 4$; for the refinement of the *cis*-AB structure, in which $\sigma^2$ is fixed, $d_{f,n} = k + 3$. For a given motion combination, $d_{f,n}$ enters the reduced chi-square only as a constant normalization, so the refined structure is independent of this count.

During the minimization process, various candidate structures for the reaction intermediates were generated by adjusting the structural parameters. For each candidate structure, the corresponding simulated PEPC-treated SADS, $\mathrm{SADS}_n'^{\perp}(q)$, was calculated, and its scaling factor (equation (S11)) was optimized to minimize the $\chi^2$ of equation (S12) against the experimental SADS. The method for calculating $\mathrm{SADS}'(q)$ and $\mathrm{SADS}'^{\perp}(q)$ is detailed in Supplementary Method 5. The optimal structural parameters were then determined by selecting the candidate structure with the lowest $\chi^2$ value. Further details on the structural parameters used in the fitting are provided in Methods section “Structure analysis”.

**Supplementary Method 4 | Error estimation for structural parameters**

To estimate the uncertainties associated with the structural parameters of the intermediate species, we employed the profile likelihood method[8,9]. We denote the $i$-th structural parameter by $p_i$. It can be either an angle or a distance, and the procedure below is identical in both cases. For each $p_i$, we scanned the parameter through a series of values across a scan range. At each scan point, $p_i$ was held fixed at that value while all the remaining free parameters were re-optimized by minimizing $\chi^2 + P_{\mathrm{steric}}$, the chi-square plus the steric penalty of Supplementary Method 12.2 (see below). The minimized value, taken as a function of $p_i$, is the profile likelihood $\chi^2_{\mathrm{profile}}(p_i)$. Because $P_{\mathrm{steric}}$ is zero unless the minimum inter-ring atomic distance is below 2.0 Å, $\chi^2_{\mathrm{profile}}(p_i)$ coincides with the $\chi^2$ for all physically admissible structures. The confidence interval for $p_i$ was then determined from the $\Delta\chi^2 = 1$ criterion[10]: the upper and lower crossing points correspond to the values of $p_i$ at which

$$\chi^2_{\mathrm{profile}}(p_i) - \chi^2_{\mathrm{min}} = 1 \tag{S14}$$

Here, $\chi^2_{\mathrm{min}}$ is the value of $\chi^2_{\mathrm{profile}}(p_i)$ at the reference minimum. The reference minimum is the local minimum reached from the reported value of $p_i$ by moving along the $p_i$ axis in the

direction of decreasing $\chi^2_{\text{profile}}(p_i)$. It is therefore the minimum of $\chi^2_{\text{profile}}(p_i)$ within the basin that contains the reported value. For most parameters the profile has a single minimum, which serves as the reference minimum; profiles with more than one minimum are treated later in this section.

This approach does not rely on a quadratic (parabolic) approximation of the $\chi^2$ surface, and it accounts for parameter correlations naturally, since the remaining parameters are free to adjust at every scan point. It also permits asymmetric error bars when the $\chi^2$ surface is asymmetric about the minimum. The re-optimization used the sequential quadratic programming algorithm with tight convergence criteria (optimality tolerance $10^{-8}$, step tolerance $10^{-10}$), and 30 scan points per direction, above and below the optimum, were evaluated for each parameter.

The refinement of *cis*-AB included a Tikhonov penalty towards the CASPT2 reference geometry (Supplementary Method 12.7). The profiles for *cis*-AB were computed with that Tikhonov penalty term excluded. In other words, the uncertainties reported for *cis*-AB contain no contribution from how far the refined geometry is from the CASPT2 reference. The steric penalty $P_{\text{steric}}$, in contrast, was retained, because every candidate conformation of azobenzene must satisfy it, whereas the Tikhonov term encodes prior knowledge of which geometry is expected.

For a parameter that was refined, the reference minimum was required to be within the bound imposed on that parameter during the refinement; the reported parameter value is within that bound, and so is the reference minimum reached from it. For a parameter that was held fixed, no such bound exists, and the whole scanned range was admissible. For most parameters, the $\chi^2_{\text{profile}}(p_i)$ rises monotonically on either side of the reference minimum until it reaches the $\Delta\chi^2 = 1$ level, and the parameter displacement from the reference minimum to each crossing point is then a direct measure of the precision of the reported solution.

For several parameters, the $\chi^2_{\text{profile}}(p_i)$ contained a further minimum in addition to the reference minimum. The quantity of interest is the steepness of $\chi^2_{\text{profile}}(p_i)$ about the reported solution. The scan, however, is not confined to the neighbourhood of that solution, because it explores a larger region of the parameter space than the refinement did. At every scan point, every parameter that the refinement varied is re-optimized alongside the displaced $p_i$, so the profile of a parameter that the refinement held fixed spans one more dimension than the refinement itself; and $p_i$ is varied across its full scan range, beyond any bound the refinement imposed on it. For *cis*-AB, the Tikhonov penalty term that was present in the structural refinement is

excluded when calculating the profiles; the structural refinements of $I_1$ and $I_2$ included no Tikhonov penalty term. A further solution reached in this way can even have a lower chi-square value than the reported one, because, for $I_1$ and $I_2$, the reported structure was selected in Supplementary Method 12 by the Bayesian information criterion, which penalizes model complexity in addition to the chi-square value. An uncertainty measured about a further solution would describe that structure rather than the reported one. Two rules ensure that the uncertainties quoted in Extended Data Table 3 are measured about the reported solution alone. First, a crossing point was retained only when no further minimum intervened between it and the reference minimum; a crossing point separated from the reference minimum by a further minimum was discarded. On a side where every crossing point was discarded, the uncertainty was determined by the one-sided convention stated below. The displacement from the reference minimum to a discarded crossing point is the sum of two terms: the separation between the reference minimum and the further minimum, and the displacement from the further minimum to the crossing point. Neither term describes the precision of the reported solution. This applies to the ∠CCNN of *cis*-AB. Its profile has a second minimum at 54.4°, which is lower in chi-square than the reference minimum by 21.3 but is outside the ±30° bound imposed on that parameter (Supplementary Method 12.4) and is therefore not eligible as the reference. That minimum lies between the reference minimum and the crossing point on the upper side (i.e., the direction of increasing $p_i$). Therefore, that crossing point was discarded, and only the uncertainty determined from the lower crossing point is reported.

Second, where the parameter values with $\Delta\chi^2 \leq 1$ formed two separate parameter intervals, one about the reference minimum and one about a further solution, both crossing points were measured within the interval containing the reported value. This occurred for the ∠CCNN and ∠CNN profiles of $I_2$. For ∠CCNN the floor of the further interval is within 0.02 in chi-square of the reference minimum, and for ∠CNN it is lower by 0.19; the barriers between the intervals rise about 50 and 5.0 above the floors, respectively. The barrier shows that the two intervals correspond to distinct solutions rather than to a single broad basin.

In most cases the displacements from the reference minimum to the two crossing points were not equal. The two could be reported separately as asymmetric uncertainties; we instead quoted the larger of the two, so that the reported uncertainty is a single value that is not smaller than either one-sided displacement. Where a crossing point was retained on only one side, we quoted the displacement from the reference minimum to that crossing point as the magnitude of the

uncertainty.

Some structural parameters were held fixed during the refinement, and the errors estimated for these parameters are marked with an asterisk in Extended Data Table 3. Their profiles were constructed in the same way. Because such a parameter was not refined, its reference minimum need not coincide with the value at which the parameter was held fixed. The quoted value therefore measures how tightly the data would constrain the parameter if it were released, and it does not indicate that the reported structure is displaced from the best fit.

**Supplementary Method 5 | Calculation of simulated SADSs**

The simulated SADS for the $n$-th species, denoted as $\text{SADS}_n'(q)$, was calculated as the sum of two distinct components: $\text{SADS}_{n,\text{solute}}'(q)$ and $\text{SADS}_{n,\text{cage}}'(q)$, which represent the simulated difference solute term and the simulated difference cage term, respectively. To calculate $\text{SADS}_{n,\text{solute}}'(q)$, the theoretical total solute scattering curves for both the reactant (*trans*-AB, $S_{trans,\text{solute}}'(q)$) and the transient $n$-th species ($S_{n,\text{solute}}'(q)$) were first calculated using the Debye scattering equation, based on the molecular structure of each species. Details of this calculation can be found in Supplementary Method 6. The difference between these two total solute terms was then calculated to yield $\text{SADS}_{n,\text{solute}}'(q)$, using the following equation: $\text{SADS}_{n,\text{solute}}'(q) = S_{n,\text{solute}}'(q) - S_{\text{trans,solute}}'(q)$. For $\text{SADS}_{n,\text{cage}}'(q)$, the theoretical total cage terms for both the reactant ($S_{\text{trans,cage}}'(q)$) and the transient $n$-th species ($S_{n,\text{cage}}'(q)$) were calculated. These cage terms were derived from molecular dynamics (MD) simulations, and $\text{SADS}_{n,\text{cage}}'(q)$ was obtained by calculating the difference between these two terms as follows: $\text{SADS}_{n,\text{cage}}'(q) = S_{n,\text{cage}}'(q) - S_{\text{trans,cage}}'(q)$. Details of the MD simulations, as well as the procedure for calculating the cage terms, are provided in Supplementary Method 9.

To enable direct comparison with the experimental SADS, which is in the solvent-independent space, $\text{SADS}_n'(q)$ were also subjected to PEPC treatment. As a result, the final equation for the PEPC-treated simulated SADS is expressed as follows:

$$\begin{aligned}\text{SADS}_n'^{\perp}(q) &= \left(\text{SADS}_n'(q)\right)^{\perp} \\ &= \left(\text{SADS}_{n,\text{solute}}'(q) + \text{SADS}_{n,\text{cage}}'(q)\right)^{\perp} \\ &= \text{SADS}_{n,\text{solute}}'^{\perp}(q) + \text{SADS}_{n,\text{cage}}'^{\perp}(q)\end{aligned} \tag{S15}$$

The equation for the scaled, PEPC-treated simulated SADS, $\text{SADS}_n'^{\perp *}(q)$, is expressed as:

$$\begin{aligned}\text{SADS}_1'^{\perp *}(q) &= \alpha \cdot \text{SADS}_1'^{\perp}(q) \\ \text{SADS}_2'^{\perp *}(q) &= \alpha \cdot \beta \cdot \text{SADS}_2'^{\perp}(q) \\ \text{SADS}_3'^{\perp *}(q) &= \alpha \cdot \beta \cdot \gamma \cdot \text{SADS}_3'^{\perp}(q)\end{aligned} \tag{S16}$$

It is important to note that the simulated curves in Fig. 3f, corresponding to $\text{SADS}_n'^{\perp *}(q)$, exhibit noisy patterns in $q$-space rather than being smooth. This is because the simulated curves in this figure were PEPC-treated to allow for a direct comparison with the PEPC-treated experimental curves. While the original simulated curves are smooth, the PEPC process involves applying known solvent components that were derived from a separate experiment

and contain inherent noise. As a result, when the original smooth simulated curves are transformed into the solvent-independent space via PEPC, the noise from the solvent component is introduced into the PEPC-treated simulated curves.

## Supplementary Method 6 | Debye scattering equation for simulating the scattering signal from solute structures

$S'_{n,\text{solute}}(q)$s were calculated using the Debye scattering equation based on the candidate structures. The Debye scattering equation calculates scattering intensities as a function of $q$, based on interatomic distances, which are determined from the molecular structures, and the X-ray atomic form factors[5,11-19]: $I(\text{q}) = \sum_i f_i^2 + \sum_i \sum_j f_i f_j \left(\sin(qr_{ij})/qr_{ij}\right)$ where $f_i(q)$ is the atomic form factor of the $i$-th atom and $r_{ij}$ is the interatomic distance between the $i$-th and $j$-th atoms.

Upon photoexcitation, molecules generally undergo a conversion of photon energy into thermal and kinetic energy during the course of the reaction, often leading to the activation of vibrational modes. This activation can broaden the distribution of interatomic distances within the molecule. To account for this broadening caused by vibrational motion in azobenzene, we used Debye–Waller factors (DWFs) in the analysis. The scattering signal arising from atomic pairs with broadened interatomic distance distributions due to thermal activation can be effectively described by incorporating the DWFs. When applying the DWF, the simulated scattering curves calculated using the Debye scattering equation were modified as follows:

$$S'_{n,\text{solute}}(q) = S'_{n,\text{solute,Debye}}(q) \times \text{e}^{\left(-\sigma^2 q^2/2\right)} \quad \text{(S17)}$$

Here, $\text{e}^{(-\sigma^2 q^2/2)}$ is the DWF, which damps the scattering intensity to account for the vibrational fluctuations of the interatomic distances; $\sigma^2$, the mean-square displacement of the interatomic distance, is used as a fitting parameter. $S'_{n,\text{solute,Debye}}(q)$ is the scattering intensity calculated using the Debye scattering equation for the static structure (without the DWF), and $S'_{n,\text{solute}}(q)$ is the intensity after the DWF is applied. We used a single $\sigma^2$ value for all atomic pairs within a molecule. This simplification was made to avoid overfitting, as assigning a separate $\sigma^2$ value to each atomic pair would significantly increase the number of fitting parameters and lead to severe overfitting. The DWF parameter $\sigma^2$ captures the excess vibrational disorder of each transient species: the nascent intermediates are likely vibrationally hot and therefore carry a

non-zero $\sigma^2$, whereas the equilibrium reactant and the fully relaxed product are expected to carry little or no excess vibrational energy and therefore have negligible $\sigma^2$. Therefore, $\sigma^2$ was fixed to 0 for the ground-state reactant *trans*-AB, which serves as the reference structure. The intermediates $I_1$ and $I_2$ are formed vibrationally hot and are probed before cooling is complete, so they sample a broad distribution of structures, and their $\sigma^2$ is expected to be nonzero; the refinement gave 0.017 ± 0.003 Å$^2$ for $I_1$ and 0.005 ± 0.002 Å$^2$ for $I_2$. *cis*-AB, by contrast, is the stable, thermally relaxed final product and is expected to be structurally well-defined, with a small $\sigma^2$ like that of the *trans*-AB reference. When $\sigma^2$ for *cis*-AB is allowed to vary, it refines to 0.023 ± 0.007 Å$^2$, which is larger even than the values obtained for the hot intermediates. Because such a broad structural distribution is not physically reasonable for a relaxed product, we fix $\sigma^2 = 0$ for *cis*-AB, on the same footing as the *trans*-AB reference. By explicitly incorporating the DWF, our analysis effectively decouples the vibrational effects from the structural changes. In this framework, the refined structural parameters define the average molecular geometry, whereas the $\sigma^2$ parameters capture the width of the vibrational ensemble around that centre. Therefore, the presence of vibrational broadening does not invalidate the identified parsimonious motions; rather, by explicitly accounting for it through the $\sigma^2$ parameter, the framework ensures that the refined structural parameters isolate the mean molecular geometry from the vibrational envelope surrounding it.

**Supplementary Method 7 | Equation for simulating the anisotropic scattering signal from solute structures**

The anisotropic component of the scattering signal, $S_{\text{aniso}}(q, t)$, was calculated using the standard expression commonly employed in TRXL studies[11,12,14-16,19]. Specifically, the signal was obtained as:

$$S_{\text{aniso}}(q) = -\sum_{n}\sum_{m} f_n(q) f_m(q) P_2[\cos \xi_{nm}] j_2[q r_{nm}] \tag{S18}$$

Here, $f_{\text{m}}(q)$ and $f_{\text{n}}(q)$ are the atomic form factors of atoms $m$ and $n$, respectively, $r_{\text{nm}}$ is the interatomic distance, $\xi_{nm}$ is the angle between the vector connecting atoms $m$ and $n$ and the laser polarization axis, $P_2$ is the second-order Legendre polynomial, and $j_2$ is the second-order spherical Bessel function. This expression accounts for the anisotropic orientational distribution of the molecules induced by photoselective excitation, which arises because the

excitation probability depends on the angle between the transition dipole moment of the molecule and the linear polarization axis of the pump laser. More detailed descriptions of this formalism can be found elsewhere[11,12,14-16,19].

When applied to the *trans*-AB structure, equation (S18) yields the simulated anisotropic scattering curve of *trans*-AB before PEPC treatment, $S_{\text{aniso},trans}'(q)$. Because the experimental $S_{\text{aniso}}(q, t)$ are subjected to the PEPC procedure to remove the solvent contribution, the identical PEPC projection was applied to this simulated curve. The resulting PEPC-treated simulated curve, $S^{\perp}_{\text{aniso},trans}'(q)$ (Extended Data Fig. 3e), is therefore directly comparable to the measured $S^{\perp}_{\text{aniso}}(q, t)$.

In the LCF analysis, the time-dependent contribution of $S^{\perp}_{\text{aniso},trans}'(q)$ to $S^{\perp}_{\text{aniso}}(q, t)$, denoted as $c(t)$, was determined at each time delay by a weighted linear least-squares projection of the measured $S^{\perp}_{\text{aniso}}(q, t)$ onto $S^{\perp}_{\text{aniso},trans}'(q)$; $c(t)$ is the value that minimizes the chi-square at that delay (Extended Data Fig. 3f). The shape of $S^{\perp}_{\text{aniso},trans}'(q)$ is governed by the orientation of the transition-dipole within the molecular frame, which is a global parameter common to all time delays, whereas $c(t)$ is the only time-dependent fitting parameter. The orientation of the transition dipole was optimized by minimizing the total chi-square, summed over all $q$ and $t$, between the measured $S^{\perp}_{\text{aniso}}(q, t)$ and the reconstruction $S^{\perp}_{\text{aniso},trans}'(q)\cdot c(t)$, which correspond to the Experiment and Simulation maps in Extended Data Fig. 3a, respectively. The DWF was not included here, because the anisotropic signal originates from the thermally cold, unreactive ground-state *trans*-AB ensemble, which carries no excess vibrational energy (mean-square atomic displacement of zero).

**Supplementary Method 8 | Atomic charge calculations for MD simulations**

Atomic partial charges for the azobenzene solute were determined through natural population analysis (NPA) performed on quantum-chemically computed electron densities. Two distinct levels of electronic structure theory were employed depending on the electronic state of the species. All calculations were performed using ORCA 6.0.1[20,21].

For the *trans*-AB and *cis*-AB structures in the electronic ground state, single-point calculations were carried out at the B3LYP-D3BJ/def2-TZVP level of theory with the SMD[22] implicit solvation model parameterized for methanol and the dynamic radii adjustment for

continuum solvation (DRACO) scheme[23], using the resolution-of-identity approximation with chain-of-spheres exchange (RIJCOSX) and the def2/J auxiliary basis set. The ground-state electron density was exported in MOLDEN format, and the NPA was performed using JANPA 2.02[24,25].

For the excited-state intermediates $I_1$ and $I_2$, which reside on the $S_1(n\pi^*)$ potential energy surface, the electronic structure was characterized using multi-configurational quantum chemical methods. State-specific complete active space self-consistent field (SS-CASSCF) calculations for the $S_1$ state were performed with a CAS(10,8) active space including the nitrogen lone pairs, azo π/π*, and phenyl π/π* orbitals, consistent with that used for the geometry optimizations in the main text. The aug-cc-pVDZ basis set was used for all atoms, with the SMD implicit solvation model parameterized for methanol to account for solvent polarization effects on the excited-state electron density. The DRACO scheme, which was applied in the ground-state calculations, was not used here because it is not currently compatible with CASSCF in ORCA. The RIJCOSX approximation was employed to accelerate the calculations. To ensure robust convergence of the state-specific CASSCF, which can be prone to root flipping (a change in the energetic ordering of the CASSCF roots during the orbital optimization that can switch the state being optimized), a multi-step procedure was employed, starting from B3LYP orbitals and proceeding through state-averaged CASSCF to the final state-specific solution. The converged solution was validated by confirming that the $S_1$ root retained its nπ* character and that adding dynamic correlation via strongly contracted N-electron valence state second-order perturbation theory (SC-NEVPT2) on top of the SS-CASSCF reference wavefunction did not change the energetic ordering of the electronic states, so that the nπ* state remained the lowest excited state ($S_1$). The natural population analysis (NPA) charges used in the subsequent MD simulations were derived from the state-specific $S_1$ electron density obtained with strongly contracted NEVPT2 (SC-NEVPT2) applied on top of the SS-CASSCF(10,8)/aug-cc-pVDZ reference. This density was exported in MOLDEN format and analysed with JANPA 2.02[24,25]. The NPA charges provide a physically meaningful partitioning of the electron density based on natural atomic orbital (NAO) occupancies, which is less sensitive to basis set size than Mulliken analysis and more robust than electrostatic-potential-based methods for molecules with buried atoms.

The charge calculations and MD simulations were performed within a self-consistent iterative framework. In the initial iteration, NPA charges were computed on the XMS-CASPT2-calculated geometries described in the main text, and these charges were used to parameterize

the solute for MD simulations in GROMACS (Supplementary Method 9). Solute–solvent pair distribution functions (PDFs) were extracted from the resulting trajectories and used to compute the cage term of the TRXL signal. The cage term was then employed in the TRXL structural refinement to yield updated solute geometries. In subsequent iterations, NPA charges were recomputed on the TRXL-refined structures at the same levels of theory described above, and the MD simulations, PDF extraction, cage term calculation, and TRXL refinement were repeated with the updated charges. This cycle was iterated until convergence was achieved, ensuring self-consistency between the solute partial charges, the solute–solvent PDFs, and the TRXL-refined molecular geometries.

**Supplementary Method 9 | MD simulations for simulating cage terms**

The solvent cage contribution, $S_{n,\mathrm{cage}}'(q)$, representing the contribution of the solvent–solute interatomic distances to the scattering signal, was calculated from PDFs obtained through classical MD simulations.[26,27] The simulations were performed using GROMACS 2024.4[28-31] under periodic boundary conditions.

For describing intermolecular interactions, we used the OPLS-AA (Optimized Potentials for Liquid Simulations, All-Atom) force field[32,33] for both solvent and solute molecules. The methanol solvent was modelled with the standard six-site all-atom OPLS-AA methanol model, which includes explicit Lennard-Jones and Coulomb interaction sites on each atom. For the azobenzene solute, standard OPLS-AA aromatic carbon and aromatic hydrogen atom types were used for the phenyl ring atoms. The azo nitrogen atoms were parameterized with Lennard-Jones parameters appropriate for aromatic nitrogen within the OPLS-AA framework. Bonded interaction parameters (bonds, angles, proper and improper dihedrals) were taken from the standard OPLS-AA aromatic parameter set. Atomic partial charges for the solute were assigned from the NPA calculations described above, providing state-specific charge distributions for each species (ground-state charges for *trans*-AB and *cis*-AB, $S_1$ excited-state charges for $I_1$ and $I_2$).

The solute was initialized at the target geometry (the XMS-CASPT2 geometry in the first self-consistency iteration and the TRXL-refined geometry thereafter). Throughout all simulation stages the solute and the methanol solvent were fully flexible and mobile, and no freeze groups or positional restraints were applied. The same free-solute treatment was applied to all four species (*trans*-AB, *cis*-AB, $I_1$, and $I_2$). Each simulation system consisted of one

azobenzene solute molecule centred in a cubic box of approximately 5.2 nm edge length, solvated with ~1,995 methanol molecules (approximately 12,000 atoms total). The methanol solvent box was pre-equilibrated for 500 ps at 298 K and 1 bar.

The production simulations were carried out in the NPT ensemble at 298 K and 1 bar. The protocol consisted of four stages: (1) energy minimization using the steepest descent algorithm with a force tolerance of 1,000 kJ/mol/nm; (2) NVT equilibration for 100 ps at 298 K using the velocity-rescale thermostat[34] with a coupling time constant of 0.1 ps; (3) NPT equilibration for 200 ps at 298 K and 1 bar using the velocity-rescale thermostat together with the cell-rescale barostat[35] with a coupling time constant of 2.0 ps; and (4) an NPT production run of 1 ns using the velocity-rescale thermostat and the cell-rescale barostat, saving coordinates every 0.2 ps. A 2-fs integration time step was used with all bonds involving hydrogen atoms constrained via the LINCS algorithm[36]. Electrostatic interactions were computed using the Particle Mesh Ewald (PME) method[37] with a real-space cutoff of 1.2 nm. Van der Waals interactions were truncated at 1.2 nm with long-range dispersion corrections applied to both energy and pressure.

For each solute species, 30 independent 1-ns production runs were performed, each initialized with a different random velocity seed (seeds 10,001–10,030), yielding 5,000 coordinate frames per run and a cumulative sampling of 30 ns. The use of independent short trajectories with distinct initial conditions, rather than a single continuous trajectory, provides statistically independent samples for ensemble averaging and enables straightforward assessment of convergence. Site-site PDFs $g_{ij}(r)$ were computed independently from each of the 30 trajectories and then averaged. Convergence was assessed by monitoring the variation of the averaged PDFs as a function of the number of included trajectories; the 30-run average was found to be well converged for all solute–solvent site pairs.

The PDFs were computed for all unique pairs among 28 interaction sites: 24 solute atom sites (12 C, 10 H, 2 N of azobenzene) and 4 solvent site types (C, O, $H_{\mathrm{methyl}}$, $H_{\mathrm{hydroxyl}}$ of methanol), yielding 406 unique site-site PDFs per species (comprising 300 solute–solute, 96 solute–solvent, and 10 solvent–solvent pairs). A bin width of 0.02 Å and a maximum radial distance of 40 Å were used.

The cage-term scattering signal $S_{n,\mathrm{cage}}'(q)$ was then computed from the solute–solvent PDFs using the Debye scattering equation formalism[5,11-19,26,27,38-40]. For each solute–solvent site pair $(i, j)$, the scattering contribution is given by:

$$S_{ij}(q) = \left(N_i N_j / V\right) \cdot f_i(q) f_j(q) \cdot 4\pi \int_{r=0}^{r_{\max}} r\,[\sin(qr)/q]\left[g_{ij}(r) - 1\right] dr \qquad \text{(S19)}$$

Here, $N_i$ and $N_j$ are the numbers of atoms of types $i$ and $j$ in the simulation box, $V$ is the box volume, $f_i(q)$ and $f_j(q)$ are the X-ray atomic form factors, $r_{\max}$ is the upper limit of the radial integration, and $g_{ij}(r)$ is the site-site PDF between types $i$ and $j$. The constant 1 subtracted from $g_{ij}(r)$ in the integrand is its asymptotic value at large $r$, which is unity for intermolecular pairs. Because the cage term describes solute–solvent structural correlations, only the 96 solute–solvent site pairs (24 solute sites × 4 solvent site types) were used; the total cage signal was obtained by summing these contributions, with a factor of 2 applied for cross-pair terms. In evaluating equation (S19), the sine transform was computed numerically from the individual $g_{ij}(r)$ functions, which are statistically noisy; two measures were therefore applied to suppress the spurious fluctuations that these two factors would otherwise introduce into the cage term. First, the radial integral was carried out to an upper limit of $r_{\max}$ = 25 Å; because every site–site PDF $g_{ij}(r)$ has already reached its asymptotic value within this range, the finite integration range introduces negligible termination ripple, and a standard sine transform with kernel $r \cdot \sin(qr)$ was therefore used without any Lorch or window modification function. Second, and independently of the choice of $r_{\max}$, the statistical noise in each $g_{ij}(r)$ at large $r$ was suppressed by setting $g_{ij}(r)$ to its asymptotic value at all radii beyond the point where the running mean of $|g_{ij}(r) - 1|$ over a 2 Å window had settled below a fixed $2\sigma$ threshold (0.02, where $\sigma \approx 0.01$ is the noise floor of the $g_{ij}(r)$ functions around their asymptotic value observed in the simulations). The resulting $S_{n,\mathrm{cage}}'(q)$ curves were used in the structural refinement to calculate the simulated species-associated difference scattering curves ($\mathrm{SADS}_n'(q)$s).

To ensure self-consistency between the solute structure, atomic charges, and solute–solvent PDFs, the MD simulations and subsequent cage-term calculations were embedded within the iterative framework described in the preceding section on atomic charge calculations. In each iteration, the NPA charges and solute geometries were updated based on the latest TRXL-refined structures, and the MD simulations were repeated until convergence was achieved.

**Supplementary Method 10 | Fourier sine transform of SADSs in *q*-space to retrieve structural information in *r*-space**

X-ray scattering curves in reciprocal space, denoted as $S(q)$, can be transformed into radial distribution functions (RDFs) in real space, $R(r)$, using a Fourier sine transform (FST). The FST applied in this study follows the relation:

$$r^2 \Delta R(r) = r/(2\pi^2) \int q\Delta S(q) \cdot \exp(-\eta q^2) \cdot \sin(qr)\, dq \qquad \text{(S20)}$$

In this equation, $\eta$ is a Gaussian damping factor that suppresses truncation artefacts arising from the finite $q$-range of the experimental data. The damping factor was set to $\eta = 0.03$ Å$^2$, which yields $\exp(-\eta q^2) \approx 0.23$ at $q_{\text{max}} = 7$ Å$^{-1}$, retaining the majority of the measured scattering information while adequately suppressing high-$q$ truncation ripples. This procedure follows standard practices in TRXL studies, with further details available elsewhere[5,11-19,26,27,38-40]. In some of our previous publications, $S(r)$ was used in place of $R(r)$. Here, we instead use $R(r)$ for greater mathematical rigour. The resulting $r^2\Delta R(r)$ representation provides a more intuitive way to understand molecular structural changes, as the interatomic pair distances are directly represented in the real-space distribution, offering straightforward insights into which distances are formed or depleted during the reaction.

This transform can be applied not only to $S(q)$ but also to difference scattering curves. When applied to difference scattering curves, the positive amplitudes of the resulting difference RDFs reflect newly formed interatomic distances, while the negative amplitudes indicate depleted interatomic distances that arise during the course of the reaction. These difference RDFs capture atomic pair distances not only within solute molecules but also between solute and solvent molecules, and among solvent molecules. We note that to extract an accurate difference RDF from PEPC-treated difference scattering curves, it is essential to first correct the PEPC-treated difference scattering signals in $q$-space before applying the FST. This step is necessary because the PEPC process can distort the shape of the difference scattering curves during their projection into a space orthogonal to the solvent terms (Supplementary Fig. 6). The methodology used for correcting such distortions is detailed elsewhere[5,7]. Following established procedures, both the SADS($q$)s and SADS′($q$)s were corrected to yield PEPC-corrected curves: SADS$(q)^{\text{cor}}$s and SADS′$(q)^{\text{cor}}$s.

The PEPC-corrected SADSs represent the solute-related term, $\Delta S_{\text{solute-related}}(q) = \Delta S_{\text{solute}}(q) + \Delta S_{\text{cage}}(q)$. Applying the FST to the solute-related term yields information in $r$-space

that reflects both changes within the solute's molecular structure and changes in the atomic pair distances between solute and solvent molecules. However, our primary interest lies in the solute's structural changes, that is, the changes in atomic pair distances within the solute molecules. To focus on these solute-specific structural changes, we subtracted the cage contribution from the PEPC-corrected SADSs in $q$-space, yielding solute-only experimental and simulated PEPC-corrected SADSs, referred to as $\mathrm{SADS_{solute}}(q)^{\mathrm{cor}}$s and $\mathrm{SADS_{solute}}'(q)^{\mathrm{cor}}$s, respectively. These solute-only SADSs were then subjected to the FST to extract atomic pair distance information exclusively related to the solute's structural dynamics. The FST of $\mathrm{SADS_{solute}}(q)^{\mathrm{cor}}$ and $\mathrm{SADS_{solute}}'(q)^{\mathrm{cor}}$ yielded the experimental and simulated species-associated RDFs, referred to as $\mathrm{SADS_{solute}}(r)$ and $\mathrm{SADS_{solute}}'(r)$, respectively. The $\mathrm{SADS_{solute}}(r)$s and $\mathrm{SADS_{solute}}'(r)$s obtained from this FST are presented in Extended Data Fig. 5. These real-space representations provide a clearer understanding of how specific interatomic distances emerge and disappear throughout the course of the reaction.

**Supplementary Method 11 | Validating the kinetic model through linear combination fitting analysis**

If the established kinetic model is valid, the time-dependent concentration changes of the species, calculated based on the model, should accurately describe the experimentally extracted concentration profiles of the species. To verify this, we determined the concentration changes of the species from the experimental data. These concentrations were extracted using linear combination fitting (LCF), which is based on the following relationship.

$$\Delta S_{\mathrm{iso}}^{\perp}(q,\, t) \approx \Delta S_{\mathrm{iso,LCF}}^{\perp}(q,\, t) = \sum_{n=1}^{3} \mathrm{SADS}_{n}^{\perp}(q) \times C_{\mathrm{SADS},n}(t) \qquad \text{(S21)}$$

$\Delta S_{\mathrm{iso,LCF}}{}^{\perp}(q)$ is the solvent-independent isotropic difference scattering data reconstructed as a linear combination of the $\mathrm{SADSs}^{\perp}$, and $\mathrm{C_{SADS,}}_{n}(t)$ denotes the weighting factor for the $n$-th $\mathrm{SADS}^{\perp}$ in this linear combination, which is proportional to the concentration of the species at time $t$.

Then, the optimal concentration factors, $\mathrm{C_{SADS,}}_{n}(t)$, were obtained by minimizing the chi-square value ($\chi^2{}_t$), which quantifies the discrepancy between the experimental and the fitted linear combination ($\Delta S_{\mathrm{iso,LCF}}{}^{\perp}(q)$) data at each time delay. $\chi^2{}_t$ is defined as follows.

$$\sum_q \left(\left(\Delta S_{\text{iso}}^{\perp}(q,\, t) - \Delta S_{\text{iso,LCF}}^{\perp}(q,\, t)\right) / \sigma(q,\, t)\right)^2 = \chi_t^2 \tag{S22}$$

Here, $\sigma(q, t)$ represents the standard error of the mean for $\Delta S_{\text{iso}}^{\perp}(q, t)$ at time delay $t$. The values of $C_{\text{SADS,n}}(t)$ are shown as open circles in Fig. 3e. In Fig. 3e, the optimal concentration profile and the concentration profile derived from the kinetic model show good agreement, confirming that our kinetic model accurately describes the structural dynamics of *trans*-AB.

**Supplementary Method 12 | Systematic Scan for Parsimonious Motions (SSPM) to characterize structural motions**

**12.1 | SSPM Overview**

In conventional SADS analyses[5,17,41], all structural parameters, or equivalently all possible motions, are typically allowed to vary simultaneously to minimize the $\chi^2$ value. However, this approach makes it difficult to identify which motions contribute most to the structural evolution of the intermediates, because the $\chi^2$ score decreases whenever an additional structural degree of freedom is introduced; this happens even when the added parameter is not chemically or quantum-mechanically meaningful. This also increases the risk of overfitting, in which additional degrees of freedom improve the apparent fit without providing physically meaningful structural information. To overcome this problem, rather than considering every mathematically possible motion, we aimed to describe the intermediates using only motions with clear physical relevance to the classical *trans* → *cis* isomerization mechanisms. We selected the following four representative motions (Fig. 1a): rotation (ROT), inversion (INV), N–N bond length change (NND), and C–N bond torsion (CNT). We considered not only the motions individually but also their possible combinations. Specifically, we examined $\chi^2$ distributions for intermediates modelled with single motions (4 cases), pairs (6 cases), triples (4 cases), and all four combined (1 case), for a total of 15 cases. We then evaluated the Bayesian Information Criterion (BIC), which penalizes model complexity to prevent over-parameterization, to identify the most parsimonious model. We refer to this systematic scan of all possible motion combinations using $\chi^2$ and BIC as Systematic Scan for Parsimonious Motions (SSPM).

The model yielding the minimum BIC was identified as the optimal parsimonious fit to the data without overfitting. A detailed formulation of the $\chi^2$-based BIC and the determination of the effective number of data points ($N_{\mathrm{eff}}$) are provided in Supplementary Method 13. Throughout this section, the total number of free parameters in each SSPM fit is denoted $k + 4$, where $k$ is the number of active structural (motional) parameters and the four additional parameters are the mean-square displacement parameter $\sigma^2$, the scaling coefficient, and the two baseline coefficients $a_n$ and $b_n$. The parameter $\sigma^2$ is incorporated into the simulated scattering curves through an isotropic DWF of the form $\exp(-\sigma^2 q^2/2)$, which accounts for thermal broadening of interatomic distances. As detailed in the following subsections, SSPM was applied to $SADS_1$ (for $I_1$) and $SADS_2$ (for $I_2$), but not to $SADS_3$ (for *cis*-AB); the *cis*-AB

structure was instead refined by a direct two-parameter adjustment of the CASPT2-calculated *cis*-AB geometry (Section 12.4). Nevertheless, the $SADS_3$ refinement is included in this section because the scaling coefficients are determined sequentially in the order $SADS_3 \rightarrow SADS_1 \rightarrow SADS_2$ (Section 12.3): the composite scaling factor $\alpha \cdot \beta \cdot \gamma$ obtained from the structural refinement against $SADS_3$ provides an experimentally determined lower bound for the subsequent SSPM fits of $SADS_1$ and $SADS_2$. Sections 12.4 through 12.6 therefore follow this refinement order so that each step can reference the scaling constraints established in the preceding step.

### 12.2 | Structural parameter bounds

In SSPM, the structural parameters were allowed to vary within physically reasonable ranges. The bounds for ROT (Δ∠CNNC) and CNT (Δ∠CCNN) were set to −180° to 180° for all transitions, covering the full angular range. For NND ($\Delta r_{NN}$), the range was −0.012 to +0.100 Å, where the lower bound of −0.012 Å corresponds to the shortest N–N distance obtained from CASPT2 calculations (1.264 Å) relative to the *trans*-AB reference value of 1.276 Å. The bounds for the INV angles (Δ∠CNN) were adapted to each transition. For the *trans*-AB → $I_1$ transition, the range was restricted to 0° to 30°, because the starting *trans*-AB geometry is nearly planar and the structural change towards $I_1$ is expected to be moderate. For $I_1 \rightarrow I_2$, a wider range of −120° to 120° was adopted to accommodate the possibility of larger conformational changes without unnecessarily constraining the optimization. In all cases, steric clashes were prevented during the fit: candidate structures in which the two phenyl rings approached too closely were penalized (for the *cis*-AB refinement, a penalty, $P_{steric}$, was added to the chi-square whenever the minimum inter-ring atomic distance fell below 2.0 Å) or excluded. As a result, all refined structures are free of steric overlap and have chemically plausible bond geometries.

### 12.3 | Sequential refinement strategy and scaling factor determination

Within each SADS fit, the scaling factor (concentration coefficient) and the structural parameters were optimized simultaneously because they are coupled through the amplitude of the fitted signal. The experimental SADS is reproduced as the product of the scaling factor and a simulated difference-scattering curve calculated from the candidate structure. Because the experimental SADS is fixed, the scaling factor and the amplitude of the simulated curve are

inversely related, so that a larger scaling factor requires a simulated curve of smaller amplitude and a smaller scaling factor a curve of larger amplitude. Since the amplitude of the simulated curve generally increases with the magnitude of the structural change, the scaling factor and the structural parameters are coupled accordingly, and fixing either one in advance would bias the other. Therefore, the scaling factor and the structural parameters were refined together rather than sequentially.

The three experimental SADSs were refined sequentially in the order $SADS_3$ → $SADS_1$ → $SADS_2$ to progressively constrain the scaling factors $\alpha$, $\beta$, and $\gamma$, where $\alpha$ is the concentration coefficient for $I_1$ formation, defined as the number of *trans*-AB molecules forming $I_1$ upon photoexcitation divided by the number of solvent molecules, $\beta$ is the branching fraction of $I_1$ molecules that proceed to $I_2$, and $\gamma$ is the branching fraction of $I_2$ molecules that convert to *cis*-AB. The scaling factors relate the experimentally observed SADSs to the unit-concentration theoretical SADSs as $SADS_1^{\perp *}(q) = \alpha \cdot SADS_1'^{\perp}(q)$, $SADS_2^{\perp *}(q) = \alpha \cdot \beta \cdot SADS_2'^{\perp}(q)$, and $SADS_3^{\perp *}(q) = \alpha \cdot \beta \cdot \gamma \cdot SADS_3'^{\perp}(q)$, where $SADS_n^{\perp *}(q)$ denotes the experimentally observed (amplitude-scaled) signal and $SADS_n'^{\perp}(q)$ the unit-concentration difference scattering curve computed from the structural model (Supplementary Method 5).

The refinement proceeded in three sequential steps. First, $SADS_3$ was refined to determine the *cis*-AB structure and the composite scaling factor $\alpha \cdot \beta \cdot \gamma = (3.339 \pm 0.062) \times 10^{-4}$ (Section 12.4). Since $0 \leq \beta \leq 1$ and $0 \leq \gamma \leq 1$, the inequality $\alpha \cdot \beta \cdot \gamma \leq \alpha \cdot \beta \leq \alpha$ provides physically motivated lower bounds for the subsequent steps. Second, the SSPM for the *trans*-AB → $I_1$ transition determined the $I_1$ structure and $\alpha = (3.655 \pm 0.629) \times 10^{-4}$, subject to the constraint $\alpha \geq \alpha \cdot \beta \cdot \gamma$ (Section 12.5). Third, the SSPM for the $I_1 \rightarrow I_2$ transition determined the $I_2$ structure and $\alpha \cdot \beta = (3.655 \pm 0.287) \times 10^{-4}$, bounded by $\alpha \cdot \beta \cdot \gamma \leq \alpha \cdot \beta \leq \alpha$ (Section 12.6). The individual branching fractions were then obtained as $\beta = (\alpha \cdot \beta) / \alpha = (1.00 \pm 0.19)$ and $\gamma = (\alpha \cdot \beta \cdot \gamma) / (\alpha \cdot \beta) = (0.91 \pm 0.07)$, indicating that 100 ± 19% of $I_1$ molecules proceed to $I_2$ (with a time constant of 2.7 ps) and 91 ± 7% of the resulting $I_2$ molecules subsequently convert to *cis*-AB. The sub-unity central value of $\gamma$ suggests that a minor fraction (~9%) of the $I_2$ population may return to the *trans*-AB ground state via a competing pathway. In this framework, the observed time constant of 14 ps for the decay of $I_2$ is an apparent value reflecting the sum of the rate constants forming *cis*-AB and *trans*-AB ($k_{obs} = k_{cis} + k_{trans}$), rather than the intrinsic rate of the productive $I_2$ → *cis*-AB channel alone. A detailed decomposition into intrinsic rate constants and a comparison with spectroscopic quantum yield measurements

are provided in Supplementary Note 10.

### 12.4 | Structural refinement of *cis*-AB (SADS3)

The structure of *cis*-AB was determined by direct refinement against the experimental $SADS_3$, rather than by applying motions from the $I_2$ intermediate via SSPM (as done for $I_1$ and $I_2$ in Sections 12.5 and 12.6). This approach was adopted because: (i) *cis*-AB is a ground-state ($S_0$) product for which CASPT2 provides a reliable reference geometry, unlike the excited-state intermediates $I_1$ and $I_2$ where multi-reference character complicates the theoretical prediction; (ii) The large conformational difference between $I_2$ (∠CNNC ≈ 116°) and *cis*-AB (∠CNNC ≈ 11°) makes the $I_2$ → *cis*-AB fitting landscape contain multiple local minima. An SSPM starting from $I_2$ would require extensive regularization and the result would be difficult to validate independently; (iii) The experimental $SADS_3$ directly encodes the *cis*-AB – *trans*-AB difference signal. Starting from the well-characterized CASPT2 *cis*-AB geometry and refining a minimal number of parameters provides the most robust and interpretable result.

Starting from the CASPT2-calculated *cis*-AB geometry, two structural parameters were refined: the ∠CNNC dihedral angle and the ∠CCNN angle. These two angles were selected because they exhibit the largest changes between the CASPT2-calculated *trans*-AB and *cis*-AB structures, whereas other structural parameters (the ∠CNN inversion angles, $r_{NN}$, $r_{CN}$, ∠CCCN improper angles, and ∠CCN angles) show only small changes (Extended Data Table 3) and were therefore fixed at their CASPT2 values during refinement. $C_2$ symmetry (with the $C_2$ axis perpendicular to N–N) was imposed, reflecting the symmetry of the equilibrium *cis*-AB structure ($\Delta\angle CCNN_1 = \Delta\angle CCNN_2$, one free parameter). During the refinement, the two structural parameters were varied relative to their CASPT2-calculated values, Δ∠CNNC and Δ∠CCNN, the quantities listed in Extended Data Table 3. Δ∠CCNN was restricted to ±30°, so that the refined orientation of the phenyl rings remained close to the CASPT2-calculated orientation, which reason (i) above establishes as a reliable reference; Δ∠CNNC was not restricted. The mean-square displacement parameter $\sigma^2$ was fixed at zero, because *cis*-AB is a long-lived ground-state product (formed with a time constant of 14 ps) that has sufficient time to relax vibrationally to thermal equilibrium before contributing to the measured $SADS_3$. Together with the scaling coefficient ($= \alpha \cdot \beta \cdot \gamma$) and the two baseline coefficients $a_n$ and $b_n$, this gives a total of $k + 3 = 5$ free parameters ($k = 2$ motional parameters; $\sigma^2$ is fixed). The objective function was a Tikhonov-regularized $\chi^2$:

$$F = \chi^2 + \lambda \cdot \mathrm{RMSD}^2(\mathbf{r}_{\mathrm{fitted}}, \mathbf{r}_{\mathrm{CASPT2}}) \quad \text{(S23)}$$

$\mathbf{r}_{\mathrm{CASPT2}}$ is the CASPT2-calculated *cis*-AB geometry, $\mathbf{r}_{\mathrm{fitted}}$ is the candidate *cis*-AB structure generated by applying the basis motions to $\mathbf{r}_{\mathrm{CASPT2}}$, and RMSD is computed over all 24 atoms after optimal Kabsch superposition. The regularization strength $\lambda$ = 7.5 Å$^{-2}$ was determined by evidence maximization (Section 12.7). The *trans*-AB reference structure was fixed at its CASPT2 geometry throughout, and the simulated $\mathrm{SADS}_3$ was computed as the difference between the Debye scattering curves of the candidate *cis*-AB and the reference *trans*-AB, with solvent cage terms included for both species. The simulated SADS was then subjected to the same PEPC projection as the experimental data to ensure that the comparison between experiment and simulation is performed on a consistent basis.

Starting from the zero-motion CASPT2 reference ($\chi^2$ = 610.6, $\chi_v^2$ = 1.20), the two-parameter refinement reduced $\chi^2$ to 492.8 ($\chi_v^2$ = 0.97), a 19% improvement, with a concentration coefficient $\alpha \cdot \beta \cdot \gamma$ = (3.339 ± 0.062) × 10$^{-4}$. The refined structure deviates from the CASPT2 reference primarily in the ∠CCNN angle (Δ∠CCNN = 20.9° ± 4.4°), with a modest change in ∠CNNC (Δ∠CNNC = 4.8° ± 7.4°). The RMSD from the CASPT2 reference (0.52 Å) is consistent with a structural refinement rather than a major rearrangement, confirming that the CASPT2-calculated *cis*-AB structure serves as a reliable starting point. The full set of refined structural parameters is listed in Extended Data Table 3. The profile likelihood of the ∠CCNN displacement contains two minima: the reference minimum at 21.6°, which lies within the ±30° bound, and a second minimum at 54.4°, which lies 24.4° outside the bound imposed in the refinement and is therefore not an alternative solution of this refinement. Because this minimum intervenes between the reference minimum and the crossing point on that side, the crossing point was discarded, following the convention stated in Supplementary Method 4. A crossing point was retained on only one side, at 17.2°, and an uncertainty of 4.4° was quoted.

### 12.5 | *trans*-AB → $\mathbf{I_1}$ (SSPM)

SSPM was used for fitting $\mathrm{SADS}_1$. Starting from the CASPT2-calculated *trans*-AB geometry, inversion symmetry ($C_i$) was imposed, reflecting one of the symmetry elements of the $C_{2h}$ point group of the *trans*-AB starting geometry. For comparison, we also tested unconstrained refinement, $C_2$-constrained, and 90°-fixed geometries (see below). Under the $C_i$ constraint, the two CNN bending angles are linked ($\angle\mathrm{CNN}_1 = \angle\mathrm{CNN}_2$, one free parameter) and the two CCNN

angles are equal in magnitude but opposite in sign ($\angle CCNN_1 = -\angle CCNN_2$, one free parameter), halving the number of free parameters for INV and CNT from two to one each. The objective function was the unregularized $\chi^2$ between the experimental $SADS_1$ and the simulated $SADS_1$ derived from the candidate $I_1$ structure; no regularization was necessary, as the *trans*-AB → $I_1$ transition involves a moderate conformational change and the fitting landscape is well-behaved. The scaling factor $\alpha$ was optimized simultaneously, subject to the constraint $\alpha \geq \alpha \cdot \beta \cdot \gamma$ (Section 12.3).

The $C_i$ symmetry was validated by comparing the BIC values obtained with and without the symmetry constraint. Both analyses identified CNT as the BIC-best model, but the $C_i$-constrained fit achieved a lower BIC (146.6 vs 151.2 for the unconstrained fit) because the symmetry constraint reduces the number of free parameters ($k + 4 = 5$ vs 6) while incurring only a marginal increase in $\chi^2$. We also tested two alternative symmetry constraints: $C_2$ symmetry ($\angle CCNN_1 = \angle CCNN_2$) and a fixed constraint ($\angle CCNN_1 = \angle CCNN_2 = 90°$). All three variants identified CNT as the BIC-best model, and the BIC differences among the three symmetry variants were less than 2, indicating that the data cannot statistically distinguish among these symmetry assumptions (see Supplementary Note 3 for a detailed discussion). Given that the $C_i$-constrained fit yields $\angle CCNN = \pm 87.0° \pm 14.0°$, a range that encompasses 90°, we consider the $I_1$ structure to be effectively described by $\angle CCNN_1 = \angle CCNN_2 \approx 90°$, at which the $C_i$ and $C_2$ constraints become equivalent and the molecule retains approximate $C_{2h}$ symmetry.

### 12.6 | $I_1$ → $I_2$ (SSPM)

Starting from the converged $I_1$ geometry obtained in Section 12.5, we performed the SSPM for the $I_1$ → $I_2$ transition under two parameterizations, in order to test whether $I_2$ retains the symmetry of $I_1$. In the first, we imposed the inversion (*i*) symmetry that had been selected as the best-fitting model for the *trans*-AB → $I_1$ transition ($\angle CCNN_1 = -\angle CCNN_2$, Section 12.5). In the second, we removed all symmetry constraints and varied every structural parameter independently. In both cases the objective function was the unregularized $\chi^2$ between the experimental $SADS_2$ and the simulated $SADS_2$, and the scaling factor $\alpha \cdot \beta$ was optimized simultaneously, bounded by $\alpha \cdot \beta \cdot \gamma \leq \alpha \cdot \beta \leq \alpha$ (Section 12.3).

The unconstrained parameterization is strongly preferred. Its best model, ROT + CNT ($k + 4 = 7$), reaches the absolute minimum BIC($\chi^2$) = 201.4. Imposing the $C_i$-constraint on this

same ROT + CNT model raises its BIC($\chi^2$) to 227.1, a substantial worsening. The data therefore do not support the inversion-symmetric description for $I_2$. Rather than being imposed, the symmetry of $I_2$ is revealed by the unconstrained fit: the two ∠CCNN angles, refined independently, converge to equal values of the same sign (∠$CCNN_1$ = ∠$CCNN_2$ = −71.9°, differing by less than 0.01°). Equal angles of the same sign are the signature of $C_2$ symmetry (∠$CCNN_1$ = ∠$CCNN_2$), whereas the $C_i$ description requires equal angles of opposite sign (∠$CCNN_1$ = −∠$CCNN_2$).

The difference between the $C_2$ symmetry of $I_2$ and the higher $C_{2h}$ symmetry of $I_1$ lies in the inversion centre, and this is set by the ∠CNNC dihedral. In $I_1$, ∠CNNC = 180°, which is compatible with an inversion centre; in $I_2$, it refines to 115.5° (Extended Data Table 3). Because an inversion centre requires ∠CNNC = 0° or 180° (inversion maps the dihedral onto its negative), a value of 115.5° is incompatible with it. $I_2$ therefore retains the two-fold axis but not the inversion centre, so its symmetry is $C_2$ rather than the approximate $C_{2h}$ of $I_1$, as discussed in detail in Supplementary Note 3.

**12.7 | Tikhonov regularization for the $SADS_3$ *cis*-AB refinement**

The *cis*-AB geometry was determined through the refinement against $SADS_3$ described in Section 12.4; no separate $I_2$ → *cis*-AB SSPM was performed. This refinement employed Tikhonov regularization towards the CASPT2 reference geometry. Because *cis*-AB is a ground-state ($S_0$) product, its equilibrium geometry is well described by CASPT2. In general, CASPT2 geometries can be less reliable for excited-state species, where multi-reference character, conical-intersection proximity, and non-adiabatic dynamics complicate the calculation; however, these issues do not arise for a ground-state molecule such as *cis*-AB. The regularization term penalizes large deviations from this well-characterized reference, preventing the optimizer from settling into unphysical local minima while still allowing the experimental scattering data to refine the structure where the data warrant it. The validity of this approach is further supported by the refinement results (Section 12.4), which showed that the CASPT2 geometry requires only a two-parameter adjustment to match the experimental $SADS_3$ with a $\chi^2$ improvement of 19%. In contrast, no regularization was applied to the *trans*-AB → $I_1$ and $I_1$ → $I_2$ transitions (Sections 12.5 and 12.6), as the corresponding intermediates ($I_1$ and $I_2$) reside on the $S_1$ excited-state surface, where multi-reference character and conical-intersection proximity render CASPT2 geometries less reliable as structural priors.

The regularization strength $\lambda$ controls the balance between fidelity to the experimental data ($\chi^2$) and proximity to the CASPT2 reference structure ($RMSD^2$). An optimal $\lambda$ should be large enough to exclude unphysical solutions, but small enough to allow the data to drive the structural determination. The regularization strength $\lambda$ was determined by the Bayesian method of evidence maximization following MacKay[42]. Here, the penalty term $\lambda \cdot RMSD^2$ is equivalent to a Gaussian prior distribution on the deviation of the structure from the CASPT2 reference, with a width set by $\lambda$; each value of $\lambda$ therefore defines a complete statistical model of the data. Models with different values of $\lambda$ are compared through the marginal likelihood (evidence) $P(D|\lambda)$, the probability of the observed data D under the model with a given $\lambda$, computed by averaging the likelihood of the data over all values of the structural parameters, weighted by the prior. When $\lambda$ is too small, the prior admits a wide range of structures, most of which fit the data poorly, and the evidence is low. When $\lambda$ is too large, the prior confines the structure to the immediate neighbourhood of the reference, the deviations favoured by the data become inaccessible, and the evidence is again low. The evidence therefore reaches its maximum at an intermediate value of $\lambda$, at which the prior is neither too permissive nor too restrictive. We evaluated $\ln P(D|\lambda)$ using the Laplace approximation:

$$\ln P\,(D|\lambda) \approx -1/2 \cdot F(\hat{\boldsymbol{p}}) + (k_{total}/2) \cdot \ln(2\pi) - 1/2 \cdot \ln(\det \mathbf{H}) + (m/2) \cdot \ln(\lambda/2\pi) \tag{S24}$$

$F(\hat{\boldsymbol{p}})$ is the regularized objective function of Eq. (S23) evaluated at the optimal parameter vector $\hat{\boldsymbol{p}}$, $\mathbf{H}$ is the Hessian matrix of $F(\hat{\boldsymbol{p}})$, $k_{total}$ is the total number of free parameters ($k_{total} = k + 3$), and $m$ is the number of regularized structural parameters. The Hessian was computed numerically by finite differences. We first scanned $\lambda$ over 25 logarithmically spaced values from $10^{-8}$ to $10^{4}$ Å$^{-2}$, and then refined the position of the maximum of the evidence with two further rounds of sampling (5 points each, centred on the best value of the preceding round). This procedure returned a maximum at $\lambda = 7.5$ Å$^{-2}$, which was adopted for all subsequent analyses. The $\ln P(D|\lambda)$ function in equation (S24) is relatively flat around its maximum. Specifically, $\ln P(D|\lambda)$ changes by less than 0.1 between $\lambda = 5$ Å$^{-2}$ and $\lambda = 10$ Å$^{-2}$, indicating that the optimal $\lambda$ is not sharply determined within this range. At $\lambda = 7.5$ Å$^{-2}$, the regularization penalty term contributes only $\lambda \cdot RMSD^2_{cis} = 2.03$ to the total objective function $F(\hat{\boldsymbol{p}}) = 494.9$, representing about 0.4% of $F(\hat{\boldsymbol{p}})$. This confirms that the refinement is governed by the experimental data, and the CASPT2 reference acts only as a mild constraint that excludes unphysical regions of the parameter space. The $\lambda$ scan was performed at an earlier stage of the

iterative refinement, before the final solvent cage was adopted; given the breadth of the maximum and the small contribution of the penalty, $\lambda$ was not re-determined for the final refinement. The penalty entered the structural refinement only, and not the error analysis: the profile likelihood analysis described in Supplementary Method 4, from which the uncertainties listed in Extended Data Table 3 were obtained, was carried out on the unregularized $\chi^2$, with the penalty term removed, so the reported uncertainties reflect how sharply the experimental data determine the parameters, and are not altered by the prior derived from the CASPT2 calculations. The resulting *cis*-AB geometry deviates from the CASPT2 reference by an RMSD of 0.52 Å, a modest but statistically significant structural adjustment required by the experimental scattering data.

**12.8 | Validation with an extended seven-motion basis set**

To verify that the four-motion basis set (ROT, INV, NND, CNT) is sufficient to capture the essential structural degrees of freedom, we performed an extended SSPM analysis incorporating three additional motions: C–N bond elongation (CND, parameterized by $\Delta r_{CN}$), pyramidalization at the nitrogen atom (PYR, quantified by the ∠CCCN improper torsion angle), and ∠CCN valence angle variation (CCN, parameterized by Δ∠CCN). Including these seven motions generates $2^7 - 1 = 127$ possible non-empty subsets. To keep the total number of free parameters manageable, we evaluated all combinations with up to four simultaneously active motions. For the *trans*-AB → $I_1$ transition under $C_i$ (inversion) symmetry, the BIC analysis showed that the three additional motions provided no improvement in model selection. The BIC-optimal model remained CNT alone ($\chi^2$ = 123.1, BIC($\chi^2$) = 146.6), identical to the result from the four-motion SSPM. Although the best-performing extended model by $\chi^2$ (NND + CNT + CND + PYR, $k$ = 4, $\chi^2$ = 121.2) achieved a marginally lower $\chi^2$, its BIC (158.8) was substantially worse owing to the complexity penalty for the additional parameters. The best extended model by BIC, CNT + PYR ($\chi^2$ = 122.2, BIC($\chi^2$) = 150.4), also failed to improve upon CNT alone. These results confirm that the extended motions do not contribute meaningfully to the description of this transition, and therefore that no degrees of freedom beyond the initial four-motion basis set are required to describe it. For the $I_1 \rightarrow I_2$ transition, several extended models achieved lower BIC values than ROT + CNT (BIC($\chi^2$) = 201.4). On inspecting the optimized geometries, however, we found that these models lower the BIC only by introducing distortions of the phenyl rings and of the nitrogen geometry that are chemically

unreasonable. Such distortions are neither present in the CASPT2-calculated structures nor consistent with the known characteristics of azobenzene, whose aromatic carbons and nitrogen atoms are $sp^2$-hybridized and therefore resist large angular deformation. Specifically, the models that activate the CCN require ∠CCN to change by at least 12°, and by as much as 27°, even though ∠CCN varies by no more than about 3° across the CASPT2 structures of *trans*-AB, $I_1$, $I_2$, and *cis*-AB. Likewise, the models that activate the PYR require the nitrogen atoms to bend well out of the plane of the attached phenyl ring, whereas in the CASPT2 structures the nitrogen stays close to planar, with an out-of-plane displacement below about 2° at the $I_1$ and $I_2$ stages. In both cases the additional degrees of freedom absorb experimental noise through unphysical deformations rather than capturing genuine structural change. ROT + CNT therefore remains the most parsimonious and physically reasonable model, consistent with the result from the four-motion basis set.

These results are consistent with the expectation that the dominant structural changes during azobenzene photoisomerization involve the central N–N and C–N bond coordinates, rather than peripheral bond-length adjustments or angular distortions of the phenyl rings themselves. The four-motion basis set (ROT, INV, NND, CNT) was therefore adopted for all SSPM analyses.

**Supplementary Method 13 | $\chi^2$-Based BIC for SSPM and $N_{eff}$ Determination**

When fitting experimental data with candidate models of differing complexity, the chi-square ($\chi^2$) statistic alone is generally insufficient for selecting the optimal model. A model with a larger number of free parameters will invariably yield a lower $\chi^2$ than a simpler one, because the additional degrees of freedom allow it to absorb statistical noise in addition to the true signal. The critical question is therefore whether the improvement in $\chi^2$ achieved by the more complex model is statistically significant, that is, whether the gain in goodness-of-fit outweighs the cost of the added parameters, or whether it merely reflects overfitting. The BIC provides a principled framework for this assessment by augmenting the goodness-of-fit term with an explicit penalty proportional to the number of free parameters, thereby favouring the most parsimonious model consistent with the data.

In the SSPM framework, the goodness-of-fit between the experimental and simulated SADSs is quantified by the chi-square statistic, with the experimental uncertainties $\sigma^{\perp}(q_j)$ at each $q$-point obtained from error propagation.

$$\chi^2 = \sum_{j=1}^{n_q} \left[ \left( \mathrm{SADS}_n^{\perp}(q_j) - \mathrm{SADS}_n'^{\perp}(q_j) \right) / \sigma^{\perp}(q_j) \right]^2 \tag{S25}$$

The BIC is defined in general as:

$$\mathrm{BIC} = k \cdot \ln(N) - 2 \cdot \ln(\hat{L}) \tag{S26}$$

Here, $k$ is the number of model parameters, $N$ is the number of data points, and L is the maximized likelihood of the model. For data with Gaussian-distributed residuals and unknown noise variance $\sigma^2$, the maximized log-likelihood takes the form:

$$\ln(\hat{L}) = -(N/2) \cdot [1 + \ln(2\pi) + \ln(\widehat{\sigma^2})] \tag{S27}$$

$\widehat{\sigma}^2$ = RSS / $N$ is the maximum-likelihood estimate of the noise variance, where RSS is the minimum residual sum of squares of the fitted model. Substituting into the general BIC definition:

$$\mathrm{BIC} = k \cdot \ln(N) + N \cdot \ln(\widehat{\sigma^2}) + N(1 + \ln(2\pi)) \tag{S28}$$

The last term, $N \cdot (1 + \ln(2\pi))$, is a constant that does not depend on the model and therefore cancels when comparing ΔBIC between competing models. Dropping this constant yields the working form:

$$\mathrm{BIC} = N \cdot \ln(\widehat{\sigma^2}) + k \cdot \ln(N) \tag{S29}$$

In the present case, the experimental noise level at each $q$-point is independently known through the propagated standard errors ($\sigma(q_j)$), and this information is already incorporated into the $\chi^2$ statistic via the denominator. Because the noise variance need not be estimated from the residuals, the goodness-of-fit term $N \cdot \ln(\hat{\sigma}^2)$ is replaced by $\chi^2$, and the BIC assumes a particularly simple form, which we term the "$\chi^2$-based BIC" to distinguish it from the RMSD-based BIC introduced later in the BMA framework (see Supplementary Method 14). In the general BIC definition above, $k$ denotes the total number of model parameters. In the present SSPM analysis, the total number of model parameters is $k + 4$, where $k$ is the number of active structural (motional) parameters and the four additional parameters are the $\sigma^2$ (the Debye–Waller mean-square displacement), the scaling coefficient, and the two baseline coefficients $a_n$ and $b_n$. Substituting this into the general BIC gives:

$$\mathrm{BIC}_{\chi^2} = \chi^2 + (k + 4) \cdot \ln(N) \tag{S30}$$

The first term ($\chi^2$) quantifies the goodness-of-fit between the model and the experimental data, and the second term ($(k + 4) \cdot \ln(N)$) is the complexity penalty, with $k$ being the number of structural motion parameters in the candidate motion model and $N$ the number of independent data points. Among competing models, the one with the lowest BIC2 is selected as optimal. $\Delta\mathrm{BIC}_{\chi^2} = \mathrm{BIC}_{\chi^2} - \mathrm{BIC}_{\chi^2,\min}$ is computed for each model, where $\mathrm{BIC}_{\chi^2,\min}$ is the lowest BIC2 among all models. Following established conventions[43], models with $\Delta\mathrm{BIC}_{\chi^2} < 2$ relative to the best model have substantial support and cannot be confidently distinguished; those with $2 < \Delta\mathrm{BIC}_{\chi^2} < 6$ have considerably less support; and those with $\Delta\mathrm{BIC}_{\chi^2} > 10$ have essentially no support.

In a typical scattering experiment, however, the measured $q$-points are oversampled relative to the information content of the signal, so that the total number of measured data points cannot be directly used for $N$. Instead, $N$ must be replaced by the effective number of independent data points, $N_{\mathrm{eff}}$, giving:

$$\mathrm{BIC}_{\chi^2} = \chi^2 + (k + 4) \cdot \ln(N_{\mathrm{eff}}) \quad \text{(S31)}$$

For most curve-fitting problems, $N_{\mathrm{eff}}$ is simply the total number of measured data points, provided each point contributes independent information. In solution scattering experiments, however, this condition is not satisfied: adjacent $q$-points are strongly correlated because the underlying scattering signal varies on a spatial-frequency scale much coarser than the detector pixel spacing in $q$-space. Determining $N_{\mathrm{eff}}$ for such oversampled data is non-trivial and requires an explicit estimation procedure.

We adopted the following approach: if a polynomial of order K is sufficient to reproduce the experimental curves within statistical significance, then K represents the number of independently resolvable features in the data and serves as an estimate of $N_{\mathrm{eff}}$. We note that this procedure yields a lower bound on $N_{\mathrm{eff}}$, because it quantifies the information content actually present in the current set of experimental curves, which need not exhaust the full information capacity of the scattering measurement. The physical signals may occupy only a subset of the available independent channels, just as an object moving along a straight line in three-dimensional space utilizes only one of the three available spatial degrees of freedom. Nonetheless, this data-driven approach provides a robust and model-independent estimate of $N_{\mathrm{eff}}$ without requiring prior knowledge of quantities such as the maximum interatomic distance ($D_{\max}$), which is not uniquely defined for solution scattering data that include both solute and

solvent contributions.

To determine this optimal polynomial order in a statistically objective manner, we employed the BIC. Increasing the polynomial order monotonically reduces the fitting residual; however, beyond a certain order the improvement reflects fitting of noise rather than genuine signal. For each trial order K, a BIC score was computed to assess whether the marginal reduction in residual justified the additional polynomial coefficients. The polynomial fitting was applied to a set of ten curves that collectively span the full information content of the scattering data: (i) three PEPC-treated SADSs, which represent the solute and cage contributions independent of the solvent response, and (ii) seven solvent-response basis vectors (LSVs from the SVD of the neat methanol data) used in the PEPC procedure, which represent the solvent contribution. Together, these ten curves encompass the solute, cage, and solvent terms that constitute the measured scattering signal. Each of these ten curves was independently fitted with a Chebyshev polynomial of order $K$ over the $q$-range of 1–7 Å$^{-1}$, and the residuals were pooled across all ten curves to evaluate the BIC:

$$\mathrm{BIC}(K) = N_{\mathrm{total}} \cdot \ln\left(\widehat{\sigma^2}\right) + k_{\mathrm{total}} \cdot \ln(N_{\mathrm{total}}) \tag{S32}$$

$N_{\mathrm{total}} = n_q \times 10$ is the total number of data points across all ten curves, $k_{\mathrm{total}} = (K + 1) \times 10$ is the total number of polynomial coefficients, and $\hat{\sigma}^2 = \mathrm{RSS}_{\mathrm{total}} / N_{\mathrm{total}}$ is the pooled variance estimated from the total residual sum of squares. Scanning $K$ from 1 to 250, the BIC($K$) curve exhibited a clear global minimum at $K$ = 110, indicating that a 110th-order Chebyshev polynomial captures all statistically resolvable features in the data. We therefore adopted $N_{\mathrm{eff}}$ = 110 as the effective number of independent data points for all subsequent $\chi^2$-based BIC calculations over this $q$-range. Notably, this value lies in the upper part of the $N_s \approx$ 60–120 range estimated independently in the Methods section “Structure analysis”, corresponding to an effective maximum distance of $D_{\mathrm{max}} \approx 58$ Å that reflects the solvent response (bulk-density and thermal-fluctuation contributions) extending well beyond the immediate solvation shell. This agreement between the two independent estimates validates the robustness of our BIC-based estimation of $N_{\mathrm{eff}}$. With $N_{\mathrm{eff}}$ = 110, the BIC complexity penalty amounts to ln(110) ≈ 4.70 per additional structural parameter ($k$ + 4 in total per model). We note that $N_{\mathrm{eff}}$ = 110 is used only for the BIC complexity penalty $(k + 4) \cdot \ln(N_{\mathrm{eff}})$, which governs model selection. The reduced chi-square (Eq. S13) is a separate, conventional goodness-of-fit measure and is therefore normalized by $(n_q - d_f)$ with $n_q$ = 511 (the actual number of $q$-points), not by $N_{\mathrm{eff}}$. This choice of denominator affects only the reported numerical value of the reduced chi-square.

Because dividing by a constant scales the score of every candidate structure by the same factor, it leaves their relative ranking unchanged, so the same structure minimizes the score (the best-fit structure is identical) whether $n_q$ or $N_{eff}$ is used.

The ten curves employed in the determination of $N_{eff}$ differ in amplitude: the seven solvent-response basis vectors are normalized to unit length (because they are obtained from the SVD analysis), whereas the three SADSs retain the absolute scale of the experimental data. Accordingly, the determination of $N_{eff}$ is governed mainly by the solvent-response basis vectors. This dominance is physically reasonable for the purpose of counting independent data points. The number of independent channels (information content) in a scattering curve is set by the longest real-space distances that contribute to the signal: a distance $r$ appears in the scattering curve as an oscillation of period ~$2\pi / r$ in $q$-space, so the longest distances produce the finest resolvable features over the measured $q$-range, giving $N_s \approx (q_{max} - q_{min}) \cdot D_{max} / \pi$ (see Methods). Among the contributions to the measured signal, it is the solvent response that extends farthest in real space. As noted above, the resulting $N_{eff} = 110$ corresponds to $D_{max} \approx 58$ Å, in agreement with the independent estimate that includes the solvent contribution ($N_s \approx 60$–$120$).

To examine the influence of this amplitude imbalance, we repeated the determination of $N_{eff}$, varying both the set of curves entering the fit and their relative weights. The seven solvent-response basis vectors alone return the same value, $N_{eff} = 110$. The three SADSs alone return $N_{eff} = 183$. We also varied only the weights, keeping all ten curves. In one case, each curve was rescaled so that its root mean square amplitude equals one. This rescaling returns $N_{eff} = 91$. In the other case, the three SADSs were rescaled together, so that their total sum of squares equals that of the seven solvent curves. This rescaling also returns $N_{eff} = 91$.

We then examined how the model selection of SSPM depends on the value of $N_{eff}$. The results show that the selected models are unchanged over the entire range of $N_{eff}$ examined (Supplementary Table 6). Since $BIC_{\chi 2} = \chi^2 + (k + 4) \cdot \ln N_{eff}$ (Eq. S31) and the four non-structural parameters are common to all models, the difference in BIC between two models is $\Delta BIC = \Delta\chi^2 + \Delta k \cdot \ln N_{eff}$, where $\Delta k$ is the difference in the number of structural parameters. We evaluated this difference for both *trans*-AB → $I_1$ and $I_1$ → $I_2$. The values of $N_{eff}$ tested included every estimate described above (91, 110, 183), geometric estimates based on the Shannon number (23, 29, 57, and 115), and the number of sampled points itself (511), which is the largest value $N_{eff}$ can take. For each transition, all 15 candidate motion combinations were compared. At every value of $N_{eff}$ tested, the reported model (CNT for *trans*-AB → $I_1$ and

ROT + CNT for $I_1 \rightarrow I_2$) has the lowest BIC among the 15. Supplementary Table 6 lists ΔBIC between the reported model and the model with the second-lowest BIC, which at every tested $N_{eff}$ is NND + CNT for *trans*-AB → $I_1$ and ROT + NND + CNT for $I_1 \rightarrow I_2$.

The ΔBIC increases with $N_{eff}$, because a larger $N_{eff}$ imposes a larger penalty per parameter and the reported models are the simplest in both transitions. The choice of $N_{eff}$ would alter the selection only below $N_{eff} = 2.512$ for *trans*-AB → $I_1$ and $N_{eff} = 2.31$ for $I_1 \rightarrow I_2$. These values correspond to a maximum real-space distance of about 1.3 Å, shorter than a carbon-carbon bond, and are therefore not values $N_{eff}$ can take for this measurement.

## Supplementary Method 14 | Basis-Motion Analysis: Algorithm, Alignment, and RMSD-Based BIC

While the SSPM-based structural fitting (see Supplementary Methods 12 and 13) identifies the motions that best reproduce the experimental SADSs, an independent structure-based analysis is needed to characterize the motions connecting pairs of three-dimensional molecular structures, including those obtained from quantum chemical calculations for which experimental scattering data are not available. The Basis-Motion Analysis (BMA) addresses this by evaluating how well a given set of basis motions, when applied to an initial structure A, can reproduce a target structure B.

The structural changes of azobenzene are represented using four elementary basis motions, each controlled by one or two structural parameters: (i) Rotation (ROT): Torsion of one phenyl ring about the N–N bond axis by an angle $\varphi_{ROT}$ (degrees), which directly modifies the CNNC dihedral angle ($k$ = 1). The resulting dihedral is $\angle CNNC = \angle CNNC^{init} + \varphi_{ROT}$. (ii) Inversion (INV): Two independent CNN bending angles, $\angle CNN_1$ and $\angle CNN_2$, controlled by parameters $\theta_{INV1}$ and $\theta_{INV2}$ (degrees). Each phenyl ring rotates in the plane defined by the N–N axis and the C–N bond, about an axis perpendicular to this plane through the respective nitrogen atom ($k$ = 2). The resulting angles are $\angle CNN_i = \angle CNN^{init}_i + \theta_{INVi}$, where $\angle CNN^{init}_i$ is the angle before applying this motion. (iii) N–N bond-length change (NND): Elongation or contraction of the N=N bond by a displacement $d_{NND}$ (Å), applied by translating one $C_6H_5N$ subunit along the inter-nitrogen axis ($k$ = 1). The resulting N–N bond length is $r_{NN} = r_{NN}^{init} + d_{NND}$, where $r_{NN}^{init}$ is the N–N bond length of the structure before applying this NND motion. (iv) C–N bond torsion (CNT): Torsion about its respective C–N bond axis by angles $\varphi_{CNT1}$ and

$\varphi_{\mathrm{CNT2}}$ (degrees). The resulting ∠CCNN angles are $\angle\mathrm{CCNN}_i = \angle\mathrm{CCNN}_i^{\mathrm{init}} + \varphi_{\mathrm{CNT}i}$, where $\angle\mathrm{CCNN}_i^{\mathrm{init}}$ is the angle before applying this motion. Unlike ROT, which rotates a ring about the N–N axis and modifies ∠CNNC, CNT rotates each ring or the azo group about the C–N axis and modifies the ∠CCNN angles while leaving the ∠CNNC dihedral and N–N framework unaffected ($k = 2$). Each of the four motions acts exclusively on a distinct structural degree of freedom (∠CNNC, ∠CNN, $r_{\mathrm{NN}}$, and ∠CCNN, respectively) and preserves the geometric reference frames of the others, rendering them mutually commutative: the individual contribution of each motion retrieved by the BMA is independent of the order in which the motions are applied. In the implementation, the motions are applied sequentially in the fixed canonical order: INV → NND → ROT → CNT to ensure algorithmic reproducibility.

For a given motion combination (model) M with $k$ free parameters, the objective function is the RMSD between the transformed and target structures:

$$\mathrm{RMSD}(\boldsymbol{p}) = \sqrt{\frac{1}{N_{\mathrm{atoms}}} \sum_{i=1}^{N_{\mathrm{atoms}}} \left\| \boldsymbol{r}_i^{\mathrm{transformed}} - \boldsymbol{r}_i^{\mathrm{target}} \right\|^2} \tag{S33}$$

Here, $\boldsymbol{p} = (p_1, \ldots, p_k)$ is the parameter vector, $\boldsymbol{r}_i^{\mathrm{transformed}}$ and $\boldsymbol{r}_i^{\mathrm{target}}$ are the position vectors of atom $i$ in the transformed and target structures, and ‖·‖ denotes the Euclidean norm, so that $\| \boldsymbol{r}_i^{\mathrm{transformed}} - \boldsymbol{r}_i^{\mathrm{target}} \|$ is the distance between these two positions of atom $i$. The $k$ components of $\boldsymbol{p}$ are the amplitudes of the basis motions active in the candidate model, each equal to the change in the structural coordinate that defines the motion (Δ∠CNNC for ROT, Δ∠CCNN for CNT, $\Delta r_{\mathrm{NN}}$ for NND, and Δ∠CNN for INV). The summation runs over all $N_{\mathrm{atoms}} = 24$ atoms of azobenzene. The optimization employed a three-phase global strategy. Phase 1a (coarse grid scan): The parameter space was uniformly sampled on a grid with 20° spacing for angular dimensions, and the RMSD was evaluated at every grid point. Phase 1b (Nelder–Mead refinement): The top 200 grid points were each refined by the Nelder–Mead simplex algorithm (MATLAB fminsearch). Phase 1c (particle swarm): Three independent runs of particle-swarm optimization (100 particles each) were performed to ensure stochastic coverage of basins not captured by the grid. Phase 2 (Levenberg–Marquardt refinement): The top 50 candidates from Phases 1a–1c were each further refined by the Levenberg–Marquardt algorithm (MATLAB lsqnonlin) on the vectorized Cartesian residuals, with all 40 alignment variants (defined below) evaluated independently per candidate to ensure that each solution converges under its optimal alignment. Phase 3 (parsimony selection): All Phase 2 solutions were grouped by RMSD within

a tolerance of $10^{-4}$ Å. Among solutions achieving the same lowest RMSD, the most physically compact one was selected (described in detail below).

The 40 alignment variants evaluated for each candidate in Phase 2 arise from the symmetry of azobenzene, as follows. When computing the RMSD, the two structures were optimally superposed using the Kabsch algorithm, which determines the rotation that minimizes the sum of squared deviations between corresponding atoms. Because the RMSD depends on how the atoms of the two structures are paired, and the atoms of azobenzene are related by symmetry, several different pairings are equally valid and must all be tested to find the true minimum. Two sources of equivalent pairings arise. First, the two phenyl rings are equivalent under the molecular pseudo-$C_2$ axis and may therefore be interchanged, and each ring in addition has local $C_2$ symmetry about its C–N bond axis, so the two ortho (and the two meta) positions within a ring may be exchanged. Together these give 8 atomic permutations: 2 for interchanging the two rings, and a further factor of 2 for the within-ring reversal of each of the two rings (2 × 2 × 2 = 8). Second, because the Kabsch algorithm optimizes only proper rotations, a mirror-related pairing cannot be reached by rotation alone. To test these improper alignments as well, 5 sign operations were applied to the coordinate axes before superposition: the identity, the three reflections across the coordinate planes, and the inversion. Combining the 8 atomic permutations with the 5 sign operations yields 40 distinct alignments, and the alignment giving the minimum RMSD was retained.

To identify the simplest motion combination that adequately describes the structural transition, we employed the BIC. The residual at each Cartesian coordinate (x, y, or z of each atom) is defined as the difference between the transformed candidate structure and the target structure after optimal Kabsch superposition. The RMSD-based BIC was derived assuming that these residuals are independently and identically distributed, each following a normal (Gaussian) distribution with mean zero and unknown variance $\sigma^2$: $\varepsilon_j \sim N(0, \sigma^2)$, $j = 1, \ldots, n$ where $\varepsilon_j$ is the residual of the $j$-th coordinate, and ~ denotes "is distributed as"; $N(0, \sigma^2)$ denotes a normal (Gaussian) distribution with mean zero and variance $\sigma^2$. Here $n = 3 \times N_{\text{atoms}} = 72$ is the total number of fitted Cartesian coordinates. The BIC is constructed from the likelihood of the data under this Gaussian model, and this likelihood depends on the residuals only through their sum of squares, the residual sum of squares $\text{RSS}(\boldsymbol{p}) = \sum_{j=1}^{n} \varepsilon_j^2$. This RSS is related to the fitting objective RMSD($\boldsymbol{p}$) of equation (S33) by $\text{RSS}(\boldsymbol{p}) = N_{\text{atoms}} \times \text{RMSD}(\boldsymbol{p})^2$. At the best-fit parameters, $\hat{p}$ RSS($\boldsymbol{p}$) and RMSD($\boldsymbol{p}$) are RSS($\hat{p}$) and RMSD($\hat{p}$), respectively. Unlike the $\chi^2$-

based BIC, where the noise variance is known from experimental error propagation, here $\sigma^2$ must be estimated from the data. Its maximum-likelihood estimate is $\hat{\sigma}^2 = \mathrm{RSS}(\hat{p}) / n = \mathrm{RMSD}(\hat{p})^2 / 3$ (using $n = 3 \times N_{\mathrm{atoms}}$). The general BIC (equation (S29), Supplementary Method 13) is BIC $= N\ln(\hat{\sigma}^2) + k \cdot \ln(N)$, where $N$ is the number of fitted data points and $k$ is the number of free parameters. For the RMSD-based fit the data points are the n = 72 Cartesian residuals, so $N = n$. Substituting $\hat{\sigma}^2 = \mathrm{RSS}(\hat{p}) / n = \mathrm{RMSD}(\hat{p})^2 / 3$ into equation (S29) gives:

$$\mathrm{BIC}_{\mathrm{RMSD}} = n \cdot \ln\left(\mathrm{RMSD}(\widehat{p})^2/3\right) + k \cdot \ln(n) \quad \text{(S34)}$$

The subscript "RMSD" distinguishes this criterion from the $\chi^2$-based BIC used in the SSPM analysis. The model with the lowest $\mathrm{BIC}_{\mathrm{RMSD}}$ is preferred. Here $k$ is the number of structural motion parameters of the candidate model. Unlike the $\chi^2$-based BIC (equation S30), no additional parameters for the Debye–Waller mean-square displacement or the scaling coefficient appear, because $\sigma^2$ is estimated from the residuals (profiled out of the likelihood) and no scaling coefficient enters the RMSD objective. $\Delta\mathrm{BIC}_{\mathrm{RMSD}} = \mathrm{BIC}_{\mathrm{RMSD}} - \mathrm{BIC}_{\mathrm{RMSD,min}}$ is computed for each model, where $\mathrm{BIC}_{\mathrm{RMSD,min}}$ is the lowest $\mathrm{BIC}_{\mathrm{RMSD}}$ among all tested combinations. Following established conventions, models with $\Delta\mathrm{BIC}_{\mathrm{RMSD}} < 2$ relative to the best model have substantial support and cannot be confidently distinguished; those with $2 < \Delta\mathrm{BIC}_{\mathrm{RMSD}} < 6$ have considerably less support; and those with $\Delta\mathrm{BIC}_{\mathrm{RMSD}} > 10$ have essentially no support. For azobenzene with $n = 72$, the penalty per additional parameter is $\ln(72) \approx 4.28$. As a complementary metric, the reduced chi-square $\chi_\nu^2 = \chi^2 / (n - k)$ was also computed, where $\chi^2 = \mathrm{RSS} / \sigma_{\mathrm{ref}}^2$ and $\sigma^2_{\mathrm{ref}} = \mathrm{RSS}_{\mathrm{full}} / n$ is the variance estimated from the residual sum of squares of the full four-motion model (ROT + INV + NND + CNT), which achieves the minimum RMSD among all tested combinations. The rationale is that the full model, which includes all available degrees of freedom, is assumed to capture the complete structural transformation; its residual therefore provides the best available estimate of the intrinsic precision limit of the basis-motion fitting.

We note that, because the basis motions involve rotations (which are periodic) and can partially compensate one another, multiple parameter sets can produce the same final structure and thus yield essentially identical RMSD values. For example, a target structure whose ∠CNNC differs from *trans*-AB by 180° can be equally well described by a pure ROT (torsion about N–N) or by a pure INV (bending of ∠CNN angles), leading to distinct parameter sets with comparable RMSD. To resolve this degeneracy, the total effort, the summed per-step RMSD over all active motions, was used:

$$\text{Effort} = \sum_j \text{RMSD}_{\text{step}}(j) \tag{S35}$$

$\text{RMSD}_{\text{step}}(j)$ is the root-mean-square atomic displacement produced by the $j$-th motion alone, evaluated by applying each motion sequentially at its optimized parameter value and recording the per-step displacement. Among all solutions within $10^{-4}$ Å of the global minimum RMSD, the one with the smallest effort was selected. This criterion favours the solution in which each motion contributes the least atomic displacement, avoiding parameter sets where large, mutually compensating motions artificially inflate the individual contributions.

When the true generating motions are contained within the model, the optimizer can achieve RMSD values at or below the numerical precision of the input coordinates. In such cases, the BIC log-likelihood term $n \cdot \ln\left(\text{RMSD}(\hat{p})^2/3\right)$ in equation (S34) diverges to $-\infty$, causing the complexity penalty $k \cdot \ln(n)$ to become negligible regardless of the number of parameters. This allows models augmented with inactive parameters (optimized values indistinguishable from zero) to achieve lower BIC than the correct, more parsimonious model. To prevent this artefact, an RMSD floor of $\text{RMSD}_{\text{floor}} = 10^{-4}$ Å was imposed, so that the effective RMSD used in equation (S34) is $\text{RMSD}_{\text{eff}} = \max(\text{RMSD}, \text{RMSD}_{\text{floor}})$. Because $\sigma^2 = \text{RMSD}^2 / 3$, this corresponds to a variance floor of $\sigma^2_{\text{floor}} = (10^{-4}\ \text{Å})^2 / 3 \approx 3.3 \times 10^{-9}\ \text{Å}^2$. This threshold reflects three independent precision limits of the input coordinates. First, the XYZ file format records atomic positions with finite decimal precision (typically 4–6 digits), setting an I/O-level floor of ~$10^{-4}$–$10^{-6}$ Å per coordinate. Second, the intrinsic accuracy of the CASPT2-calculated geometries used in this work is ~$10^{-3}$ Å, as established by benchmark comparisons between CASPT2 and CC3 excited-state bond lengths[44] and by earlier assessments of CASPT2 versus CASSCF structural differences of 0.01–0.02 Å[45]. Third, structural parameters determined by TRXL experiments are typically reported with uncertainties on the order of $10^{-2}$–$10^{-3}$ Å[5,13,38,46], confirming that RMSD differences below $10^{-4}$ Å are well below the experimentally resolvable limit. The chosen floor of $10^{-4}$ Å is conservative (one or two orders of magnitude below the method accuracy) and ensures that RMSD differences below this threshold, which carry no structural significance, do not influence model ranking. With the floor in place, models whose RMSD falls below $10^{-4}$ Å are assigned identical log-likelihood, and selection among them is governed solely by the parsimony penalty $k \cdot \ln(n)$. The BMA results are discussed in Supplementary Note 4.

**Supplementary Method 15 | Motional Contribution Analysis (MoCA)**

The $BIC_{RMSD}$ analysis described above identifies which combination of basis motions best describes a given structural transition. When the selected combination contains more than one basis motion, for example, ROT + INV rather than ROT alone, a natural question arises: what is the relative contribution of each individual basis motion to the overall structural change? To address this, we developed the Motional Contribution Analysis (MoCA), which evaluates the functional necessity of each basis motion within the assigned combination using a leave-one-out (LOO) approach. Specifically, MoCA measures the increase in the optimized RMSD when a specific basis motion is excluded from the combination.

Let N = $\{M_1, M_2, \ldots, M_n\}$ denote the set of motions assigned to a given structural transition. For any subset $S \subseteq N$, we define RMSD*(S) as the minimum RMSD achievable when only the motions in S are used to reproduce the target structure, obtained by the same optimization protocol described above. Two special cases are RMSD*(∅) ≡ $R_0$, the RMSD between the initial and target structures with no motion applied, and RMSD*(N), the RMSD corresponding to the combination of all the motions. A basic property of this definition is that adding a motion to a subset can never worsen the fit. For any subset $S \subseteq N$ and any motion $M_i$ not contained in S ($M_i \notin S$), consider the subset $S \cup \{M_i\}$ obtained by adding $M_i$ to S. Setting the amplitude (weight) of $M_i$ to zero reproduces exactly the fit obtained with S alone, so optimizing over $S \cup \{M_i\}$ cannot yield a larger RMSD than optimizing over S, RMSD*($S \cup \{M_i\}$) ≤ RMSD*(S). Equivalently, removing a motion from a subset can never improve the fit. The LOO contribution of motion $M_i$ quantifies how much the fit quality deteriorates when $M_i$ is removed from the best solution, while all other motions are re-optimized:

$$\Delta_i = \mathrm{RMSD}^*(\mathrm{N} \setminus \{\mathrm{M}_i\}) - \mathrm{RMSD}^*(\mathrm{N}) \quad \text{(S36)}$$

Here, $N \setminus \{M_i\}$ denotes the set N with motion $M_i$ removed (which is itself one of the subsets S defined above). Because $N \setminus \{M_i\}$ is a subset of N, the property above guarantees $\Delta_i \geq 0$: a large $\Delta_i$ means that $M_i$ is essential for reproducing the target structure, while $\Delta_i \approx 0$ means that the remaining motions can compensate for its absence. The fractional LOO contribution is:

$$f_i = \left( \Delta_i / \sum_j \Delta_j \right) \times 100\% \quad \text{(S37)}$$

The sum of LOO contributions $\sum_j \Delta_j$ does not, in general, equal the total RMSD

reduction $R_0 - \mathrm{RMSD}^*(N)$. This is because each basis motion displaces atoms along its own structural coordinate (e.g., ∠CNN bending, N–N stretching), which in general does not coincide with the shortest Cartesian path between the initial and target structures. As a result, the individual RMSD reductions are not additive. Nevertheless, the fractional LOO contribution, $f_i$, remains valid for comparing the relative contribution of each basis motion to the structural transition.

A schematic for structural analysis, including SSPM, BIC, BMA, and MoCA is shown in Supplementary Fig. 3.

## Supplementary Notes

### Supplementary Note 1 | Explanation of three mechanisms for *trans*-to-*cis* isomerization proposed in the literature

Extensive studies using time-resolved spectroscopies[2-4,47-53] and theoretical calculations[54-76] have proposed three distinct mechanisms for the *trans*-to-*cis* isomerization of azobenzene: rotation[3,47,54,56-63,65-68], inversion[49,50,52,55], and hula-twist[4,64], along with their combinations[53,69-76] (Fig. 1a). In the rotation mechanism, also known as the one-bond flip or twisting mechanism, isomerization proceeds through the rotation of one phenyl ring around the N–N bond axis. In the inversion mechanism, also referred to as in-plane rotation, the phenyl ring adjacent to each nitrogen atom rotates within the molecular plane to form the *cis* isomer. The hula-twist mechanism involves the displacement of nitrogen atoms, which induces a twist in the attached phenyl ring[4,64].

### Supplementary Note 2 | Analysis of the anisotropic scattering signal

To elucidate the time-dependent orientational dynamics, we applied SVD analysis to the solvent-independent anisotropic difference scattering curve, $\Delta S^{\perp}_{\mathrm{aniso}}(q, t)$. SVD decomposes the data matrix into time-independent LSVs and time-dependent RSVs. Based on the singular values and autocorrelation analysis, the anisotropy data were found to contain only a single significant component (Extended Data Fig. 3). Consequently, to determine the structural origin of this single component, $\Delta S^{\perp}_{\mathrm{aniso}}(q, t)$ was fitted with the simulated anisotropic scattering curve of each species involved in the reaction (*trans*-AB, $I_1$, $I_2$, and *cis*-AB), calculated using the standard expression for photoselectively excited ensembles (Supplementary Method 7). The *trans*-AB structure reproduces the data well, and the resulting $S^{\perp}_{\mathrm{aniso},trans}{}'(q)$ overlaps LSV1 (blue and black curves in Extended Data Fig. 3e), indicating that the signal is dominated by the photoselective anisotropy of the ground-state depletion hole. However, we note that the simulated anisotropic signal calculated for the $I_1$ structure also exhibits a highly similar $q$-dependent shape, owing to the structural resemblance between $I_1$ and *trans*-AB. This implies that the single significant component identified by SVD likely represents a superposition of contributions from both the depletion of *trans*-AB and the $I_1$ intermediate, which are spectrally indistinguishable within the current signal-to-noise ratio (SNR).

Indeed, the subsequent kinetic analysis supports the contribution of $I_1$. To extract

kinetic information, the relative concentration profile of $S^{\perp}_{\mathrm{aniso},\mathit{trans}}{}'(q)$, $c(t)$ (Extended Data Fig. 3f), was fitted with a sum of exponential functions convolved with the IRF, using the same model that we had applied to the RSVs of the isotropic difference scattering data (equation (S3)); the time constants were obtained by minimizing the chi-square between the experimental and calculated concentration profile. Just as in the fit to the RSVs of the isotropic difference scattering data (Supplementary Method 1), the model included a fast exponential with its time constant fixed at 20 fs, which accounts for the instrument-limited onset of the signal and is not interpreted as a kinetic time constant of the reaction. In that fit to the isotropic RSVs, the model also contained, besides the three free time constants and the 20-fs onset, one further exponential whose time constant was fixed at a very large value (1 μs); over the measured time window it stays essentially constant, representing the long-lived product that has not decayed by the end of the window. Like the 20-fs term, this long-lived term is not interpreted as a kinetic time constant of the reaction. For $c(t)$, this long-lived term was likewise retained; it accounts for a small residual offset that even dips slightly below zero at late delays. Because a negative concentration is unphysical, this offset is regarded as an artefact arising from the weakness of the anisotropic signal rather than a genuine long-lived component. The fit required only two free time constants, and it yields an instrument-limited (IRF-limited) instantaneous rise (from the fixed 20-fs term) followed by these two time constants, 1.2 ± 0.2 ps and 24.6 ± 1.3 ps (Extended Data Fig. 3f).

The instantaneous rise is prominent in the anisotropic signal but weak in the isotropic signal. This contrast can be understood by considering that the anisotropic signal is sensitive to the orientational distribution of the molecular ensemble, whereas the isotropic signal is predominantly sensitive to changes in intramolecular interatomic distances. The instantaneous component is consistent with an impulsive rotational response of the solute molecules induced by the linearly polarized pump pulse, in which the interaction between the pump electric field and the anisotropic molecular polarizability exerts a torque on the solute, analogous to the optical Kerr effect but acting on the solute rather than the bulk solvent. This process reorients the solute molecules on a timescale faster than the IRF, producing a strong anisotropic signal through the change in orientational distribution. Because this reorientation does not alter the intramolecular structure of the solute, its contribution to the isotropic signal is weak, limited to minor perturbations of the solute–solvent intermolecular distances. We note that the conventional optical Kerr effect of the bulk solvent, which would also produce an impulsive anisotropic signal, does not contribute here: this bulk solvent response was independently

measured from neat solvent data and removed through the PEPC procedure. The remaining alignment-related component therefore originates exclusively from the orientational dynamics of the solute molecules.

In addition to the impulsive rotational response discussed above, photoselective excitation creates a further source of anisotropy through orientational selection: molecules whose transition dipole moments are aligned with the pump polarization are preferentially excited, producing an anisotropic depletion (hole) in the *trans*-AB ground-state population. Although the impulsive alignment and the photoselective depletion arise from distinct physical mechanisms, both manifest as changes in the orientational distribution of the *trans*-AB ground-state ensemble and therefore produce the same $q$-dependent scattering profile. The photoselection simultaneously generates a correspondingly anisotropic excited-state population that evolves towards the $I_1$ intermediate. One would therefore expect this excited-state contribution to appear as an additional, independent anisotropy component in the SVD analysis. However, the SVD identifies only a single significant component (Extended Data Fig. 3), indicating that the excited-state contribution is not independently resolved. This can be understood as a direct consequence of the structural similarity between $I_1$ and *trans*-AB. As determined by the BIC analysis of the SSPM result (Supplementary Table 2), the *trans*-AB → $I_1$ transition is described entirely by the CNT motion, which modifies only the ∠CCNN dihedral angles while the azo backbone, including the ∠CNNC dihedral, the ∠CNN bond angles, and the N–N bond length, remains essentially unchanged. Because $I_1$ differs from *trans*-AB only in the ∠CCNN angles, the hole (removal) and $I_1$ (creation) contributions are highly similar in their $q$-dependent profile but opposite in sign, and partially cancel one another. The two contributions therefore project almost entirely onto a single singular vector, explaining why SVD identifies only one component despite the presence of two physically distinct species.

The subtle rise observed with a 1.2 ps time constant can thus be attributed to the $I_1$ → $I_2$ transition, which removes the cancelling $I_1$ contribution and allows the net anisotropy to increase slightly; this 1.2 ± 0.2 ps anisotropic constant is of the same order as the $\tau_2$ (2.7 ± 1.0 ps) obtained from the kinetic analysis of the isotropic signal. Finally, the signal fully vanishes with a time constant of 24.6 ps, which is assigned to the rotational dephasing of the ground-state *trans*-AB ensemble. As the orientational distribution of the unexcited *trans*-AB molecules randomizes through rotational diffusion, the anisotropic depletion (hole) created by the pump pulse is filled in, and the net anisotropy signal decays to zero.

Notably, no additional anisotropy component with a distinct $q$-space profile is

observed at any time delay, despite the fact that $I_2$ and *cis*-AB are structurally distinct from *trans*-AB and would be expected to produce anisotropy signals with a different $q$-dependent profile. Given that the structures of these species (as determined from our structural refinement) lack spherical or high-order rotational symmetry (e.g., $O_h$ or $T_d$ point groups), the lack of detectable anisotropy from $I_2$ and *cis*-AB cannot be attributed to intrinsic isotropy of their molecular structures. Instead, we attribute the lack of detectable anisotropy from these species to the large-amplitude ROT motion that dominates the $I_1 \rightarrow I_2$ transition (67% LOO contribution): the substantial torsional reorientation of the molecular framework during this step effectively randomizes the orientational distribution of the $I_2$ population at the moment of its formation, so that $I_2$ is produced with little or no residual alignment relative to the pump polarization. The same applies to *cis*-AB, which is formed subsequently from the already randomized $I_2$ ensemble.

In summary, the observed anisotropy originates solely from the ground-state depletion hole of *trans*-AB and the transient contribution of the *trans*-AB-like $I_1$ intermediate, while neither $I_2$ nor *cis*-AB exhibits any detectable anisotropy.

**Supplementary Note 3 | Discussion on the SSPM results**

Supplementary Table 2 summarizes the structural motions and their combinations evaluated in the SSPM analysis, listing the corresponding fitting parameters, motional degrees of freedom, $\chi^2$ values, and $\Delta\mathrm{BIC}(\chi^2)$ values. Extended Data Fig. 6a plots the SSPM and BIC results for $I_1$. The BIC-best model is CNT alone ($k + 4 = 5$, $\chi^2 = 123.1$, $\chi_\nu^2 = 0.243$, $\mathrm{BIC}(\chi^2) = 146.6$; Supplementary Table 2), where $k = 1$ is the single active motional parameter (one CCNN angle, the other determined by $C_i$ symmetry) and the four additional parameters are $\sigma^2$, the scaling coefficient, and the two baseline coefficients $a_n$ and $b_n$. Including all four motions ($k + 4 = 8$) reduces the chi-square only marginally ($\chi^2 = 122.1$, $\chi_\nu^2 = 0.243$, $\mathrm{BIC}(\chi^2) = 159.7$, $\Delta\mathrm{BIC}(\chi^2) = 13.2$), demonstrating that CNT alone accounts for essentially all of the structural information contained in $\mathrm{SADS}_1$. The substantially higher BIC for the full model arises because the additional parameters do not provide meaningful improvement to the fit while incurring a larger complexity penalty. The next-ranked model, NND + CNT ($k + 4 = 6$, $\mathrm{BIC}(\chi^2) = 150.3$, $\Delta\mathrm{BIC}(\chi^2) = 3.8$), does not achieve a statistically significant improvement over CNT alone, further confirming that CNT is sufficient.

The primary structural parameters (∠CNNC, ∠CCNN, ∠CNN, $r_{\mathrm{NN}}$, $d_{\mathrm{TL}}$, and $R_g$) of the

refined $I_1$ structure are listed in Extended Data Table 3. Because the BIC-best model activates only CNT, the ∠CNNC dihedral and ∠CNN inversion angles remain at their *trans*-AB values (180.0° and 113.4°, respectively), while the ∠CCNN angles undergo a large change (0°, 0° → 87.0°, −87.0°). Physically, $I_1$ can be generated from *trans*-AB in two ways: (i) a near-90° twist of the phenyl rings about their C–N bond axes without any perturbation of the central CNNC framework (Supplementary Fig. 8; Supplementary Videos 3 and 4), or (ii) bicycle-pedal-like concerted displacement of the azo group with the phenyl rings remaining nearly stationary (Fig. 4b; Supplementary Videos 1 and 2). Both scenarios produce identical internal coordinate changes and are compared in Supplementary Videos 5 and 6; the distinction between them is discussed in the main text and Supplementary Note 11.

*trans*-AB belongs to the $C_{2h}$ point group, whose non-trivial symmetry elements are a $C_2$ rotation axis, a mirror plane $\sigma_h$ coinciding with the molecular plane, and an inversion centre $i$. When the phenyl rings twist out of the molecular plane (∠CCNN ≠ 0°), $\sigma_h$ is necessarily broken; the remaining two elements, $C_2$ and $i$, impose distinct constraints on the ∠CCNN angles: $C_2$ requires $\angle CCNN_1 = \angle CCNN_2$, while $i$ requires $\angle CCNN_1 = -\angle CCNN_2$. The SSPM analysis for the *trans*-AB → $I_1$ transition was performed under $C_i$ symmetry ($\angle CCNN_1 = -\angle CCNN_2$). We also tested alternative symmetry constraints: $C_2$ symmetry ($\angle CCNN_1 = \angle CCNN_2$) and a fixed constraint ($\angle CCNN_1 = \angle CCNN_2 = 90°$). All three variants produced comparable BIC values with ΔBIC < 2, indicating that the data cannot distinguish among these symmetry assumptions. The $C_i$-constrained fit yields ∠CCNN = ±87.0° with a profile likelihood uncertainty of ±14.0° (Extended Data Table 3), a range that encompasses 90°. A ∠CCNN angle of exactly 90° would place the phenyl rings perpendicular to the C–N–N–C plane, a configuration that simultaneously satisfies both the $C_i$ constraint ($\angle CCNN_1 = -\angle CCNN_2$) and the $C_2$ constraint ($\angle CCNN_1 = \angle CCNN_2$), because +90° and −90° describe the same ring orientation due to the 180° periodicity of ∠CCNN arising from the local $C_2$ symmetry of each phenyl ring (as discussed in Supplementary Note 11). The $I_1$ structure is therefore consistent with approximate $C_{2h}$ symmetry, the same point group as the *trans*-AB ground state. In other words, the *trans*-AB → $I_1$ transition preserves the molecular symmetry: the phenyl rings twist out of the molecular plane by ~90°, but the symmetry elements ($C_2$ and $i$) are both maintained within the experimental uncertainty.

This symmetry is broken in the subsequent $I_1$ → $I_2$ transition, where a substantial ∠CNNC rotation occurs. For $I_1$ → $I_2$, Extended Data Fig. 6b plots the corresponding SSPM and BIC results. In the unconstrained analysis, ROT + CNT ($k + 4 = 7$) yields the absolute

minimum BIC($\chi^2$) = 201.4. The seven free parameters are ∠CNNC (ROT), $\angle CCNN_1$ and $\angle CCNN_2$ (CNT, two independent parameters), $\sigma^2$, the scaling coefficient, and the two baseline coefficients $a_n$ and $b_n$. The full four-motion model ($k + 4 = 10$, BIC($\chi^2$) = 213.0, ΔBIC($\chi^2$) = 11.6) showed negligible improvement, confirming that neither NND nor INV is statistically required for this transition.

The primary structural parameters (∠CNNC, ∠CCNN, ∠CNN, $r_{NN}$, $d_{TL}$, and $R_g$) of the refined $I_2$ structure are also listed in Extended Data Table 3. The ∠CNNC dihedral rotates to 115.5°, departing from the 180° value maintained through the *trans*-AB → $I_1$ step. This non-trivial ∠CNNC value breaks the inversion symmetry that was preserved in $I_1$: inversion maps the CNNC dihedral to its negative, so that *i* can be a symmetry element only when ∠CNNC = 0° or 180°. With ∠CNNC = 115.5°, the inversion centre is lost. Remarkably, although no symmetry constraint was imposed during the $I_1 \rightarrow I_2$ fitting, the two independently optimized CCNN angles converged to equal values of the same sign ($\angle CCNN_1 = \angle CCNN_2 = -71.9°$, difference < 0.01°). Because this equality was obtained with the two angles free to refine independently and with no symmetry imposed, it provides strong evidence that the experimental data themselves favour a symmetric arrangement of the two ∠CCNN angles. The two candidate symmetry elements, a two-fold rotation axis and an inversion centre, are distinguished by how they relate the two ∠CCNN angles. A two-fold rotation maps one angle onto the other while preserving its sign, so that $C_2$ symmetry requires $\angle CCNN_1 = \angle CCNN_2$. An inversion centre instead maps the angle onto its negative, so that it requires $\angle CCNN_1 = -\angle CCNN_2$. The refined angles, which are equal and of the same sign, are therefore the signature of $C_2$ symmetry rather than of an inversion centre. Together with the loss of the inversion centre at ∠CNNC = 115.5°, the equal, same-sign ∠CCNN angles establish the molecular symmetry of $I_2$ as $C_2$: the two-fold rotation axis is retained, but the inversion centre is not. The symmetry is thereby lowered from the approximate $C_{2h}$ of $I_1$ to $C_2$, the same point group as the *cis*-AB product. The $I_1 \rightarrow I_2$ step is thus the point at which the molecular symmetry switches from the *trans*-like $C_{2h}$ to the *cis*-like $C_2$, driven by the onset of ∠CNNC rotation. The spontaneous emergence of this *cis*-like $C_2$ symmetry from a fit in which no symmetry was imposed provides strong, model-independent evidence that $I_2$ has already acquired the symmetry character of the *cis*-AB product.

**Supplementary Note 4 | Discussion on the basis motions analysed by BMA and MoCA**

The structural evolution from *trans*-AB to *cis*-AB was systematically examined through a comparative BMA and MoCA of the TRXL-refined and CASPT2-calculated geometries, focusing on four dominant, candidate internal motions, ROT, INV, NND, and CNT, and their possible combinations (Supplementary Tables 3−5; Extended Data Fig. 7). BMA combined with BIC analysis of the TRXL-refined $I_1$ and $I_2$ structures fully confirmed the SSPM conclusion: the *trans*-AB → $I_1$ transition is optimally described by a single CNT motion (RMSD: < 0.0001 Å, the lowest BIC), and the $I_1$ → $I_2$ transition by CNT + ROT (RMSD: < 0.0001 Å, the lowest BIC), both giving the lowest RMSD and BIC values among all candidates. Because SSPM was not used for the $I_2$ → *cis*-AB step, BMA was essential for identifying the responsible motions. It showed that the combination of ROT, INV, and CNT best explains this transition, with the lowest BIC (−563.1) and an RMSD of 0.0299 Å. Furthermore, MoCA provided a quantitative breakdown of these motions: 100% CNT for *trans*-AB → $I_1$, 67% ROT and 33% CNT for $I_1$ → $I_2$, and 56% ROT, 39% INV, and 5% CNT for $I_2$ → *cis*-AB.

The same BMA, BIC, and MoCA analyses applied to the CASPT2-calculated structures indicate that a larger number of collective motions are involved and that their patterns differ substantially from those obtained from the TRXL-refined structures. Specifically, the *trans*-AB → $I_1$ transition is best represented by a combination of ROT (69%), INV (22%), NND (3%), and CNT (6%), rather than by a single motion (RMSD: 0.0283 Å, the lowest BIC). The $I_1$ → $I_2$ transition is dominated by ROT (90%) but additionally requires minor contributions from INV (7%), NND (1%), and CNT (2%) (RMSD: 0.0073 Å, the lowest BIC). Finally, the $I_2$ → *cis*-AB transition is described by three motions, ROT (43%), INV (5%), and CNT (52%) (RMSD: 0.0262 Å, the lowest BIC). The largest RMSD among these steps remains small (~0.03 Å), reflecting internally consistent geometries but requiring at least three distinct types of motion to reproduce the entire reaction coordinate. Considering the relative contributions of motions reveals that ROT contributes significantly for all three steps (69% for *trans*-AB → $I_1$, 90% for $I_1$ → $I_2$, and 43% for $I_2$ → *cis*-AB) and the contribution of CNT becomes important for the last step (52% for $I_2$ → *cis*-AB). Thus, a minimal two-motion representation, ROT for *trans*-AB → $I_1$ → $I_2$ and ROT + CNT for $I_2$ → *cis*-AB, reasonably approximates the CASPT2 pathway, which contrasts with the TRXL-determined pathway, where CNT is dominant only in the first step (100% for *trans*-AB → $I_1$, 33% for $I_1$ → $I_2$, and 5% for $I_2$ → *cis*-AB).

**Supplementary Note 5 | Comparison of present CASPT2-calculated structures with previously reported theoretical structures**

Azobenzene has long served as a prototypical system for the study of photoisomerization, and the mechanism of its nπ* isomerization has been studied by quantum-chemical calculations for four decades. Over this period, successive studies have applied progressively higher levels of theory as quantum-chemical methods advanced (Extended Data Table 2)[54-76]. Extended Data Table 2 compiles 23 theoretical studies reported to date, which fall into four categories listed in its Research Focus column: nine potential-energy-surface (PES) calculations[54-56,58,60,62,66,67,69], nine nonadiabatic molecular dynamics (NAMD) simulations[57,59,61,63-65,70,71,75], four studies that combine PES calculations with nonadiabatic wave-packet dynamics[72-74,76], and one global-reaction-coordinate calculation[68].

In contrast to the experimental studies compiled in Extended Data Table 1, which remain divided among competing mechanistic interpretations, recent theoretical studies have largely converged on a single mechanism, inversion-assisted rotation, either as proposed in the original study or as interpreted in the present work[69-75]. The sole recent exception instead proposes rotation-assisted inversion, reversing the relative ordering of the rotational and inversion motions[68]. None of these studies, however, identified C–N bond torsion (CNT), the torsion of each phenyl ring about its C–N bond axis, as a significant component of the isomerization coordinate. The BMA of the TRXL-refined structures reveals that CNT is the dominant motion for the *trans*-AB → $I_1$ transition and contributes to every subsequent step of the reaction pathway (Supplementary Table 5). A motion overlooked by all of these calculations thus emerges from the TRXL experiment as the defining structural change of the first step.

To test whether this discrepancy stems from the level of theory used in the earlier calculations, we characterized the excited-state landscape with our own high-level CASPT2 calculations, using an extended active space and a refined basis set (see Methods). The resulting $S_1$ PES exhibits two well-defined minima between the Franck–Condon (FC) region and the conical intersection leading to $S_0$. These minima correspond to $I_1$ and $I_2$ and are consistent with the sequential three-step kinetic model. The CASPT2 calculations performed in the present work can likewise be classified as inversion-assisted rotation (see below).

At the CASPT2-calculated $I_1$ minimum of the present work, the two ∠CNN angles increase from 113.4° in *trans*-AB to 128.1° and 128.0°, whereas ∠CNNC decreases from 180.0°

to 168.4°. At the CASPT2-calculated $I_2$ minimum, ∠CNNC decreases further to 117.9°, while both ∠CNN angles remain enlarged at approximately 124.1°. Within the sequential $I_1$-to-$I_2$ assignment, the changes in ∠CNN therefore precede the larger change in ∠CNNC and remain substantial as ∠CNNC rotation proceeds. This sequence is conventionally associated with inversion-assisted rotation.

In Extended Data Table 2, we indicate whether each study detected or merely mentioned an excited-state structure with *trans*-like or conical-intersection (CI)-like character. We assigned these two categories, listed as $S_1$ *trans*-like structure and $S_1$ CI-like structure, based on qualitative geometric criteria. Specifically, a minimum that retains a geometry close to that of *trans*-AB was classified as *trans*-like, whereas a minimum in which ∠CNNC is substantially rotated towards an $S_1/S_0$ conical intersection was classified as CI-like. The CASPT2-calculated $I_1$ falls into the *trans*-like category and the CASPT2-calculated $I_2$ into the CI-like category, so that a minimum reported in an earlier study can be compared with $I_1$ or with $I_2$ according to the category to which that minimum belongs.

Although the present calculations and many recent theoretical studies are both classified as inversion-assisted rotation, that classification does not mean that the underlying structures are the same. The reported excited-state geometries are compared below. For each of the four categories of calculation listed in the Research Focus column of Extended Data Table 2, we take the most recent study as representative and compare it with the present calculations. Such a comparison requires an optimized excited-state minimum, which not every study classified as inversion-assisted rotation reports. An optimized excited-state minimum is reported for only two of the four quantum-chemical methods, as listed for these studies in the "$S_1$ *trans*-like structure" and "$S_1$ CI-like structure" columns of Extended Data Table 2. The global-reaction-coordinate calculation did not report an optimized excited-state minimum[68]. Likewise, the four studies combining PES calculations with nonadiabatic wave-packet dynamics did not report optimized excited-state minima[72-74,76], because those studies focused on propagating nuclear wave packets on precomputed PESs rather than on locating stationary points. The most recent NAMD study, based on multiple spawning[75], reported an optimized *trans*-like excited-state minimum corresponding to $I_1$, but no second CI-like minimum corresponding to $I_2$.

The closest correspondence with the present calculations is therefore found among the studies classified as "PES calculations" in Extended Data Table 2, the category to which the

present work itself belongs. In particular, the most recent of those studies, a RASPT2 study[69], identified both a *trans*-like and a CI-like excited-state minimum, as the present calculations do for $I_1$ and $I_2$. This correspondence may reflect the methodological similarity between the two studies, as both employ multireference second-order perturbation theory to characterize the excited-state surface, with particular attention to the ∠CNNC and ∠CNN coordinates.

The two studies differ, however, in how the reported minima were obtained. In the present CASPT2 calculations, both minima were obtained by full geometry optimization directly at the XMS-CASPT2 level, with symmetry constraints disabled and all internal coordinates allowed to relax (Methods). In the RASPT2 study, by contrast, only one of the two minima was fully optimized: the CI-like minimum, which corresponds to $I_2$ in the present assignment, was obtained by full geometry optimization, whereas the *trans*-like structure, corresponding to $I_1$, was identified within a rigid scan in which the two ∠CNN angles were constrained to remain equal and all other internal coordinates were held fixed[69]. The resulting *trans*-like structure therefore represents a point on a rigid scan rather than a fully optimized stationary point. The two sets of geometries nonetheless agree at the qualitative level, both showing the enlargement of the ∠CNN angles and the reduction of ∠CNNC that characterize inversion-assisted rotation. For the *trans*-like minimum, the RASPT2 study gives ∠CNNC = 180° and ∠CNN = 126°, compared with ∠CNNC = 168.4° and ∠CNN = 128.1° for the CASPT2-calculated $I_1$ structure reported here. For the CI-like minimum, the RASPT2 study gives ∠CNNC = 96° and ∠CNN of 115° and 145°, compared with ∠CNNC = 117.9° and ∠CNN of 124.1° for the CASPT2-calculated $I_2$ structure. The agreement is closer for the *trans*-like minimum than for the CI-like minimum. Despite being obtained from a rigid scan, the *trans*-like structures from the RASPT2 study reproduce the essential geometric features of our fully optimized $I_1$.

**Supplementary Note 6 | Comparison of quantum chemically predicted and TRXL-refined intermediate structures**

Extended Data Table 3 compares the structural parameters of the intermediates determined from fs-TRXL experiments with those derived from high-level quantum chemical calculations using CASPT2. To assess the reliability of the CASPT2-calculated structures, we simulated theoretical SADSs (SADS′s) from these structures and compared the SADS′s with experimental SADSs. While SADS′s captured the overall features of the experimental SADSs,

quantitative discrepancies were evident (Fig. 3f), motivating a detailed parameter-level comparison.

For $I_1$, the most prominent difference between the TRXL-refined and CASPT2-calculated $I_1$ structures lies in the ∠CCNN angles. The TRXL-refined $I_1$ exhibits large ∠CCNN angles (∠$CCNN_1$ = 87.0°, ∠$CCNN_2$ = −87.0°), corresponding to a near-90° twist between the phenyl rings and the CNN plane about the C–N bond axes. We note that an identical ∠CCNN change can, in principle, be produced by torsion of the phenyl rings alone, counter-rotation of the azo framework, or a concerted combination of both (as in a bicycle-pedal-like motion); the present scattering data, which are sensitive to interatomic distances rather than absolute orientations, cannot distinguish among these scenarios. In contrast, the CASPT2-calculated $I_1$ geometry has near-zero ∠CCNN angles (∠CCNN = 0.7°), retaining a nearly planar ring orientation. This ~87° discrepancy indicates that the CNT degree of freedom, which the SSPM analysis identifies as the sole motion required to describe the *trans*-AB → $I_1$ transition, is not captured by the CASPT2 excited-state geometry optimization. The two methods also disagree on the ∠CNN inversion angles: CASPT2 predicts substantial bending at the $I_1$ stage (∠$CNN_1$ = 128.1°, ∠$CNN_2$ = 128.0°, compared with 113.4° in *trans*-AB), whereas the ∠CNN angles in the TRXL-refined structure remain at 113.4°, unchanged from *trans*-AB (∠CNN = 113.4°), consistent with the SSPM result that INV is not active in the *trans*-AB → $I_1$ transition. The ∠CNNC dihedral also differs (180.0° from TRXL vs 168.4° from CASPT2), with the TRXL result indicating that no N–N bond rotation has occurred at the $I_1$ stage. The N–N bond lengths show a small difference (1.276 Å from TRXL vs 1.264 Å from CASPT2). In summary, the CASPT2 calculation predicts that the initial structural response to n$\pi$* excitation involves rotation (∠CNNC change) and inversion (∠CNN change) with minimal ring twist, consistent with an inversion-assisted rotational pathway, whereas the TRXL data indicate the opposite: the initial response is dominated by ring twist (∠CCNN change) with no rotation or inversion.

For $I_2$, the TRXL-refined and CASPT2-calculated structures agree in some parameters but diverge sharply in others. The ∠CNNC dihedral angles agree closely (115.5° from TRXL vs 117.9° from CASPT2, a difference of only 2.4°), indicating that both methods converge on a similar degree of N–N bond rotation at the $I_2$ stage. The N–N bond lengths are also essentially identical (1.276 Å vs 1.277 Å), and the $d_{TL}$ values are comparable (0.700 Å vs 0.591 Å). In stark contrast, the ∠CCNN angles differ dramatically: the TRXL-refined values (∠$CCNN_1$ = ∠$CCNN_2$ = −71.9°) indicate a large, persistent phenyl-ring torsion, whereas the CASPT2

values (∠$CCNN_1$ = −0.8°, ∠$CCNN_2$ = −0.7°) are near zero. This ~72° discrepancy, comparable in magnitude to that observed for $I_1$, confirms that the CNT degree of freedom is systematically absent from the CASPT2-calculated excited-state geometries. The ∠CNN bending angles show a moderate discrepancy (113.4° from TRXL vs 124.1° from CASPT2), with CASPT2 predicting significant inversion at the $I_2$ stage that is not supported by the TRXL analysis. The TRXL result instead assigns the inversion motion to the subsequent $I_2$ → *cis*-AB transition.

Taken together, the comparison reveals a consistent pattern across both intermediates: CASPT2 systematically underestimates the ∠CCNN angles by ~72–87° while overestimating the ∠CNN inversion and, in the case of $I_1$, the ∠CNNC rotation. These discrepancies likely reflect the inherent difficulty of accurately describing the $S_1$(nπ*) PES of azobenzene, where the relative energetics of the ∠CNNC, ∠CNN, and ∠CCNN coordinates are sensitive to the level of theory, the active space, and the treatment of dynamic electron correlation. The CNT coordinate, in particular, involves a relatively soft torsional degree of freedom with a shallow potential, making it susceptible to even small errors in the calculated energy surface. Solution-phase effects, which are not included in the gas-phase CASPT2 optimizations, may also play a role, as the solvent environment can preferentially stabilize certain conformations over others. These observations underscore the value of direct experimental structural determination for excited-state intermediates, where purely computational approaches, especially at current levels of theory, may not reliably capture all relevant structural degrees of freedom.

The *cis*-AB product structure shows better agreement between TRXL and CASPT2 than the excited-state intermediates. The ∠CNNC dihedral angles agree within the experimental uncertainty (10.8 ± 7.4° from TRXL vs 6.0° from CASPT2), and the $d_{TL}$ values are nearly identical (1.252 Å from TRXL vs 1.256 Å from CASPT2). The ∠CCNN angles show a moderate discrepancy (77.0°, 77.3° ± 4.4° from TRXL vs 56.1°, 56.4° from CASPT2), which is smaller than the ~72–87° differences observed for $I_1$ and $I_2$. However, the closer agreement for *cis*-AB should be interpreted with the caveat that the Tikhonov regularization employed in the structural refinement of *cis*-AB (Supplementary Methods 12.4 and 12.7) constrains the refined structure to remain in the vicinity of the CASPT2 reference geometry, limiting the extent to which the TRXL data can drive the structure away from the computational prediction.

To further compare the TRXL and CASPT2 descriptions of the isomerization pathway, we performed BMA analysis on both sets of structures to decompose each structural transition into its constituent motions (Supplementary Table 5). For the TRXL-refined pathway, the

motion sequence is CNT (100%) → ROT (67%) + CNT (33%) → ROT (56%) + INV (39%) for the *trans*-AB → $I_1$, $I_1$ → $I_2$, and $I_2$ → *cis*-AB transitions, respectively. For the CASPT2-calculated pathway, the corresponding sequence is ROT (69%) + INV (22%) → ROT (90%) → ROT (43%) + CNT (52%).

The two pathways share an important common feature: ROT dominates the central $I_1$ → $I_2$ step in both cases (67% in TRXL, 90% in CASPT2), indicating that both methods agree that the major N–N bond torsion occurs at this stage. However, the ordering of the other motions is strikingly different. In the TRXL-refined pathway, CNT initiates the isomerization (100% of the *trans*-AB → $I_1$ step), with INV contributing only in the final $I_2$ → *cis*-AB step (39%). In the CASPT2-calculated pathway, this sequence is essentially reversed: INV contributes early (22% of *trans*-AB → $I_1$), while CNT appears predominantly in the final step (52% of $I_2$ → *cis*-AB). Thus, although both descriptions involve a similar (largely overlapping) set of elementary motions, the temporal ordering is fundamentally different: the TRXL data support a CNT-first, INV-last sequence, whereas CASPT2 predicts an INV-first, CNT-last sequence.

This reversal is consistent with the parameter-level discrepancies discussed above. The large ∠CCNN values observed in the TRXL-refined $I_1$ and $I_2$ structures, which are absent in the corresponding CASPT2 geometries, reflect the early activation of CNT in the experimental pathway. Conversely, the large ∠CNN changes predicted by CASPT2 at the $I_1$ stage, which are not observed experimentally, correspond to the early INV predicted by the CASPT2 pathway. The CNT coordinate involves a relatively soft torsional potential that becomes accessible upon nπ* excitation and disruption of π-conjugation, whereas the INV coordinate requires bending at the nitrogen centres against a stiffer potential. The TRXL observation that CNT precedes INV suggests that the molecule follows the path of least resistance in conformational space, activating the softest available degree of freedom first.

**Supplementary Note 7 | Comparison of observed isomerization pathway with theoretical predictions**

The photoisomerization mechanisms proposed by previous theoretical studies on *trans*-AB upon nπ* excitation are summarized in Extended Data Table 2. Notably, most studies proposed a mechanism that can be classified as a rotation-driven mechanism[54,56-63,65-76], either a rotation

about the N–N bond (which some studies call torsion) or an inversion-assisted rotation. It should be noted that the "torsion" referred to in these studies denotes twisting about the N–N bond and is therefore equivalent to rotation (ROT), rather than the C–N torsion (CNT) defined in the present study; inversion-assisted rotation is a distinct mechanism that couples rotation with inversion. Two other studies argue for non-rotational mechanisms: one supports inversion[55], and the other proposes a pedal-like motion[64]. Despite the latest advances in quantum chemical calculations, the isomerization processes of azobenzene proposed by those studies are not perfectly aligned. Notably, none of the theoretically proposed pathways correspond perfectly to the one revealed by our TRXL experiment. Over the past decades, theoretical studies have mostly supported an inversion-assisted rotation mechanism, where rotation involving changes in the ∠CNNC is facilitated by changes in the ∠CNNs. However, this mechanism is not consistent with the TRXL results. In this context, the experimentally identified pathway may serve as a valuable reference for future theoretical investigations into chemical reaction dynamics.

Several previous theoretical studies have reported a reaction intermediate that resembles our TRXL-derived $I_1$ in terms of either kinetics or structure.[57,69,75] For example, a combined fs-TA and quantum chemical study[4] reported relaxation to an intermediate state with a time constant of 0.3 ps, which closely aligns with the 0.4 ps time constant observed in the TRXL data; the accompanying XMCQDPT2 calculations identified this state as a local minimum, named $S^L_{1t}$, on the $S_1$ surface near the FC region, with a planar $C_{2h}$-symmetric structure closely resembling the azo-backbone geometry of $I_1$ obtained from TRXL, which likewise retains a ∠CNNC of 180.0° ± 6.0°. Further support comes from RASPT2 PES calculations,[69] which also identified an "$S_1$ *trans* minimum", with ∠CNNC retained at 180°, consistent with the $I_1$ state observed using TRXL. Additionally, a CASPT2 study proposed a *trans*-like minimum on the $S_1$ surface, termed "E-Ab ($S_1$)", with a ∠CNNC of 180°, aligning with our $I_1$ structure[77].

In contrast to previous studies supporting $I_1$, the characterization of a second intermediate analogous to $I_2$ has received less attention, though recent theoretical work is beginning to shed light on its properties. Several theoretical investigations suggest the presence of an $I_2$-like intermediate[57,58,62,64,65,67,69]. For instance, one study employing RASPT2 calculations identified an "optimized $S_1$ minimum" with a ∠CNNC of 96°.[69] This value is fairly close to the 115.5° ∠CNNC observed in our $I_2$ structure determined by TRXL, and both

structures are distinct from *trans*-AB and $I_1$, which have a ∠CNNC of 180°. Moreover, one of the two ∠CNN angles of the "optimized $S_1$ minimum" (115°) is close to those of our $I_2$ obtained from TRXL (113.4° and 113.4°), whereas the other (145°) is larger. Another CASPT2 study[67] reported an "$S_1$(nπ*)-min" state with a CI-like conformation (∠CNNC = 90.1°), also exhibiting structural similarities to our $I_2$ determined through TRXL, in that the ∠CNNC is rotated away from the 180° of *trans*-AB and $I_1$ towards the 115.5° ± 1.7° of $I_2$.

**Supplementary Note 8 | Comparison of observed isomerization pathway with spectroscopic assignments**

Numerous time-resolved spectroscopic studies have investigated the *trans*-to-*cis* isomerization of azobenzene upon nπ* excitation. However, significant discrepancies persist regarding the number of intermediates and the specific mechanisms involved (Extended Data Table 1). Even recent studies remain inconclusive about the exact mechanism.[2-4,47-53] Specifically, two studies support the rotation mechanism[3,47], three favour the inversion mechanism[49,50,52], one suggests the hula-twist mechanism[4], one suggests the inversion-assisted rotation mechanism[53], and three did not assign any specific mechanism[2,48,51]. This lack of consensus underscores the challenges in interpreting the photoisomerization of azobenzene, which, according to the complex structural dynamics revealed in this study, cannot be easily categorized within a simple, classical mechanistic framework such as rotation, inversion, and hula-twist. It is important to note that the assignments made by previous studies are not entirely incorrect. Our structural analysis suggests that these seemingly conflicting assignments rather represent partial views of the full reaction coordinate. Specifically, studies that assigned the mechanism to "rotation" likely detected the large rotational motion associated with the ∠CNNC change, which is indeed a major component of our observed pathway. Similarly, studies supporting the "inversion" mechanism may have been sensitive to the ∠CNN changes that contribute to the later stage of the isomerization ($I_2$ → *cis*-AB, where INV accounts for 39% of the motional contribution), rather than the early steps. The structural dynamics we observed suggest that the *trans*-to-*cis* isomerization of azobenzene is not solely driven by rotation but also involves significant contributions from C–N torsion associated with ∠CCNN changes, particularly in the initial step towards $I_1$, whereas changes in ∠CNN are not apparent in the early steps (*trans*-AB → $I_1$ and $I_1$ → $I_2$).

Regarding the kinetics of the isomerization, the time constants observed in this work

are 0.4 ps ($\tau_1$), 2.7 ps ($\tau_2$), and 14 ps ($\tau_3$). Spectroscopy studies have reported varying subsets of these time constants, depending on the technique employed. The assignments of the observed time constants also show discrepancies among studies, particularly for $\tau_2$ and $\tau_3$. Even among the studies reporting all three time constants, the interpretation of $\tau_3$ remains controversial. Two transient absorption (TA) studies, reported in 2003[2] and 2020[53], attributed $\tau_3$ to vibrational cooling of the hot $S_0$ state (*trans*-AB and *cis*-AB), whereas a TA study reported in 2014[4] assigned it to the transformation from a second intermediate, $S_1^D$, to *trans*-AB and *cis*-AB. This latter assignment aligns with our findings. Moreover, the number of intermediates identified in the 2014 TA study matches our observations. Therefore, our TRXL results are consistent with those of the 2014 TA study[4] in terms of both the number of intermediates and their kinetic assignments. However, we note that previous spectroscopic studies have not addressed the collective action of C–N torsion and rotation motions revealed by our TRXL study.

**Supplementary Note 9 | Quantitative assessment of volume-conserving character using radius of gyration**

One cannot rely solely on changes in bond lengths or bond angles during the photoisomerization process to capture the overall volumetric characteristics of the molecule and provide a quantitative measure of the volume-conserving character of *trans*-AB. The structural compression or expansion of intermediates is directly linked to the overall volume occupied by the molecule. In this respect, the radius of gyration ($R_g$), which represents how far the atoms are distributed from the molecular centre of mass, serves as an appropriate metric to quantitatively compare the volume-conserving character of each intermediate. Therefore, we analysed the $R_g$ values obtained from TRXL-refined and CASPT2-calculated structures to evaluate how each intermediate along the isomerization pathway differs in volumetric character.

The $R_g$ for each molecular structure was calculated using the commonly used mass-weighted average model, according to the following equation.

$$R_g = \sqrt{\frac{1}{M}\sum_i m_i |\boldsymbol{r}_i - \boldsymbol{r}_{cm}|^2} \quad \text{(S38)}$$

Here, $M$ is the total mass of the molecule, $m_i$ is the mass of the $i$-th atom, $\boldsymbol{r}_i$ is the coordinate of the $i$-th atom, and $\boldsymbol{r}_{cm}$ is the coordinate of the molecular centre of mass.

Fig. 4a shows $R_g$ values obtained from TRXL-refined and CASPT2-calculated structures of *trans*-AB, $I_1$, $I_2$, and *cis*-AB. For *trans*-AB, the TRXL analysis uses the CASPT2-calculated ground-state geometry as the reference structure ($R_g$ = 3.26 Å). For *cis*-AB, the TRXL-refined and CASPT2-calculated structures yield nearly identical $R_g$ values (2.61 Å vs 2.60 Å), consistent with the close agreement between the two geometries discussed in Supplementary Note 6. In contrast, the TRXL-refined $I_1$ and $I_2$ structures are both more compact than their CASPT2 counterparts: $R_g$ decreases from 3.30 Å (CASPT2) to 3.24 Å (TRXL) for $I_1$ and from 3.14 Å (CASPT2) to 3.06 Å (TRXL) for $I_2$. This systematic reduction in $R_g$ for the excited-state intermediates reflects the large CNT component in the TRXL-refined structures, which orients the phenyl rings inward, producing more compact geometries than the nearly planar CASPT2-calculated structures that lack this motion. This deviation provides direct quantitative evidence for the volume-conserving nature of these key intermediates during the isomerization process.

We note that the large ∠CCNN values (~72–87°) in $I_1$ and $I_2$ orient the phenyl rings approximately perpendicular to the CNN plane, which may further reduce the hydrodynamic cross-section during the ROT step. A quantitative assessment of this effect would require MD simulations with explicit solvent, which is beyond the scope of the present study.

## Supplementary Note 10 | Comparison of TRXL and spectroscopy results on the determination of the *cis*/*trans* product fractions

According to the branching yields derived from the structural refinement (Supplementary Method 12.3), the $I_1 \rightarrow I_2$ transition proceeds with unity yield within error (100 ± 19%), whereas the $I_2 \rightarrow$ *cis*-AB yield is 91 ± 7%. The near-unity $I_1 \rightarrow I_2$ yield indicates that essentially all $I_1$ molecules proceed forward along the isomerization pathway, with no detectable branching back to the *trans*-AB ground state at this stage. For the $I_2$ stage, the sub-unity central value of $\gamma = 0.91$ raises the possibility that a minor fraction (~9%) of the $I_2$ population returns to the *trans*-AB ground state rather than proceeding to *cis*-AB. Within a competing first-order kinetic framework, the observed apparent time constant $\tau_3$ = 14 ps corresponds to the sum of the forward and backward rate constants ($k_{obs} = k_{cis} + k_{trans}$). Decomposing using the branching yield yields intrinsic time constants of $\tau_{cis} = \tau_3 / \gamma \approx 15 \pm 5$ ps for the productive $I_2 \rightarrow$ *cis*-AB channel and $\tau_{trans} = \tau_3 / (1 - \gamma) \approx 161$ ps (lower-bounded at ~110 ps, with a poorly constrained upper limit because $\gamma$ is close to unity) for the non-productive $I_2 \rightarrow$ *trans*-AB channel, meaning

that the back-reaction channel, if it exists, is roughly an order of magnitude slower than the forward channel. We note that $\gamma$ is also consistent with unity within the experimental uncertainty.

Meanwhile, only a few time-resolved spectroscopic studies have addressed the *cis*/*trans* product fractions[2,3,47,50,52]. The 2014 TA study[4] quantitatively analysed the nπ*-excited dynamics by comparing the ground-state bleaching and the excited-state absorption signals from the *cis*-AB product obtained from TA measurements with the static absorption spectra of *trans*- and *cis*-AB, thereby reporting *cis*/*trans* product fractions of 0.32 / 0.68 in methanol, the same solvent used in our TRXL experiment. Combining this spectroscopic quantum yield ($\Phi_{cis}$ = 0.32) with the branching yields determined from the TRXL structural refinement ($\beta \approx 1.00$, $\gamma = 0.91$; Supplementary Method 12.3) provides insight into where along the reaction pathway the dominant loss of quantum efficiency occurs. Since the overall yield from $I_1$ onward is $\beta \cdot \gamma \approx 0.91$, the fraction of photoexcited molecules that enter the productive $I_1$ pathway is $\Phi_{cis} / (\beta \cdot \gamma) = 0.32 / 0.91 \approx 0.35$. This implies that the FC state bifurcates into approximately 65% through the FC → *trans*-AB relaxation pathway and 35% through the FC → $I_1$ pathway leading to *cis*-AB. The near-unity values of $\beta$ and $\gamma$ indicate that once a molecule enters the $I_1$ pathway, it proceeds to *cis*-AB with high efficiency, and the dominant source of quantum yield loss is the initial partitioning at the FC region, not the branching at the $I_1$ and $I_2$ stages. It should be noted that the FC state has essentially the same geometry as the ground state. The decay of the FC state back to *trans*-AB is therefore not accompanied by any appreciable change in the structure of the solute. Because our structural analysis is based on the signal that arises from a structural change of the solute, this channel does not produce such a signal and is consequently not resolved in that analysis. This does not mean, however, that the FC → *trans*-AB channel is absent from the measured data. Each molecule that returns to *trans*-AB along this channel releases the absorbed photon energy as heat, which is transferred to the surrounding solvent. The resulting heating of the solvent would contribute to the TRXL signal as a hydrodynamic response. This contribution is indeed observable. However, the FC → *trans*-AB channel is not the only process that releases energy into the solvent: energy is also released along the reactive pathway, for example during the FC → $I_1$ and $I_1$ → $I_2$ transitions, and the vibrationally hot intermediates relax further by transferring their excess energy to the surrounding solvent. Because all of these processes contribute to the same hydrodynamic response, it is not straightforward to isolate the part that arises specifically from the FC → *trans*-AB channel. Although this channel is therefore difficult to characterize directly from the TRXL data, its existence is independently supported by a previous fluorescence study[2] of *trans*-

AB, which reported ultrafast fluorescence originating from the FC region.

**Supplementary Note 11 | Cartesian realization of internal coordinate changes**

The structural transitions of azobenzene are characterized by changes in internal coordinates (∠CNNC, ∠CCNN, ∠CNN, $r_{NN}$) determined from the SSPM analysis. However, mapping these internal coordinate changes onto atomic displacements in three-dimensional Cartesian space is not straightforward, because a given set of internal coordinate changes can be realized by multiple distinct atomic rearrangements in the laboratory frame, depending on which atoms move and which remain stationary. In solution-phase reactions, solute–solvent friction is a key factor governing reaction kinetics, and the magnitude of this friction depends on how much solvent is physically displaced as the solute rearranges. Estimating this solvent displacement requires knowledge of the atomic motions in the laboratory frame, not merely the internal coordinate changes. To address this, we compared two limiting scenarios for each structural transition and quantified the solvent volume swept in each case.

The first scenario represents the most intuitive picture of how internal coordinate changes translate into real-space motion: the azo bridge ($N_1$ and $N_2$) remains stationary in the laboratory frame while the phenyl rings rotate to accommodate the full extent of the internal coordinate changes. This is the natural visualization that arises when one thinks of internal coordinate changes. For instance, a ∠CNNC change corresponds to one ring rotating about the N–N axis, a ∠CCNN change to a ring twisting about its C–N bond, and a ∠CNN change to a ring bending relative to the N–N direction, without displacement of the nitrogen atoms. Because the azobenzene molecule maintains an approximate $C_2$ symmetry, a two-fold axis relating its two phenyl rings, throughout the isomerization ($I_2$ and *cis*-AB have $C_2$ symmetry, and $I_1$ has approximate $C_{2h}$ symmetry, which contains this same two-fold axis; Supplementary Method 12.5 and Supplementary Note 3), we imposed $C_2$ symmetry on the trajectory so that both rings share each internal coordinate change equally, rather than assigning all displacement to one ring while the other remains stationary.

The second scenario asks: what is the most economical way to connect two structures in the laboratory frame? Rather than holding any part of the molecule fixed, we allow all atoms to move and seek the rearrangement that minimizes the overall atomic displacement. This is obtained by all-atom Kabsch superposition[78] of each intermediate structure onto its predecessor, which removes overall translation and rotation and yields the residual displacement that cannot

be eliminated by rigid-body motion. In this frame, the nitrogen atoms are no longer stationary; they participate in the rearrangement along with the phenyl rings. If this minimum-displacement rearrangement sweeps substantially less solvent than the N–N fixed scenario, it implies that the N–N fixed picture overestimates the solvent perturbation, and that a more concerted rearrangement involving the azo bridge is a more physically realistic description of the solution-phase process.

For each transition (*trans*-AB → $I_1$, $I_1$ → $I_2$, and $I_2$ → *cis*-AB), 31 intermediate structures were generated by linear interpolation of the six active internal coordinates. At each interpolation step, the phenyl rings were treated as rigid bodies to preserve the internal ring geometry. Because each phenyl ring possesses local $C_2$ symmetry about the C–N bond axis, ∠CCNN = $\alpha$ and ∠CCNN = $\alpha$ + 180° describe equivalent ring orientations; ∠CCNN was therefore treated as 180°-periodic, and the shortest-path convention was adopted to avoid artificially large ring rotations. For example, the $I_1$ → $I_2$ transition involves a change in ∠CCNN from 87.0° to −71.9°; without accounting for the 180° periodicity, this would appear as a 158.9° rotation, whereas the shortest path is only 21.1° (since −71.9° and 108.1° describe the same ring orientation, giving 108.1° − 87.0° = 21.1°). ∠CNNC, which describes rotation about the N=N bond, was treated with standard 360° periodicity, because it has no analogous symmetry: ∠CNNC = 0° (*cis*) and ∠CNNC = 180° (*trans*) are physically distinct structures, so a value and its 180° counterpart are not equivalent.

The solvent-swept volume was estimated using a grid-based approach designed to provide a physically meaningful measure of the solvent volume displaced by the molecular rearrangement. A three-dimensional grid with a spacing of 0.15 Å was constructed around the molecular trajectory. For each frame along the interpolated pathway, the van der Waals sphere of every atom was marked on the grid using the Bondi radii (C: 1.70 Å, N: 1.55 Å, H: 1.20 Å[79]). Each voxel was marked as occupied if any atom's van der Waals sphere overlapped with it at any point along the trajectory. The swept volume was then calculated as the total volume of voxels that were occupied during the trajectory but not in the initial (reactant) structure, representing the new solvent space that the molecule invaded during the transition; this definition excludes the static molecular volume (~175 $Å^3$) that the reactant already occupies.

The results are summarized in Supplementary Table 7. The minimum-displacement pathway sweeps substantially less solvent than the N–N fixed pathway for the *trans*-AB → $I_1$ and $I_1$ → $I_2$ transitions (81% and 44% reduction, respectively), but the two frames give nearly

identical swept volumes for $I_2$ → *cis*-AB (~0.5% reduction). This trend has a clear physical origin. The *trans*-AB → $I_1$ transition involves only ∠CCNN changes with no ∠CNNC rotation. In the N–N fixed frame, each ring must sweep through a large arc to achieve the near-90° torsion. In the minimum-displacement frame, the same ∠CCNN change is achieved with both rings sweeping much less volume, because the nitrogen atoms absorb a significant fraction of the displacement. Notably, we confirmed that the bicycle-pedal-like motion emerges naturally from the minimum-displacement analysis for the *trans*-AB → $I_1$ step: the Kabsch superposition, which simply minimizes the overall atomic displacement without imposing any specific type of molecular motion, yields a trajectory in which the azo bridge rearranges concertedly while the rings remain nearly stationary. That this motion is independently identified as the most efficient rearrangement, rather than assumed a priori, provides further support for the bicycle-pedal mechanism as the physically preferred pathway within the solvent cage.

The $I_1$ → $I_2$ transition involves a 64.5° ∠CNNC rotation accompanied by ∠CCNN adjustments of +21.1° and +15.1° for the two rings (180°-periodicity corrected; see Extended Data Table 3 footnote a). If $I_1$ had perfect $C_{2h}$ symmetry with ∠CCNN = 90°, both rings would undergo identical ∠CCNN changes of 18.1°. Here, the minimum-displacement pathway still achieves a 44% reduction in swept volume relative to the N–N fixed frame, indicating that concerted rearrangement remains effective even when ∠CNNC rotation is the dominant motion. The ∠CCNN angles of $I_1$, which are near 90° and therefore position the phenyl rings approximately perpendicular to the C–N–N–C plane, are optimally configured for this: the rings are pre-oriented so that the subsequent ∠CNNC rotation can proceed with minimal additional ring displacement.

For the $I_2$ → *cis*-AB step, which requires a 104.7° ∠CNNC rotation along with 6° of ∠CNN bending and a 0.007 Å contraction of the N–N bond, the two frames give nearly identical swept volumes (114 vs 115 Å$^3$, ~0.5% reduction). This indicates that beyond a certain magnitude of dihedral rotation, the minimum-displacement pathway offers little advantage: the atoms must physically traverse a large region of space to reach the *cis* geometry, regardless of how the displacement is distributed among them.

Supplementary Table 8 lists the RMSD between the initial and final structures of each transition, computed in both the N–N fixed and minimum-displacement frames. In the N–N fixed frame, the RMSD reflects the displacement of the phenyl rings while the nitrogen atoms remain stationary, whereas in the minimum-displacement frame it reflects the most compact

rearrangement achievable. For the *trans*-AB → $I_1$ transition, the minimum-displacement-frame RMSD is only 0.40 Å, indicating that the near-90° ∠CCNN change can be accommodated with remarkably small atomic displacements when the entire molecule rearranges concertedly. The corresponding N–N fixed RMSD is five times larger (1.98 Å), illustrating how the choice of reference frame dramatically affects the apparent magnitude of the structural change. The ratio of N–N fixed to minimum-displacement RMSD decreases along the pathway (5.0 for *trans*-AB → $I_1$, 1.6 for $I_1$ → $I_2$, 1.3 for $I_2$ → *cis*-AB), mirroring the trend observed in the swept volume analysis: concerted rearrangement is most advantageous for the early, CNT-dominated steps and least so for the later steps dominated by large ∠CNNC rotation. The progressive increase in minimum-displacement-frame RMSD along the isomerization pathway (0.40, 1.10, and 2.31 Å for the three successive transitions) is consistent with the increasing swept volume and indicates that the early steps of the isomerization proceed with minimal perturbations in both solute structure and surrounding solvent cage, while the later steps necessarily involve larger-scale rearrangement.

**Supplementary Note 12 | Solute-only SADSs in real space**

Experimental and simulated SADSs for the solute-only term in real space (*r*-space), $r^2\mathrm{SADS}_{\mathrm{solute}}(r)$, are presented in Extended Data Fig. 5. This *r*-space representation serves as a complementary validation to the *q*-space data shown in Fig. 3f. While the *q*-space comparison in Fig. 3f includes contributions from both the solute and the cage terms, the *r*-space comparison presented here compares the experimental data with the calculated signal from the solute structure alone (excluding the cage term). In these difference RDFs, negative amplitudes indicate a loss of atomic pair density at the corresponding distance compared to *trans*-AB, while positive amplitudes indicate an increase in atomic pair density at the corresponding distance.

The *r*-space signal for $I_1$ is the weakest among the three SADSs, consistent with the relatively small structural change from *trans*-AB to $I_1$, which primarily involves a change in the ∠CCNN dihedral angle while the overall molecular framework remains largely intact. The difference RDF does not exhibit a single dominant feature but instead shows weak negative amplitudes at both short (~2.4 Å) and long (~9 Å) distances, with modest positive amplitudes at intermediate distances (~3–5 Å). This pattern reflects the breaking of molecular planarity: the loss of the planar *trans* geometry redistributes interatomic distances that were previously

at the extremes (the shortest and longest intramolecular distances in the planar configuration) towards intermediate values.

The *r*-space signal for $I_2$ shows a clear positive feature at shorter distances (~3–5 Å) and a negative feature at longer distances (~7–9 Å). This clear separation indicates that the molecular structure becomes more compact upon the $I_1 \rightarrow I_2$ transition, with the two phenyl rings approaching each other. The depletion of long-range interatomic distances (negative peak) and the concurrent formation of shorter distances (positive peak) together reflect the progressive folding of the molecular backbone as the CNNC dihedral angle decreases.

The *r*-space features observed in $SADS_2$ become even more pronounced in $SADS_3$. Both the positive peak at ~3.5–5 Å and the negative peak at ~7 Å significantly increase in amplitude compared to $SADS_2$, indicating that the structural difference from *trans*-AB becomes substantially larger upon the $I_2 \rightarrow$ *cis*-AB transition.

In Extended Data Fig. 5, the $SADS_1$ ($I_1$) and $SADS_2$ ($I_2$) curves are vertically scaled by factors of 8 and 4, respectively, so that all three curves can be displayed on a common scale, whereas $SADS_3$ (*cis*-AB) is shown without scaling. Their true amplitudes are therefore 8 and 4 times smaller than they appear in the figure. Taking this scaling into account, the actual signal magnitude increases progressively from $SADS_1$ to $SADS_2$ to $SADS_3$, consistent with the progressive decrease in $R_g$ along the isomerization pathway (Extended Data Table 3). The overall agreement between experiment and simulation confirms that the extracted experimental SADSs accurately represent the intrinsic structural changes of the azobenzene molecule, effectively isolated from the bulk solvent contribution.

# Supplementary Figures

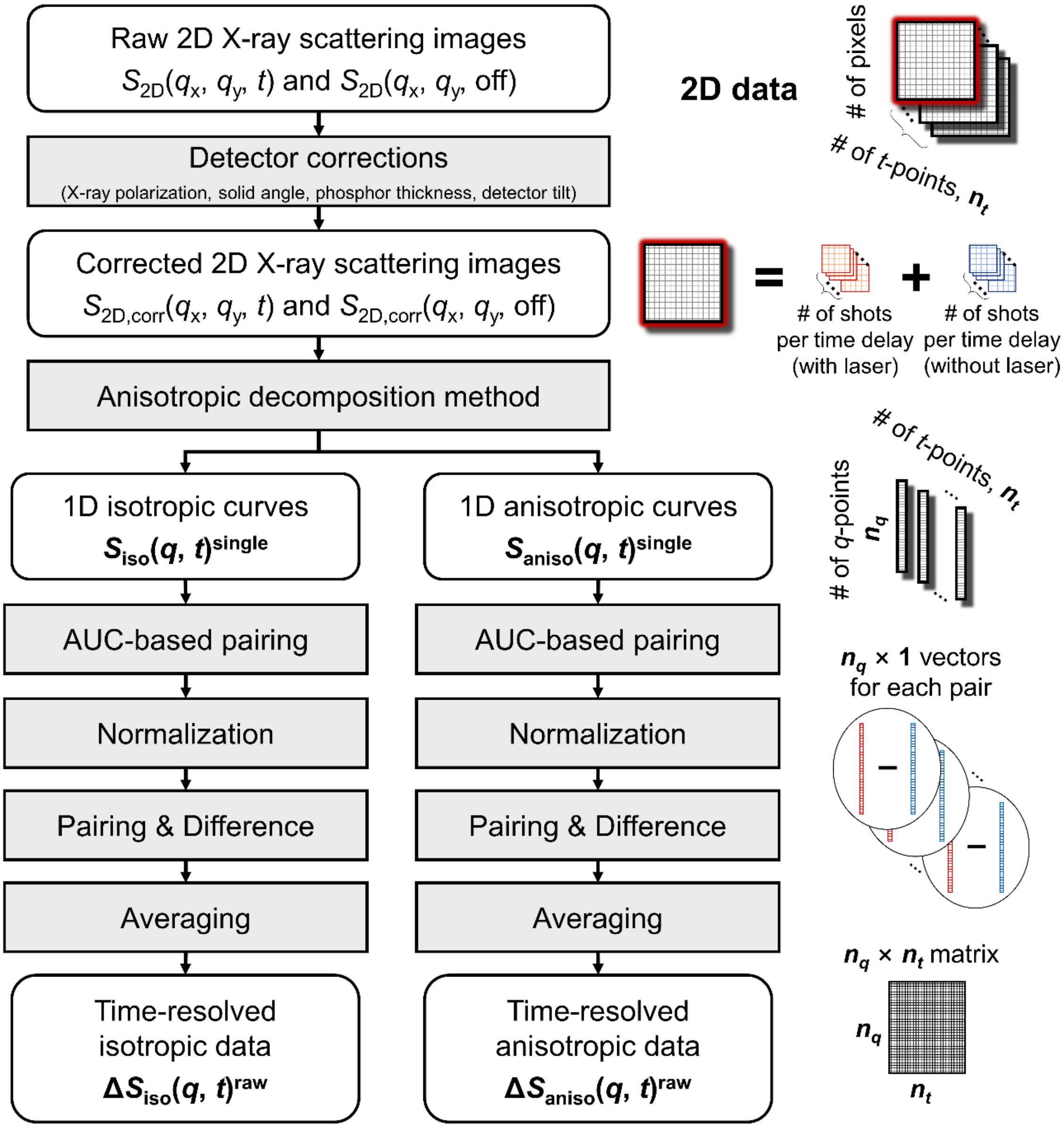


**Supplementary Fig. 1 | Data reduction pipeline for time-resolved X-ray liquidography.** The workflow illustrates the processing steps starting from the collection of raw 2D X-ray scattering images. These images undergo standard corrections (including polarization and solid angle corrections) to produce corrected 2D images, which are then decomposed into 1D isotropic and anisotropic curves using Legendre polynomial-based decomposition. The curves corresponding to "laser-on" and "laser-off" shots are matched based on an area-under-the-curve (AUC) criterion, and each curve is subsequently normalized individually. The difference curves calculated from these normalized pairs are then statistically averaged to yield the final time-resolved isotropic ($\Delta S_{iso}(q, t)^{raw}$) and anisotropic ($\Delta S_{aniso}(q, t)^{raw}$) difference scattering signals.

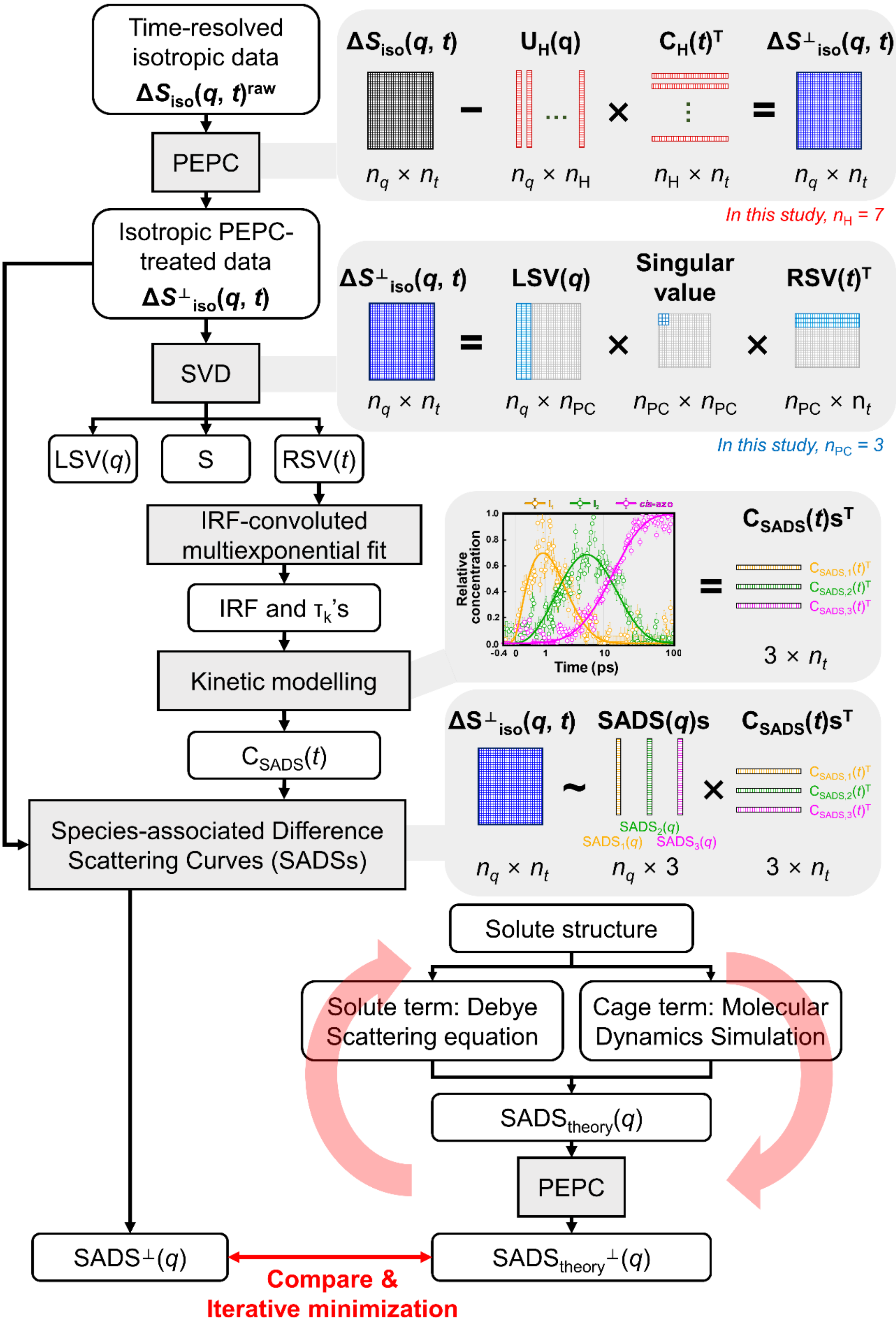


**Supplementary Fig. 2 | Data analysis pipeline for time-resolved X-ray liquidography.** The data framework encompasses both kinetic analysis and structural refinement. The difference

TRXL data are processed using the projection to extract the perpendicular component (PEPC) method. Here, the number of solvent-related principal components ($n_H$) was independently determined from the TRXL data of neat methanol, and the corresponding left singular vectors ($U_H$) were employed for the PEPC. The PEPC-treated isotropic data are then analysed via singular value decomposition (SVD) to identify the number of significant signal components ($n_{PC}$). In this study, we confirmed that $n_H = 7$ and $n_{PC} = 3$ best describe the TRXL data. Subsequently, a kinetic model is applied to extract species-associated difference scattering curves (SADSs) and their corresponding concentration profiles. Structural refinement is performed by combining a solute term derived from the Debye scattering equation and a cage term derived from MD simulations. The final structural parameters are obtained through an iterative minimization process by comparing experimental SADSs with simulated SADSs until convergence is achieved.

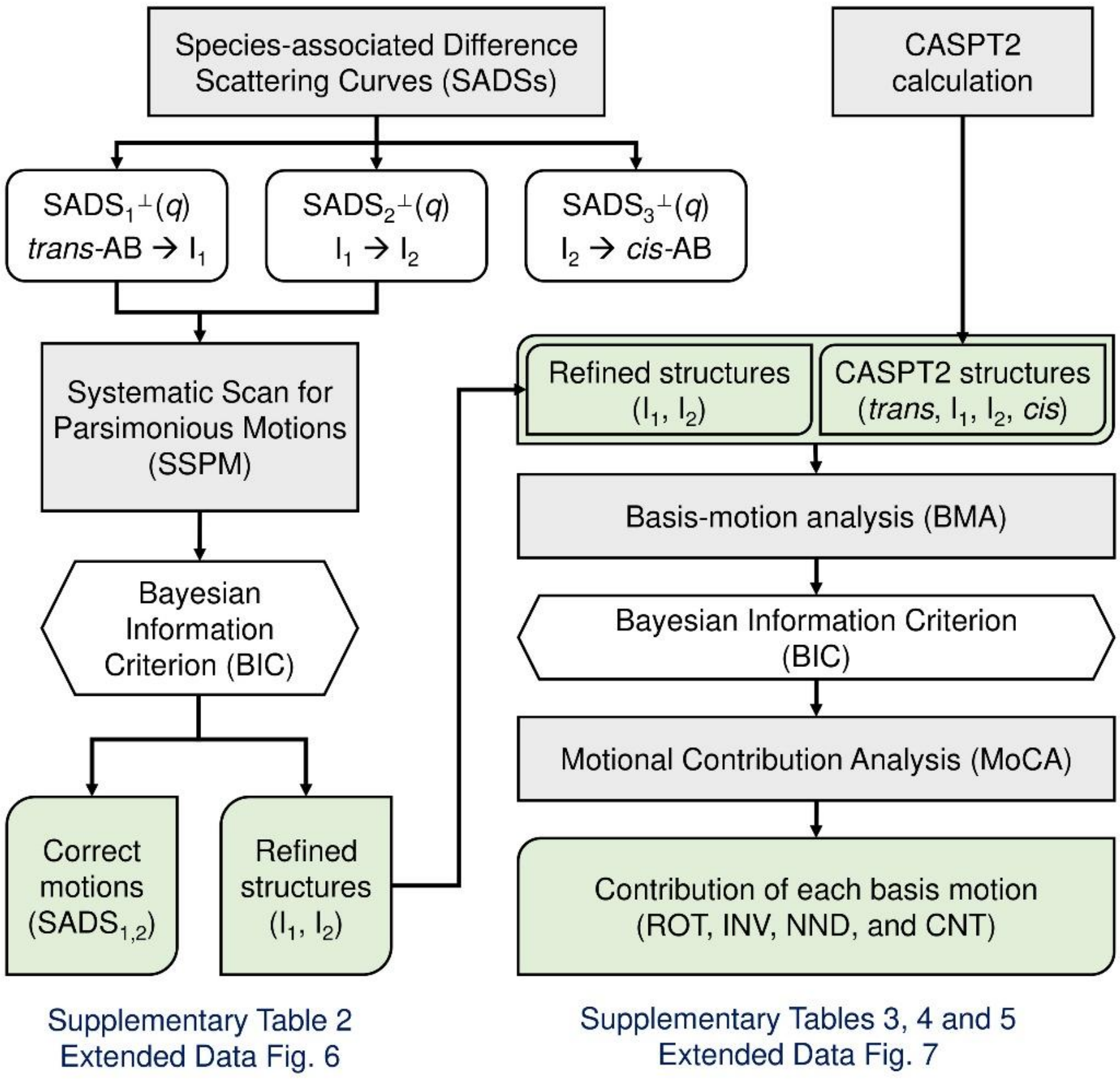


**Supplementary Fig. 3 | A schematic for structural analysis, including SSPM, BIC, BMA, and MoCA.** Workflow for extracting parsimonious structural motions from SADSs and quantifying their motional contributions. Three SADSs are assigned to sequential steps along the photoisomerization pathway (*trans*-AB to $I_1$, $I_1$ to $I_2$, and $I_2$ to *cis*-AB). A systematic scan for parsimonious motions (SSPM), evaluated using the Bayesian information criterion (BIC), identifies the minimal set of structural motions consistent with $SADS_1$ and $SADS_2$ and yields refined structures for $I_1$ and $I_2$. In parallel, reference geometries (*trans*-AB, $I_1$, $I_2$, *cis*-AB) from CASPT2 are analysed together with the refined structures using basis-motion analysis (BMA). BIC-guided model selection and motional contribution analysis (MoCA) then quantify the relative contributions of the basis motions (ROT, INV, NND, and CNT) to each structural transition. The SSPM results are summarized in Supplementary Table 2 and Extended Data Fig. 6, and the related BMA and MoCA results are reported in Supplementary Tables 3–5 and Extended Data Fig. 7.

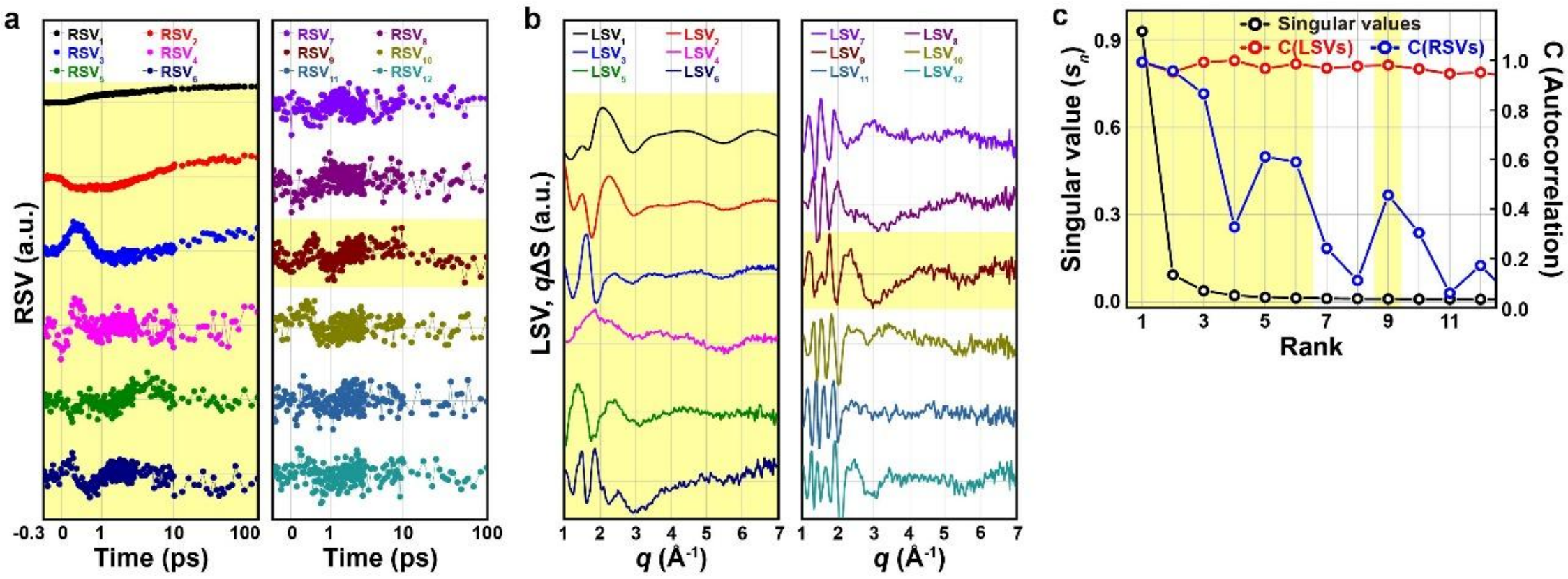

**Supplementary Fig. 4 | Results of SVD analysis of the isotropic component of the neat methanol dataset for extracting solvent reference components for PEPC treatment. a,** The top 12 right singular vectors (RSVs) representing the time-dependent contributions of the corresponding left singular vectors (LSVs), extracted from the raw isotropic difference scattering curves, $\Delta S_{\mathrm{iso}}(q, t)^{\mathrm{raw}}$, of the neat methanol. **b,** The corresponding $q$-space profiles of the 12 LSVs. **c,** Singular values (black circles, left axis), and autocorrelation values of the RSVs (blue circles, right axis) and LSVs (red circles, right axis) plotted against the component rank. The seven components highlighted with a yellow background (components 1–6 and 9) were selected to serve as the reference basis for the PEPC procedure to generate solvent-independent isotropic difference scattering signals for azobenzene. Component 1 accounts for ~90% of the variance. These seven components act as mathematical basis vectors that collectively span the isotropic solvent response, accounting for >99% of the total variance for $\Delta S_{\mathrm{iso}}(q, t)^{\mathrm{raw}}$ of the neat methanol.

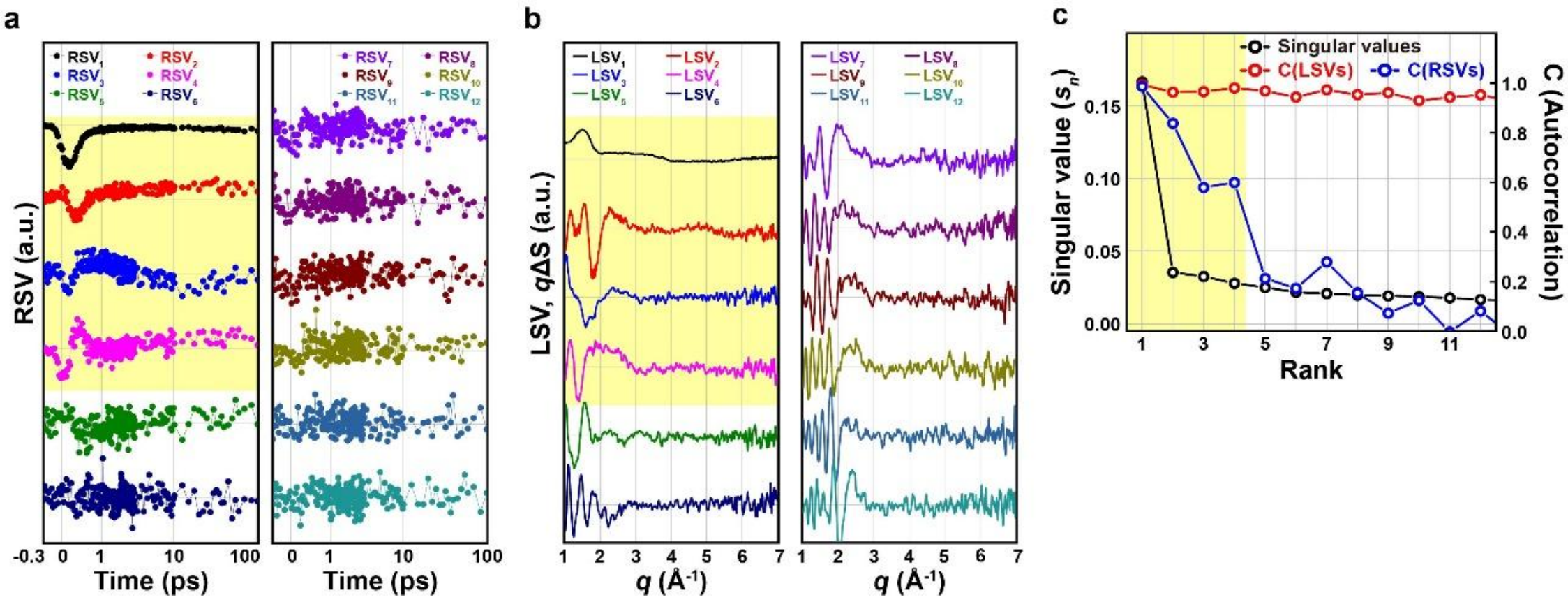


**Supplementary Fig. 5 | Results of SVD analysis of the anisotropic component of the neat methanol dataset for extracting solvent reference components for PEPC treatment. a,** The top 12 RSVs representing the time-dependent contributions of the corresponding LSVs, extracted from the raw anisotropic difference scattering curves, $\Delta S_{\mathrm{aniso}}(q,\ t)^{\mathrm{raw}}$, of the neat methanol. **b,** The corresponding *q*-space profiles of the 12 LSVs. **c,** Singular values (black circles, left axis), and autocorrelation values of the RSVs (blue circles, right axis) and LSVs (red circles, right axis) plotted against the component rank. The four components highlighted with a yellow background (components 1–4) were selected to serve as the reference basis for the PEPC procedure to generate solvent-independent anisotropic difference scattering signals for azobenzene.

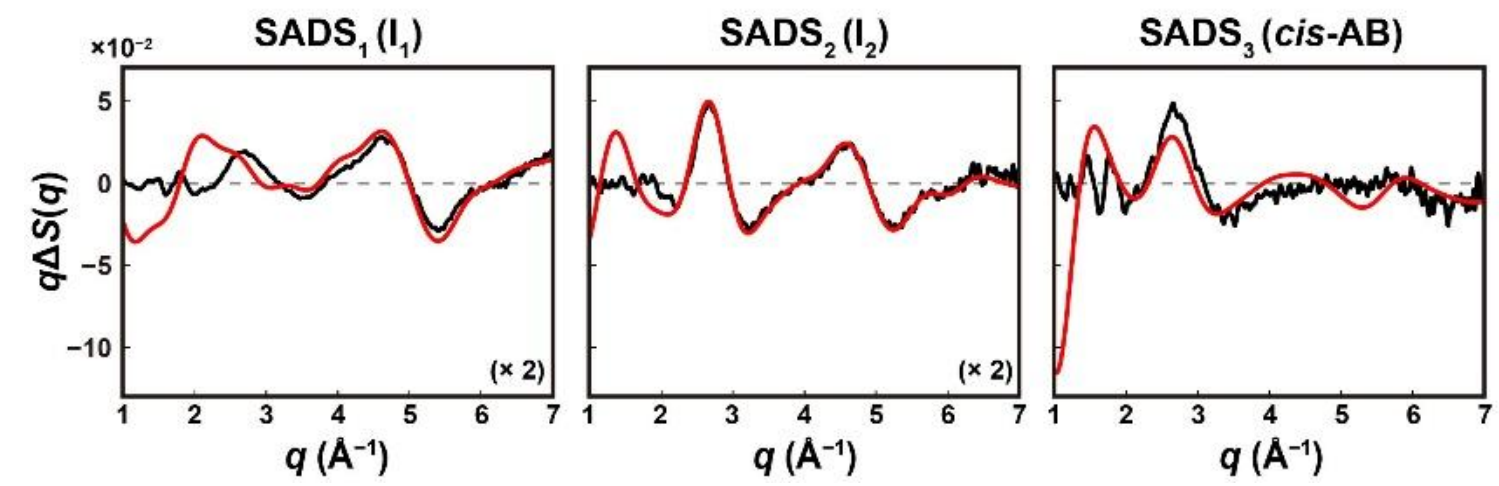


**Supplementary Fig. 6 | Comparison of theoretical difference scattering curves before and after PEPC treatment. a–c**, Comparison of the raw theoretically calculated species-associated difference scattering curves ($SADS_n'(q)$s, red lines) and the corresponding PEPC-treated theoretical curves ($SADS_n'^{\perp}(q)$s, black lines) for the refined structures: (**a**) $I_1$, (**b**) $I_2$, and (**c**) *cis*-AB. The PEPC operator projects the signal onto a subspace orthogonal to the dominant solvent scattering components, mathematically modifying the shape of the solute signal. The structural refinement minimizes the discrepancy between the experimentally extracted PEPC-treated SADS and these PEPC-projected theoretical curves, ensuring a mathematically consistent scaling within the solvent-free subspace. For clarity, selected panels are displayed with a vertical scaling factor of 2 as indicated.

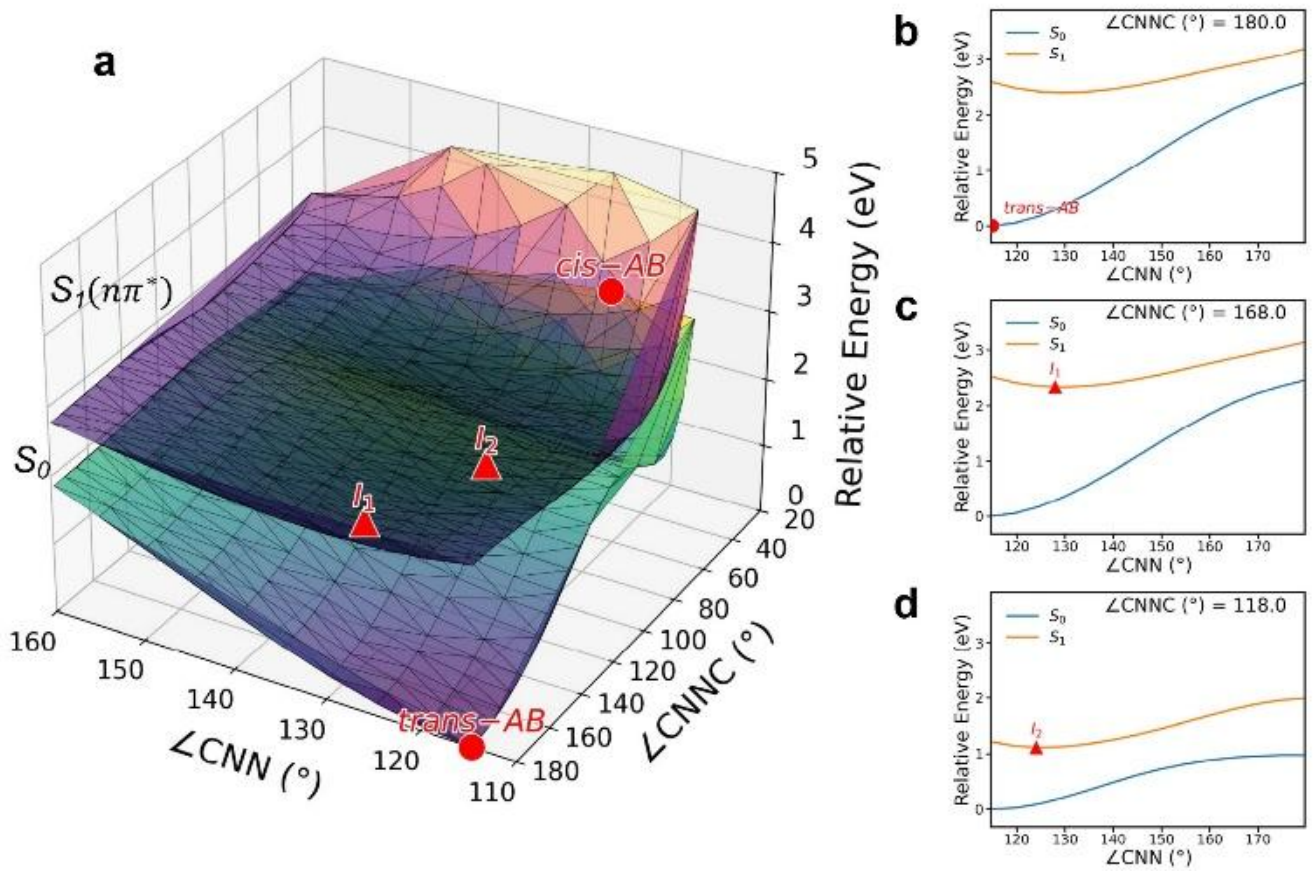


**Supplementary Fig. 7 | CASPT2 potential energy surfaces of azobenzene along CNNC and CNN angles.** Two-dimensional potential energy surfaces (PESs) of azobenzene computed at the XMS-CASPT2 level for the ground ($S_0$) and first excited ($S_1$, nπ*) electronic states. **a,** Three-dimensional side view of the PESs obtained from rigid scans along the CNNC dihedral angle and the symmetric CNN angle, with all remaining degrees of freedom kept fixed. This representation enables a direct visualization of the *trans*-AB minimum on the $S_0$ surface and of the two $S_1$ (nπ*) minima, $I_1$ and $I_2$. The *cis*-AB minimum cannot be reached within the present rigid scans, which do not allow concerted rotation of the phenyl rings; the corresponding angular region is nevertheless indicated for completeness. Red filled circles mark optimized ground-state stationary points, while red filled triangles denote the $I_1$ and $I_2$ minima on the $S_1$ surface. **b–d,** One-dimensional cuts of the two-dimensional PESs along the CNN bending angle at fixed CNNC dihedral angle, corresponding to the *trans*-AB ground-state minimum (**b**), $I_1$ (**c**), and $I_2$ (**d**) minima on the $S_1$ surface.

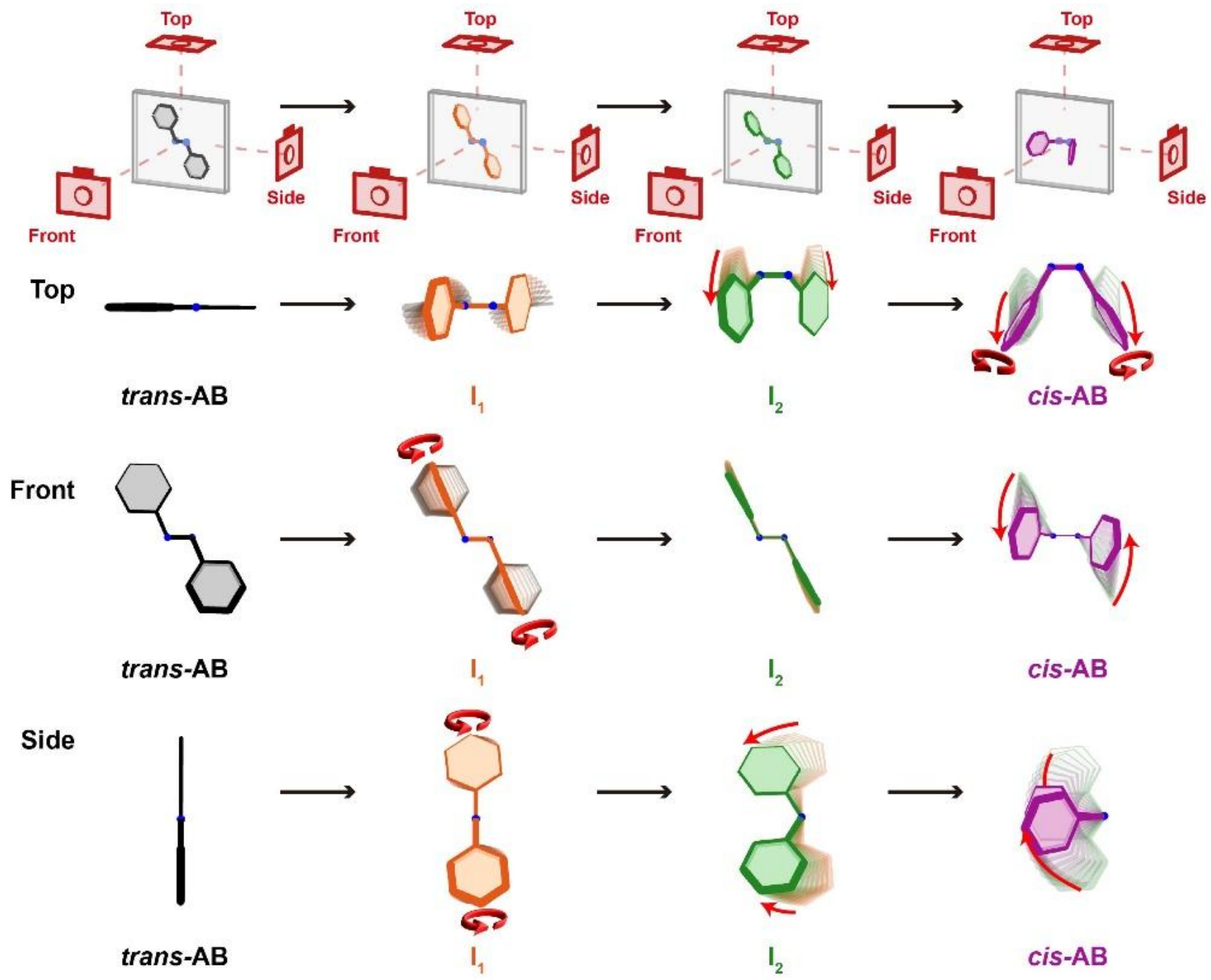


**Supplementary Fig. 8 | Three-dimensional molecular structures of *trans*-AB, $I_1$, $I_2$, and *cis*-AB obtained from TRXL, aligned in the N–N fixed frame.** This figure shows a non-volume-conserving counterpart to the volume-conserving pathway in Fig. 4b. (Top) Schematics illustrating the three viewing orientations (top, front, and side) used in this figure: top (second panel), front (third panel), and side (bottom panel). To effectively convey the structural changes, the subpanels of $I_1$, $I_2$, and *cis*-AB include the preceding structure, with the transition depicted by a sequence of interpolated geometries. Red arrows serve to highlight the direction of motion where intricate. These views visualize the sequential structural transformations: phenyl-ring torsion representing C–N torsion (CNT) from *trans*-AB to $I_1$, coupled motions dominated by N–N rotation (ROT) from $I_1$ to $I_2$ and from $I_2$ to *cis*-AB. For $I_1$, $I_2$, and *cis*-AB, each structure includes a semi-transparent overlay of its preceding species to indicate atomic displacements. Movies are provided in Supplementary Videos 3 and 4, and Supplementary Videos 5 and 6 overlay the volume-conserving and N–N fixed pathways for direct comparison.

## Supplementary Tables

**Supplementary Table 1 | Summary of key experimental parameters for fs-TRXL measurements**

| Parameter | Value |
|---|---|
| **X-ray (probe)** | |
| **Source** | PAL-XFEL |
| **Photon energy** | 15.01 ± 0.03 keV (λ = 0.83 Å) |
| **Spot size** | 20 μm × 20 μm (FWHM) |
| **Photon flux** | $1 \times 10^{12}$ (photons / pulse) |
| **Repetition rate** | 60 Hz |
| **Optical laser (pump)** | |
| **Source** | fs Ti:sapphire laser (fundamental: 800 nm) |
| **Wavelength** | 400 nm |
| **Spot size** | 55 μm × 66 μm (H × V) |
| **Pulse duration** | ~100 fs (FWHM) |
| **Laser fluence** | 1.5 mJ/mm$^2$ (*trans*-azobenzene); 5.3 mJ/mm$^2$ (neat methanol) |
| **Repetition rate** | 30 Hz |
| **Timing** | |
| **Time delays** | −0.5 ps to 100 ps (140 nominal time delays) |
| **Instrument response function** | 169 ± 5 fs (FWHM) |
| **Sample and detection** | |
| **Concentration** | 50 mM (*trans*-azobenzene in methanol) |
| **Delivery system** | 100 μm inner diameter capillary nozzle |
| **Vertical flow rate** | ~3 m/s |
| **Detector** | Rayonix MX225-HS (6×6 binning mode) |
| **Available *q*-range** | 1.0 Å$^{-1}$ to 7.0 Å$^{-1}$ |

| | |
|---|---|
| **Number of scattering images per time delay** | 18 runs × 250 laser-on images/run = 4,500 laser-on images per time delay, with an equal number (4,500) of laser-off images (*trans*-azobenzene); 4 runs × 250 laser-on images/run = 1,000 laser-on images per time delay (with an equal number of laser-off images) (neat methanol) |
| **Data retention ratio after outlier rejection** | 95–99% |

**Supplementary Table 2 | Structural motions and their combinations evaluated in the SSPM analysis**.

For each combination, the table lists the fitting parameters, the number of active motion parameters ($k$), and the resulting $\chi^2$ and $\Delta$BIC($\chi^2$) values. The $\chi^2$ and $\Delta$BIC($\chi^2$) values correspond to the SSPM analysis presented in Extended Data Fig. 6, where various combinations of structural motions are evaluated to determine the simplest structural models consistent with the experimental data. For each structural transition indicated by the arrow (→, indicating a transition from the initial structure to the target structure), the $\chi^2$ and $\Delta$BIC($\chi^2$) values corresponding to the best structural model are highlighted in bold.

| Structural motions | Fitting parameters* | SADS1(q): *trans*-AB → $I_1$ | | | SADS$_2$($q$): $I_1$ → $I_2$ | | |
|---|---|---|---|---|---|---|---|
| | | $k^{\#}$ | $\chi^2$ | $\Delta$BIC($\chi^2$) | $k^{\#}$ | $\chi^2$ | $\Delta$BIC($\chi^2$) |
| ROT | ∠CNNC (°) | 1 | 232.7 | 109.7 | 1 | 208.8 | 30.9 |
| INV | ∠CNN$_1$ (°), ∠CNN$_2$ (°) | 1 | 198.0 | 74.9 | 2 | 284.4 | 111.2 |
| NND | $r_{NN}$ (Å) | 1 | 228.1 | 105.0 | 1 | 345.0 | 167.1 |
| CNT | ∠CCNN$_1$ (°), ∠CCNN$_2$ (°) | **1** | **123.1**† | **0.0**† | 2 | 369.9 | 196.7 |
| ROT + INV | ∠CNNC (°), ∠CNN$_1$ (°), ∠CNN$_2$ (°) | 2 | 198.0 | 79.6 | 3 | 192.0 | 23.5 |
| ROT + NND | ∠CNNC (°), $r_{NN}$ (Å) | 2 | 228.1 | 109.7 | 2 | 200.2 | 26.9 |
| ROT + CNT | ∠CNNC (°), ∠CCNN$_1$ (°), ∠CCNN$_2$ (°) | 2 | 123.1 | 4.7 | 3 | **168.5**† | **0.0**† |
| INV + NND | ∠CNN$_1$ (°), ∠CNN$_2$ (°), $r_{NN}$ (Å) | 2 | 192.5 | 74.2 | 3 | 283.3 | 114.8 |
| INV + CNT | ∠CNN$_1$ (°), ∠CNN$_2$ (°), ∠CCNN$_1$ (°), ∠CCNN$_2$ (°) | 2 | 123.1 | 4.7 | 4 | 188.9 | 25.1 |
| NND + CNT | $r_{NN}$ (Å), ∠CCNN$_1$ (°), ∠CCNN$_2$ (°) | 2 | 122.1 | 3.8 | 3 | 337.8 | 169.3 |

| | | | | | | | |
|---|---|---|---|---|---|---|---|
| ROT + INV + NND | ∠CNNC (°), ∠$CNN_1$ (°), ∠$CNN_2$ (°), $r_{NN}$ (Å) | 3 | 192.5 | 78.9 | 4 | 190.7 | 26.8 |
| ROT + INV + CNT | ∠CNNC (°), ∠$CNN_1$ (°), ∠$CNN_2$ (°), ∠$CCNN_1$ (°), ∠$CCNN_2$ (°) | 3 | 123.1 | 9.4 | 5 | 168.0 | 8.8 |
| ROT + NND + CNT | ∠CNNC (°), $r_{NN}$ (Å), ∠$CCNN_1$ (°), ∠$CCNN_2$ (°) | 3 | 122.1 | 8.5 | 4 | 168.2 | 4.4 |
| INV + NND + CNT | ∠$CNN_1$ (°), ∠$CNN_2$ (°), $r_{NN}$ (Å), ∠$CCNN_1$ (°), ∠$CCNN_2$ (°) | 3 | 122.1 | 8.5 | 5 | 188.1 | 29.0 |
| ROT + INV + NND + CNT | ∠CNNC (°), ∠$CNN_1$ (°), ∠$CNN_2$ (°), $r_{NN}$ (Å), ∠$CCNN_1$ (°), ∠$CCNN_2$ (°) | 4 | 122.1 | 13.2 | 6 | 166.0 | 11.6 |

* Fitting parameter definitions:

∠CNNC (°): The CNNC dihedral angle describing torsional rotation around the N–N bond, used to parameterize the rotational (ROT) motion of the azo group.

∠$CNN_1$ and ∠$CNN_2$ (°): The C–N–N bond angles at the two phenyl-azo junctions, independently describing inversion (INV) motions on each side of the azo group.

$r_{NN}$ (Å): The N–N bond length, used to represent the N–N bond stretching motion (NND).

∠$CCNN_1$ and ∠$CCNN_2$ (°): The dihedral angles describing the relative twist of each phenyl ring with respect to the CNN plane about the respective C–N bond, used to parameterize the CNT motion.

$\sigma^2$ (Å$^2$): The mean-squared displacement parameter introduced through a Debye–Waller factor to account for thermal broadening of interatomic distances in the calculated scattering curves.

$c$: The scaling coefficient, which relates the experimentally observed SADS amplitude to the unit-concentration simulated SADS (see Section 12.3). For $SADS_1$, $c = \alpha$; for $SADS_2$, $c = \alpha \cdot \beta$.

$a_n$, $b_n$: The coefficients of the linear baseline term $B_n(q) = a_n + b_n / q$ (Supplementary Method 3).

$k^{\#}$ denotes the number of active motion parameters. Every model additionally includes four parameters common to all models ($\sigma^2$, the scaling coefficient $c$, and the two baseline coefficients $a_n$ and $b_n$), which are omitted from the Fitting parameters column for clarity; the total number of fitting parameters is therefore $k + 4$. For the $SADS_1$ analysis, $C_i$ symmetry was imposed, constraining $\angle CNN_1 = \angle CNN_2$ and $\angle CCNN_1 = -\angle CCNN_2$, so that INV and CNT each contribute one free parameter rather than two. For the $SADS_2$ analysis, no symmetry constraint was imposed, and all parameters were varied independently. The BIC was computed as $BIC(\chi^2) = \chi^2 + (k + 4) \cdot \ln(N_{eff})$, where $N_{eff} = 110$ is the effective number of independent data points determined by polynomial-order optimization via BIC minimization (see Supplementary Method 13).

[†]The best model for each transition was selected based on the minimum $BIC(\chi^2)$. For the *trans*-AB → $I_1$ transition, CNT ($k = 1$, $BIC(\chi^2) = 146.6$) is the best model; the next-ranked model, NND + CNT ($k = 2$, $BIC(\chi^2) = 150.3$, $\Delta BIC(\chi^2) = 3.8$), does not achieve a statistically significant improvement, confirming that the CNT motion alone is sufficient to describe this transition. For the $I_1 \rightarrow I_2$ transition, ROT + CNT ($k = 3$, $BIC(\chi^2) = 201.4$) yields the lowest $BIC(\chi^2)$; the next-ranked model, ROT + NND + CNT ($k = 4$, $BIC(\chi^2) = 205.8$, $\Delta BIC(\chi^2) = 4.4$), does not achieve a statistically significant improvement, and ROT + INV + CNT (k = 5, $BIC(\chi^2) = 210.3$, $\Delta BIC(\chi^2) = 8.8$) confirms that INV does not provide a statistically justified improvement. ROT ($k = 1$, $BIC(\chi^2) = 232.3$, $\Delta BIC(\chi^2) = 30.9$) is substantially worse, confirming that CNT is a necessary component for this transition.

**Supplementary Table 3 | RMSD and ΔBIC(RMSD) values from BMA analysis for TRXL-refined structures.** All 15 possible combinations of four structural motions (ROT, INV, NND, CNT) were optimized to reproduce each structural transition between TRXL-refined structures. The best model for each transition, identified by the minimum BIC(RMSD) (ΔBIC(RMSD) = 0), is indicated in bold.

| **Structural motions** | ***trans*-AB → $I_1$** | | **$I_1$ → $I_2$** | | **$I_2$ → *cis*-AB** | |
|---|---|---|---|---|---|---|
| | **RMSD (Å)** | **ΔBIC(RMSD)*** | **RMSD (Å)** | **ΔBIC(RMSD)*** | **RMSD (Å)** | **ΔBIC(RMSD)*** |
| ROT | 0.3134 | 1154.9 | 0.3752 | 1176.6 | 0.2862 | 308.2 |
| INV | 0.3107 | 1158.0 | 0.8904 | 1305.3 | 0.5873 | 415.9 |
| NND | 0.2867 | 1142.1 | 0.9040 | 1303.2 | 1.5229 | 548.9 |
| CNT | **< 0.0001**[†] | **0.0**[†] | 0.7633 | 1283.1 | 0.9451 | 484.4 |
| ROT + INV | 0.3107 | 1162.2 | 0.2098 | 1101.4 | 0.0438 | 46.3 |
| ROT + NND | 0.2867 | 1146.4 | 0.3509 | 1171.2 | 0.2013 | 261.7 |
| ROT + CNT | < 0.0001 | 4.3 | **< 0.0001**[†] | **0.0**[†] | 0.1423 | 216.1 |
| INV + NND | 0.2536 | 1133.0 | 0.8894 | 1309.4 | 0.5580 | 412.8 |
| INV + CNT | < 0.0001 | 8.6 | 0.3395 | 1175.0 | 0.1938 | 264.8 |
| NND + CNT | < 0.0001 | 4.3 | 0.7389 | 1282.7 | 0.7069 | 446.9 |
| ROT + INV + NND | 0.2536 | 1137.3 | 0.2089 | 1105.0 | 0.0438 | 50.5 |
| ROT + INV + CNT | < 0.0001 | 12.8 | < 0.0001 | 8.6 | **0.0299**[†] | **0.0**[†] |
| ROT + NND + CNT | < 0.0001 | 8.6 | < 0.0001 | 4.3 | 0.1416 | 219.6 |
| INV + NND + CNT | < 0.0001 | 12.8 | 0.2405 | 1129.6 | 0.1781 | 257.0 |
| ROT + INV + NND + CNT | < 0.0001 | 17.1 | < 0.0001 | 12.8 | 0.0299 | 4.1 |

* ΔBIC(RMSD) = BIC(RMSD) − $BIC(RMSD)_{min}$. $BIC(RMSD)_{min}$ values: −1396.8 (*trans*-AB → $I_1$), −1392.6 ($I_1$ → $I_2$), −563.1 ($I_2$ → *cis*-AB).

$\mathrm{BIC(RMSD)} = n \cdot \ln(\mathrm{RMSD}^2 / 3) + k \cdot \ln(n)$, where $n = 72$ ($3 \times 24$ atoms), $k$ = number of free parameters.

RMSD values below $10^{-4}$ Å are reported as < 0.0001.

† Minimum BIC ($\Delta$BIC(RMSD) = 0). For the *trans*-AB → $I_1$ and $I_1$ → $I_2$ transitions, the target structures were determined by the SSPM fitting procedure, in which the structural parameters of specific motion combinations were optimized against the experimental SADSs. Consequently, BMA is expected to recover the same motion set with RMSD < 0.0001 Å, and the results serve as an internal consistency check confirming that the BMA decomposition correctly identifies the motions used in the SSPM refinement. For the $I_2$ → *cis*-AB transition, the target *cis*-AB structure was obtained by a structure refinement starting from the CASPT2-calculated *cis*-AB geometry, rather than by applying motions to the $I_2$ structure. Since the *cis*-AB structure was not constructed by sequential application of basis motions from $I_2$, BMA cannot be expected to achieve RMSD < 0.0001 Å, unlike the preceding two transitions where the target structures were generated directly by motion application to the initial structure. ROT + INV + CNT ($k = 5$, BIC(RMSD) = −563.1) achieves the lowest BIC with RMSD = 0.0299 Å. The next-ranked model, ROT + INV + NND + CNT ($k = 6$, RMSD = 0.0299 Å, $\Delta$BIC(RMSD) = 4.1), falls in the range of considerably less support ($2 < \Delta\mathrm{BIC} < 6$).

**Supplementary Table 4 | RMSD and ΔBIC(RMSD) values from BMA analysis for CASPT2-calculated structures.** All 15 possible combinations of four structural motions were optimized to reproduce each structural transition between CASPT2-calculated geometries. The best model for each transition, identified by the minimum BIC(RMSD) (ΔBIC(RMSD) = 0), is indicated in bold.

| **Structural motions** | ***trans*-AB → $I_1$** | | **$I_1$ → $I_2$** | | **$I_2$ → *cis*-AB** | |
|---|---|---|---|---|---|---|
| | **RMSD (Å)** | **ΔBIC(RMSD)*** | **RMSD (Å)** | **ΔBIC(RMSD)*** | **RMSD (Å)** | **ΔBIC(RMSD)*** |
| ROT | 0.0691 | 107.0 | 0.0308 | 186.5 | 0.6108 | 436.5 |
| INV | 0.1384 | 211.3 | 0.6595 | 632.1 | 0.2736 | 325.1 |
| NND | 0.1415 | 210.2 | 0.7046 | 637.3 | 0.9784 | 504.3 |
| CNT | 0.1230 | 194.2 | 0.5382 | 602.8 | 1.1009 | 525.6 |
| ROT + INV | 0.0356 | 19.9 | 0.0132 | 73.1 | 0.2443 | 313.1 |
| ROT + NND | 0.0569 | 83.1 | 0.0291 | 182.5 | 0.4066 | 382.1 |
| ROT + CNT | 0.0612 | 98.1 | 0.0220 | 147.0 | 0.0490 | 81.7 |
| INV + NND | 0.1377 | 214.8 | 0.6592 | 636.3 | 0.2588 | 321.3 |
| INV + CNT | 0.1019 | 175.7 | 0.2270 | 487.0 | 0.2041 | 291.4 |
| NND + CNT | 0.1178 | 192.3 | 0.5173 | 601.4 | 0.8302 | 489.2 |
| ROT + INV + NND | 0.0337 | 16.2 | 0.0124 | 68.4 | 0.2279 | 307.3 |
| ROT + INV + CNT | 0.0313 | 10.2 | 0.0101 | 42.7 | **0.0262**† | **0.0**† |
| ROT + NND + CNT | 0.0483 | 68.2 | 0.0219 | 150.2 | 0.0385 | 51.4 |
| INV + NND + CNT | 0.0921 | 165.5 | 0.1997 | 472.8 | 0.1897 | 285.2 |
| ROT + INV + NND + CNT | **0.0283**† | **0.0**† | **0.0073**† | **0.0**† | 0.0262 | 4.3 |

* ΔBIC(RMSD) = BIC(RMSD) − $BIC(RMSD)_{min}$. $BIC(RMSD)_{min}$ values: −566.6 (*trans*-AB → $I_1$), −762.6 ($I_1$ → $I_2$), −582.3 ($I_2$ → *cis*-AB).

$BIC(RMSD) = n \cdot \ln(RMSD^2 / 3) + k \cdot \ln(n)$, where $n = 72$ ($3 \times 24$ atoms), $k$ = number of free parameters.

RMSD values below $10^{-4}$ Å are reported as < 0.0001.
† Minimum BIC ($\Delta BIC(RMSD) = 0$).

**Supplementary Table 5 | MoCA results for the best models of the *trans*-AB → $I_1$, $I_1$ → $I_2$, and $I_2$ → *cis*-AB transitions.** MoCA quantifies the relative contribution of each motion using leave-one-out (LOO) analysis. The percentage listed for each motion is the increase in RMSD caused by removing that motion from the best model, with the remaining motions re-optimized, expressed as a fraction of the sum of these increases over all motions in the model. The percentages for a given transition therefore sum to 100% (Supplementary Method 15, Eq. S37). Results are shown for both TRXL-refined and CASPT2-calculated structural transitions for comparison. RMSD values from BMA are also listed. Dominant motions are indicated in bold and italic.

| | **RMSD (Å)** | ***Motional contributions*** |
|---|---|---|
| ***trans*-AB → $I_1$ (TRXL-refined)** | < 0.0001 | ***CNT*** (100%) |
| **$I_1$ → $I_2$ (TRXL-refined)** | < 0.0001 | ***ROT*** (67%) + CNT (33%) |
| **$I_2$ → *cis*-AB (TRXL-refined)** | 0.0299 | ***ROT*** (56%) + ***INV*** (39%) + CNT (5%) |
| ***trans*-AB → $I_1$ (CASPT2-calculated)** | 0.0283 | ***ROT*** (69%) + INV (22%) + NND (3%) + CNT (6%) |
| **$I_1$ → $I_2$ (CASPT2-calculated)** | 0.0073 | ***ROT*** (90%) + INV (7%) + NND (1%) + CNT (2%) |
| **$I_2$ → *cis*-AB (CASPT2-calculated)** | 0.0262 | ***ROT*** (43%) + INV (5%) + ***CNT*** (52%) |

**Supplementary Table 6 | Dependence of the SSPM model selection on $N_{eff}$.** For each transition, all 15 candidate motion combinations were ranked by the $\chi^2$-based BIC (Eq. S31) at each value of $N_{eff}$. At every value listed, the reported model, CNT for *trans*-AB → $I_1$ and ROT + CNT for $I_1$ → $I_2$, had the lowest BIC of the 15, showing that the model selection is insensitive to the choice of $N_{eff}$ over the entire range examined. ΔBIC is the margin by which the reported model is the lowest: the BIC of the model with the second-lowest BIC minus the BIC of the reported model. The model with the second-lowest BIC is NND + CNT for *trans*-AB → $I_1$ and ROT + NND + CNT for $I_1$ → $I_2$ at every $N_{eff}$ value listed. The "basis" column states how each value of $N_{eff}$ was obtained: from a geometric (Shannon) estimate with the indicated $D_{max}$; from the Chebyshev determination of Supplementary Method 13 under the indicated weighting; or from the number of measured $q$-points, the largest value $N_{eff}$ can take.

| $N_{eff}$ | basis | ΔBIC, *trans*-AB → $I_1$ | ΔBIC, $I_1$ → $I_2$ |
|---|---|---|---|
| 23 | geometric, solute only ($D_{max}$ = 12 Å) | 2.21 | 2.84 |
| 29 | geometric, solute only (15 Å) | 2.45 | 3.08 |
| 57 | geometric, with the solvation shell (30 Å) | 3.12 | 3.75 |
| 91 | Chebyshev, ten curves equally weighted | 3.59 | 4.22 |
| 110 | Chebyshev, ten curves unweighted (adopted) | 3.78 | 4.41 |
| 115 | geometric, with the solvation shell (60 Å) | 3.82 | 4.45 |
| 183 | Chebyshev, the three SADSs alone | 4.29 | 4.92 |
| 511 | number of $q$-points (upper bound) | 5.32 | 5.94 |

**Supplementary Table 7 | Solvent swept volume for each structural transition.** Swept volumes (Å$^3$) were estimated using a grid-based approach (grid spacing 0.15 Å, Bondi van der Waals radii) as described in Supplementary Note 11. Two reference frames are compared: (i) N–N fixed, in which the nitrogen atoms are held stationary and both phenyl rings share each internal coordinate change equally ($C_2$ symmetry), and (ii) minimum displacement, obtained by all-atom Kabsch superposition. The static molecular volume is approximately 175 Å$^3$.

| Transition | Frame | Swept volume (Å$^3$) | Reduction |
|---|---|---|---|
| ***trans*-AB → $I_1$** | N–N fixed | 104 | |
| | Minimum displacement | 20 | 81% |
| **$I_1$ → $I_2$** | N–N fixed | 65 | |
| | Minimum displacement | 37 | 44% |
| **$I_2$ → *cis*-AB** | N–N fixed | 115 | |
| | Minimum displacement | 114 | ~0.5% |

**Supplementary Table 8 | RMSD between the initial and final structures of each transition.** RMSD values (Å) were computed for two reference frames: (i) N–N fixed, in which the nitrogen atoms are held stationary and $C_2$ symmetry is imposed so that both phenyl rings contribute equally to the rearrangement (as in Supplementary Table 7), and (ii) minimum displacement, obtained by all-atom Kabsch superposition. The N–N fixed RMSD reflects the magnitude of phenyl-ring displacement when the azo bridge is stationary, while the minimum-displacement-frame RMSD represents the minimum RMSD achievable through rigid-body superposition. Structures used: *trans*-AB (CASPT2-calculated), $I_1$, $I_2$, and *cis*-AB (TRXL-refined); all 24 atoms including hydrogen were included.

| Transition | Frame | RMSD (Å) |
|---|---|---|
| ***trans*-AB → $I_1$** | N–N fixed | 1.98 |
| | Minimum displacement | 0.40 |
| **$I_1$ → $I_2$** | N–N fixed | 1.78 |
| | Minimum displacement | 1.10 |
| **$I_2$ → *cis*-AB** | N–N fixed | 3.01 |
| | Minimum displacement | 2.31 |

# References


1 Rajagopal, S., Schmidt, M., Anderson, S., Ihee, H. & Moffat, K. Analysis of experimental time-resolved crystallographic data by singular value decomposition. *Acta Crystallogr. D: Struct. Biol.* **60**, 860-871 (2004).

2 Satzger, H. *et al.* Fluorescence spectra of trans- and cis-azobenzene–emission from the Franck–Condon state. *Chem. Phys. Lett.* **372**, 216-223 (2003).

3 Chang, C.-W., Lu, Y.-C., Wang, T.-T. & Diau, E. W.-G. Photoisomerization dynamics of azobenzene in solution with S1 excitation: a femtosecond fluorescence anisotropy study. *J. Am. Chem. Soc.* **126**, 10109-10118 (2004).

4 Quick, M. *et al.* Photoisomerization dynamics and pathways of trans- and cis-azobenzene in solution from broadband femtosecond spectroscopies and calculations. *J. Phys. Chem. B* **118**, 8756-8771 (2014).

5 Lee, Y. *et al.* Cerium photocatalyst in action: structural dynamics in the presence of substrate visualized via time-resolved x-ray liquidography. *J. Am. Chem. Soc.* **145**, 23715-23726 (2023).

6 Ki, H., Gu, J., Cha, Y., Lee, K. W. & Ihee, H. Projection to extract the perpendicular component (PEPC) method for extracting kinetics from time-resolved data. *Struct. Dyn.* **10**, 034103 (2023).

7 Kim, D. *et al.* Excited-State Structural Dynamics of the Cubane-Type Metal Cluster [Cu4I4(py)4] Explored by Time-Resolved X-Ray Liquidography. *Adv. Sci.* **12**, 2414970 (2025).

8 Venzon, D. & Moolgavkar, S. A method for computing profile-likelihood-based confidence intervals. *J. R. Stat. Soc. Ser. C Appl. Stat.* **37**, 87-94 (1988).

9 Raue, A. *et al.* Structural and practical identifiability analysis of partially observed dynamical models by exploiting the profile likelihood. *Bioinformatics* **25**, 1923-1929 (2009).

10 Press, W. H., Teukolsky, S. A., Vetterling, W. T. & Flannery, B. P. *Numerical Recipes: The Art of Scientific Computing*. 3 edn, Section 15.6 (Cambridge University Press, 2007).

11 Biasin, E. *et al.* Femtosecond x-ray scattering study of ultrafast photoinduced structural dynamics in solvated [Co(terpy)2]2+. *Phys. Rev. Lett.* **117**, 013002 (2016).

12 Biasin, E. *et al.* Anisotropy enhanced X-ray scattering from solvated transition metal complexes. *J. Synchrotron Rad.* **25**, 306-315 (2018).

13 Kim, J. G. *et al.* Mapping the emergence of molecular vibrations mediating bond formation. *Nature* **582**, 520-524 (2020).

14 Choi, E. H. *et al.* Filming ultrafast roaming-mediated isomerization of bismuth triiodide in solution. *Nat. Commun.* **12**, 4732 (2021).

15 Ki, H. *et al.* Optical Kerr effect of liquid acetonitrile probed by femtosecond time-resolved x-ray liquidography. *J. Am. Chem. Soc.* **143**, 14261-14273 (2021).

16 Heo, J. *et al.* Determining the charge distribution and the direction of bond cleavage with femtosecond anisotropic x-ray liquidography. *Nat. Commun.* **13**, 522 (2022).

17 Ki, H. *et al.* Photoactivation of triosmium dodecacarbonyl at 400 nm probed with time-resolved X-ray liquidography. *Chem. Commun.* **58**, 7380-7383 (2022).

18 Nimmrich, A. *et al.* Solvent-dependent structural dynamics in the ultrafast photodissociation reaction of triiodide observed with time-resolved x-ray solution scattering. *J. Am. Chem. Soc.* **145**, 15754-15765 (2023).

19 Mara, M. W. *et al.* Characterizing Ultrafast Intersystem Crossing Pathways in Molecular Pt Dimers Using Time-Resolved Wide-Angle X-ray Scattering. *J. Phys. Chem. Lett.* **16**, 7120-7126 (2025).

20 Neese, F. The ORCA program system. *WIREs Comput. Mol. Sci.* **2**, 73-78 (2012).

21 Neese, F. Software update: the ORCA program system—version 6.0. *WIREs Comput. Mol. Sci.* **15**, e70019 (2025).

22 Marenich, A. V., Cramer, C. J. & Truhlar, D. G. Universal solvation model based on solute electron density and on a continuum model of the solvent defined by the bulk dielectric constant and atomic surface tensions. *J. Phys. Chem. B* **113**, 6378-6396 (2009).

23 Plett, C., Stahn, M., Bursch, M., Mewes, J.-M. & Grimme, S. Improving quantum chemical solvation models by dynamic radii adjustment for continuum solvation (draco). *J. Phys. Chem. Lett.* **15**, 2462-2469 (2024).

24 Nikolaienko, T. Y., Bulavin, L. A. & Hovorun, D. M. JANPA: An open source cross-platform implementation of the Natural Population Analysis on the Java platform. *Comput. Theor. Chem.* **1050**, 15-22 (2014).

25 Nikolaienko, T. Y. & Bulavin, L. A. Localized orbitals for optimal decomposition of molecular properties. *Int. J. Quantum Chem.* **119**, e25798 (2019).

26 Kim, T. K., Lee, J. H., Wulff, M., Kong, Q. & Ihee, H. Spatiotemporal Kinetics in Solution Studied by Time-Resolved X-Ray Liquidography (Solution Scattering).

*ChemPhysChem* **10**, 1958-1980 (2009).

27 Kim, J., Kim, J. G., Ki, H., Ahn, C. W. & Ihee, H. Estimating signal and noise of time-resolved X-ray solution scattering data at synchrotrons and XFELs. *J. Synchrotron Rad.* **27**, 633-645 (2020).

28 Bekker, H. *et al.* Gromacs-a parallel computer for molecular-dynamics simulations in *4th international conference on computational physics (PC 92).* 252-256 (1993).

29 Van Der Spoel, D. *et al.* GROMACS: fast, flexible, and free. *J. Comput. Chem.* **26**, 1701-1718 (2005).

30 Abraham, M. J. *et al.* GROMACS: High performance molecular simulations through multi-level parallelism from laptops to supercomputers. *SoftwareX* **1**, 19-25 (2015).

31 Abraham, M. *et al.* GROMACS 2024 Manual, Zenodo, https://zenodo.org/records/14016613 (2024).

32 Jorgensen, W. L. Optimized intermolecular potential functions for liquid alcohols. *J. Phys. Chem.* **90**, 1276-1284 (1986).

33 Jorgensen, W. L., Maxwell, D. S. & Tirado-Rives, J. Development and testing of the OPLS all-atom force field on conformational energetics and properties of organic liquids. *J. Am. Chem. Soc.* **118**, 11225-11236 (1996).

34 Bussi, G., Donadio, D. & Parrinello, M. Canonical sampling through velocity rescaling. *J. Chem. Phys.* **126**, 014101 (2007).

35 Bernetti, M. & Bussi, G. Pressure control using stochastic cell rescaling. *J. Chem. Phys.* **153**, 114107 (2020).

36 Hess, B., Bekker, H., Berendsen, H. J. C. & Fraaije, J. G. E. M. LINCS: A linear constraint solver for molecular simulations. *J. Comput. Chem.* **18**, 1463-1472 (1997).

37 Darden, T., York, D. & Pedersen, L. Particle mesh Ewald: An N·log(N) method for Ewald sums in large systems. *J. Chem. Phys.* **98**, 10089-10092 (1993).

38 Ihee, H. *et al.* Ultrafast x-ray diffraction of transient molecular structures in solution. *Science* **309**, 1223-1227 (2005).

39 Cammarata, M. *et al.* Tracking the structural dynamics of proteins in solution using time-resolved wide-angle X-ray scattering. *Nat. Methods* **5**, 881-886 (2008).

40 Dohn, A. O. *et al.* On the calculation of x-ray scattering signals from pairwise radial distribution functions. *J. Phys. B: At. Mol. Opt. Phys.* **48**, 244010 (2015).

41 Kim, H. *et al.* Ultrafast structural dynamics of in-cage isomerization of diiodomethane in solution. *Chem. Sci.* **12**, 2114-2120 (2021).

42 MacKay, D. J. Bayesian interpolation. *Neural Comput.* **4**, 415-447 (1992).

43 Kass, R. E. & Raftery, A. E. Bayes factors. *J. Am. Stat. Assoc.* **90**, 773-795 (1995).

44 Budzak, S., Scalmani, G. & Jacquemin, D. Accurate excited-state geometries: A CASPT2 and coupled-cluster reference database for small molecules. *J. Chem. Theory Comput.* **13**, 6237-6252 (2017).

45 Page, C. S. & Olivucci, M. Ground and excited state CASPT2 geometry optimizations of small organic molecules. *J. Comput. Chem.* **24**, 298-309 (2003).

46 Kim, K. H. *et al.* Direct observation of bond formation in solution with femtosecond X-ray scattering. *Nature* **518**, 385-389 (2015).

47 Struve, W. S. Emission from the 1 (n, π*) state of azobenzene: spectrum and ultrashort decay time. *Chem. Phys. Lett.* **46**, 15-19 (1977).

48 Hamm, P., Ohline, S. M. & Zinth, W. Vibrational cooling after ultrafast photoisomerization of azobenzene measured by femtosecond infrared spectroscopy. *J. Chem. Phys.* **106**, 519-529 (1997).

49 Nägele, T., Hoche, R., Zinth, W. & Wachtveitl, J. Femtosecond photoisomerization of cis-azobenzene. *Chem. Phys. Lett.* **272**, 489-495 (1997).

50 Lednev, I. K. *et al.* Femtosecond time-resolved UV-visible absorption spectroscopy of trans-azobenzene: dependence on excitation wavelength. *Chem. Phys. Lett.* **290**, 68-74 (1998).

51 Pancur, T. *et al.* Femtosecond fluorescence up-conversion spectroscopy of a rotation-restricted azobenzene after excitation to the S1 state. *Phys. Chem. Chem. Phys.* **7**, 1985-1989 (2005).

52 Stuart, C. M., Frontiera, R. R. & Mathies, R. A. Excited-state structure and dynamics of cis- and trans-azobenzene from resonance Raman intensity analysis. *J. Phys. Chem. A* **111**, 12072-12080 (2007).

53 Otolski, C. J., Raj, A. M., Ramamurthy, V. & Elles, C. G. Spatial confinement alters the ultrafast photoisomerization dynamics of azobenzenes. *Chem. Sci.* **11**, 9513-9523 (2020).

54 Monti, S., Orlandi, G. & Palmieri, P. Features of the photochemically active state surfaces of azobenzene. *Chem. Phys.* **71**, 87-99 (1982).

55 Cattaneo, P. & Persico, M. An ab initio study of the photochemistry of azobenzene. *Phys. Chem. Chem. Phys.* **1**, 4739-4743 (1999).

56 Ishikawa, T., Noro, T. & Shoda, T. Theoretical study on the photoisomerization of

azobenzene. *J. Chem. Phys.* **115**, 7503-7512 (2001).

57 Ciminelli, C., Granucci, G. & Persico, M. The photoisomerization mechanism of azobenzene: A semiclassical simulation of nonadiabatic dynamics. *Chem. - Eur. J.* **10**, 2327-2341 (2004).

58 Diau, E. W.-G. A new trans-to-cis photoisomerization mechanism of azobenzene on the S1 (n, π*) surface. *J. Phys. Chem. A* **108**, 950-956 (2004).

59 Toniolo, A., Ciminelli, C., Persico, M. & Martínez, T. J. Simulation of the photodynamics of azobenzene on its first excited state: Comparison of full multiple spawning and surface hopping treatments. *J. Chem. Phys.* **123**, 234308 (2005).

60 Crecca, C. R. & Roitberg, A. E. Theoretical study of the isomerization mechanism of azobenzene and disubstituted azobenzene derivatives. *J. Phys. Chem. A* **110**, 8188-8203 (2006).

61 Granucci, G. & Persico, M. Excited state dynamics with the direct trajectory surface hopping method: azobenzene and its derivatives as a case study. *Theor. Chem. Acc.* **117**, 1131-1143 (2007).

62 Conti, I., Garavelli, M. & Orlandi, G. The different photoisomerization efficiency of azobenzene in the lowest nπ* and ππ* singlets: the role of a phantom state. *J. Am. Chem. Soc.* **130**, 5216-5230 (2008).

63 Yuan, S., Dou, Y., Wu, W., Hu, Y. & Zhao, J. Why does trans-azobenzene have a smaller isomerization yield for ππ* excitation than for nπ* excitation? *J. Phys. Chem. A* **112**, 13326-13334 (2008).

64 Böckmann, M., Doltsinis, N. L. & Marx, D. Nonadiabatic hybrid quantum and molecular mechanic simulations of azobenzene photoswitching in bulk liquid environment. *J. Phys. Chem. A* **114**, 745-754 (2010).

65 Cusati, T., Granucci, G. & Persico, M. Photodynamics and time-resolved fluorescence of azobenzene in solution: a mixed quantum-classical simulation. *J. Am. Chem. Soc.* **133**, 5109-5123 (2011).

66 Harabuchi, Y., Ishii, M., Nakayama, A., Noro, T. & Taketsugu, T. A multireference perturbation study of the NN stretching frequency of trans-azobenzene in nπ* excitation and an implication for the photoisomerization mechanism. *J. Chem. Phys.* **138**, 064305 (2013).

67 Casellas, J., Bearpark, M. J. & Reguero, M. Excited-State Decay in the Photoisomerisation of Azobenzene: A New Balance between Mechanisms.

*ChemPhysChem* **17**, 3068-3079 (2016).
68 Tavadze, P. *et al.* A machine-driven hunt for global reaction coordinates of azobenzene photoisomerization. *J. Am. Chem. Soc.* **140**, 285-290 (2018).
69 Aleotti, F. *et al.* Multidimensional potential energy surfaces resolved at the RASPT2 level for accurate photoinduced isomerization dynamics of azobenzene. *J. Chem. Theory Comput.* **15**, 6813-6823 (2019).
70 Yu, J. K., Bannwarth, C., Hohenstein, E. G. & Martínez, T. J. Ab initio nonadiabatic molecular dynamics with hole–hole Tamm–Dancoff approximated density functional theory. *J. Chem. Theory Comput.* **16**, 5499-5511 (2020).
71 Yu, J. K., Bannwarth, C., Liang, R., Hohenstein, E. G. & Martínez, T. J. Nonadiabatic dynamics simulation of the wavelength-dependent photochemistry of azobenzene excited to the nπ* and ππ* excited states. *J. Am. Chem. Soc.* **142**, 20680-20690 (2020).
72 Gu, B. *et al.* Photoisomerization transition state manipulation by entangled two-photon absorption. *Proc. Natl. Acad. Sci. USA* **118**, e2116868118 (2021).
73 Keefer, D. *et al.* Imaging conical intersection dynamics during azobenzene photoisomerization by ultrafast X-ray diffraction. *Proc. Natl. Acad. Sci. USA* **118**, e2022037118 (2021).
74 Keefer, D. *et al.* Diffractive imaging of conical intersections amplified by resonant infrared fields. *J. Am. Chem. Soc.* **143**, 13806-13815 (2021).
75 Liang, R. First-Principles Nonadiabatic Dynamics Simulation of Azobenzene Photodynamics in Solutions. *J. Chem. Theory Comput.* **17**, 3019-3030 (2021).
76 Rouxel, J. R. *et al.* Coupled electronic and nuclear motions during azobenzene photoisomerization monitored by ultrafast electron diffraction. *J. Chem. Theory Comput.* **18**, 605-613 (2022).
77 Cembran, A., Bernardi, F., Garavelli, M., Gagliardi, L. & Orlandi, G. On the Mechanism of the cis−trans Isomerization in the Lowest Electronic States of Azobenzene: S0, S1, and T1. *J. Am. Chem. Soc.* **126**, 3234-3243 (2004).
78 Kabsch, W. A solution for the best rotation to relate two sets of vectors. *Acta Crystallogr. A* **32**, 922-923 (1976).
79 Bondi, A. van der Waals Volumes and Radii. *J. Phys. Chem.* **68**, 441-451 (1964).
