# Supplementary material for: X-ray liquidography decodes complex motions in azobenzene isomerization
Source: Nature. 2026 Sep 30;658(8136):658–65. doi: 10.1038/s41586-026-11068-4 (PMC13668673; doi:10.1038/s41586-026-11068-4)
Supplement: Supplementary file 3 [file 41586_2026_11068_MOESM2_ESM.docx]

**Supplementary Data 1: Combined atomic coordinates of all calculated structures. TRXL-refined structures: I_1_ (Supplementary Data 2), I_2_ (Supplementary Data 3), and *cis*-AB (Supplementary Data 4). CASPT2-calculated structures: *trans*-AB (Supplementary Data 5), I_1_ (Supplementary Data 6), I_2_ (Supplementary Data 7), and *cis*-AB (Supplementary Data 8).**

**Supplementary Data 2: TRXL-refined atomic coordinates of the I1 intermediate. Cartesian coordinates in angstroms (Å). Chemical formula: C12H10N2 (24 atoms). (File: Supplementary Data 2 TRXL-refined I1.xyz)**

| **Atom** | **X (Å)** | **Y (Å)** | **Z (Å)** |
| --- | --- | --- | --- |
| C | 2.31124 | 3.88398 | -0.24986 |
| C | 1.94708 | 3.16445 | -1.40060 |
| C | 1.39382 | 1.87573 | -1.27378 |
| C | 1.20677 | 1.31126 | 0.00406 |
| C | 1.57247 | 2.03314 | 1.16675 |
| C | 2.12203 | 3.31425 | 1.03085 |
| H | 2.74146 | 4.88550 | -0.34253 |
| H | 2.09177 | 3.60146 | -2.39261 |
| H | 1.10124 | 1.29277 | -2.15169 |
| H | 1.42099 | 1.58146 | 2.14926 |
| H | 2.40722 | 3.87865 | 1.92359 |
| C | -1.20676 | -1.31126 | -0.00405 |
| C | -1.39380 | -1.87572 | 1.27381 |
| C | -1.57248 | -2.03314 | -1.16672 |
| C | -1.94707 | -3.16443 | 1.40063 |
| H | -1.10122 | -1.29275 | 2.15172 |
| C | -2.12204 | -3.31425 | -1.03080 |
| H | -1.42102 | -1.58148 | -2.14924 |
| C | -2.31122 | -3.88394 | 0.24990 |
| H | -2.09176 | -3.60146 | 2.39262 |
| H | -2.40725 | -3.87868 | -1.92351 |
| H | -2.74144 | -4.88544 | 0.34259 |
| N | 0.63808 | 0.00001 | 0.00613 |
| N | -0.63808 | -0.00000 | -0.00613 |

**Supplementary Data 3: TRXL-refined atomic coordinates of the I2 intermediate. Cartesian coordinates in angstroms (Å). Chemical formula: C12H10N2 (24 atoms). (File: Supplementary Data 3 TRXL-refined I2.xyz)**

| **Atom** | **X (Å)** | **Y (Å)** | **Z (Å)** |
| --- | --- | --- | --- |
| C | 2.52698 | 1.90007 | -3.22390 |
| C | 2.37255 | 2.58176 | -2.00489 |
| C | 1.45949 | 2.10215 | -1.04611 |
| C | 0.70398 | 0.94201 | -1.31027 |
| C | 0.85653 | 0.25171 | -2.53783 |
| C | 1.76707 | 0.73615 | -3.48545 |
| H | 3.23486 | 2.26823 | -3.97230 |
| H | 2.95825 | 3.48204 | -1.79886 |
| H | 1.31854 | 2.61216 | -0.08893 |
| H | 0.26362 | -0.64552 | -2.72681 |
| H | 1.89108 | 0.20912 | -4.43609 |
| C | -0.70396 | -1.60060 | 0.20566 |
| C | -1.45946 | -1.91986 | 1.35188 |
| C | -0.85654 | -2.35225 | -0.98531 |
| C | -2.37253 | -2.99136 | 1.31789 |
| H | -1.31849 | -1.32035 | 2.25569 |
| C | -1.76710 | -3.41622 | -1.00974 |
| H | -0.26365 | -2.09188 | -1.86449 |
| C | -2.52698 | -3.73955 | 0.13855 |
| H | -2.95824 | -3.23823 | 2.20783 |
| H | -1.89116 | -4.00191 | -1.92539 |
| H | -3.23486 | -4.57296 | 0.10656 |
| N | -0.18812 | 0.55269 | -0.26366 |
| N | 0.18816 | -0.49478 | 0.36084 |

**Supplementary Data 4: TRXL-refined atomic coordinates of cis-azobenzene. Cartesian coordinates in angstroms (Å). Chemical formula: C12H10N2 (24 atoms). (File: Supplementary Data 4 TRXL-refined cis-AB.xyz)**

| **Atom** | **X (Å)** | **Y (Å)** | **Z (Å)** |
| --- | --- | --- | --- |
| C | 2.94840 | -3.52054 | 0.59006 |
| C | 2.82404 | -2.94801 | -0.69084 |
| C | 2.05417 | -1.78451 | -0.87205 |
| C | 1.35761 | -1.23794 | 0.22189 |
| C | 1.50362 | -1.78269 | 1.51449 |
| C | 2.30394 | -2.92537 | 1.69191 |
| N | 0.63446 | -0.00148 | -0.00551 |
| N | -0.63445 | -0.00044 | 0.00569 |
| C | -1.35682 | -1.25782 | 0.00964 |
| C | -2.04632 | -1.59847 | 1.18831 |
| C | -2.81215 | -2.77806 | 1.22495 |
| C | -2.94015 | -3.57337 | 0.06940 |
| C | -2.30401 | -3.18546 | -1.12584 |
| C | -1.50693 | -2.02755 | -1.16249 |
| H | -3.54976 | -4.48090 | 0.09494 |
| H | -3.32451 | -3.06450 | 2.14787 |
| H | -1.96175 | -0.94800 | 2.06400 |
| H | -1.00557 | -1.72444 | -2.08560 |
| H | -2.42574 | -3.78645 | -2.03184 |
| H | 1.97280 | -1.30364 | -1.85137 |
| H | 0.99666 | -1.31943 | 2.36502 |
| H | 3.34254 | -3.39461 | -1.54409 |
| H | 2.42217 | -3.35217 | 2.69204 |
| H | 3.56134 | -4.41508 | 0.73231 |

**Supplementary Data 5: CASPT2-calculated atomic coordinates of trans-azobenzene. Cartesian coordinates in angstroms (Å). Chemical formula: C12H10N2 (24 atoms). (File: Supplementary Data 5 CASPT2-calculated trans-AB.xyz)**

| **Atom** | **X (Å)** | **Y (Å)** | **Z (Å)** |
| --- | --- | --- | --- |
| C | -5.82673 | -2.85186 | -0.00146 |
| C | -4.42188 | -2.82151 | 0.00092 |
| C | -3.74902 | -1.58448 | 0.00192 |
| C | -4.48411 | -0.38193 | 0.00053 |
| C | -5.90045 | -0.40794 | -0.00186 |
| C | -6.56105 | -1.64299 | -0.00284 |
| H | -6.35459 | -3.81003 | -0.00224 |
| H | -3.85049 | -3.75398 | 0.00200 |
| H | -2.65666 | -1.53047 | 0.00377 |
| H | -6.45495 | 0.53270 | -0.00291 |
| H | -7.65467 | -1.67215 | -0.00469 |
| C | -3.59460 | 3.06939 | 0.00164 |
| C | -4.32970 | 4.27195 | 0.00025 |
| C | -2.17827 | 3.09541 | 0.00404 |
| C | -3.65683 | 5.50897 | 0.00124 |
| H | -5.42206 | 4.21795 | -0.00160 |
| C | -1.51767 | 4.33046 | 0.00502 |
| H | -1.62375 | 2.15478 | 0.00510 |
| C | -2.25200 | 5.53930 | 0.00363 |
| H | -4.22818 | 6.44146 | 0.00016 |
| H | -0.42406 | 4.35965 | 0.00687 |
| H | -1.72415 | 6.49745 | 0.00441 |
| N | -3.69252 | 0.80811 | 0.00174 |
| N | -4.38619 | 1.87936 | 0.00044 |

**Supplementary Data 6: CASPT2-calculated atomic coordinates of the I1 intermediate. Cartesian coordinates in angstroms (Å). Chemical formula: C12H10N2 (24 atoms). (File: Supplementary Data 6 CASPT2-calculated I1.xyz)**

| **Atom** | **X (Å)** | **Y (Å)** | **Z (Å)** |
| --- | --- | --- | --- |
| C | -4.58736 | -0.40621 | 0.15063 |
| C | -4.11582 | 0.92343 | 0.03667 |
| C | -2.74938 | 1.19316 | -0.06921 |
| C | -1.81758 | 0.11136 | -0.05391 |
| C | -2.28566 | -1.23758 | 0.06007 |
| C | -3.65966 | -1.47346 | 0.16034 |
| N | -0.48813 | 0.39972 | -0.16962 |
| N | 0.48697 | -0.40521 | -0.18261 |
| C | 1.81748 | -0.11471 | -0.08125 |
| C | 2.75290 | -1.19232 | -0.12617 |
| C | 4.11955 | -0.91852 | -0.03504 |
| C | 4.58784 | 0.41107 | 0.09267 |
| C | 3.65649 | 1.47431 | 0.13151 |
| C | 2.28236 | 1.23416 | 0.04698 |
| H | 5.65959 | 0.61464 | 0.16018 |
| H | 4.83345 | -1.74677 | -0.06572 |
| H | 2.38116 | -2.21570 | -0.22753 |
| H | 1.55128 | 2.04701 | 0.07530 |
| H | 4.01141 | 2.50494 | 0.23052 |
| H | -2.37451 | 2.21636 | -0.16043 |
| H | -1.55822 | -2.05410 | 0.06611 |
| H | -4.82681 | 1.75479 | 0.02839 |
| H | -4.01731 | -2.50402 | 0.24886 |
| H | -5.65893 | -0.60687 | 0.23012 |

**Supplementary Data 7: CASPT2-calculated atomic coordinates of the I2 intermediate. Cartesian coordinates in angstroms (Å). Chemical formula: C12H10N2 (24 atoms). (File: Supplementary Data 7 CASPT2-calculated I2.xyz)**

| **Atom** | **X (Å)** | **Y (Å)** | **Z (Å)** |
| --- | --- | --- | --- |
| C | -4.22266 | -0.47050 | 0.79195 |
| C | -4.00352 | 0.80608 | 0.22941 |
| C | -2.77092 | 1.12598 | -0.35394 |
| C | -1.72731 | 0.16075 | -0.35693 |
| C | -1.94415 | -1.13393 | 0.20143 |
| C | -3.18851 | -1.43200 | 0.77107 |
| N | -0.52524 | 0.50363 | -0.94763 |
| N | 0.47024 | -0.28916 | -1.05878 |
| C | 1.70439 | -0.07148 | -0.47526 |
| C | 2.74003 | -1.01149 | -0.72991 |
| C | 4.00419 | -0.81529 | -0.15965 |
| C | 4.26205 | 0.31663 | 0.64431 |
| C | 3.23503 | 1.25701 | 0.87991 |
| C | 1.95992 | 1.07909 | 0.32843 |
| H | 5.25390 | 0.46706 | 1.07950 |
| H | 4.79787 | -1.54452 | -0.34684 |
| H | 2.52523 | -1.87782 | -1.36206 |
| H | 1.15288 | 1.79877 | 0.49532 |
| H | 3.43319 | 2.13604 | 1.50081 |
| H | -2.58670 | 2.10661 | -0.80195 |
| H | -1.13246 | -1.86691 | 0.17103 |
| H | -4.80308 | 1.55254 | 0.24108 |
| H | -3.35675 | -2.42259 | 1.20460 |
| H | -5.19038 | -0.71548 | 1.23840 |

**Supplementary Data 8: CASPT2-calculated atomic coordinates of cis-azobenzene. Cartesian coordinates in angstroms (Å). Chemical formula: C12H10N2 (24 atoms). (File: Supplementary Data 8 CASPT2-calculated cis-AB.xyz)**

| **Atom** | **X (Å)** | **Y (Å)** | **Z (Å)** |
| --- | --- | --- | --- |
| C | -3.07741 | -1.15837 | -0.28971 |
| C | -3.29239 | 0.06649 | 0.37170 |
| C | -2.36778 | 1.11724 | 0.22921 |
| C | -1.19661 | 0.91150 | -0.52357 |
| C | -0.98921 | -0.29829 | -1.21803 |
| C | -1.93683 | -1.33041 | -1.09787 |
| N | -0.31152 | 2.04781 | -0.69354 |
| N | 0.93264 | 1.91693 | -0.48099 |
| C | 1.44562 | 0.67136 | 0.05589 |
| C | 2.55525 | 0.10038 | -0.59482 |
| C | 3.12948 | -1.07579 | -0.07893 |
| C | 2.63818 | -1.63637 | 1.11630 |
| C | 1.57314 | -1.01665 | 1.79849 |
| C | 0.96901 | 0.13873 | 1.27164 |
| H | 3.09684 | -2.54107 | 1.52498 |
| H | 3.97194 | -1.54179 | -0.59801 |
| H | 2.94346 | 0.57189 | -1.50261 |
| H | 0.13791 | 0.62096 | 1.79327 |
| H | 1.20918 | -1.43434 | 2.74184 |
| H | -2.53297 | 2.08818 | 0.70556 |
| H | -0.09933 | -0.42790 | -1.83959 |
| H | -4.18916 | 0.21097 | 0.98102 |
| H | -1.78244 | -2.27079 | -1.63476 |
| H | -3.80591 | -1.96821 | -0.19183 |
